# LC-MS-based metabolomics reveals the mechanism of anti-gouty arthritis effect of Wuwei Shexiang pill

**DOI:** 10.3389/fphar.2023.1213602

**Published:** 2023-08-11

**Authors:** Jirui Lang, Li Li, Yunyun Quan, Ruirong Tan, Jinbiao Zhao, Min Li, Jin Zeng, Shilong Chen, Ting Wang, Yong Li, Junning Zhao, Zhujun Yin

**Affiliations:** ^1^ Biological Assay Key Laboratory of State Administration of Traditional Chinese Medicine for Traditional Chinese Medicine Quality, Translational Chinese Medicine Key Laboratory of Sichuan Province, Sichuan Engineering Technology Research Center of Genuine Regional Drug, Engineering Research Center for Formation Principle and Quality Evaluation of Genuine Medicinal Materials in Sichuan Province, Sichuan Academy of Chinese Medicine Sciences, Sichuan Institute for Translational Chinese Medicine, Chengdu, China; ^2^ Hunan Provincial Key Laboratory of the Research and Development of Novel Pharmaceutical Preparations, The “Double-First Class” Application Characteristic Discipline of Hunan Province (Pharmaceutical Science), Changsha Medical University, Changsha, China; ^3^ Sichuan Fengchun Pharmaceutical Co., Ltd., Deyang, China

**Keywords:** Wuwei Shexiang pill (WSP), gout, serum metabolomics, linoleic acid metabolism, phenylalanine metabolism, pantothenate and CoA biosynthesis

## Abstract

Wuwei Shexiang Pill (WSP) is a Tibetan traditional medicine, which has been demonstrated to exhibit potent anti-inflammatory and anti-gout effects. However, the specific pharmacological mechanism is not elucidated clearly. In the present study, liquid chromatography-mass spectrometry (LC-MS)-based metabolomics was applied to investigate the alteration of serum metabolites induced by WSP treatment in MSU-induced gouty rats. Subsequently, bioinformatics was utilized to analyze the potential metabolic pathway of the anti-gout effect of WSP. The pharmacodynamic data discovered that WSP could ameliorate ankle swelling and inflammatory cell infiltration, as well as downregulate the protein expression of IL-1β, p-NF-κB p65, and NLRP3 in the synovial membrane and surrounding tissues of gouty ankles. LC-MS-based metabolomics revealed that there were 30 differential metabolites in the serum between sham-operated rats and gouty ones, which were mainly involved in the metabolism of fructose and mannose, primary bile acid biosynthesis, and cholesterol metabolism. However, compared to the model group, WSP treatment upregulated 11 metabolic biomarkers and downregulated 31 biomarkers in the serum. KEGG enrichment analysis found that 27 metabolic pathways contributed to the therapeutic action of WSP, including linoleic acid metabolism, phenylalanine metabolism, and pantothenate and CoA biosynthesis. The comprehensive analysis-combined network pharmacology and metabolomics further revealed that the regulatory network of WSP against gout might be attributed to 11 metabolites, 7 metabolic pathways, 39 targets, and 49 active ingredients of WSP. In conclusion, WSP could ameliorate the inflammation of the ankle in MSU-induced gouty rats, and its anti-gout mechanism might be relevant to the modulation of multiple metabolic pathways, such as linoleic acid metabolism, phenylalanine metabolism, and pantothenate and CoA biosynthesis. This study provided data support for the secondary development of Chinese traditional patent medicine.

## 1 Introduction

Gout is a common inflammatory arthritis that is caused by monosodium urate (MSU) crystal deposition in the articular structure of the joint of a person who suffers from hyperuricemia (HUA) HUA is a metabolic disorder characterized by increased production and/or decreased excretion of serum uric acid due to purine metabolism dysfunction (FitzGerald et al., 2020). Then, long-term high levels of uric acid in the blood may lead to the precipitation and deposition of MSU crystals, triggering an innate immune response and intolerably painful arthritis, which is known as gout flares. Clinically, gouty arthritis (GA) is characterized by redness, swelling, and pain in the lower limb joints, especially in the first metatarsophalangeal joint, which can even affect physical function (Dalbeth et al., 2021). Although most patients with HUA will not develop gout in their lifetime, the incidence rate of gout is rising year by year and showing a trend in a younger age group (Danve and Neogi, 2020; Rao et al., 2022). Unfortunately, the development of medical strategies for treating gout is unsatisfactory. First-line anti-inflammatory and uric acid-lowering drugs have shown various side effects and safety problems (Jansen et al., 2004; MacDonald et al., 2014; Wechalekar et al., 2014; Ragab et al., 2017). A promising small molecule compound MCC950, which specifically targets NLRP3, was suspended in phase II clinical trials because of its liver toxicity (Mangan et al., 2018). Several IL-1 antagonists (Anakinra, Canakinumab, and Rilonacept) have been progressively applied in the treatment of refractory and recurrent gouty arthritis, which was recommended by the European League Against Rheumatism (EULAR) and the American College of Rheumatology (ACR) (Arnold et al., 2022). However, these drugs are not yet available in China. Hence, research for safer and more reliable anti-gout drugs is imperative.

Traditional Chinese medicine (TCM) has received widespread attention and is used clinically due to its preferable effectiveness and safety (Wen et al., 2021; Zhou et al., 2022). Chinese traditional patent medicine Wuwei Shexiang pill (WSP), composed of *Terminalia chebula* Retz., *Aconitum pendulum* Busch, *Aucklandia lappa* Decne., *Acorus calamus* L., and artificial musk, has been used to treat various types of arthritis in Tibetan-populated areas of China for centuries (Chinese Pharmacopoeia Commission, 2020). Recently, WSP has also been reported to significantly reduce serum uric acid levels and inhibit ear swelling in mice (Yamin and Lvyi, 2017). In our previous study, we confirmed that WSP exerted an anti-gout effect by inhibiting MAPK, NF-κB, and NLRP3 signaling pathways in MSU-stimulated THP-1 macrophages (Lang et al., 2022). Although our research has unveiled the anti-inflammatory effect and mechanism *in vitro*, the anti-gout pharmacodynamic activity and the metabolomic profiles of WSP *in vivo* have not been reported.

Metabolomics is a systematic approach to analyzing the small molecules metabolites in biological samples and unveiling the underlying mechanisms of altered endogenous metabolites in physiological and pathological states or stimulated by exogenous substances (Fu et al., 2023). Untargeted metabolomics is used to analyze changes in biological endogenous metabolites and enrich for metabolic pathways via high-throughput assays, which is widely used in the fields of pharmacology, drug toxicology, modernization of Chinese medicine, and so on (Li et al., 2021; Wang and Sun, 2022; Wang et al., 2023; Huang et al., 2023). Recently, a large number of works of literature have documented that metabolomics was a novel and high-performance strategy to screen the predictive biomarkers of gout flares and to unveil the molecular mechanism of anti-GA TCMs (Huang et al., 2019; Lyu et al., 2019; Wang and Sun, 2022; Gu et al., 2023; Lei et al., 2023). Huang et al. proposed that the metabolomics signatures of gout sufferings in the serum are mainly comprised of several metabolic pathways, including purine metabolism, branched-chain amino acids metabolism, and the tricarboxylic acid cycle and bile secretion and arachidonic acid metabolism (Huang et al., 2020). Notably, in consideration of the sensitivity of LC-MS-based metabolomics, the subtle changes in metabolites of biospecimen can be detected, which is conducive to revealing the metabolic pathways of TCMs.

In this study, a rat model of gouty arthritis induced by intra-articular injection of MSU was adopted to evaluate the anti-gout effect of WSP. Subsequently, the metabolomic profile on the effect of WSP in the GA rats was monitored by LC-MS-based metabolomics, thus revealing the anti-GA metabolomic pathways and the molecular mechanisms of WSP.

## 2 Materials and methods

### 2.1 Regents and drugs

All the materials were obtained from the suppliers as follows: PBS (Lot#8122153) (Gibco, NY, United States); uric acid (Lot#BCCC5658) (Sigma-Aldrich, Darmstadt, GER); Etoricoxib tablets (Lot#U039558) (J20180059) (Merck and Co., Inc., NJ, United States); WSP (Lot#2205001) (Z51020967) (Jiuzhaigou Natural Pharmaceutical Group Co., Ltd., Aba, CHN); 2-Chlorophenylalanine (Lot#20211126), Ammonium formate (Lot#T2122203) (Aladdin, Shanghai, CHN); Acetonitrile (Lot#R142221) (Dikma, CA, United States); formic acid (Lot#20221214) (TCI, Shanghai, China); Anti-IL-1β antibody (Lot#BB09202951), Anti-p-NF-κB P65 antibody (Lot# BB05301615), and Anti-NLRP3 antibody (Lot#BA12271673) (Bioss, Beijing, CHN).

Preparation of MSU suspension: A total measurement of 168.1 mg of uric acid and 818.6 mg of NaCl was dissolved in 100 mL of boiling water, and 1.25 mL of 1 mol/L NaOH was added, heated, and stirred until fully dissolved. The solution was left at room temperature for 2–3 days. The MSU crystal was obtained after filtering and drying and was sterilized at 180°C for 2 h. Subsequently, it was weighed in a clean area and prepared with sterile PBS as an MSU suspension (12 mg/mL).

### 2.2 Animals and treatments

A total of 66 male-specific pathogen-free Sprague-Dawley rats (6∼8 weeks, 280∼300 g) were purchased from Hunan Slake Jingda Experimental Animal Co., Ltd (License number: SCXK (Xiang) 2019-0004). After 1 week of acclimatization, the rats were randomly divided into six groups including the control group (CON), model group (MOD), WSP low-dosage group (10 mg/kg, WSP-L), WSP medium-dosage group (20 mg/kg, WSP-M), WSP high-dosage group (40 mg/kg, WSP-H), and Etoricoxib group (32 mg/kg, ETO), which were orally administrated vehicle or drugs once a day for 7 days, respectively. All the animals were housed under the standard condition with regulated temperature and humidity with an alternating 12-h light/12-h dark cycle, allowing them to take food and water freely. After the experiment, the rats were sacrificed by asphyxiation with CO_2_ (KW-AL experimental animal euthanasia device, Nanjing Calvin Biotech. Co., Ltd., Nanjing, CHN), which was delivered into the cage for less than 5 psi per second. The death of the rats was confirmed by a lack of respiration and consciousness. All the experimental procedures were approved by the Experimental Animal Ethics Committee of the Sichuan Academy of Chinese Traditional Medicine [Grant number: SYLL (2021)-031].

### 2.3 Acute gouty arthritis model

After 1 h of administration on D 6, the animals were anesthetized with 1% sodium pentobarbital solution (40 mg/kg) i. p. When the absence of the righting reflex and stable respiration was observed, acute gouty arthritis in the rats was established according to Coderre’s approach (Coderre and Wall, 1987). Briefly, a syringe was inserted from the dorsum of the animal’s left ankle joint, and 0.1 mL of the prepared MSU suspension (12 mg/mL) was injected into the articular cavity to create the GA model, while the CON group was administrated with 0.1 mL sterile PBS. After 1 h of administration on the last day, rats were anesthetized with 1% pentobarbital sodium solution (40 mg/kg) i. p., and the blood was collected through the abdominal aorta, which was allowed to stand at a room temperature for 30 min. The serum was obtained by centrifugation at 4°C, 1500× *g* for 10 min, dispensed into sterile tubes, and quickly transferred to a −80°C refrigerator after touching the bottom of the tubes to change to liquid nitrogen for quick freezing. After the animals were sacrificed using CO_2_, they were placed on an ice plate to quickly separate the joint ankle and surrounding tissue samples and were put into 10% paraformaldehyde for fixation.

### 2.4 Measurement of foot swelling

A line was drawn approximately 1 cm above the left ankle joint of the rats before modeling, and the foot was measured and recorded along the line using a foot measuring instrument (TECHMAN, Chengdu, CHN) at 0 h, 2 h, 4 h, 6 h, 8 h, and 24 h. Foot swelling was calculated as follows:

$$\text{Foot swelling }\left(\text{mL}\right)=\text{Foot volume after modeling }\left(\text{mL}\right)-\text{Foot volume at }0\;h\;\left(\text{mL}\right)$$

### 2.5 Histopathology

The ankle joint and surrounding tissues were dehydrated, embedded in paraffin, and cut into 3 μm thick slices. Slice samples were stained by hematoxylin-eosin staining, and the pathological changes of the ankle joint, synovial membrane, and surrounding tissues were observed under a light microscope (Nikon, Tokyo, JPN). At least three areas were randomly selected to be photographed. The pathologists who were blinded to the experiment uniformly scored each photo, and the scoring criteria which was set by our research team are shown in Table 1, (Zhu-jun et al., 2021).

**TABLE 1:** Pathology scoring criteria.

Grade	Description
0	The surface of the cartilage is flat and intact, with a clear hierarchy of synovial tissue and no inflammatory cell infiltration in the synovium and its surrounding tissue
1	The surface of the cartilage is discontinuous, the synovial tissue is edematous, and the capillaries are dilated, congested, and infiltrated with a few inflammatory cells
2	The surface of the cartilage is damaged and the synovial tissue is edematous, congested, and infiltrated with a large number of inflammatory cells
3	The cartilage is severely damaged and the membrane tissue structure is disturbed, edematous, congested, and infiltrated with a large number of inflammatory cells

### 2.6 Immunohistochemistry

The ankle joint thick slices were dewaxed, antigen-retrieved, immersed in 3% H_2_O_2_, and incubated for 25 min at room temperature and protected from light. Then, the tissues were blocked in 3% BSA for 25 min. Subsequently, primary antibodies (p-NF-κB p65, NLRP3, and IL-1β, dilution: 1:100) were added to the tissues overnight at 4°C Secondary antibody was added for incubation for 50 min at room temperature. DAB solution was used for color reaction; the positive protein expression was brown. Hematoxylin was used for the staining of the nucleus, which showed as blue. The slices were observed under a light microscope (Nikon, Tokyo, JPN) after dehydration and sealing. At least three areas were randomly selected to be photographed. Immunohistochemical images were processed by ImageJ software (NIH and LOCI, United States) to calculate the positive areas.

### 2.7 Metabolomics analysis

#### 2.7.1 Metabolic biomarkers collection

The serum samples were taken from the liquid nitrogen and slowly melted at 4°C and vortexed. After centrifugation for 10 min (12,000 rpm, 4°C), the supernatant was carefully dried, and then 150 µL of a 2-chlorophenylalanine solution prepared with 80% methanol (v:v = 4:1) was added to resolubilize the samples. The supernatant was filtered using a 0.22 μm microporous membrane and then added to the assay vial, pending further analysis (Demurtas et al., 2021).

#### 2.7.2 QC samples

To correct for bias and systematic errors, 10 μL of each group sample were mixed as QC samples, which were tested once for every 10 samples.

#### 2.7.3 Liquid chromatography conditions

The LC analysis was performed on an ACQUITY UPLC System (Waters, Milford, MA, United States). Chromatography was carried out with an ACQUITY UPLC ^®^ HSS T3 (150 × 2.1 mm, 1.8 µm) (Waters, Milford, MA, United States). The flow rate and injection volume were set at 0.25 mL/min and 2 μL, respectively, and the column temperature was set at 40°C. Gradient elution procedures are shown in Table 2 (Zelena et al., 2009).

**TABLE 2:** Gradient elution procedures.

Time (min)	Mobile phase				
	Positive mode			Negative mode	
	0.1% formic acid in water (%)	0.1% formic acid in acetonitrile (%)		5 mM ammonium formate (%)	Acetonitrile (%)
0–1	98	2		98	2
1–9	98–50	2–50		98–50	2–50
9–12	50–2	50–98		50–2	50–98
12–13.5	2	98		2	98
13.5–14	2–98	98–2		2–98	98–2
14–20	98	2		98	2

#### 2.7.4 Mass spectrum conditions

Mass spectrometric detection of metabolites was performed on Q Exactive (Thermo Fisher Scientific, United States) with an ESI ion source. Acquisition parameters were set as follows: spray voltage was 3.50 kV and −2.50 kV for positive mode and negative mode, sheath gas pressure was 30 arb, and auxiliary gas pressure was 10 arb. The primary scan was performed at 325°C in the range of m/z 100–1000 in the capillary tube. Then, secondary cleavage was performed using HCD at a collision energy of 30 eV and a secondary resolution of 17,500 (Want et al., 2013).

#### 2.7.5 Data processing

The raw data acquired from mass spectrometry was conversed into *mzXML files by the Proteowizard software package (v3.0.8789) (Smith et al., 2006). The R XCMS software package was used for peak detection, peak filtering, and peak alignment processing to obtain the substances quantified list with parameters set to bw = 2, ppm = 15, peakwidth = *c* (5, 30), mzwid = 0.015, mzdiff = 0.01, and method = “centWave” (Navarro-Reig et al., 2015). Public databases such as HMDB (Wishart et al., 2022), massbank (Horai et al., 2010), and LipidMaps (Sud et al., 2007) were used to perform calibration tests on metabolites. QC samples were corrected for data according to the method reported previously (Gagnebin et al., 2017), and QC samples with RSD >30% will be discarded for quality control.

#### 2.7.6 Pathway analysis

The MetaboAnalyst software package was used to perform functional pathway enrichment and topological analysis for screening metabolic biomarkers. The KEGG Mapper tool was used to visualize the pathways (Xia and Wishart, 2011).

### 2.8 The comprehensive analysis combined with network pharmacology

Based on our previous network pharmacology analysis study, we combined the metabolomics results with a network pharmacology approach for further comprehensive analysis and a metabolites-pathways-targets-ingredient network was constructed using Cytoscape 3.7.2 software to determine the underlying mechanism of WSP.

### 2.9 Statistics analysis

All data were expressed as the mean ± standard deviation. One-way analysis of variance (ANOVA) was used for multifactorial comparisons using GraphPad Prism 9 (GraphPad Software, Inc., CA, United States). Immunohistochemical images were processed by ImageJ software (NIH and LOCI, United States) to calculate the positive areas. Pathology scoring grades were performed with the Mann-Whitney *U* test using SPSS (IBM, NY, United States). The *p*-value <0.05 was considered statistically significant.

## 3 Results

### 3.1 WSP inhibits foot swelling in MSU-induced acute gouty arthritis in rats

**FIGURE 1:**
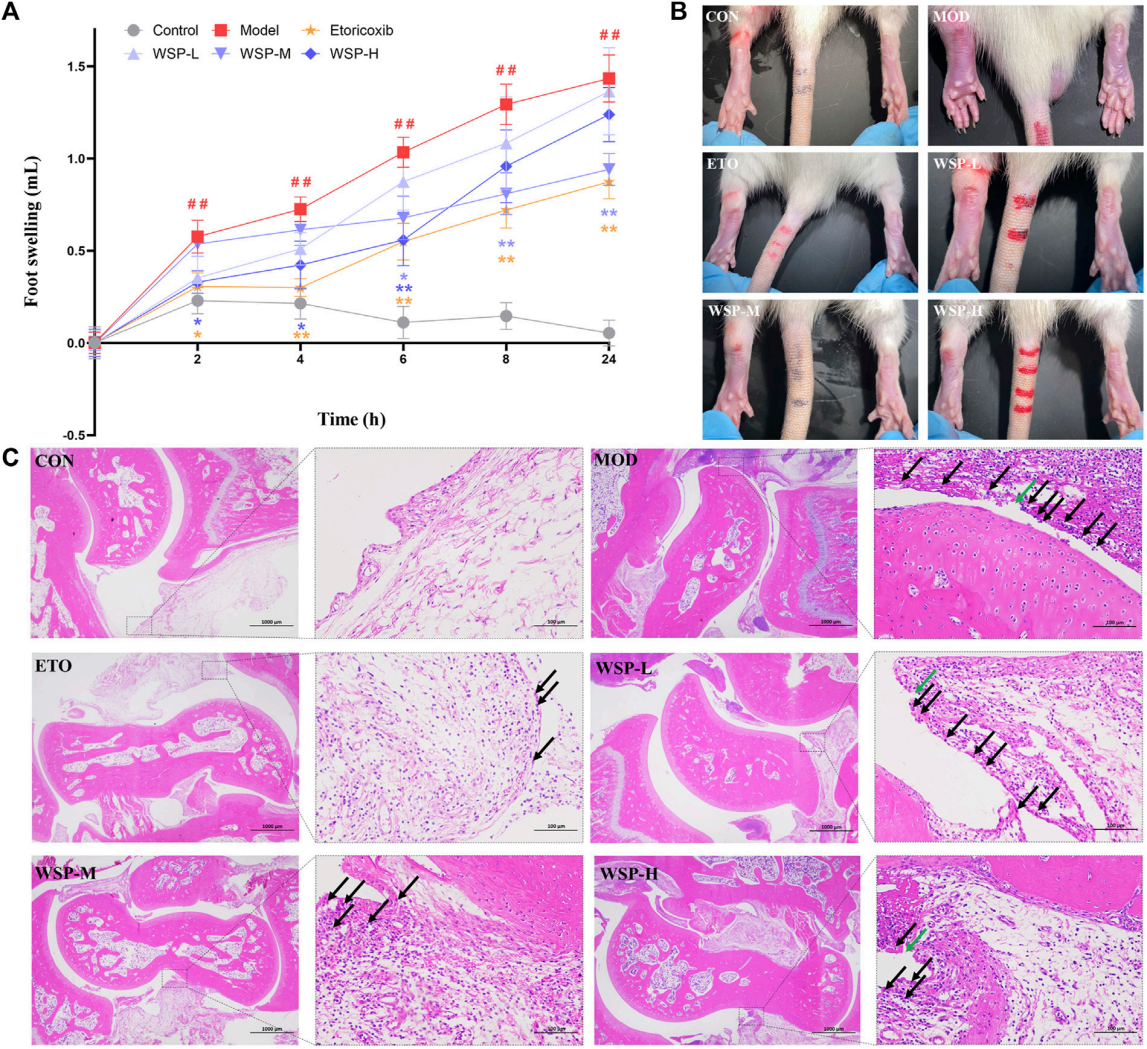
Foot swelling and pathological assay. **(A,B)** Foot swelling in each group at each time point. **(C)** Histopathology staining with HE (left 20×, right 200×). Black arrows indicate the infiltrated inflammatory cells and green arrows indicate the structurally discontinuous synovial cell layer.

As shown in Figure 1A, B and Table 3, all groups except the CON group showed significant ankle swelling after receiving MSU crystal injection. Compared with the CON group, the foot swelling of the model rats gradually increased with time, demonstrating statistical significance (*p* < 0.01), and the MSU-induced ankle swelling could be maintained until 24 h. The inhibition of foot swelling was observed at 2 h in the ETO group, with significant inhibitions at every subsequent time point. Compared with the MOD group, the WSP-H group primarily showed a prominent therapeutic effect from 2 h to 6 h after MSU injection, while the WSP-M group exhibited remarkable inhibition of foot swelling from 6 h to 24 h. However, there was no conspicuous effect observed in the ankle swelling of WSP-L rats.

**TABLE 3:** The effect of each group on foot swelling in GA model rats ( $\bar{x}\pm S$ , *n* = 8–11).

Group	n	Dose (mg/kg×*d*)	Foot swelling (mL)					
			0 h	2 h	4 h	6 h	8 h	24 h
CON	9	-	0.00 ± 0.26	0.23 ± 0.21	0.21 ± 0.26	0.11 ± 0.26	0.15 ± 0.22	0.05 ± 0.21
MOD	11	-	0.00 ± 0.19	0.58 ± 0.29^##^	0.72 ± 0.22^##^	1.03 ± 0.27^##^	1.29 ± 0.36^##^	1.43 ± 0.42^##^
WSP-L	8	10 × 7	0.00 ± 0.20	0.36 ± 0.33	0.51 ± 0.25	0.88 ± 0.44	1.08 ± 0.71	1.37 ± 0.67
WSP-M	9	20 × 7	0.00 ± 0.12	0.54 ± 0.20	0.62 ± 0.25	0.68 ± 0.35^b^	0.81 ± 0.34^c^	0.94 ± 0.26^c^
WSP-H	9	40 × 7	0.00 ± 0.21	0.33 ± 0.17^b^	0.42 ± 0.37^b^	0.56 ± 0.42^c^	0.96 ± 0.59	1.24 ± 0.44
ETO	9	32 × 7	0.00 ± 0.10	0.30 ± 0.22^b^	0.30 ± 0.15^c^	0.55 ± 0.30^c^	0.72 ± 0.29^c^	0.87 ± 0.27^c^

^a^ p < 0.05 ,^##^ p < 0.01

vs CON.

^b^ p < 0.05 .

^c^ p < 0.01 vs MOD.

### 3.2 WSP attenuated ankle joint changes in gouty rats

As shown in Figure 1C, HE pathological section analysis indicated the pathomorphological features of the synovial membrane and its surrounding tissues of the joint. The cartilage surface of the CON group appeared smooth and intact; the structure of synoviocytes was clear, and there was no apparent infiltration of inflammatory cells found in the synovial and surrounding tissues. In contrast, in the joint samples from the MOD group, the synovial cell layer was seen to have structural discontinuity (green arrows), and the synovial tissue structure was damaged and infiltrated by a large number of inflammatory cells (black arrows). Furthermore, the Mann-Whitney *U* test analysis showed that the average score of the ankle in gouty rats was much higher than that in the pseudosurgical rats, as illustrated in Table 4. On the contrary, WSP administration could alleviate the pathological lesions of the rats to significant degrees, which is displayed in Figure 1C and Table 4. In the WSP-H group, the articular cartilage surface was smooth and flat, and only a small amount of inflammatory cell infiltration was seen in the synovial and surrounding tissues. The above results suggested that MSU could cause serious damage to the joint synovial tissue. However, WSP treatment exhibited a protective effect on the gouty ankle induced by MSU, which is characterized by the improvement of the pathological changes and the reduction of the infiltrated inflammatory cells.

**TABLE 4:** Effect of WSP on the histopathological scoring of the ankle in gouty rats ( $\bar{x}\pm S$ , *n* = 8–11).

Group	Dose (mg/kg ×*d*)	n	Grades (samples)				Weighted average score
			0	1	2	3	
CON	-	9	6	3	0	0	0.3
MOD	-	11	0	0	8	3	2.3^a^
WSP-L	10 × 7	9	0	3	6	0	1.7^b^
WSP-M	20 × 7	8	0	4	4	0	1.5^c^
WSP-H	40 × 7	9	0	2	7	0	1.8^b^
ETO	32 × 7	9	0	5	4	0	1.4^c^

^a^ p < 0.01 vs CON.

^b^ p < 0.05 .

^c^ p < 0.01 vs MOD (Mann-Whitney *U* test).

### 3.3 WSP downregulated the expression of GA-related proteins in the synovium and surrounding tissues of the rats after MSU injection

**FIGURE 2:**
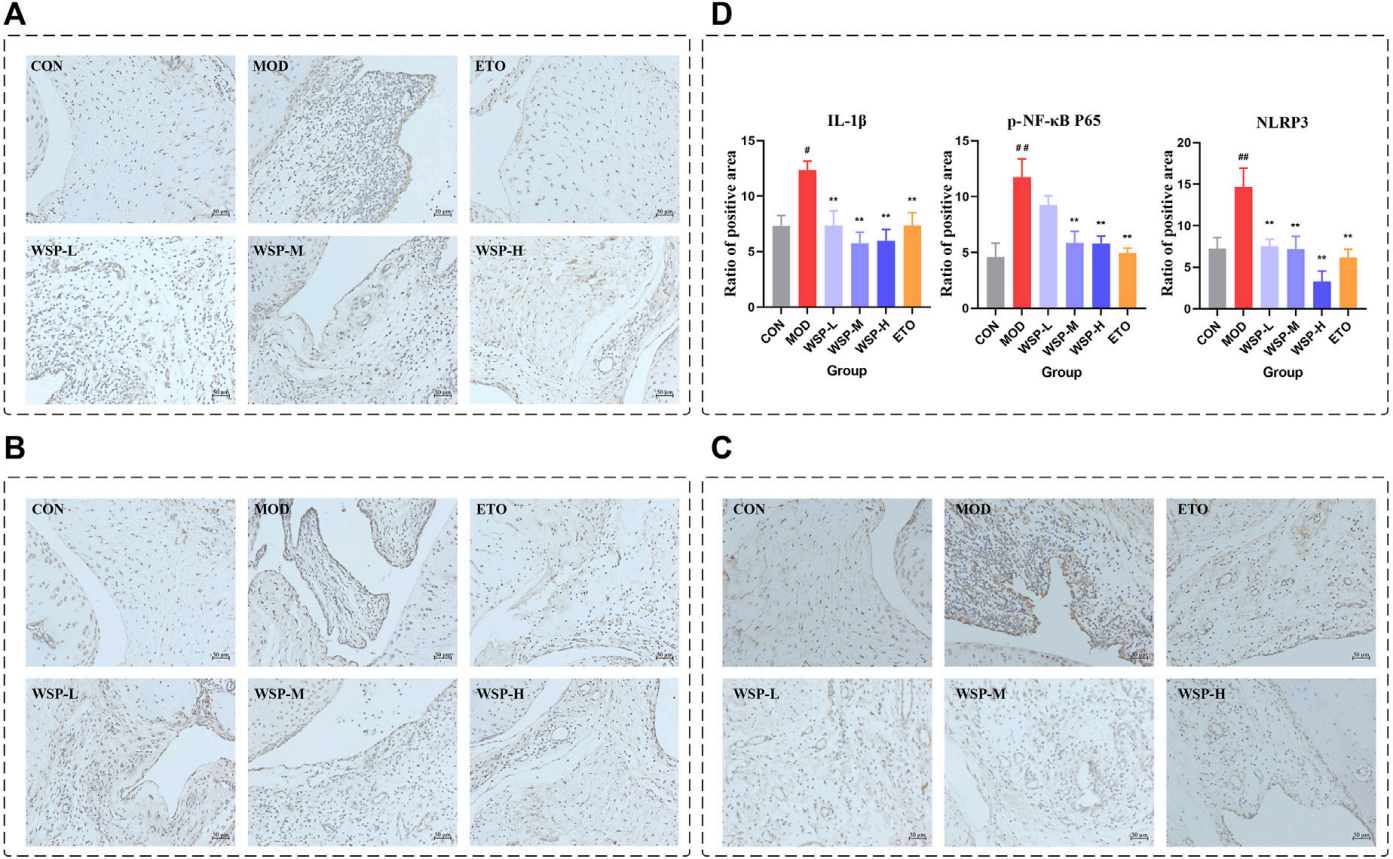
Immunohistochemistry. **(A)** IHC staining of IL-1β (200×). **(B)** IHC staining of p-NF-κB P65 (200×). **(C)** IHC staining of NLRP3 (200×). **(D)** Statistics of the ratio of positive area.

In gout flares, the initiation of NLRP3 inflammasome was considered a key step (Dalbeth et al., 2021). NLRP3 inflammasome is closely related to NF-κB signaling, which regulates the gene expression of all components of inflammasome assembly and activation (Joosten et al., 2010). Activated NLRP3 inflammasome signaling ultimately leads to the production and release of IL-1β, triggering a local inflammatory response. As shown in Figure 2, the protein expression of IL-1β, p-NF-κB P65, and NLRP3 in the synovium and surrounding tissues of the MOD group after MSU injection was significantly increased compared with the CON group, while the expression of all three proteins was downregulated by WSP treatment.

### 3.4 Effects of WSP on the metabolic pathways of MSU-induced acute gouty arthritis model rats

#### 3.4.1 BPC, TIC, and QC

The base peak chromatogram (BPC) depicts the continuous spectrum formed by the maximum ion intensity at different time points during the continuous scan of the mass spectrometry. In both positive and negative modes, all samples showed good peak shapes, intensity, and obvious peak separation, and the general trend of the base peaks in each group was similar, indicating good reproducibility and reliable results (Supplementary File S1). Meanwhile, there were visible differences in the number, intensity, and type of peaks in each group, suggesting differences in the types and amounts of metabolites in the samples. Total ion chromatogram (TIC) records the total ion signal generated by the metabolites, which reflects the overall information of the sample as well as BPC (shown in Supplementary File S1), and the distribution of QC samples (red dots) almost completely overlaps, indicating that the systematic error of this experiment is small, the experiment is reproducible, and the results are reliable (Supplementary File S1).

#### 3.4.2 Multivariate statistical analysis

**FIGURE 3:**
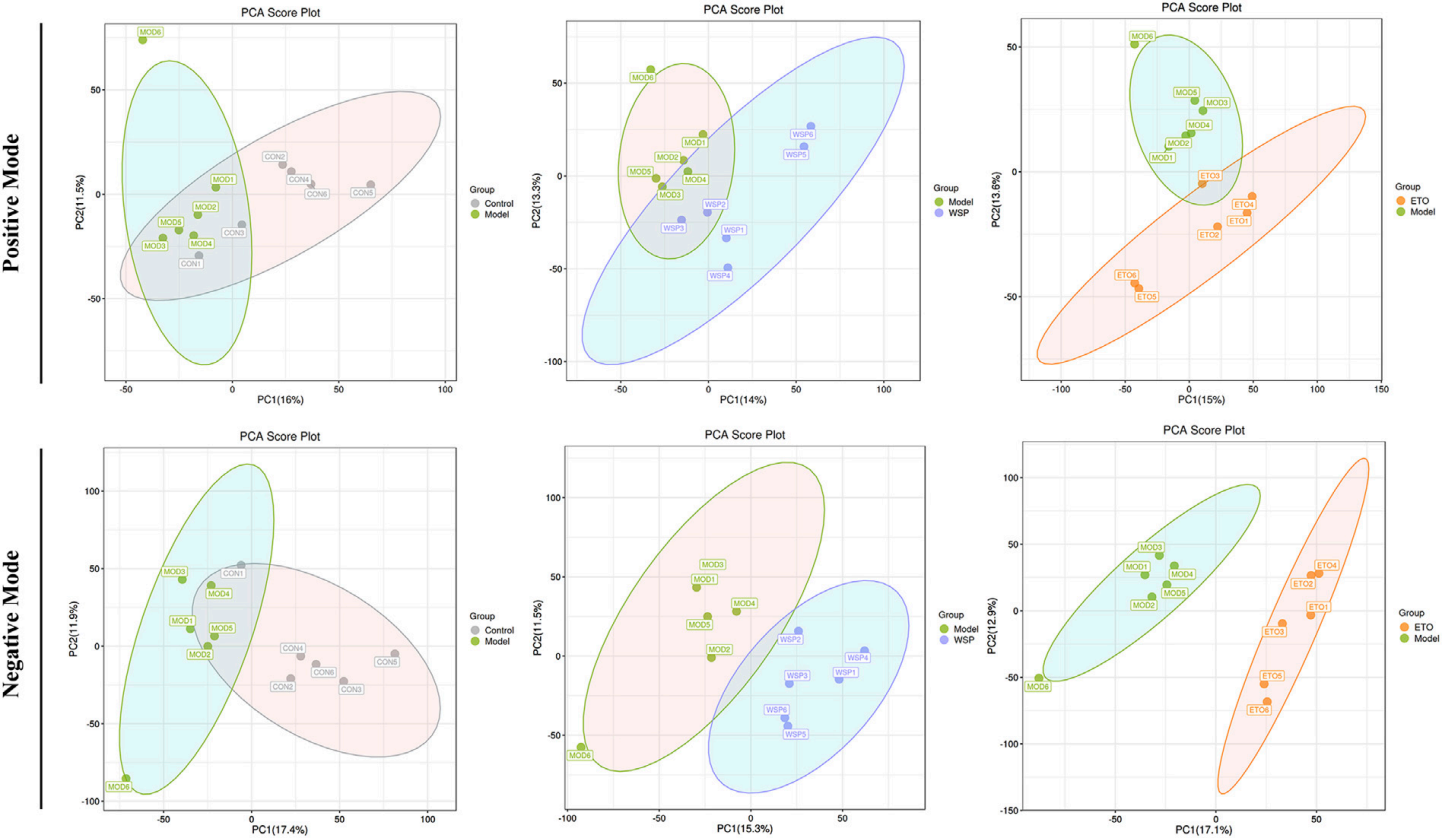
The statistical results of PCA analysis.

Metabolomics data are characterized by high latitude and multivariate, and in order to investigate the potential multidimensional data, we need to further process the obtained information, namely, principal component analysis (PCA), partial least squares discriminant analysis (PLS-DA), and orthogonal-partial least squares discriminant analysis (OPLS-DA) (Thevenot et al., 2015). PCA visually reflects the overall distribution characteristics of all samples and trends. As shown in Figure 3, each group had a good separation status and less overlap compared with the MOD group, especially in the negative mode. The R^2^X parameter of PCA was the main interpretability parameter of the model, and it was better when R^2^X > 0.5, as shown in Supplementary File S2, and R^2^X > 0.5 for each group in both positive and negative modes, suggesting that the model was accurate. The analysis results and parameters of PLS-DA and OPLS-DA are shown in Supplementary File S2 and Supplementary File S3.

#### 3.4.3 Identification and analysis of metabolic biomarkers

Compound structures could be inferred by primary MS, and further secondary MS is required to obtain precise information to improve the accuracy of the results. We performed metabolic biomarkers confirmation in HMDB, massbank, LipidMaps, mzcloud, and other databases based on MS/MS fragmentation patterns. Metabolic biomarkers were screened using VIP >1 as a condition and then were obtained using a *t*-test, *p* < 0.05 (Kieffer et al., 2016).

After MS/MS screening, a total of 30 metabolic biomarkers were identified after modeling by MSU compared with the CON group, including dodecanoic acid, taurohyocholate, mesaconate, 2-hydroxycinnamic acid, trans-ferulic acid, nicotinic acid, etc. A total of 15 biomarkers were upregulated and 15 were downregulated in the MSU-induced gouty rats (Shown in Table 5).

**TABLE 5:** Metabolic biomarkers between the MOD group and CON group.

Metabolic biomarkers	m/z	Retention time s)	Formula	-log10 *P*)	VIP	Trend in MOD
Dodecanoic acid	200.97	71	C_12_H_24_O_2_	3.45	2.39	↑
Taurohyocholate	516.30	527.1	C_26_H_45_NO_7_S	2.69	2.20	↓
Mesaconate	129.02	81	C_5_H_6_O_4_	3.10	2.18	↑
2-Hydroxycinnamic acid	165.05	209.1	C_9_H_8_O_3_	2.22	2.15	↑
trans-Ferulic acid	195.14	625.1	C_10_H_10_O_4_	2.36	2.10	↑
Nicotinic acid	124.04	104.5	C_6_H_5_NO_2_	2.44	2.08	↑
L-Methionine S-oxide	166.05	149	C_5_H_11_NO_3_S	2.36	2.06	↑
4-Guanidinobutanal	130.09	813.1	C_5_H_11_N_3_O	2.26	2.03	↑
9,10-Epoxyoctadecenoic acid	296.23	778.3	C_18_H_32_O_3_	2.12	2.02	↑
D-Xylose	149.01	821.9	C_5_H_10_O_5_	2.63	2.02	↓
Aflatoxin B_1_	312.36	770.7	C_17_H_12_O_6_	1.88	2.02	↑
26-Hydroxyecdysone	480.28	527	C_27_H_44_O_7_	2.08	2.01	↓
Mannitol	182.98	33	C_6_H_14_O_6_	2.09	1.99	↑
3-Dehydroecdysone	462.27	589.9	C_27_H_42_O_6_	1.94	1.96	↓
3-Methyloxindole	148.08	498.9	C_9_H_9_NO	1.94	1.94	↓
D-Fructose	179.06	927.6	C_6_H_12_O_6_	2.37	1.93	↓
Ursodeoxycholic acid	373.27	838.6	C_24_H_40_O_4_	1.96	1.86	↑
25-Hydroxycholesterol	401.09	471.8	C_27_H_46_O_2_	2.00	1.85	↑
4-Chlorobenzoate	138.99	980.2	C_7_H_5_ClO_2_	1.55	1.84	↓
L-Olivosyl-oleandolide	516.30	761.3	C_26_H_44_O_10_	1.71	1.83	↓
Caryophyllene alpha-oxide	203.18	816.1	C_15_H_24_O	1.68	1.80	↓
Catechol	111.02	754.2	C_6_H_6_O_2_	1.71	1.79	↓
3-Epiecdysone	464.28	669	C_27_H_44_O_6_	1.53	1.76	↓
Triacetate lactone	127.04	34.6	C_6_H_6_O_3_	1.54	1.75	↓
Eucalyptol	153.96	69.7	C_10_H_18_O	1.56	1.74	↑
Bilirubin	585.27	794.2	C_33_H_36_N_4_O_6_	1.45	1.70	↑
Nonadecanoic acid	297.24	802.5	C_19_H_38_O_2_	1.49	1.62	↓
FAPy-adenine	154.07	246.4	C_5_H_7_N_5_O	1.36	1.61	↓
Glycocholic acid	464.30	534.1	C_26_H_43_NO_6_	1.36	1.48	↑
β-D-Fructose 6-phosphate	259.02	82.2	C_6_H_13_O_9_P	1.32	1.45	↓

Furthermore, a total of 42 metabolic biomarkers were screened in GA rats treated with WSP, including 2-hydroxycinnamic acid, hydroxykynurenine, nicotinic acid, 2-methoxyestradiol, and N6-Acetyl-L-lysine. As displayed in Table 6, a total of 11 metabolites in the serum were upregulated and 31 were downregulated by the administration of WSP.

**TABLE 6:** Metabolic biomarkers between the WSP group and MOD group.

Metabolic biomarkers	m/z	Retention time s)	Formula	-log10 *P*)	VIP	Trend in WSP
2-Hydroxycinnamic acid	165.05	209.1	C_9_H_8_O_3_	2.82	2.27	↓
Hydroxykynurenine	225.03	613.6	C_10_H_12_N_2_O_4_	2.03	2.20	↓
Nicotinic acid	124.04	104.5	C_6_H_5_NO_2_	2.44	2.15	↓
2-Methoxyestradiol	283.17	825.9	C_19_H_26_O_3_	2.58	2.13	↓
N6-Acetyl-L-lysine	189.12	146.6	C_8_H_16_N_2_O_3_	2.17	2.10	↓
Caryophyllene alpha-oxide	203.18	816.1	C_15_H_24_O	1.73	2.06	↑
9,10-Epoxyoctadecenoic acid	296.23	778.3	C_18_H_32_O_3_	1.96	2.03	↓
L-2-Hydroxyglutaric acid	146.96	667.7	C_5_H_8_O_5_	2.35	2.01	↓
12-Hydroxydodecanoic acid	215.01	928.9	C_12_H_24_O_3_	2.2	2.00	↓
Ribose 1,5-bisphosphate	309.17	736.9	C_5_H_12_O_11_P_2_	2.09	1.97	↓
Palmitoleic acid	237.22	890	C_16_H_30_O_2_	1.98	1.96	↓
Ursodeoxycholic acid	373.27	838.6	C_24_H_40_O_4_	1.77	1.95	↓
L-Cysteine	120.98	52.6	C_3_H_7_NO_2_S	1.88	1.95	↓
D-Erythritol 4-phosphate	202.03	468.7	C_4_H_11_O_7_P	2.1	1.94	↑
L-2,4-diaminobutyric acid	118.07	281.9	C_4_H_10_N_2_O_2_	1.9	1.93	↓
(S)-2-Methylmalate	148.03	317.5	C_5_H_8_O_5_	1.8	1.93	↓
Benzaldehyde	107.05	283	C_7_H_6_O	1.82	1.92	↓
Bilirubin	585.27	794.2	C_33_H_36_N_4_O_6_	1.77	1.91	↓
Phenylacetaldehyde	120.06	410.9	C_8_H_8_O	1.74	1.89	↓
4-Quinolinecarboxylic acid	171.91	35.4	C_10_H_7_NO_2_	1.46	1.81	↓
α-dimorphecolic acid	279.23	794.2	C_18_H_32_O_3_	1.55	1.81	↓
9,10-DHOME	313.24	703.5	C_18_H_3_4O_4_	1.7	1.79	↓
D-Phenylalanine	165.02	921.4	C_9_H_11_NO_2_	1.75	1.79	↑
Neocnidilide	177.13	530.2	C_12_H_18_O_2_	1.36	1.79	↓
γ-Glutamylcysteine	248.96	93.7	C_8_H_14_N_2_O_5_S	1.77	1.78	↓
Sodium deoxycholate	414.32	848.2	C_24_H_39_O_4_Na	1.49	1.77	↑
L-Fucose	164.07	928.7	C_6_H_12_O_5_	1.63	1.76	↑
Pterin	163.04	931	C_6_H_5_N_5_O	1.5	1.75	↑
5-Methyl-2′-deoxycytidine	240.10	254.5	C_10_H_15_N_3_O_4_	1.57	1.75	↓
5-Hydroxyindoleacetic acid	173.98	117	C_10_H_9_NO_3_	1.49	1.71	↑
β-Tyrosine	164.07	953.9	C_9_H_11_NO_3_	1.4	1.71	↓
6-Hydroxyhexan-6-olide	130.07	315.9	C_6_H_10_O_3_	1.39	1.70	↑
Mannose 6-phosphate	260.02	146.3	C_6_H_13_O_9_P	1.57	1.70	↓
6-Keto-prostaglandin F1a	371.26	660.4	C_20_H_34_O_6_	1.43	1.70	↓
4-Guanidinobutanal	130.09	813.1	C_5_H_11_N_3_O	1.39	1.69	↓
L-Valine	118.09	126.1	C_5_H_11_NO_2_	1.39	1.68	↑
(2Z,4S,5R)-2-Amino-4,5,6-trihydroxyhex-2-enoate	178.09	295.7	C_6_H_11_NO_5_	1.43	1.67	↓
6-Hydroxynicotinic acid	138.02	536.2	C_6_H_5_NO_3_	1.53	1.66	↓
γ-Linolenic acid	277.22	864.5	C_18_H_30_O_2_	1.49	1.63	↓
Dodecanoic acid	200.97	71	C_12_H_24_O_2_	1.36	1.63	↓
15-Deoxy-d-12,14-PGJ_2_	315.20	757.2	C_20_H_28_O_3_	1.31	1.59	↑
D-Xylose	149.01	821.9	C_5_H_10_O_5_	1.33	1.55	↑

**FIGURE 4:**
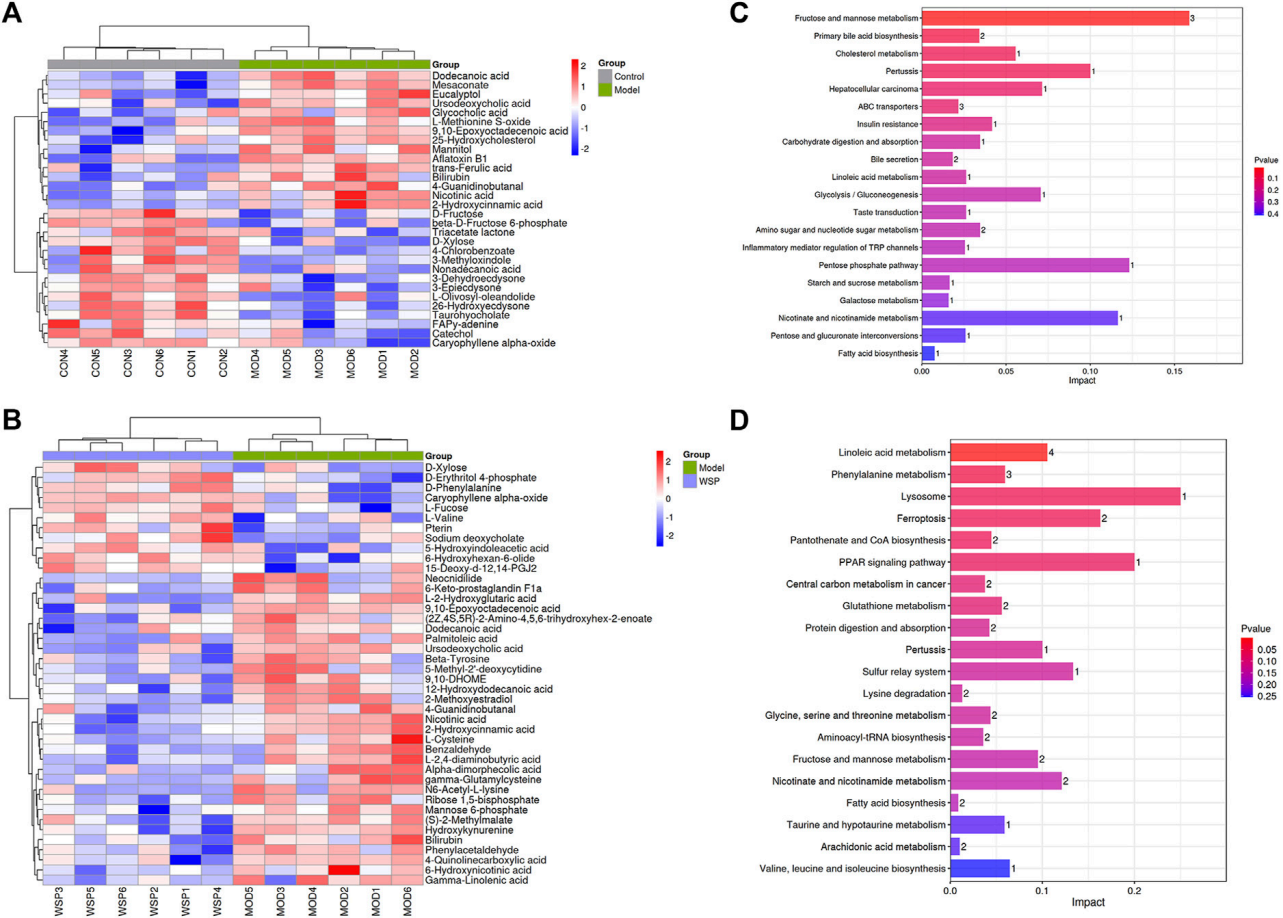
Metabolic biomarkers clustering heatmap **(A)** MOD vs CON. **(B)** WSP vs MOD. The results of metabolic pathways enrichment analysis **(C)** MOD vs CON. **(D)** WSP vs MOD.

Subsequently, the above results were collected for the agglomerative hierarchical clustering analysis, and the dramatically altered biomarkers were plotted in the form of heat maps. The metabolic biomarkers which might share the same metabolic function or metabolic pathways were clustered to visualize the changes and classification. The results are shown in Figures 4A, B.

#### 3.4.4 Metabolic pathway enrichment analysis

**FIGURE 5:**
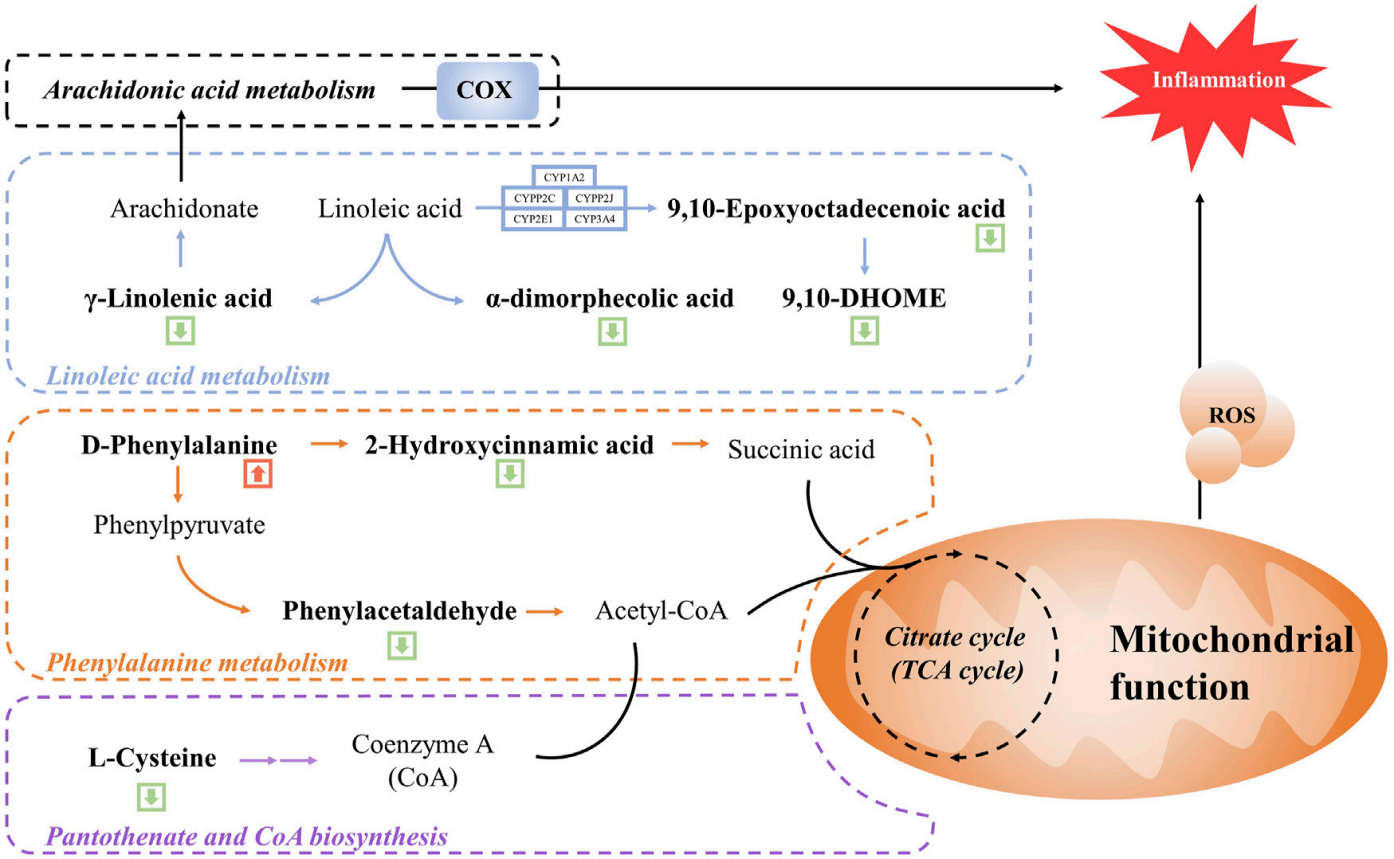
Schematic diagram of the metabolic networks regulated by WSP. Green arrow indicates decreased metabolites in the serum and red arrow indicates metabolites in the serum.

We further enriched and investigated the information by using the MetPA database to elucidate the metabolic pathways in the model rats that might be affected by the administration of WSP through KEGG metabolic pathway enrichment analysis. As shown in Figures 4C, D, 27 metabolic pathways were enriched in the MOD group compared with the CON group, mainly involving fructose and mannose metabolism, primary bile acid biosynthesis, cholesterol metabolism, and other pathways. As shown in Figures 4C, D a total of 39 metabolic pathways were enriched in the GA rats with WSP administration mainly in linoleic acid metabolism, phenylalanine metabolism, lysosomal, ferroptosis, pantothenate and CoA biosynthesis, PPAR signaling pathway, etc. [the top six pathways ranked by -log10P)]. Based on the results of metabolic biomarkers screening and the enrichment of metabolic pathways, we mapped a metabolic network in which WSP might affect GA (Figure 5).

### 3.5 Comprehensive analysis

**FIGURE 6:**
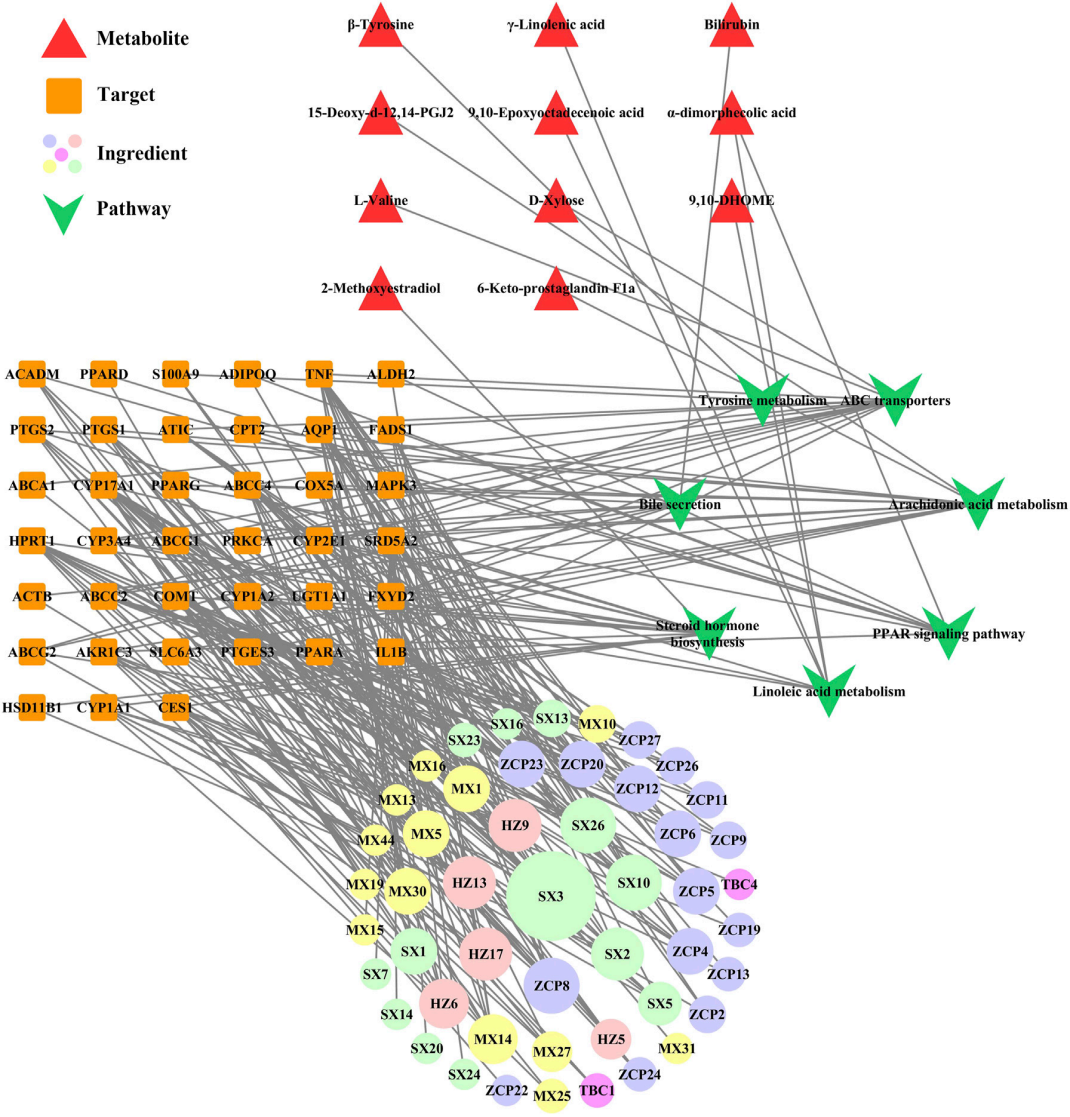
Metabolites-pathways-targets-ingredient network. Markers of the ingredients checklist are shown in Supplementary File S5, and the bigger nodes represented greater degrees. HZ: *Terminalia chebula* Retz.; TBC: *Aconitum pendulum* Busch; MX: *Aucklandia lappa* Decne.; ZCP: *Acorus calamus* L.; SX: Artificial musk.

In our previous study, we obtained information on the effective pathway of WSP in treating gout via KEGG enrichment (details shown in Supplementary File S4). To further elaborate the regulatory network of WSP, the comprehensive analysis combined metabolomics and network pharmacology was applied to construct a metabolites-pathways-targets-ingredient network (Shown in Figure 6), which involved 11 metabolites, 7 pathways, 39 targets, and 49 ingredients of WSP. The results showed that WSP mainly intervened in arachidonic acid metabolism, ABC transporters, and PPAR signaling pathway.

## 4 Discussion

WSP has been used in China, especially among Tibetan people, for the treatment of inflammation and inflammation-related diseases, such as tonsillitis, pharyngitis, and rheumatoid arthritis. Our preliminary study found that WSP might exert an anti-gout effect through multiple targets and pathways (Lang et al., 2022). However, the specific mechanism is unclear. In this study, we explored the effect of WSP on MSU-induced gouty arthritis in rats and used LC-MS untargeted metabolomics approach to reveal its possible mechanism for the first time.

Early anti-inflammatory treatment to control pain and the progression of arthritis is the current consensus treatment in acute gout flares (FitzGerald et al., 2020). An acute gouty inflammatory response is triggered by the precipitation of MSU crystals due to elevated uric acid levels in the blood, which is mainly a self-limiting inflammatory response mediated by autoimmune cells. Clinically, gout is characterized by joint swelling and heat pain, with a short time from flare to peak (usually shorter than 12 h), and severe pain can even affect activities and walking (Taylor et al., 2015). Thus, early anti-inflammatory treatment can result in greater benefits for gout patients. In view of pathogenesis, the cellular mechanism of gout is complex and it involves Toll-like receptor signaling initiation, NF-κB signaling activation, oxidative stress, and NLRP3 signaling pathway (Liu-Bryan et al., 2005; Bauernfeind et al., 2009; Dominic et al., 2022). Stimulation of pathogen-associated molecular patterns or damage associated molecular patterns triggers the migration of monocytes-macrophages and exerts the body’s autoimmune defense. MSU crystals can stimulate innate immune pathways and are closely associated with the initiation of NLRP3 inflammasome (Martinon et al., 2006). Activation of NLRP3 inflammasome is a necessary signal of gouty inflammation. Upon activation of upstream signaling, cytoplasmic pattern recognition receptors (PRRs) such as NLRP3 perform a series of reactions including recruitment, aggregation, and assembly. Pro-caspase-1 protein is recruited by ASC, forming NLRP3 inflammasome (Schroder et al., 2010). Then, it will mediate the maturation and release process of IL-1β, causing a severe inflammatory response. Hence, we used an *in vivo* model to validate the anti-gout effect of WSP. In this model, the foot swelling, pathological, and immunohistochemical performance after MSU injection were assayed to observe the effect of WSP on gout. Furthermore, metabolomics was used to detect metabolic biomarkers expression in model animals and after administration of WSP, to provide further evidence of metabolic regulation of the anti-gout effect.

In MSU-stimulated acute arthritis of the ankle joint, the foot swelling of the MOD group increased significantly at all time points from 2 h post-modeling compared to the CON group, and the foot swelling basically peaked at 8 h and lasted for 24 h. It is suggested that MSU triggered a severe inflammatory response from 2 h and was in the ascending phase of inflammation for 24 h. This result was consistent with previous reports (Goo et al., 2021; Zhou et al., 2022).

Acute gouty arthritis caused severe joint pain and swelling and might lead to changes in levels of serum metabolic biomarkers. However, the study of specific mechanisms of TCM and its preparations has become fairly complicated due to its complex components and multi-target therapeutic characteristics. Recently, network pharmacology has been used in the discovery of active compounds and the interpretation of the overall mechanisms in the TCM or TCM preparations, by collecting and analyzing data from bioinformation databases associated with herbs, disease targets, and pathways. Nevertheless, network pharmacology has several limitations if it is used alone**,** such as prediction-based false-positive results, varying relative abundance of compounds in TCM, and debatable ADME-based screening (Jiashuo et al., 2022). Thus, an integrated strategy combining network pharmacology with other approaches is increasingly being used. For instance, network pharmacology methodology is combined with multi-omics studies such as proteomics (Cheng et al., 2022; Sun et al., 2022), metabolomics (Pan et al., 2020; Zhou et al., 2020b; Li et al., 2021), transcriptomics (Xiao et al., 2021; Zhou et al., 2022a), and lipidomics (Chen et al., 2022). Furthermore, gut microbiomics (Goo et al., 2021; Yao et al., 2022) and meta-analysis (Yi et al., 2020) were adopted for overall analysis combined with network pharmacology methodology. Taking into account the multi-faceted aspects and complexities of TCM or TCM preparations at hand, the integration strategy combining multi-omics with network pharmacology is beneficial for the accuracy of prediction results. In this study, LC-MS-based untargeted metabolomics assays identified 30 potential biomarkers in the pathology of acute gouty arthritis, 15 of them were upregulated and 15 were downregulated. A total of 42 serum metabolic biomarkers were identified in model rats after treatment with WSP, 11 of them were upregulated and 31 were downregulated. The above results were enriched in metabolic pathways, mainly involving linoleic acid metabolism, phenylalanine metabolism, lysosomal, ferroptosis, pantothenate and CoA biosynthesis, PPAR signaling pathway, etc.

Linoleic acid is an essential nutrient for the human body, but disorders of linoleic acid metabolism can lead to disease. Linoleic acid is a synthetic precursor of arachidonic acid, which is metabolized to produce γ-linolenic acid. γ-linolenic acid can participate in the arachidonic acid (AA) metabolic pathway to generate AA, which exhibits pro-inflammatory and pro-thrombotic potential. Eicosanoids are locally acting bioactive signaling lipids, which are considered pro-inflammatory mediators for inflammation, immunity, and allergies, and eicosanoids are derived from arachidonic, γ-linolenic acid, and polyunsaturated fatty acids (Dennis and Norris, 2015). Cyclooxygenase (COX) is one of the metabolic mediators of the eicosanoids synthesis pathways, which controls a wide range of inflammatory processes (Smith et al., 2000). For instance, AA can be converted into prostaglandins enzyme-catalyzed by COX. During gouty inflammation, the first-line treatment strategy is COX inhibitors, such as unselective COX inhibitors (ibuprofen and aspirin) and COX-2-targeted agents (Etoricoxib) (Wu et al., 2022; Burkett et al., 2023). The above inflammatory metabolites and mediators are worthy of attention in the management of gout. Our metabolomic results showed that γ-linolenic acid, α-dimorphecolic acid, and 9,10-Epoxyoctadecenoic acid and 9,10-DHOME, as well as all the downstream of the linoleic acid metabolic pathway, were downregulated by WSP treatment, suggesting that WSP might inhibit the linoleic acid metabolic pathway and further affect AA metabolism to exert the anti-inflammatory effect. Interestingly, this conjecture was again confirmed in the integrative analysis combined with network pharmacology, which revealed a nonnegligible role of AA metabolism in WSP modulatory networks. Thus, key indicators of the linoleic acid metabolic pathway, such as lipoxygenase and cytochrome P4501A2, could be subsequently validated by qRT-PCR, Western blot, and ELISA technology (Wang et al., 2022). In consideration of the inhibition of WSP on the MAPK/NF-κB signaling pathway (Lang et al., 2022), we hypothesized that WSP inhibited the combination of NF-κB and target DNA, thereby suppressing COX expression and further reducing the production of related metabolites catalyzed by COX1/2, such as PGs. In the present study, we elucidated the role of WSP in the NF-κB signaling pathway from a metabolomics perspective for the first time.

Phenylalanine metabolism is one of the important metabolic pathways of amino acids, which has also been reported to be closely associated with hyperuricemia and gout (Jiang et al., 2017; Zhou et al., 2022). Phenylpyruvate and 2-hydroxycinnamic acid are products of the phenylalanine metabolic pathway, and phenylpyruvate can further generate phenylacetaldehyde, which generates phenylacetal-CoA, which is involved in the biosynthesis of pantothenic acid and CoA via Acetyl-CoA (Wu et al., 2018). In our study, D-phenylalanine expression was upregulated after administration of WSP, while the expression of the downstream phenylacetaldehyde and 2-hydroxycinnamic acid was downregulated. We speculated that WSP might be able to correct the abnormal phenylalanine metabolic pathway which led to the reduction of downstream phenylacetaldehyde and 2-hydroxycinnamic acid. An abnormal serum level of phenylalanine in patients with gout has been reported in several previous studies (Zhang et al., 2018; Huang et al., 2020; Shen et al., 2021), suggesting that it might be one of the metabolic biomarkers of gout, which was consistent with our findings. Furthermore, the levels of several amino acids such as valine, phenylalanine, tyrosine, and cysteine were significantly altered in this study. Notably, a previous study on serum metabolic biomarkers in gout patients also showed that the disorder of the amino acid metabolic pathways could be involved in the pathogenic mechanism of gout (Shen et al., 2021). Moreover, the phenylalanine metabolic process would continue to affect the downstream biosynthetic pathways of pantothenic acid and CoA, which was consistent with the KEGG enrichment results. Pantothenic acid is the precursor of CoA, which is the cofactor of various metabolic reactions and its synthesis is regulated by acetoacetyl-CoA synthetase (AACS) (Shan et al., 2021). CoA plays an essential role in energy metabolism and participates in the metabolism of glucose, protein, and lipids via the TCA cycle (Hrubsa et al., 2022; Wang et al., 2023). The TCA cycle is the center of energy metabolism, which is closely related to mitochondrial function. As we know, MSU could trigger mitochondrial dysfunction and cause an increase in mitochondrial ROS, thus inducing caspase-1-independent IL-1β secretion (Abderrazak et al., 2015). In this condition, an increment of mitochondrial fatty acid oxidation could induce the increased generation of acetyl CoA, followed by the augmented NADH and FADH2 levels in the TCA cycle, which amplified more ROS formation, reinforcing a vicious cycle for the activation of inflammasome (de Mello et al., 2018; Cojocaru et al., 2023). In general, pantothenic acid and CoA play an essential role in cellular metabolism. Therefore, we hypothesized that WSP indirectly affected mitochondrial function and the TCA cycle by regulating upstream phenylalanine metabolism and biosynthetic pathways of pantothenic acid and CoA, thus modulating the alteration of metabolic pathways. Indeed, some limitations of this study should be noted. Firstly, the effects of WSP in rodent models of gout did not reflect the changes it may cause in patients with gout. Therefore, it is essential to conduct clinical trials to reveal the alteration of serum metabolite composition after WSP intervention in gouty patients. Besides, integrated with network pharmacology analysis, we predicted the active anti-gout ingredients in WSP that might affect metabolic pathways associated with gout flares. Additional *in vitro* experiments should be performed to further validate the anti-gout potential and specific mechanisms of the predicted active compounds, thus clarifying the material basis of WSP.

## 5 Conclusion

In the acute gouty arthritis model of rats, WSP treatment inhibited significant foot swelling, attenuated pathological lesions, and downregulated the related protein expression of NLRP3 inflammasome signaling in synovial tissue of the ankle. Further metabolomics combined with network pharmacology analysis suggested that the therapeutic effect of WSP involved 11 biomarkers and 7 metabolic pathways. We speculated that WSP might regulate linoleic acid metabolism, phenylalanine metabolism, and pantothenate and CoA biosynthesis, then further affect the arachidonic acid metabolic pathway and mitochondrial function, thus inhibiting gouty inflammation. The above results suggested that WSP could be a prospective candidate as a novel anti-gout agent for the secondary development of Chinese traditional patent medicine.

## Data Availability

The datasets presented in this study can be found in online repositories. The names of the repository/repositories and accession number(s) can be found below: MetaboLight database under MTBLS7783.
