# Supplementary material for: LC-MS-based metabolomics reveals the mechanism of anti-gouty arthritis effect of Wuwei Shexiang pill
Source: Front Pharmacol. 2023 Aug 11;14:1213602. doi: 10.3389/fphar.2023.1213602 (PMC10450745; doi:10.3389/fphar.2023.1213602)
Supplement: Supplementary file 3 [file Table1.docx]

Supplementary table 1 Parameters of multivariate statistical analysis results

| Group | Model | Positive mode | | | Negative mode | | |
| --- | --- | --- | --- | --- | --- | --- | --- |
|  |  | R^2^X  (cum) | R^2^Y  (cum) | Q^2^  (cum) | R^2^X  (cum) | R^2^Y  (cum) | Q^2^  (cum) |
| CON vs. MOD | PCA | 0.550 |  |  | 0.572 |  |  |
|  | PLS | 0.251 | 0.999 | 0.680 | 0.263 | 0.998 | 0.717 |
|  | OPLS | 0.251 | 0.999 | 0.457 | 0.263 | 0.998 | 0.586 |
| WSP vs. MOD | PCA | 0.560 |  |  | 0.569 |  |  |
|  | PLS | 0.229 | 0.999 | 0.608 | 0.254 | 0.999 | 0.719 |
|  | OPLS | 0.229 | 0.999 | 0.485 | 0.254 | 0.999 | 0.580 |
| ETO vs.MOD | PCA | 0.557 |  |  | 0.572 |  |  |
|  | PLS | 0.234 | 0.999 | 0.687 | 0.284 | 0.999 | 0.836 |
|  | OPLS | 0.234 | 0.999 | 0.530 | 0.284 | 0.999 | 0.696 |
