# Supplementary figures and images for: LC-MS-based metabolomics reveals the mechanism of anti-gouty arthritis effect of Wuwei Shexiang pill (part 1 of 3)
Source: Front Pharmacol. 2023 Aug 11;14:1213602. doi: 10.3389/fphar.2023.1213602 (PMC10450745; doi:10.3389/fphar.2023.1213602)

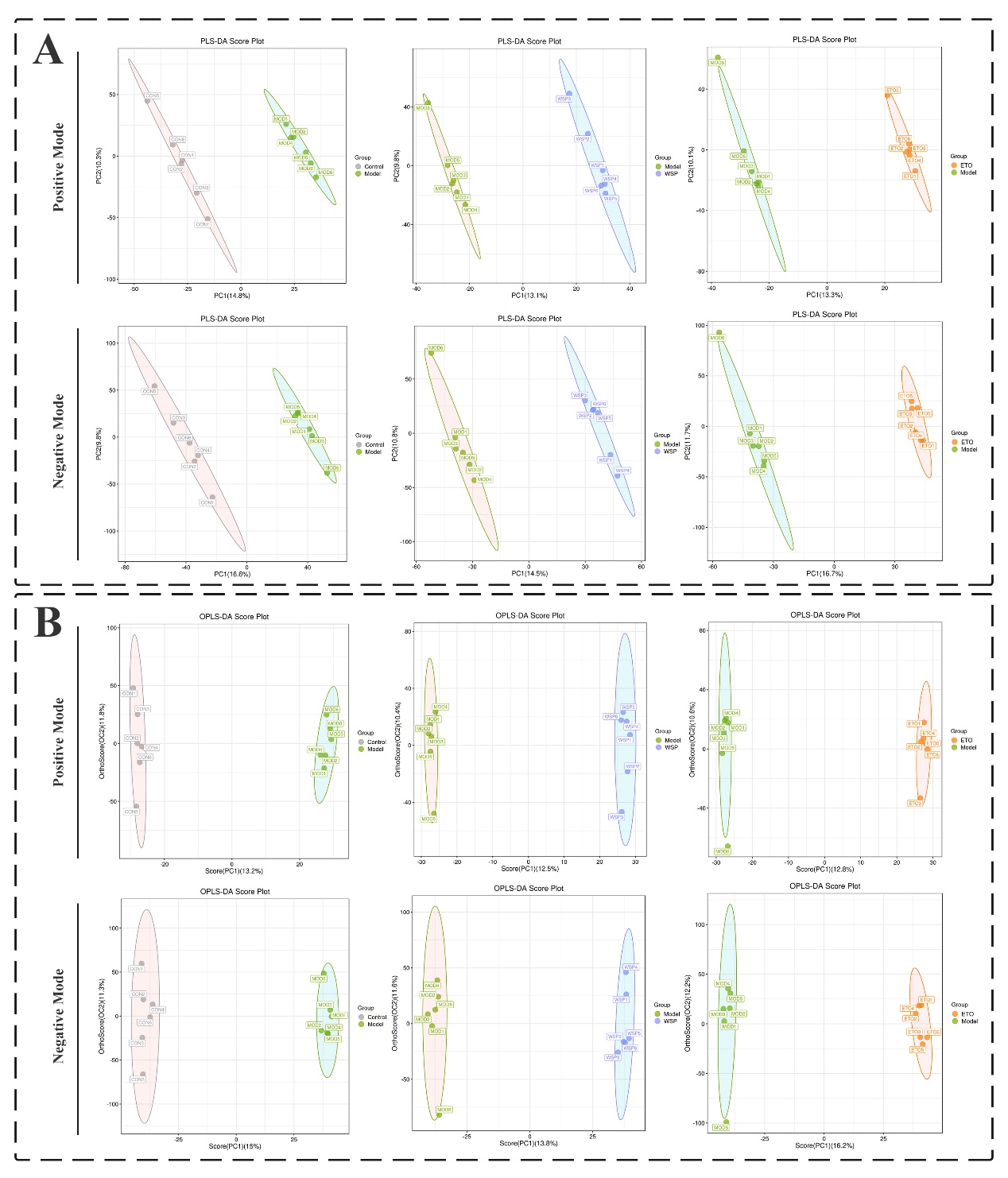


Supplementary figure 2 (A) PLS-DA. (B) OPLS-DA.

Supplement: Supplementary file 4 [file DataSheet3.docx]

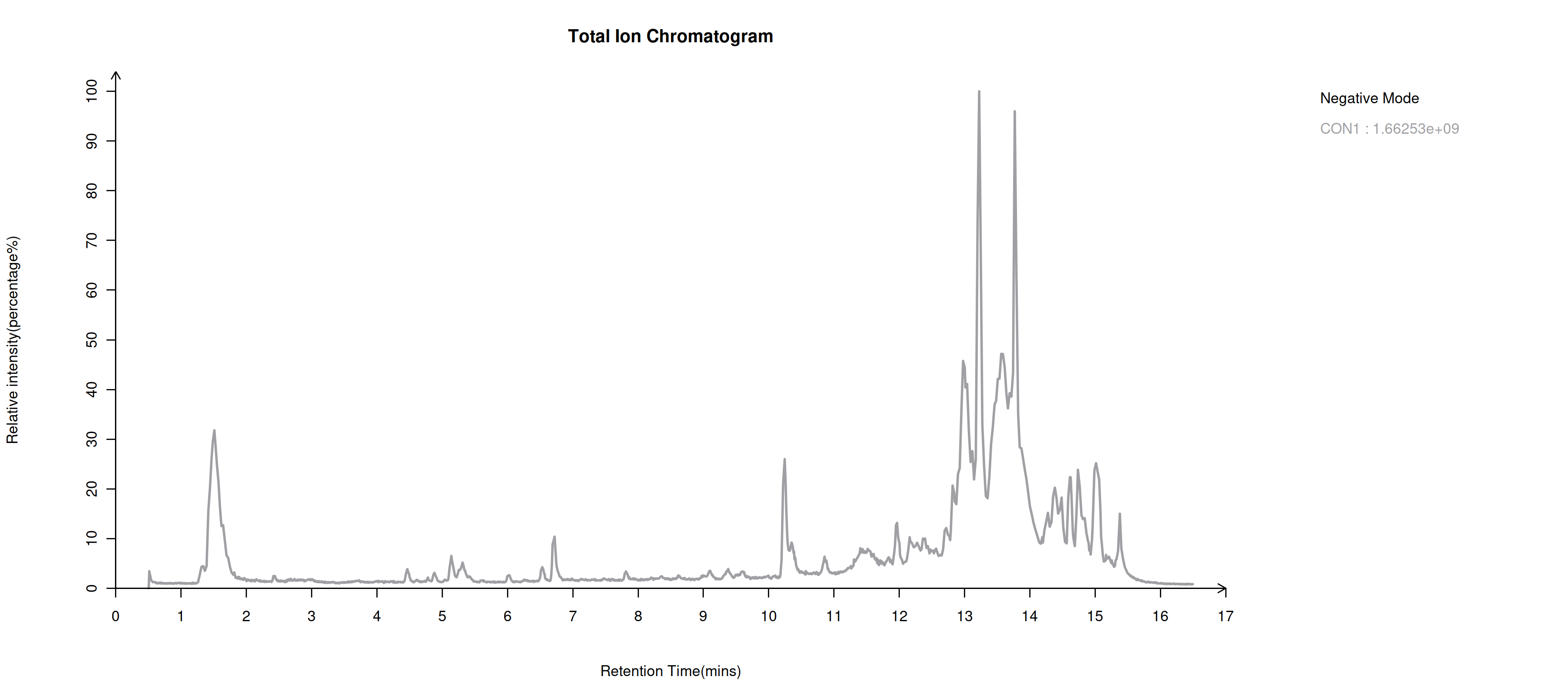

Supplement: Supplementary file 5 [file DataSheet1.ZIP › 1 TIC diagrams of all samples/Negative mode/CON1.png]

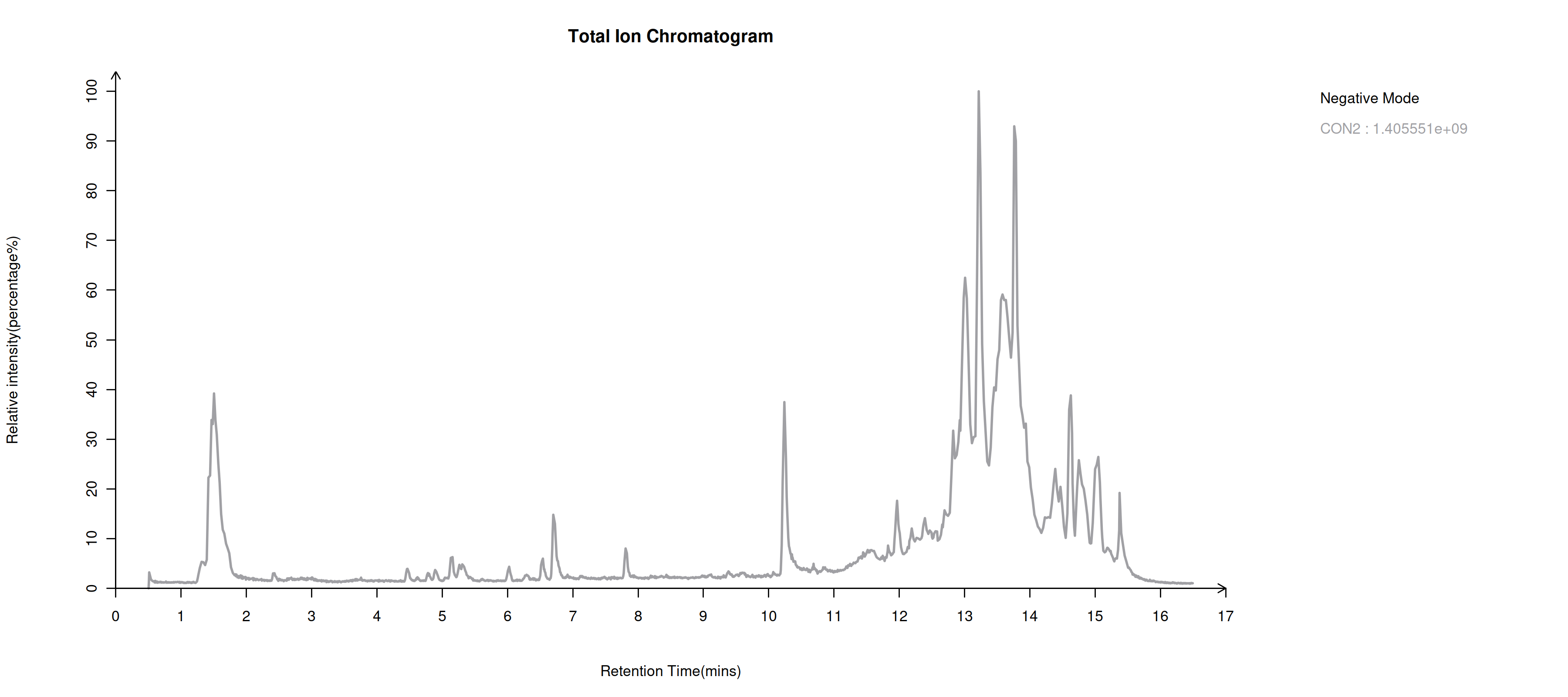

Supplement: Supplementary file 5 [file DataSheet1.ZIP › 1 TIC diagrams of all samples/Negative mode/CON2.png]

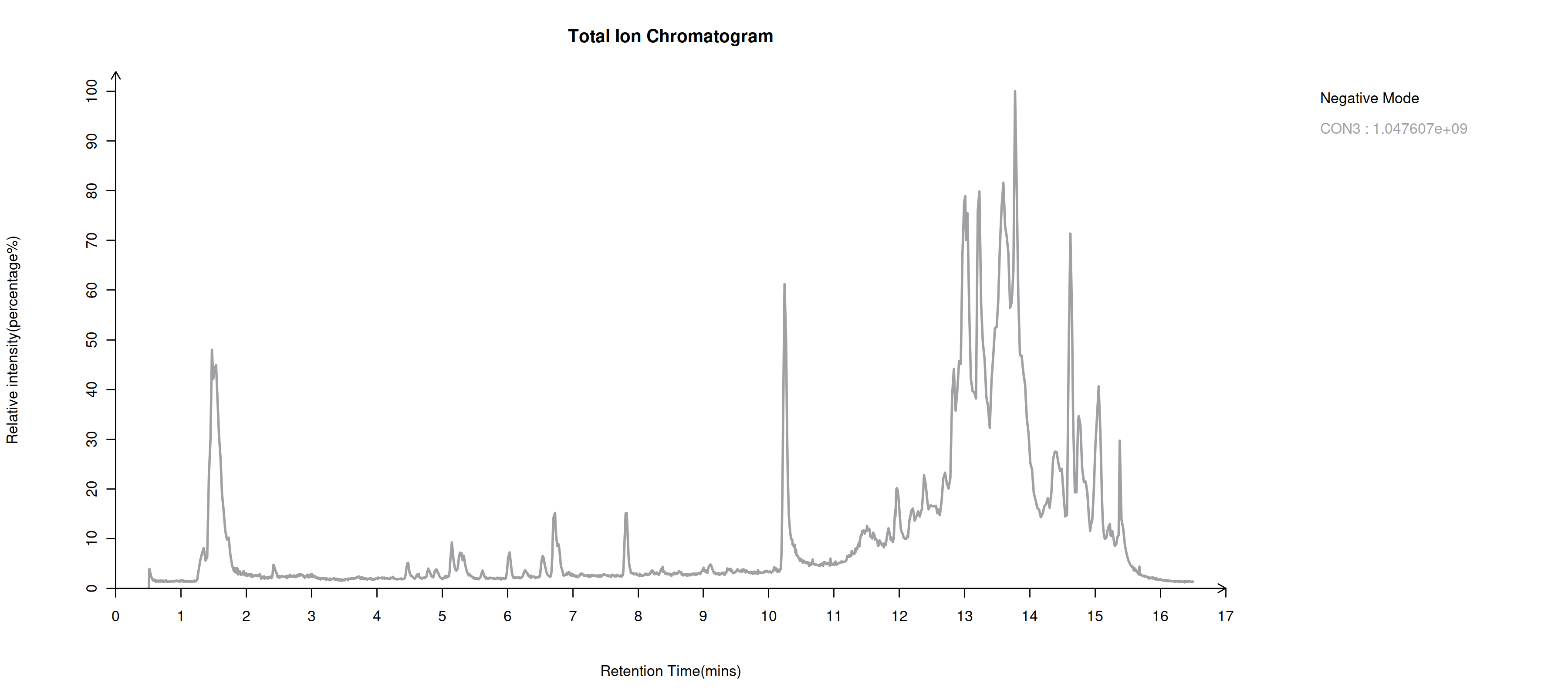

Supplement: Supplementary file 5 [file DataSheet1.ZIP › 1 TIC diagrams of all samples/Negative mode/CON3.png]

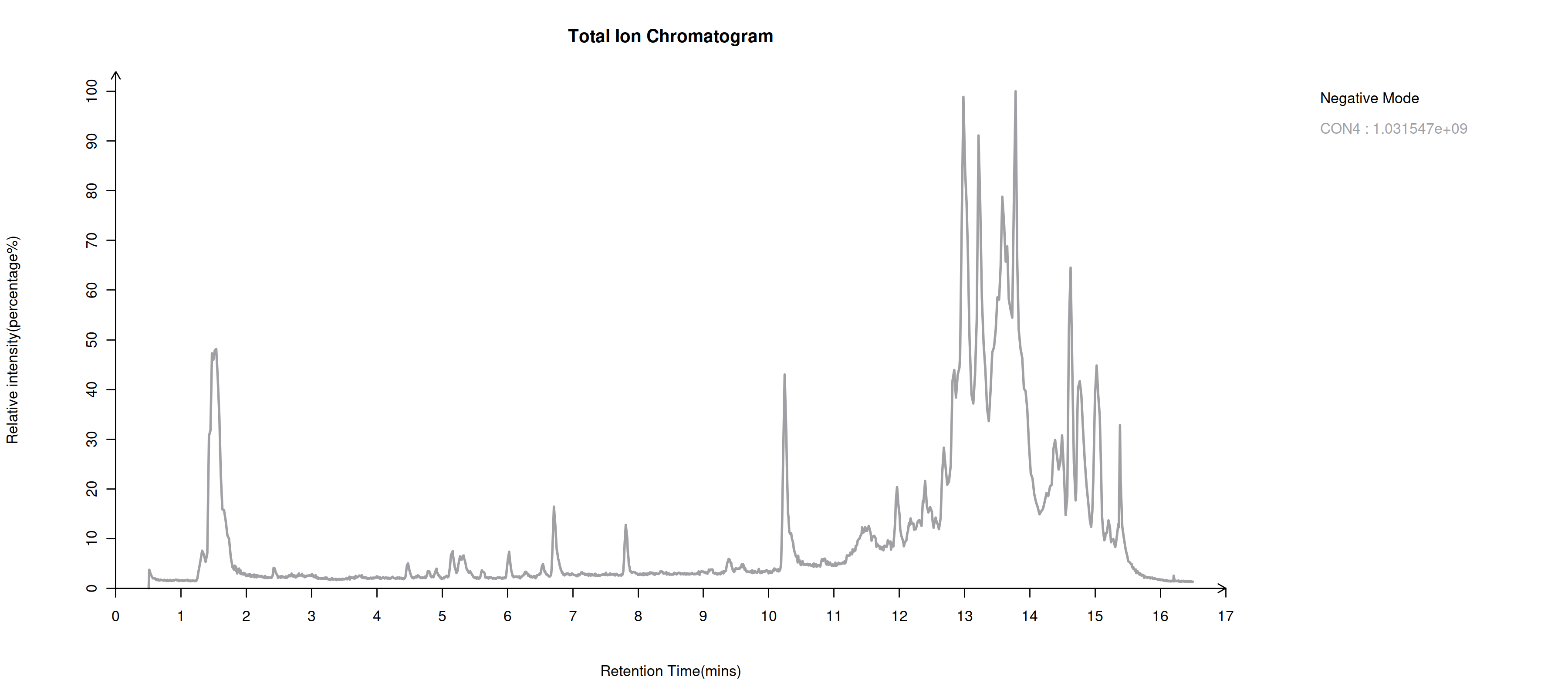

Supplement: Supplementary file 5 [file DataSheet1.ZIP › 1 TIC diagrams of all samples/Negative mode/CON4.png]

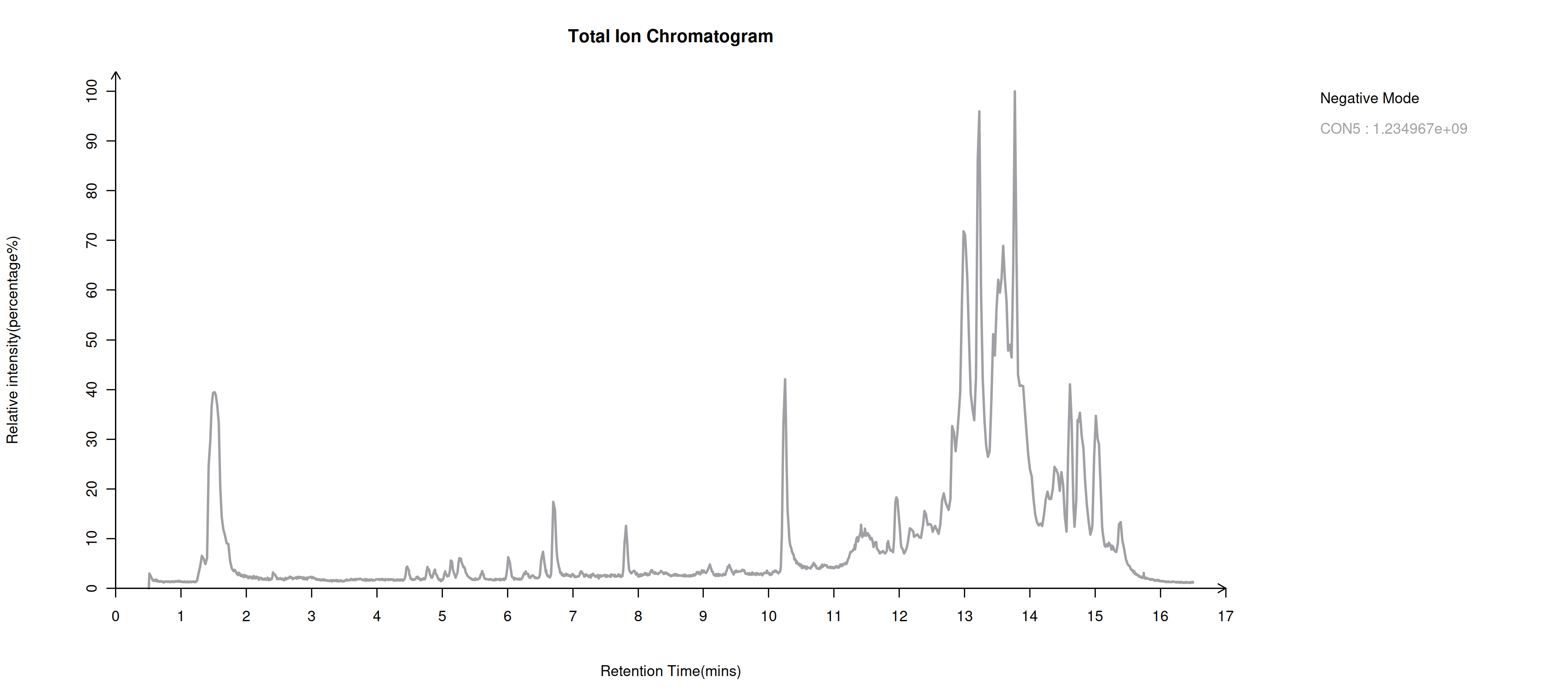

Supplement: Supplementary file 5 [file DataSheet1.ZIP › 1 TIC diagrams of all samples/Negative mode/CON5.png]

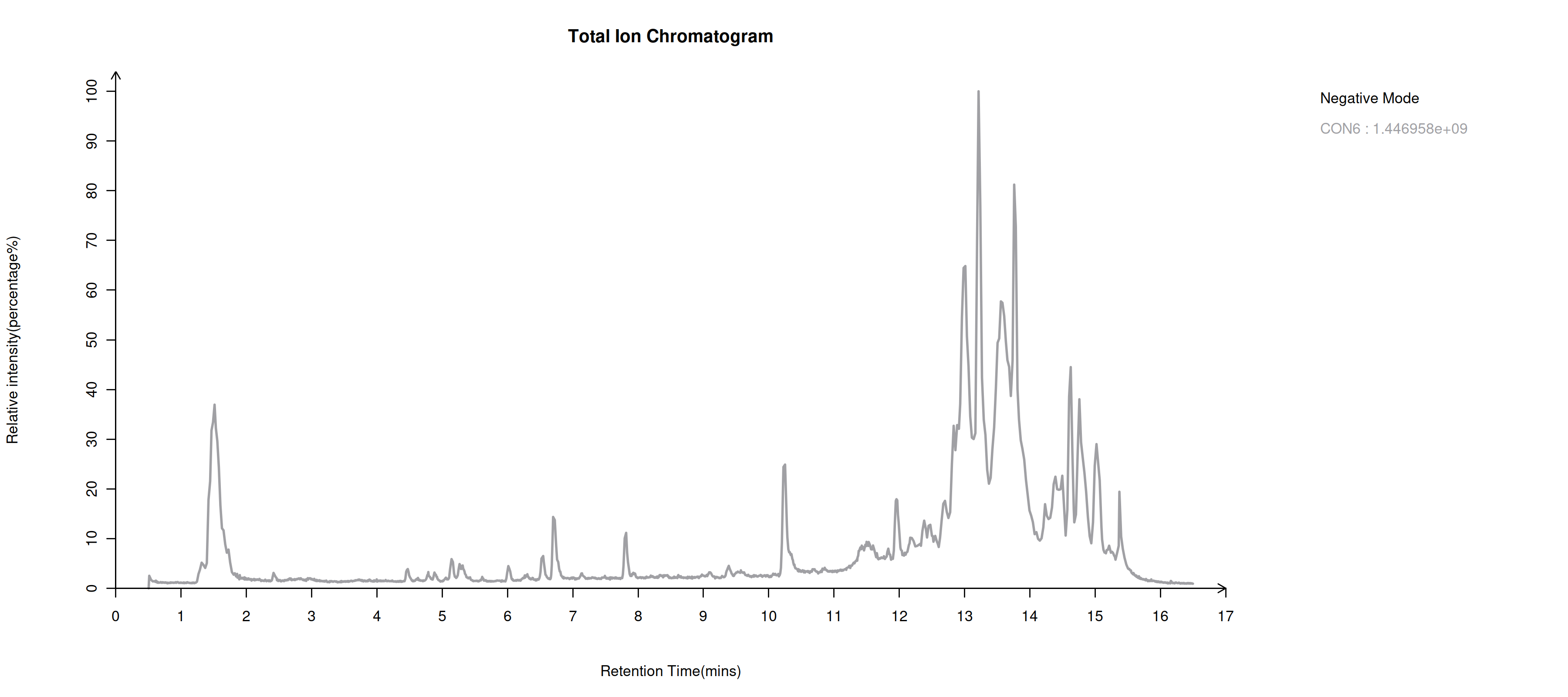

Supplement: Supplementary file 5 [file DataSheet1.ZIP › 1 TIC diagrams of all samples/Negative mode/CON6.png]

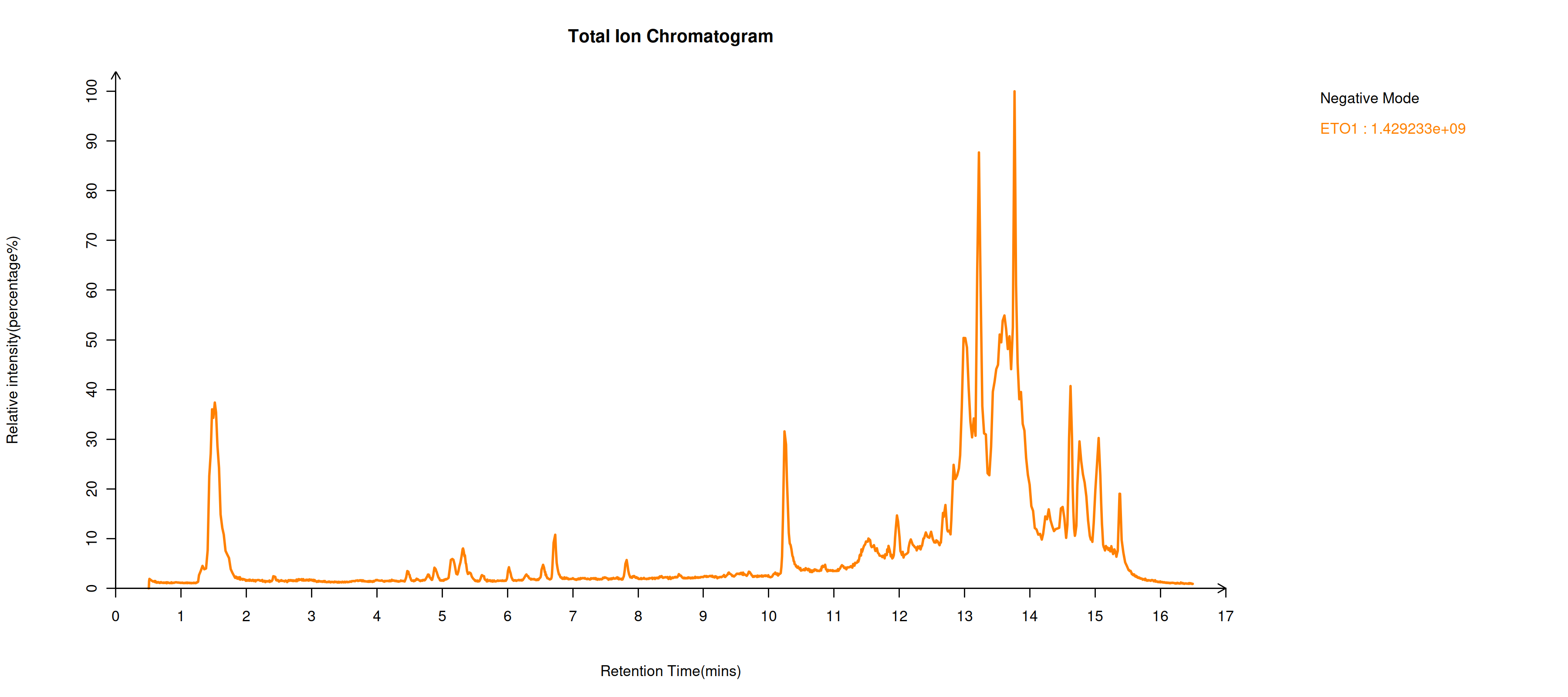

Supplement: Supplementary file 5 [file DataSheet1.ZIP › 1 TIC diagrams of all samples/Negative mode/ETO1.png]

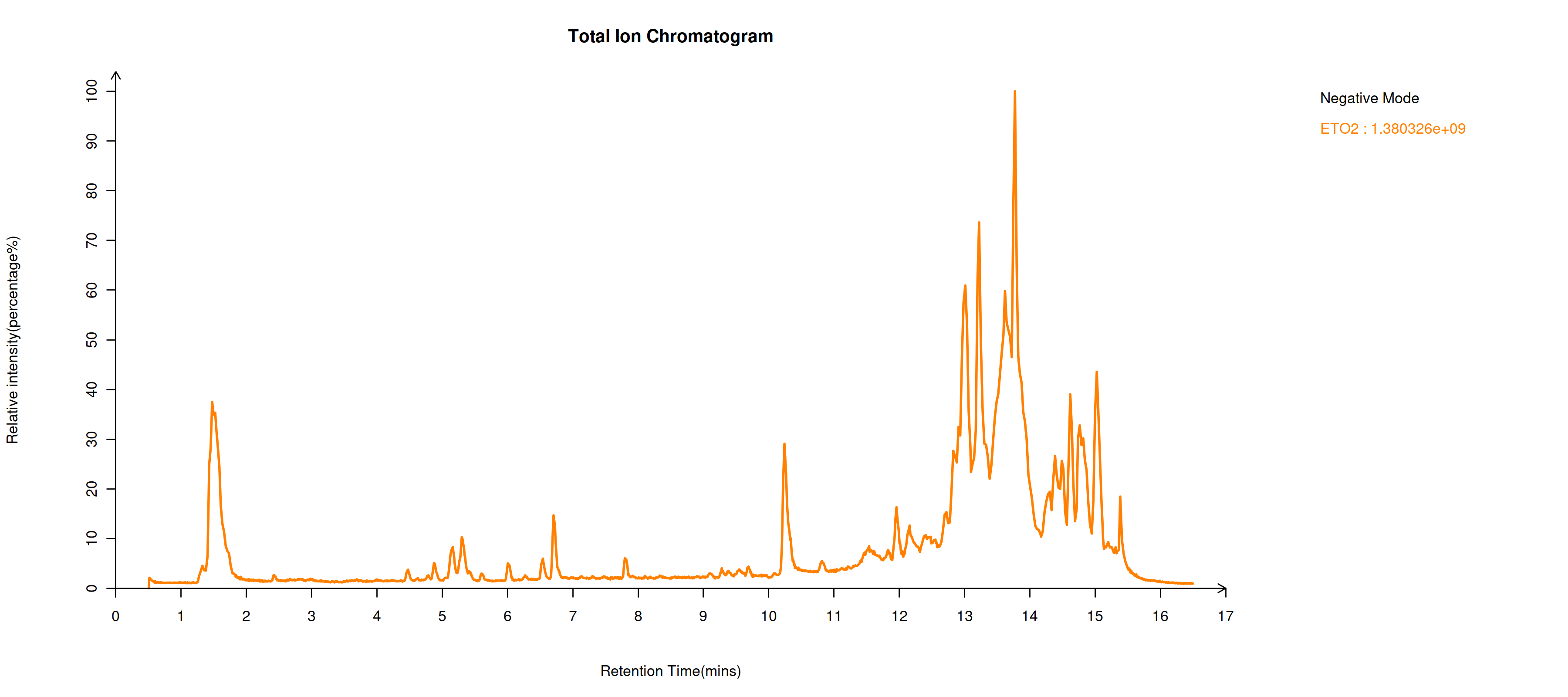

Supplement: Supplementary file 5 [file DataSheet1.ZIP › 1 TIC diagrams of all samples/Negative mode/ETO2.png]

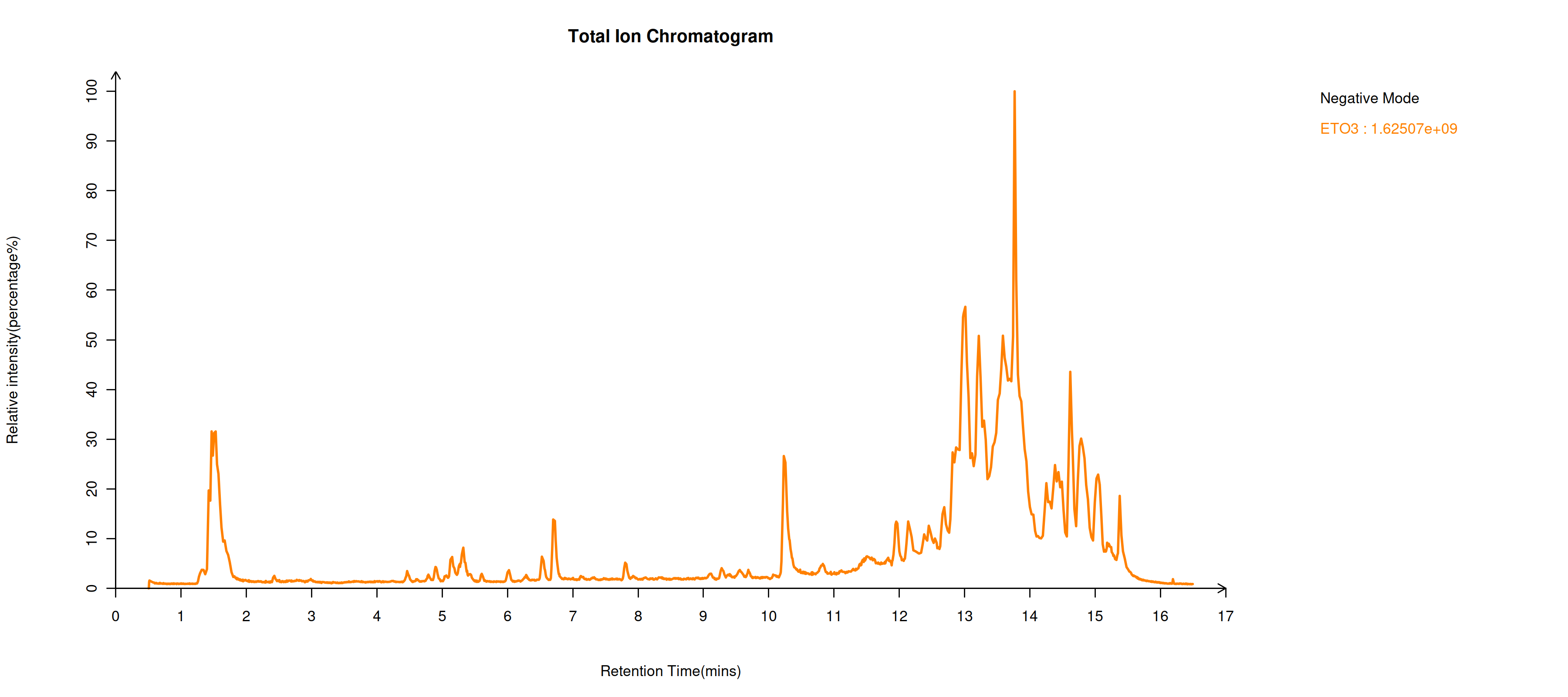

Supplement: Supplementary file 5 [file DataSheet1.ZIP › 1 TIC diagrams of all samples/Negative mode/ETO3.png]

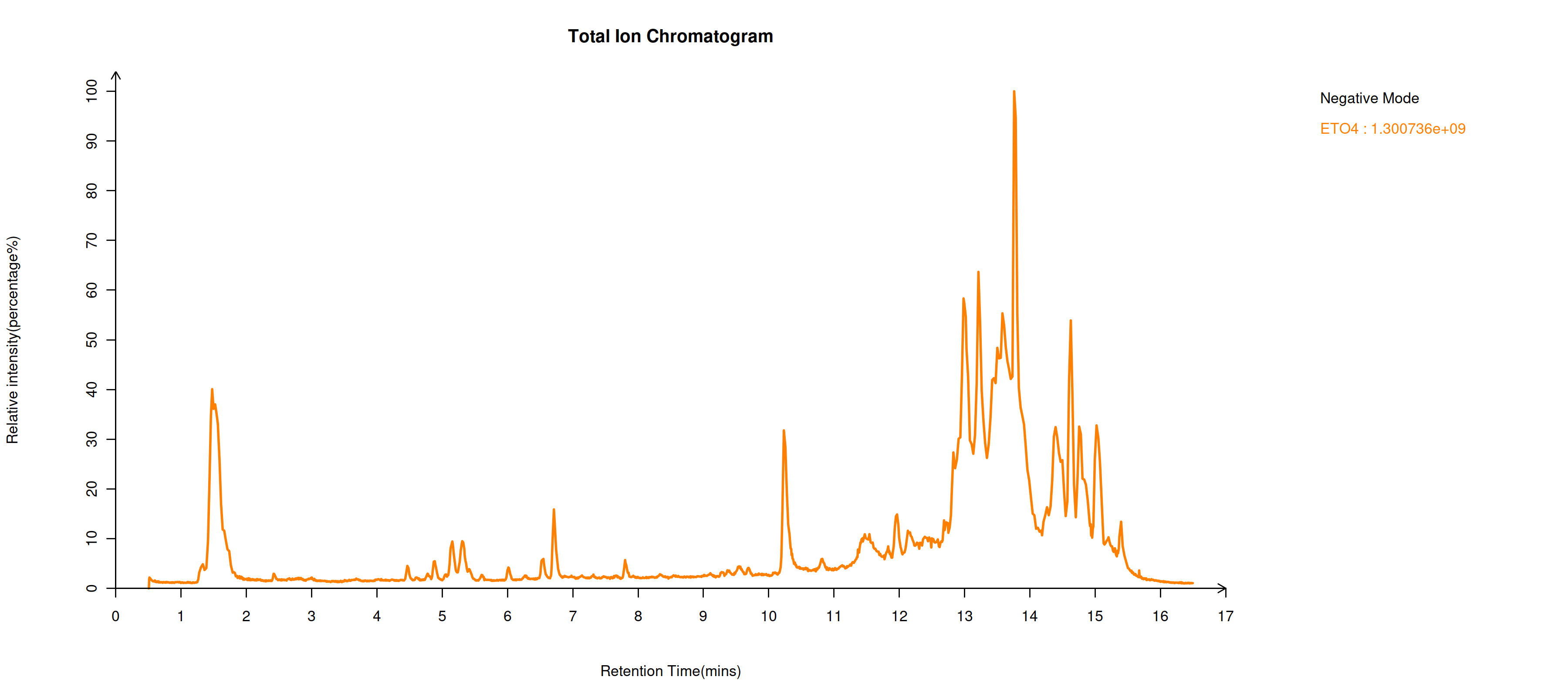

Supplement: Supplementary file 5 [file DataSheet1.ZIP › 1 TIC diagrams of all samples/Negative mode/ETO4.png]

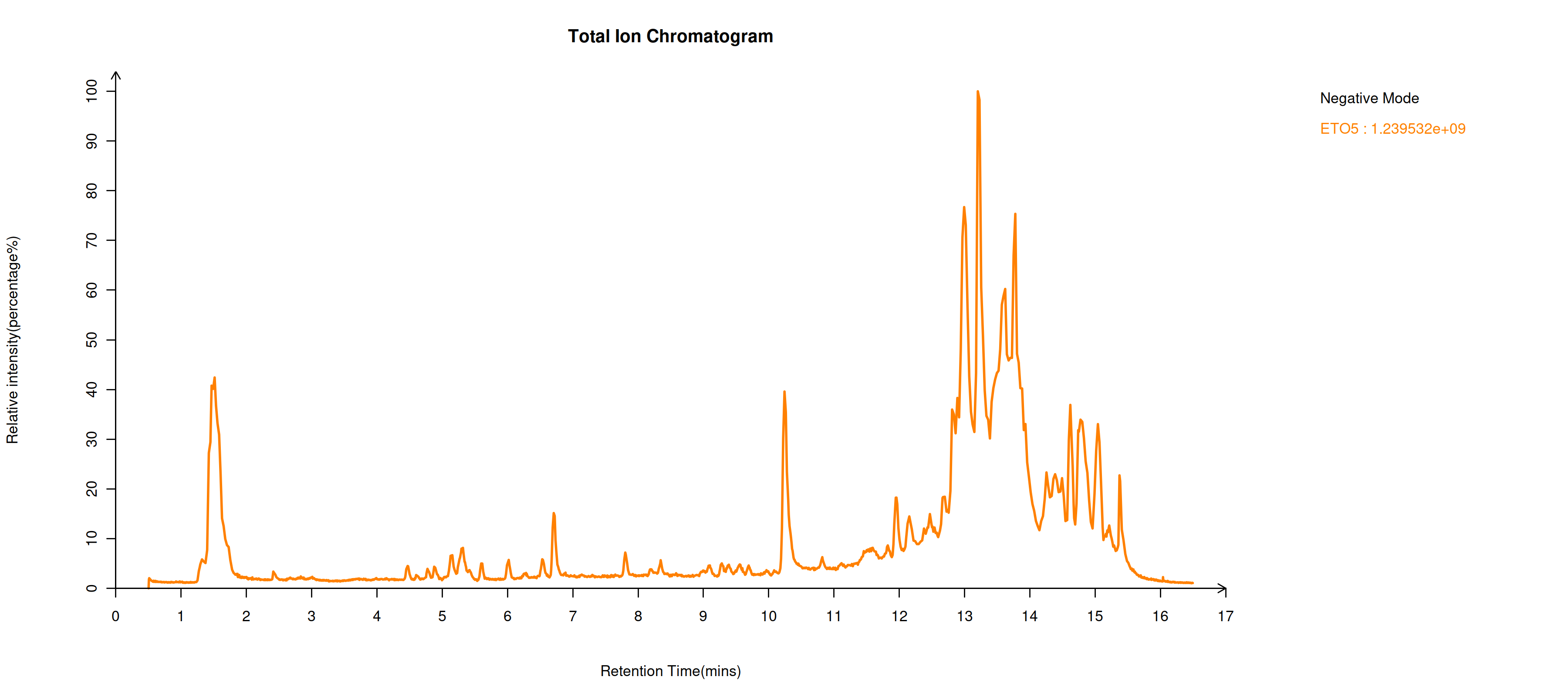

Supplement: Supplementary file 5 [file DataSheet1.ZIP › 1 TIC diagrams of all samples/Negative mode/ETO5.png]

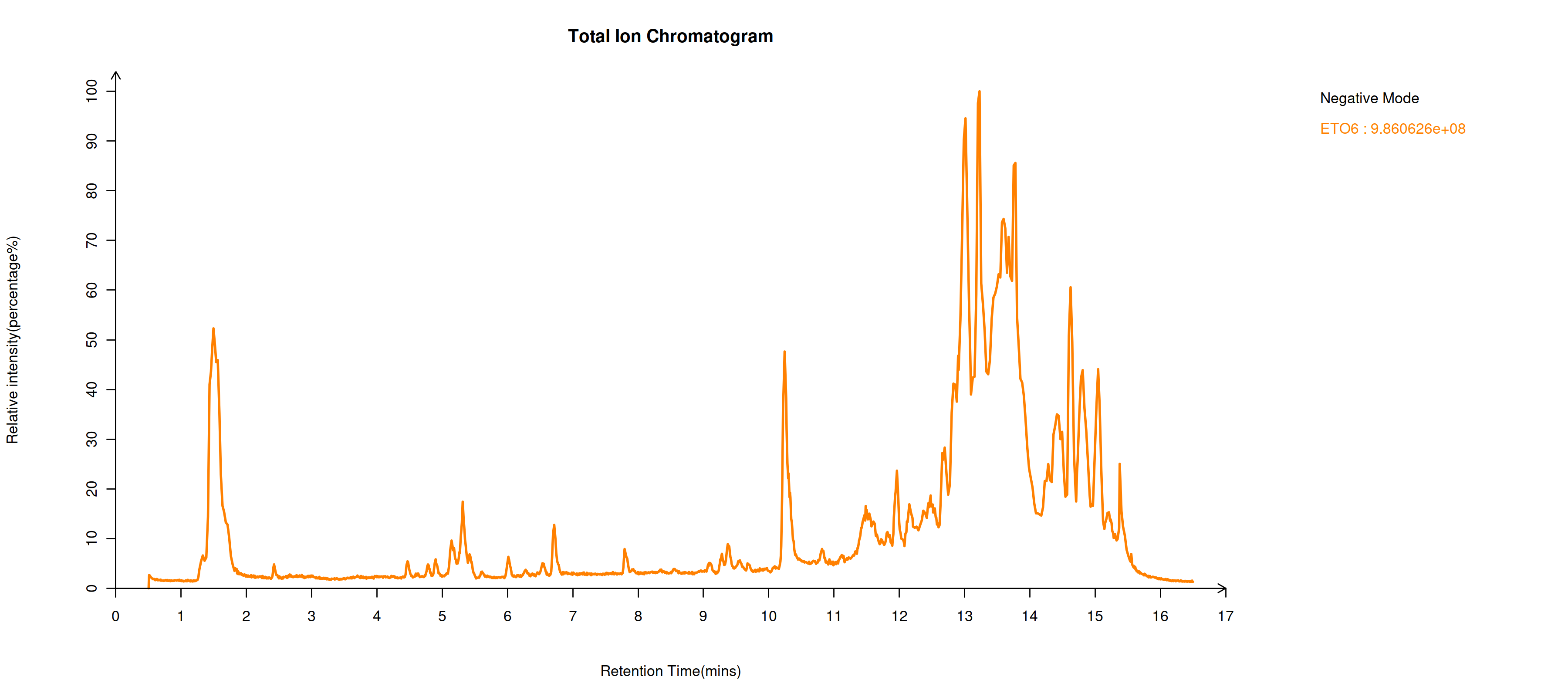

Supplement: Supplementary file 5 [file DataSheet1.ZIP › 1 TIC diagrams of all samples/Negative mode/ETO6.png]

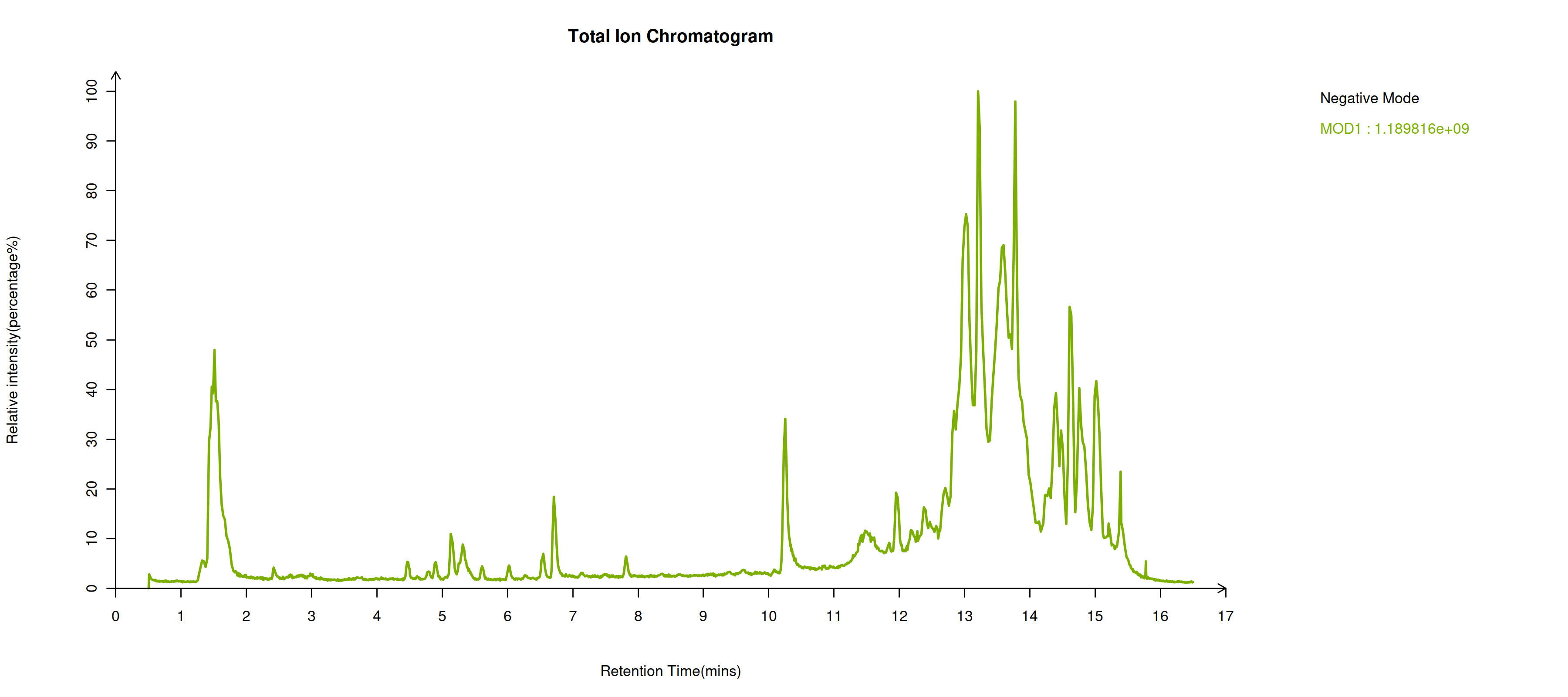

Supplement: Supplementary file 5 [file DataSheet1.ZIP › 1 TIC diagrams of all samples/Negative mode/MOD1.png]

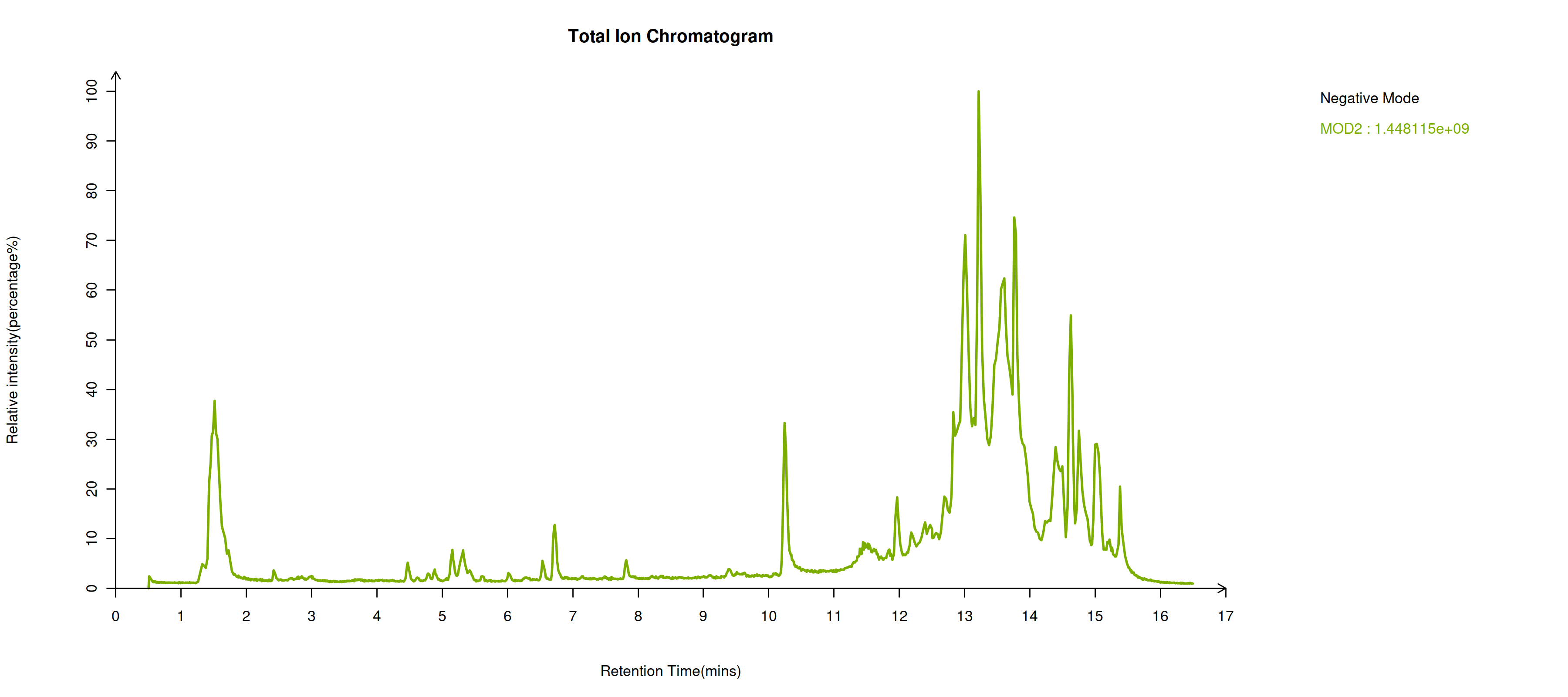

Supplement: Supplementary file 5 [file DataSheet1.ZIP › 1 TIC diagrams of all samples/Negative mode/MOD2.png]

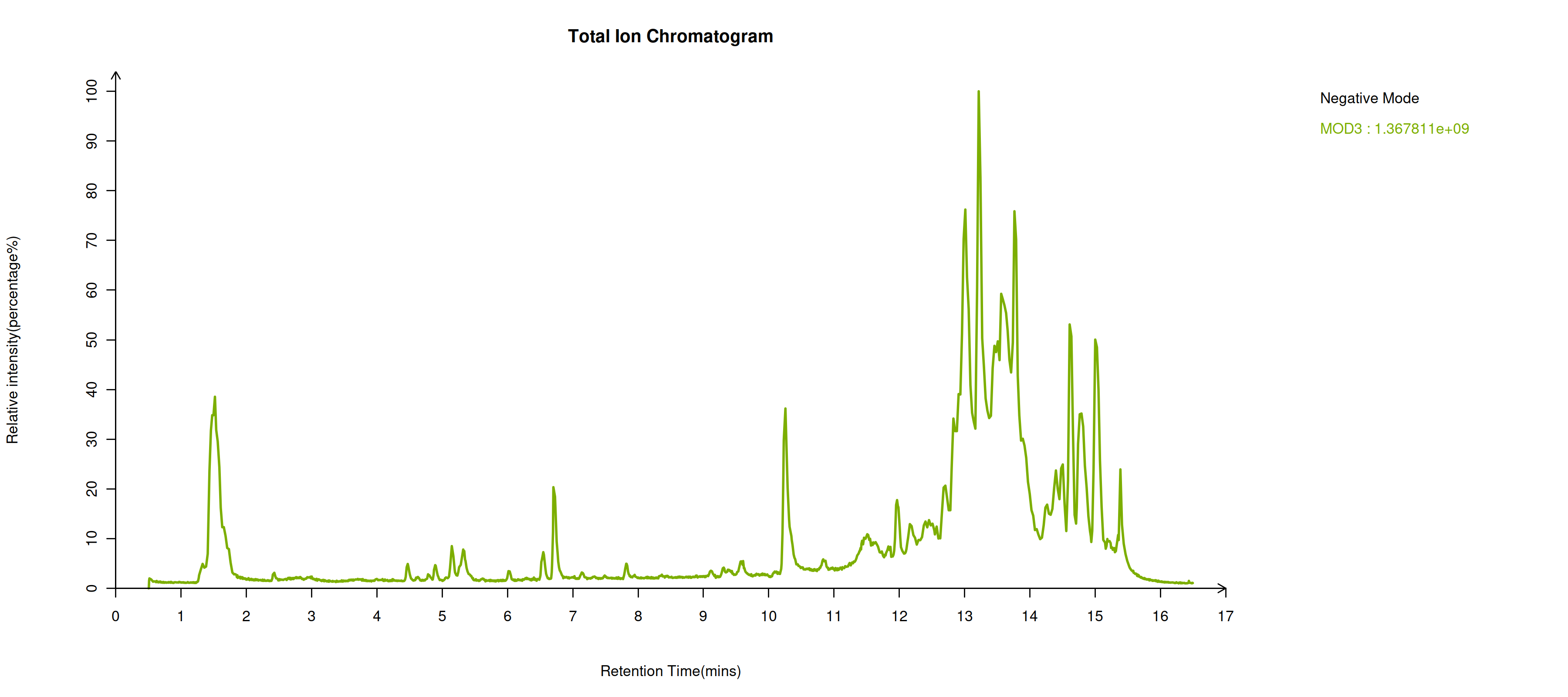

Supplement: Supplementary file 5 [file DataSheet1.ZIP › 1 TIC diagrams of all samples/Negative mode/MOD3.png]

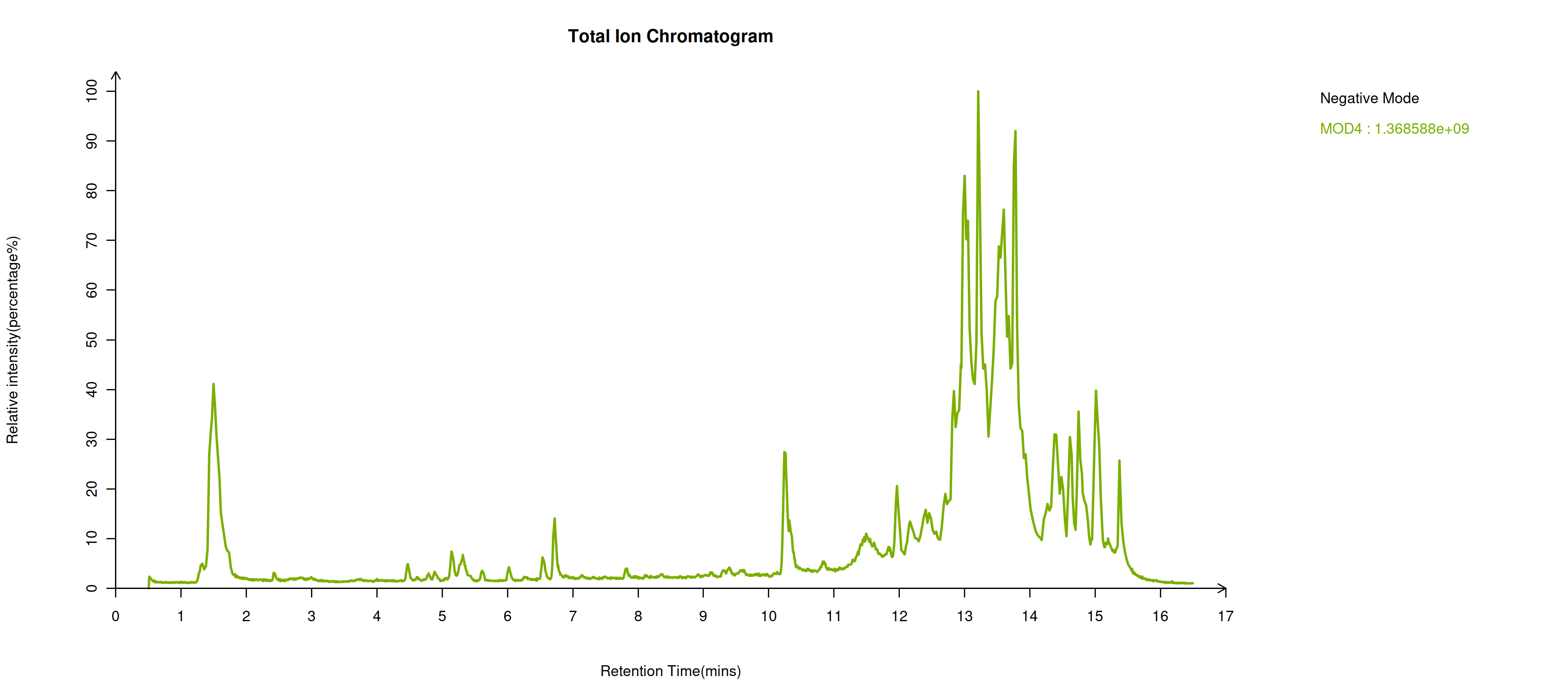

Supplement: Supplementary file 5 [file DataSheet1.ZIP › 1 TIC diagrams of all samples/Negative mode/MOD4.png]

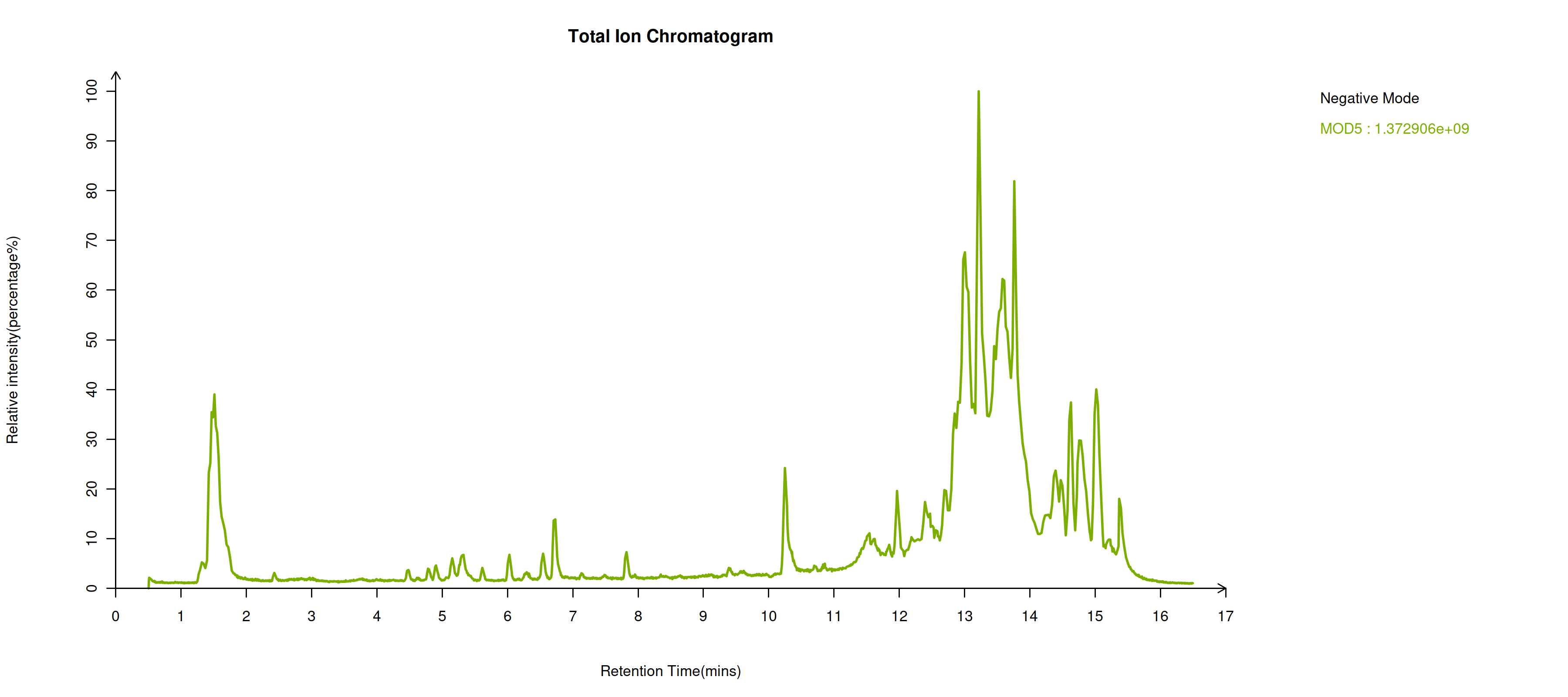

Supplement: Supplementary file 5 [file DataSheet1.ZIP › 1 TIC diagrams of all samples/Negative mode/MOD5.png]

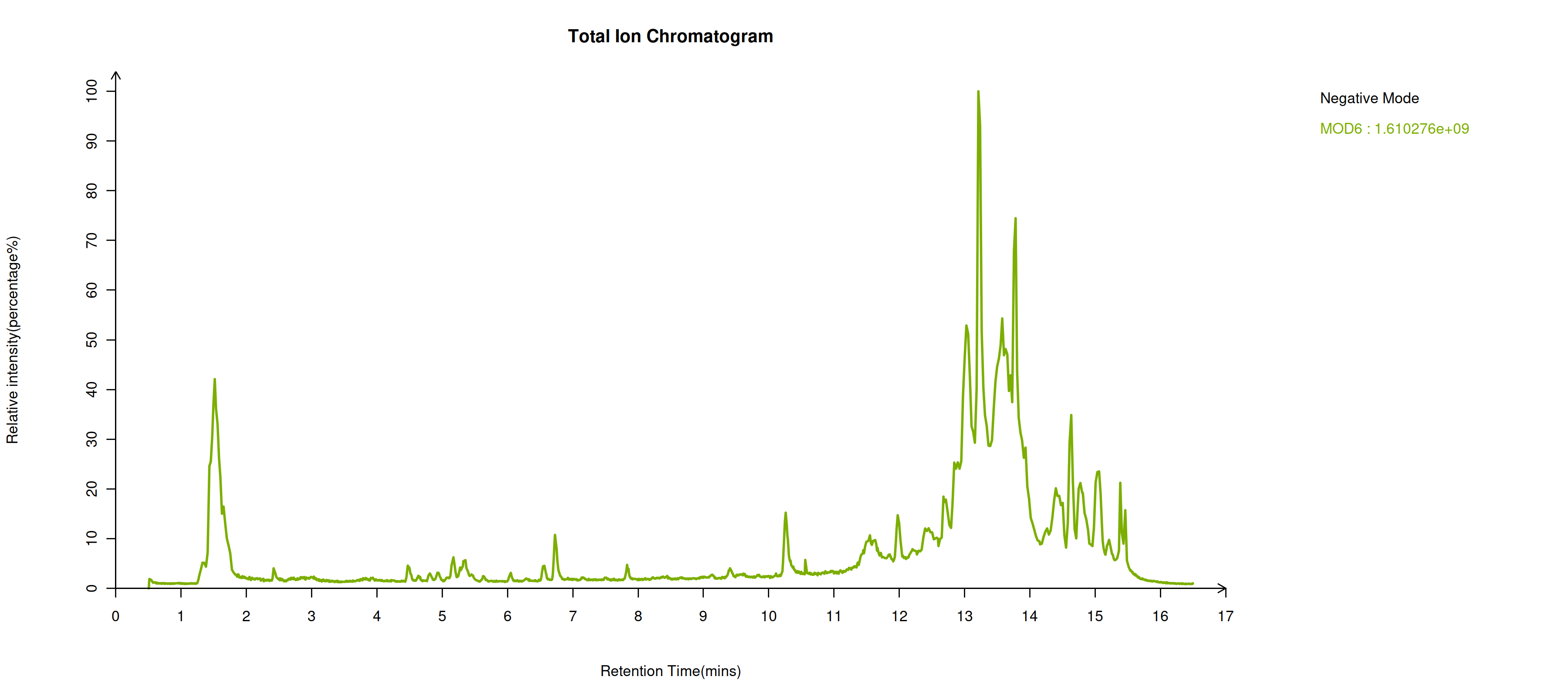

Supplement: Supplementary file 5 [file DataSheet1.ZIP › 1 TIC diagrams of all samples/Negative mode/MOD6.png]

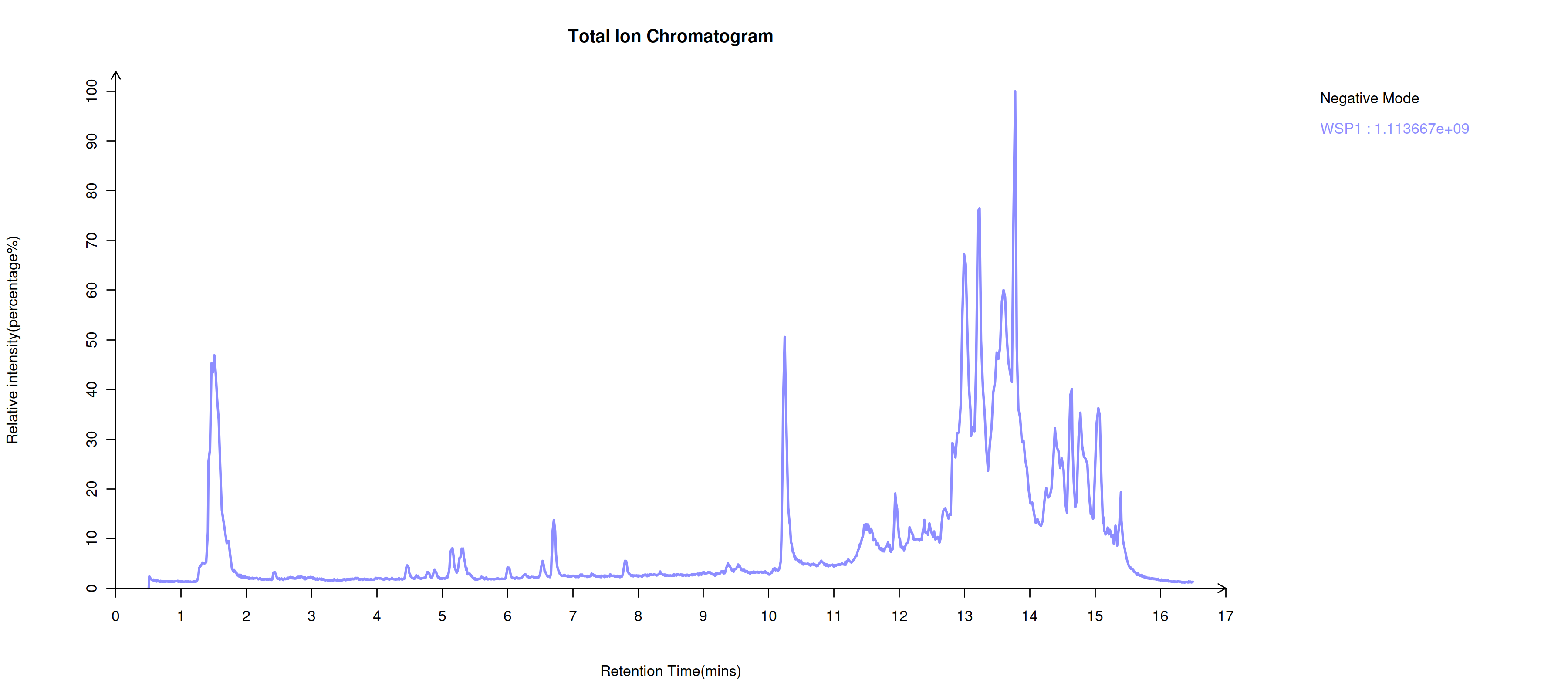

Supplement: Supplementary file 5 [file DataSheet1.ZIP › 1 TIC diagrams of all samples/Negative mode/WSP1.png]

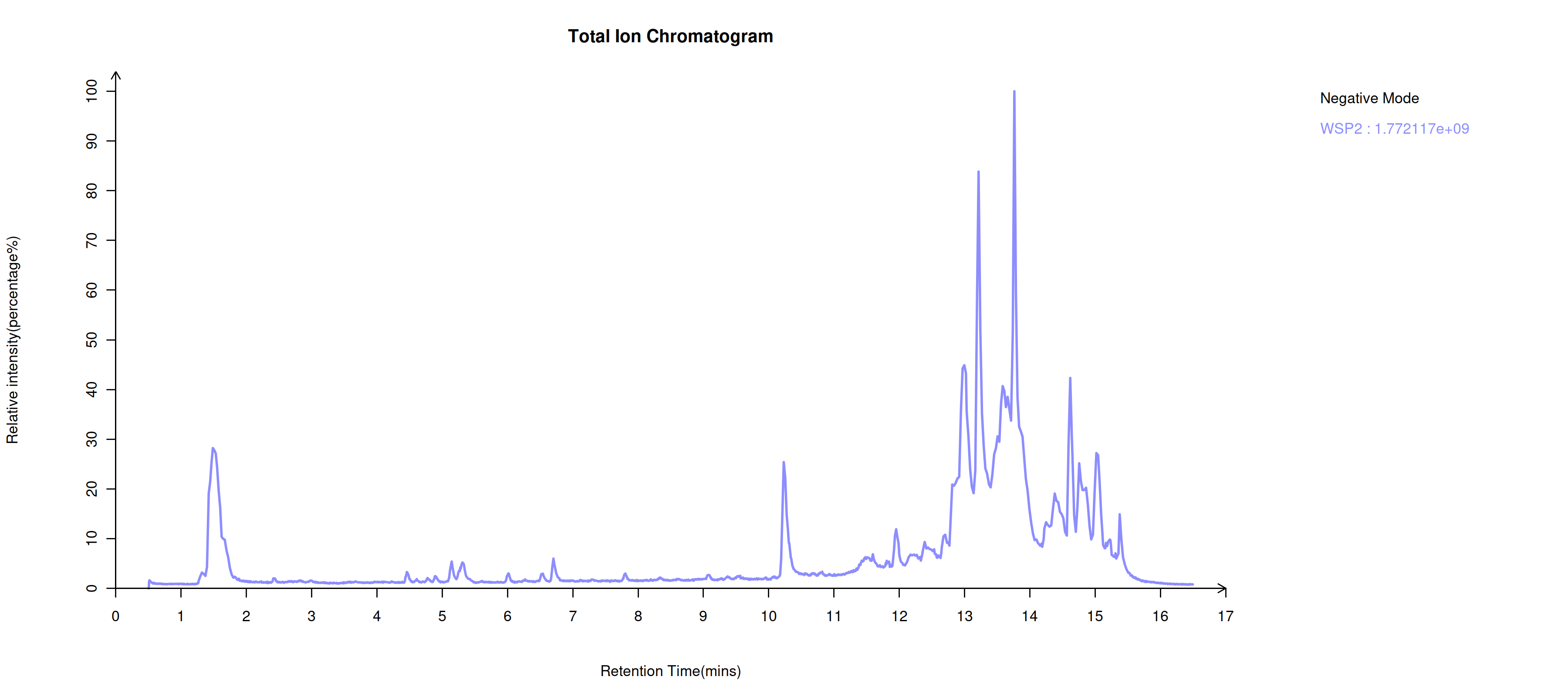

Supplement: Supplementary file 5 [file DataSheet1.ZIP › 1 TIC diagrams of all samples/Negative mode/WSP2.png]

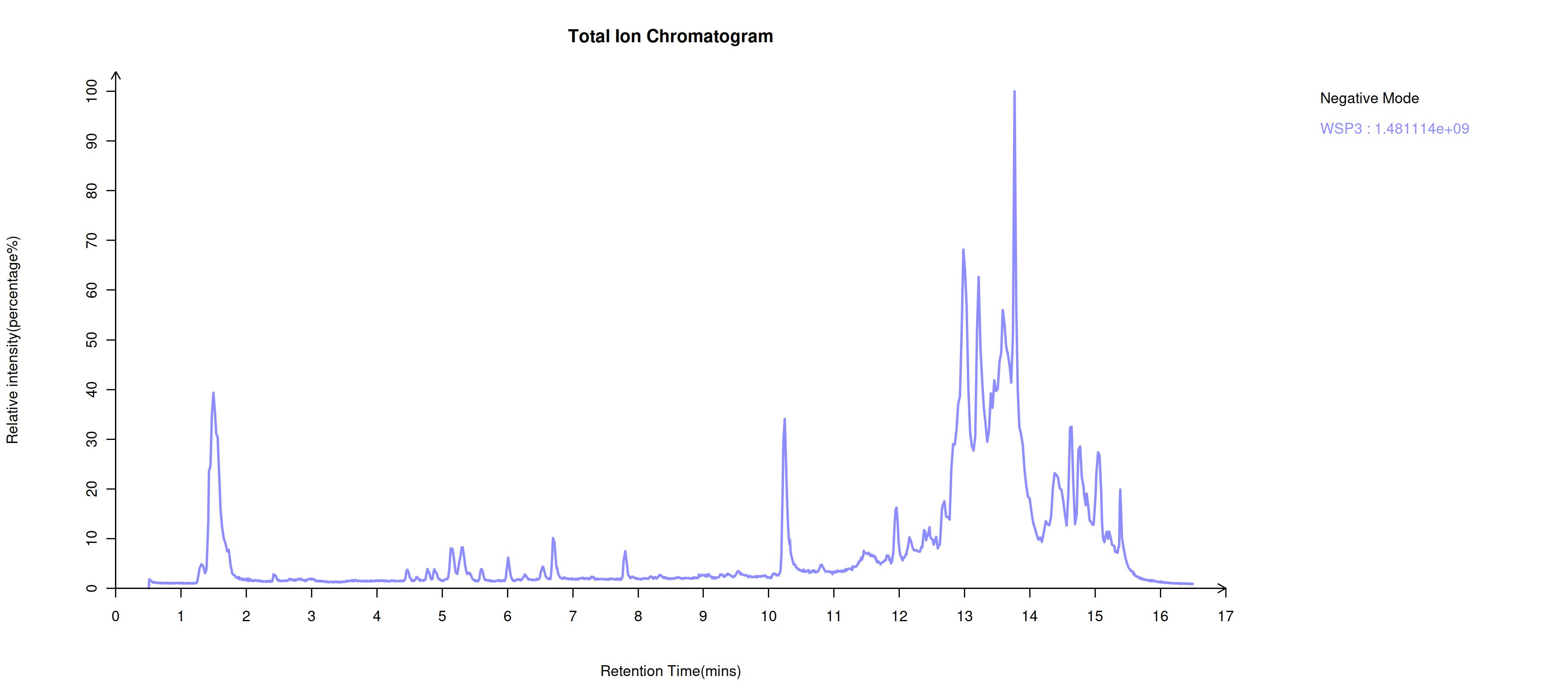

Supplement: Supplementary file 5 [file DataSheet1.ZIP › 1 TIC diagrams of all samples/Negative mode/WSP3.png]

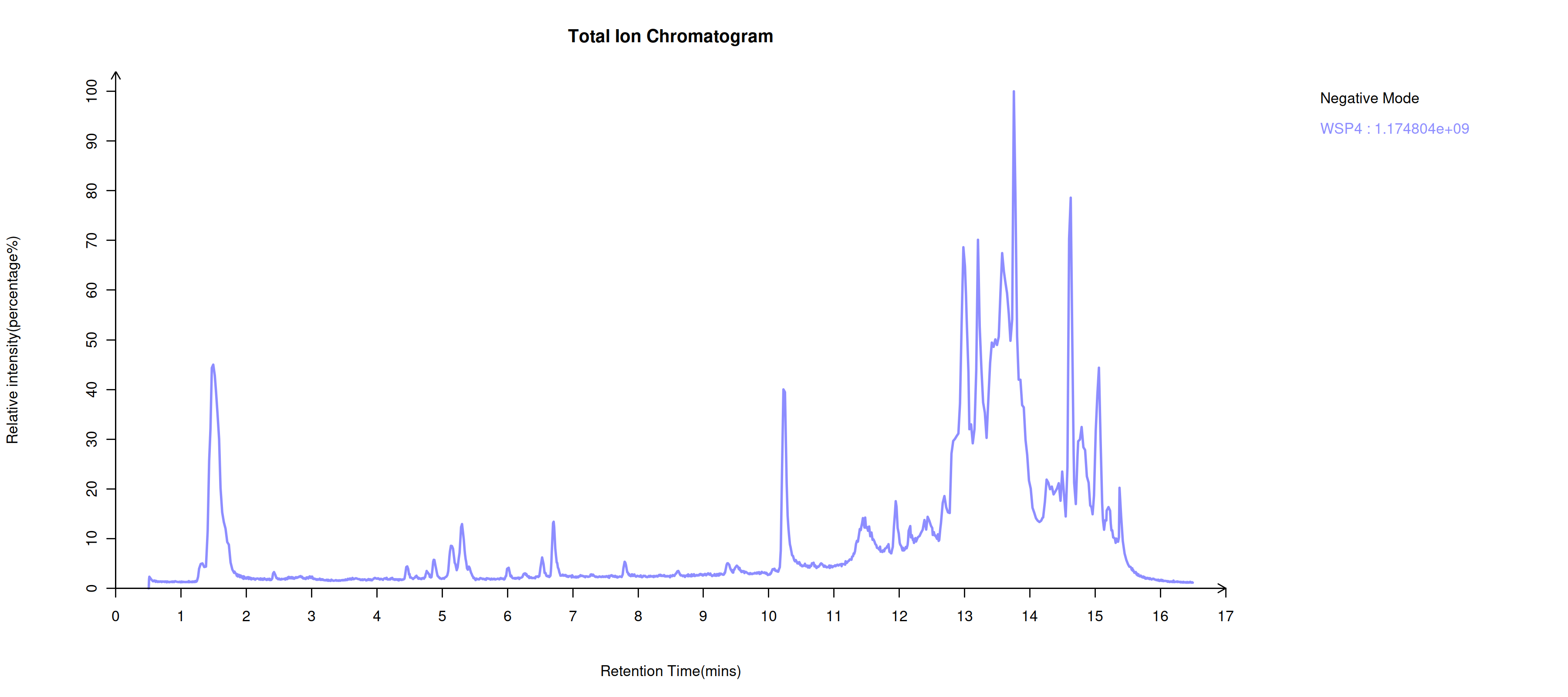

Supplement: Supplementary file 5 [file DataSheet1.ZIP › 1 TIC diagrams of all samples/Negative mode/WSP4.png]

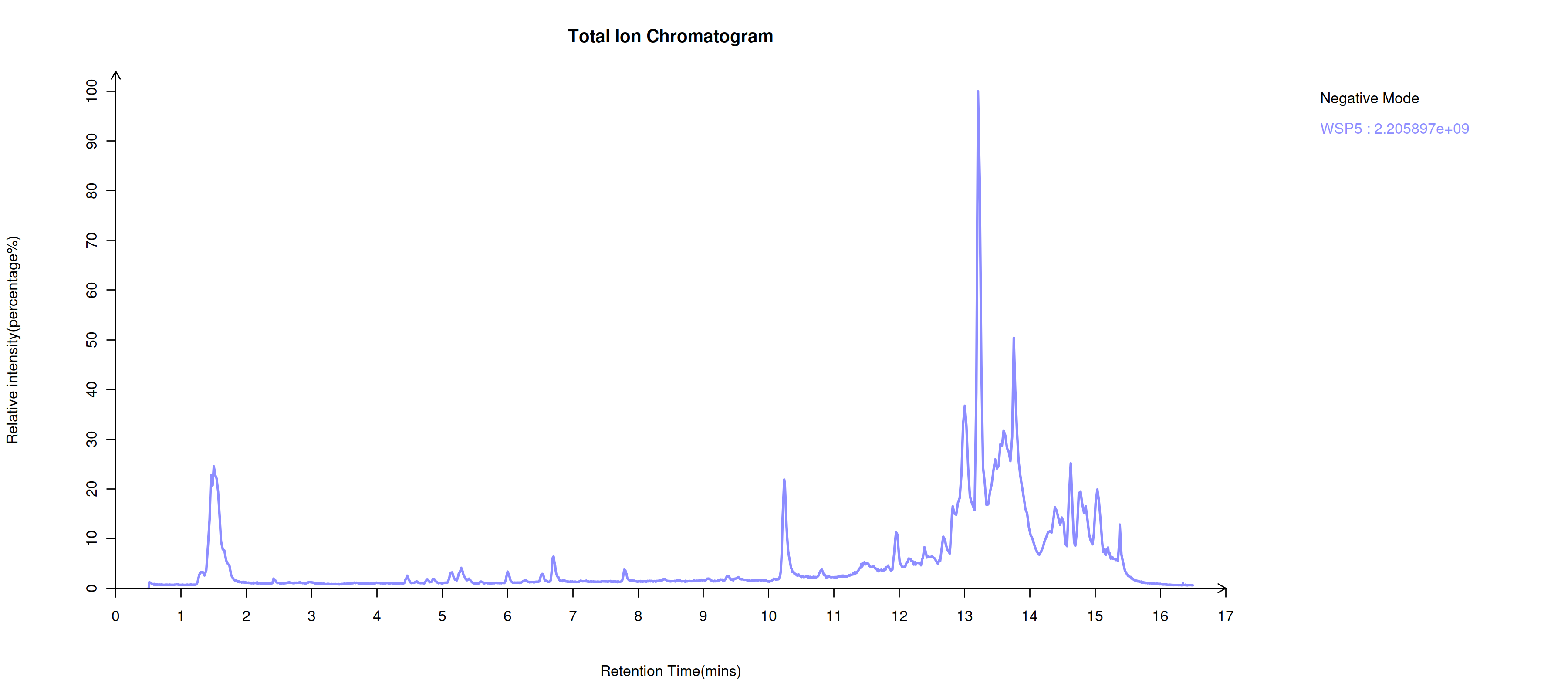

Supplement: Supplementary file 5 [file DataSheet1.ZIP › 1 TIC diagrams of all samples/Negative mode/WSP5.png]

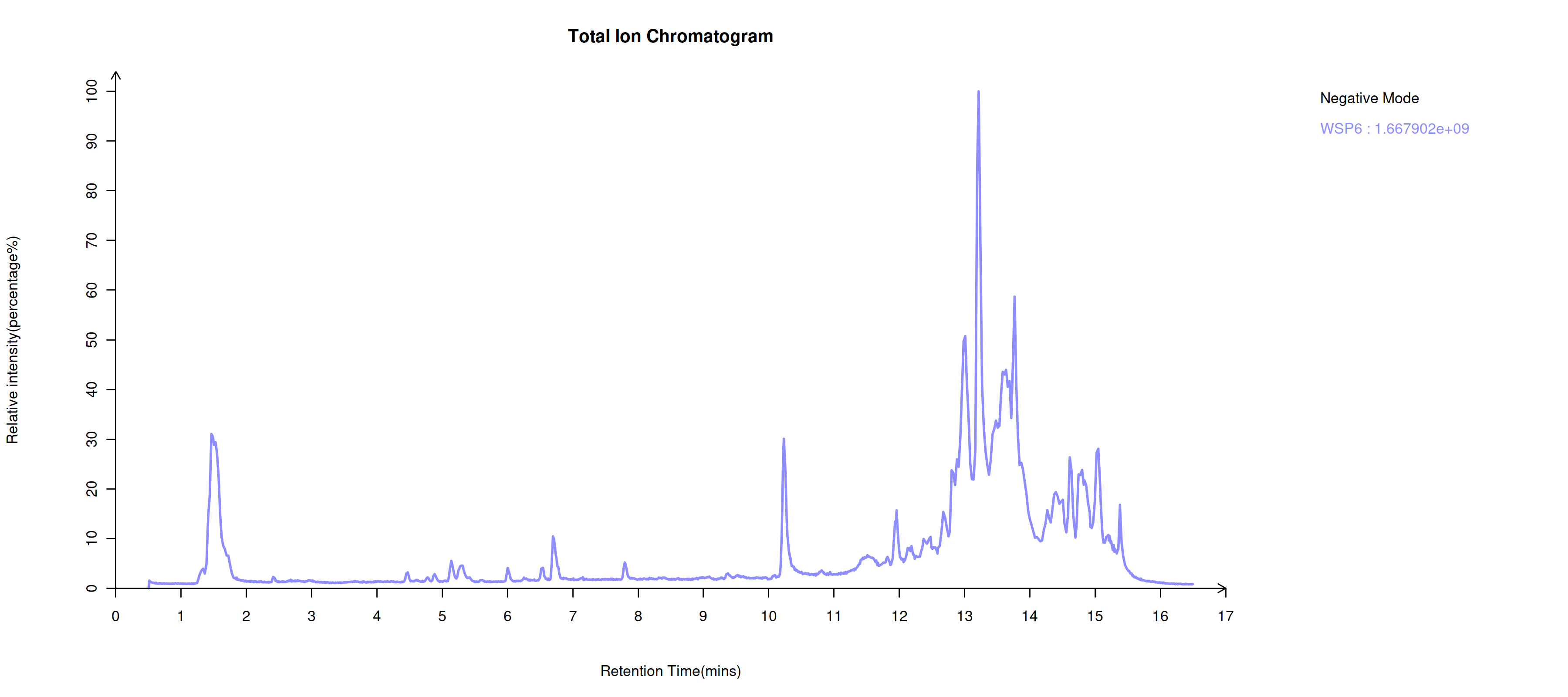

Supplement: Supplementary file 5 [file DataSheet1.ZIP › 1 TIC diagrams of all samples/Negative mode/WSP6.png]

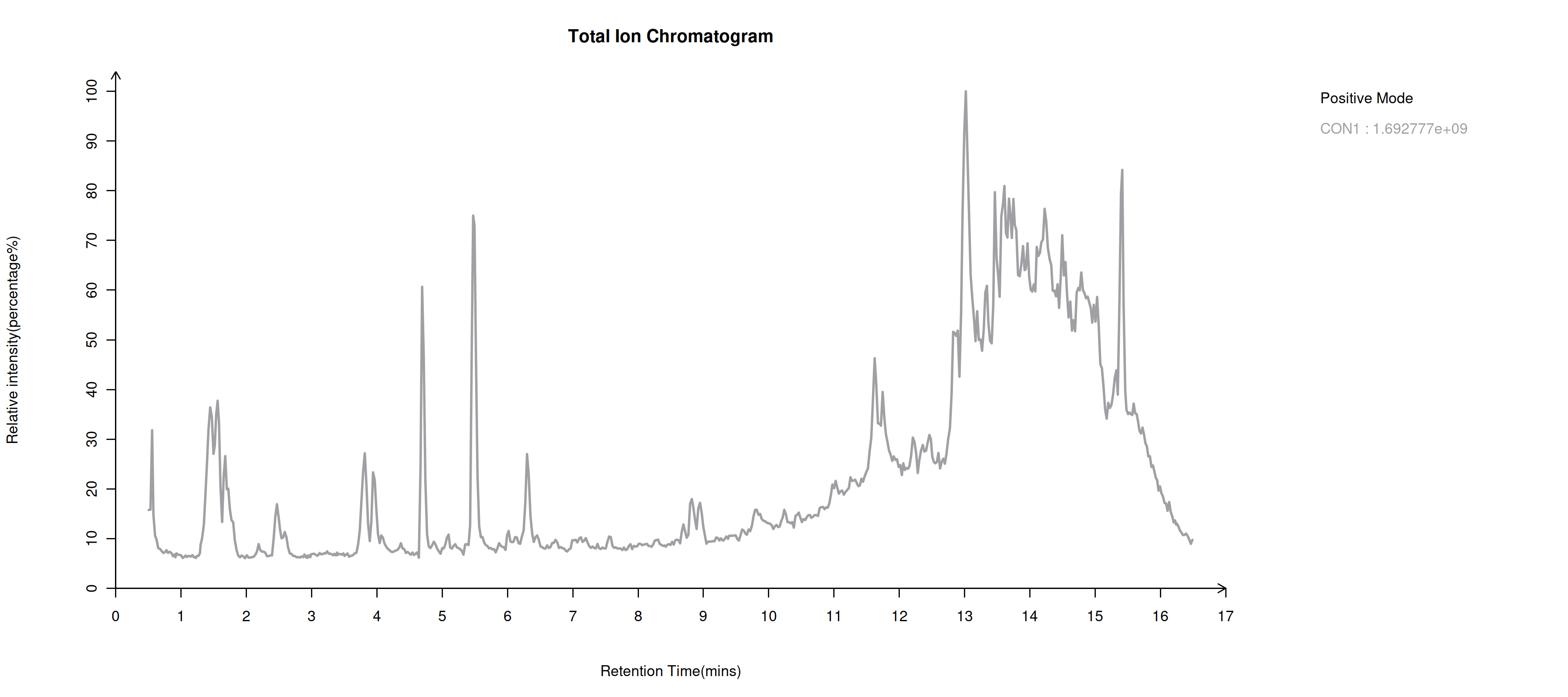

Supplement: Supplementary file 5 [file DataSheet1.ZIP › 1 TIC diagrams of all samples/Positive mode/CON1.png]

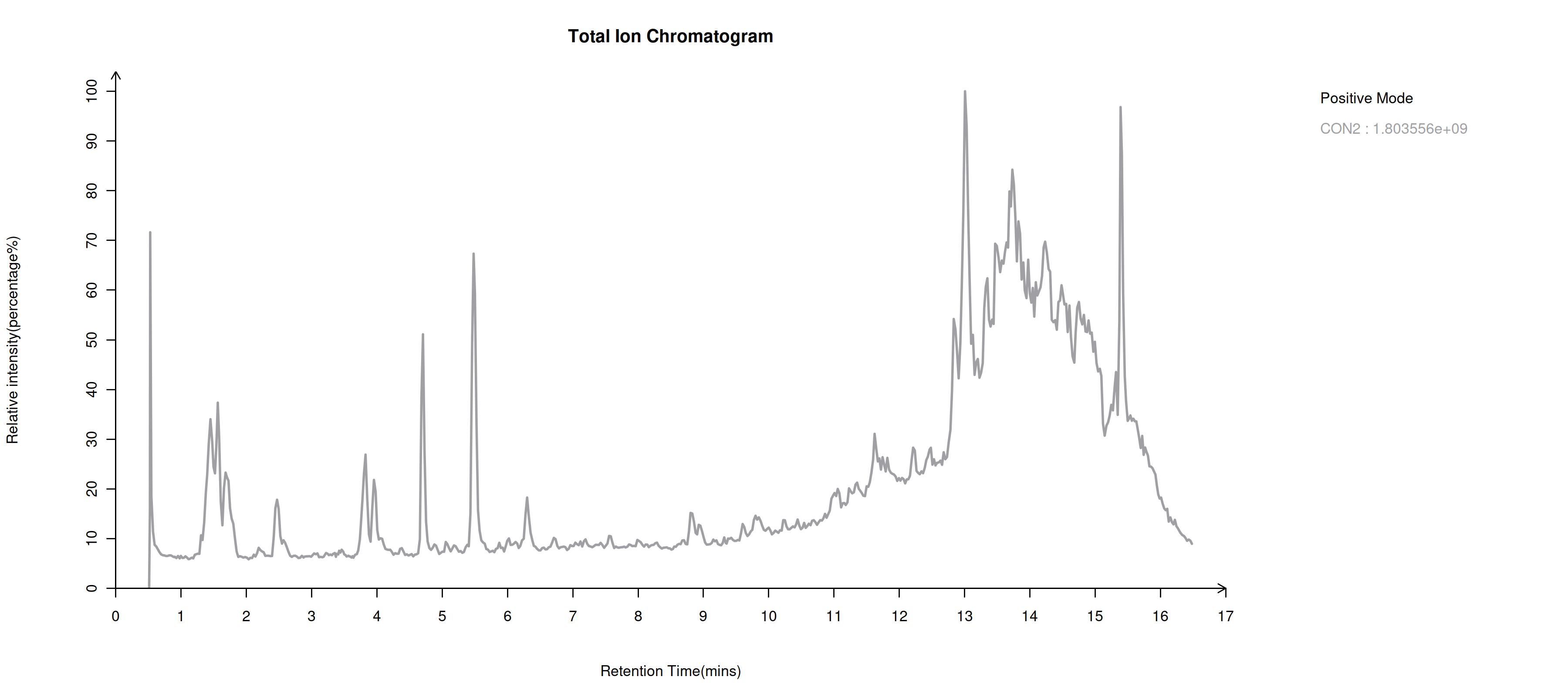

Supplement: Supplementary file 5 [file DataSheet1.ZIP › 1 TIC diagrams of all samples/Positive mode/CON2.png]

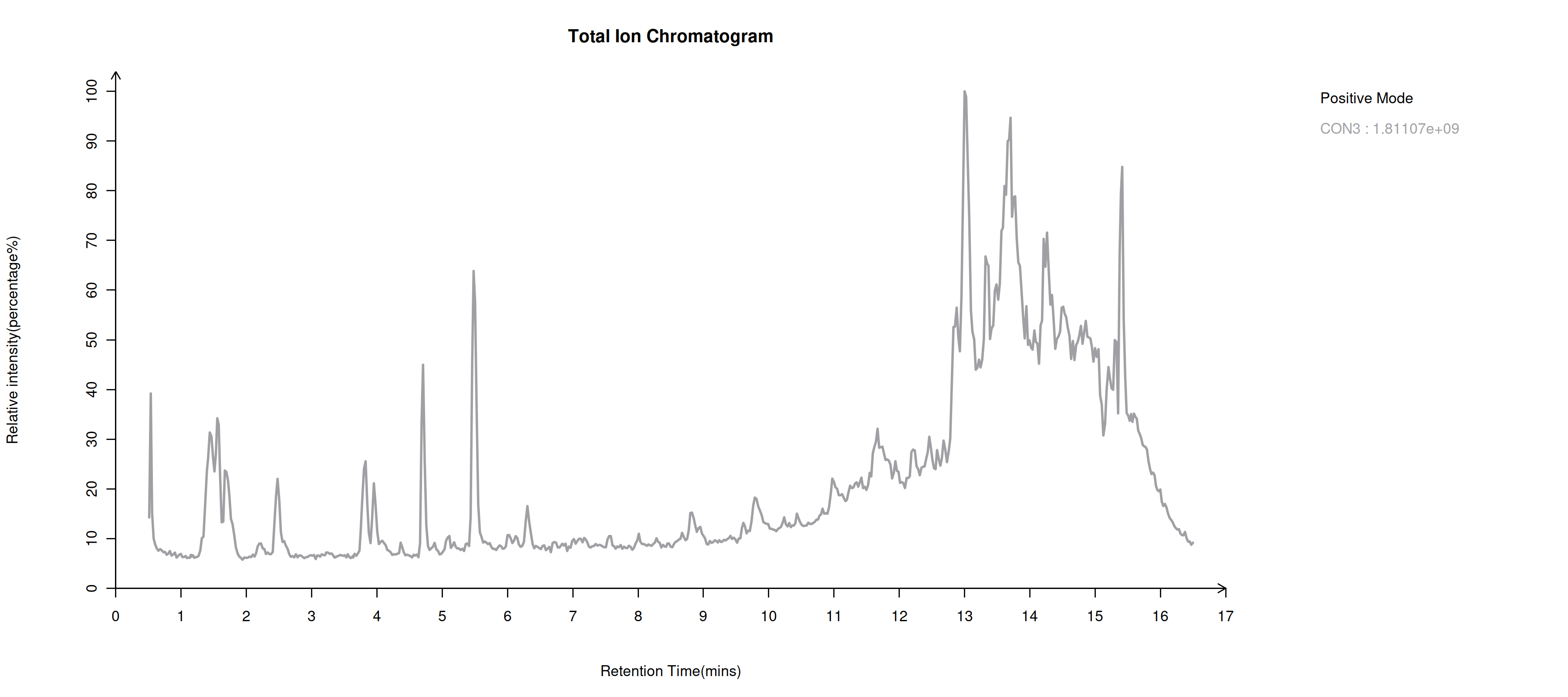

Supplement: Supplementary file 5 [file DataSheet1.ZIP › 1 TIC diagrams of all samples/Positive mode/CON3.png]

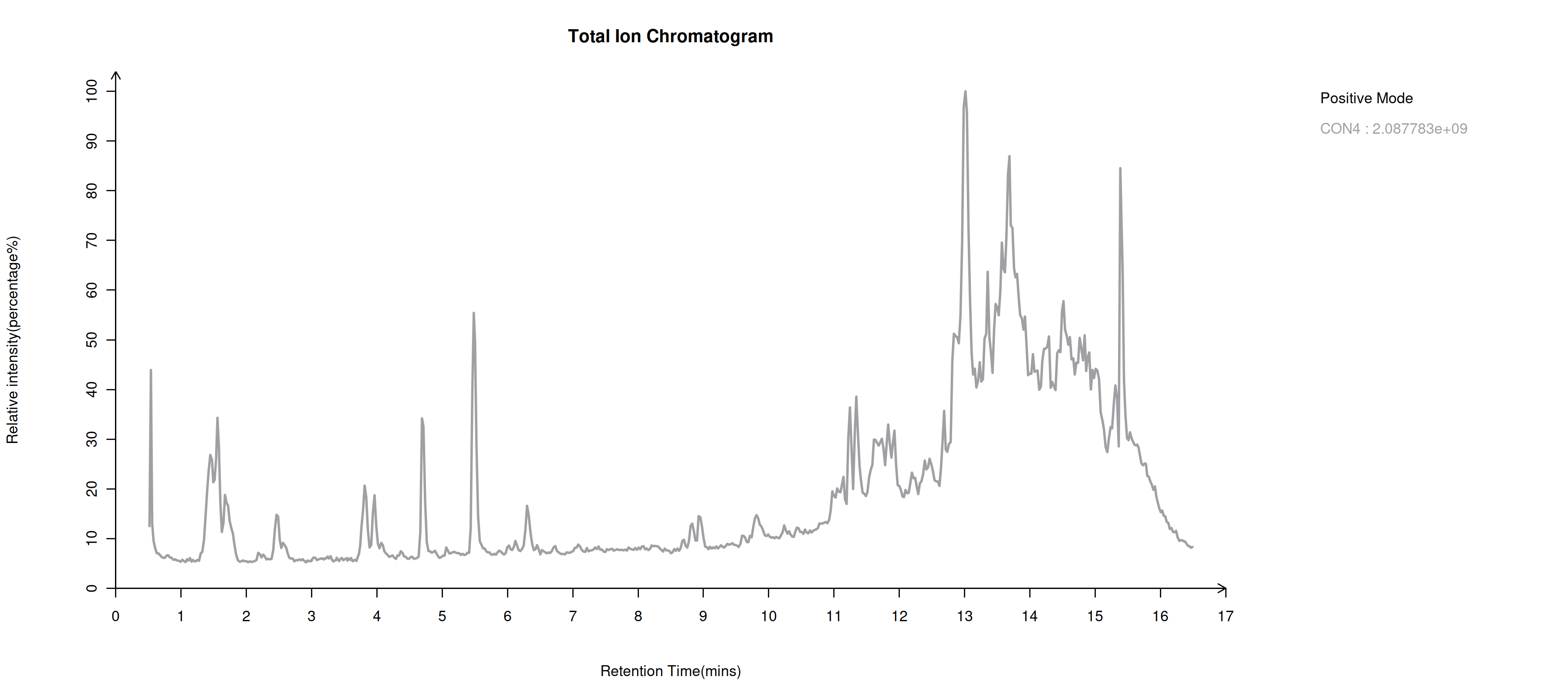

Supplement: Supplementary file 5 [file DataSheet1.ZIP › 1 TIC diagrams of all samples/Positive mode/CON4.png]

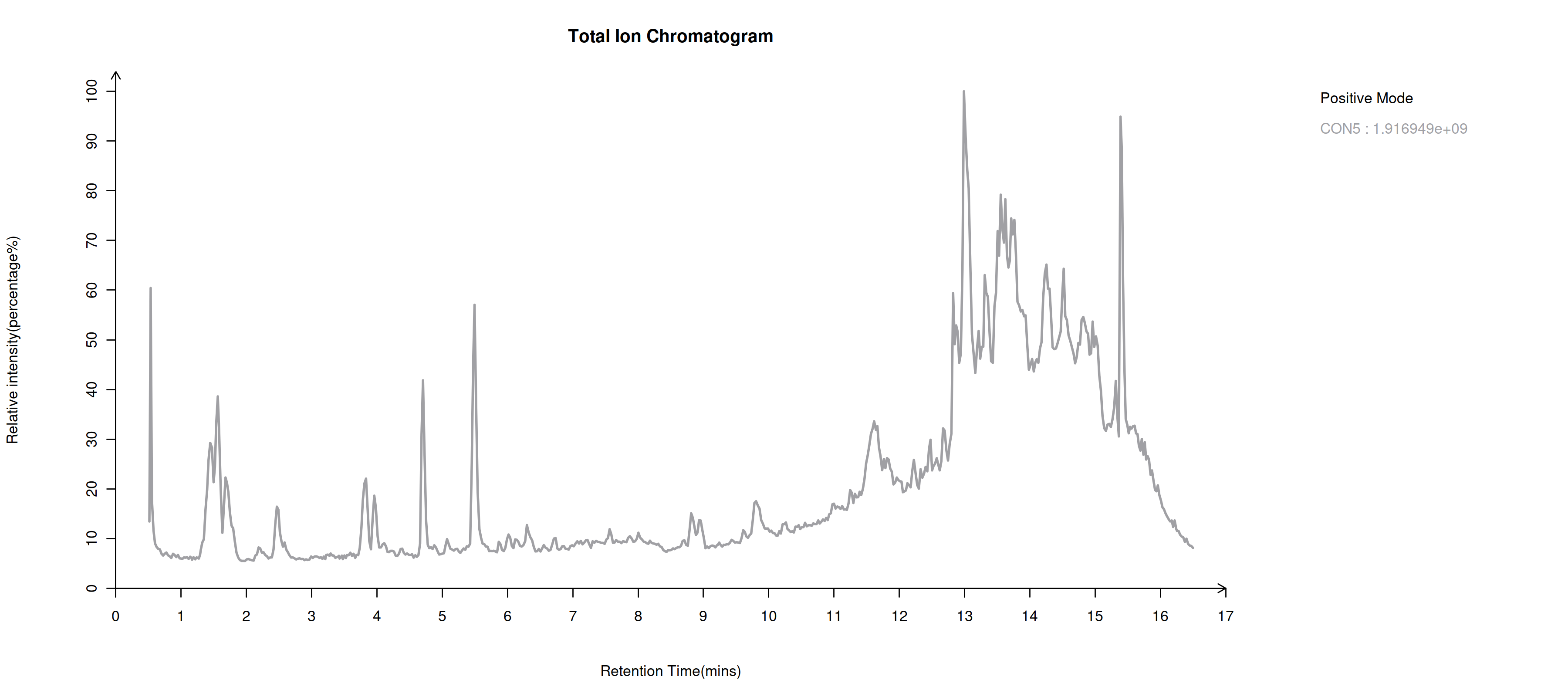

Supplement: Supplementary file 5 [file DataSheet1.ZIP › 1 TIC diagrams of all samples/Positive mode/CON5.png]

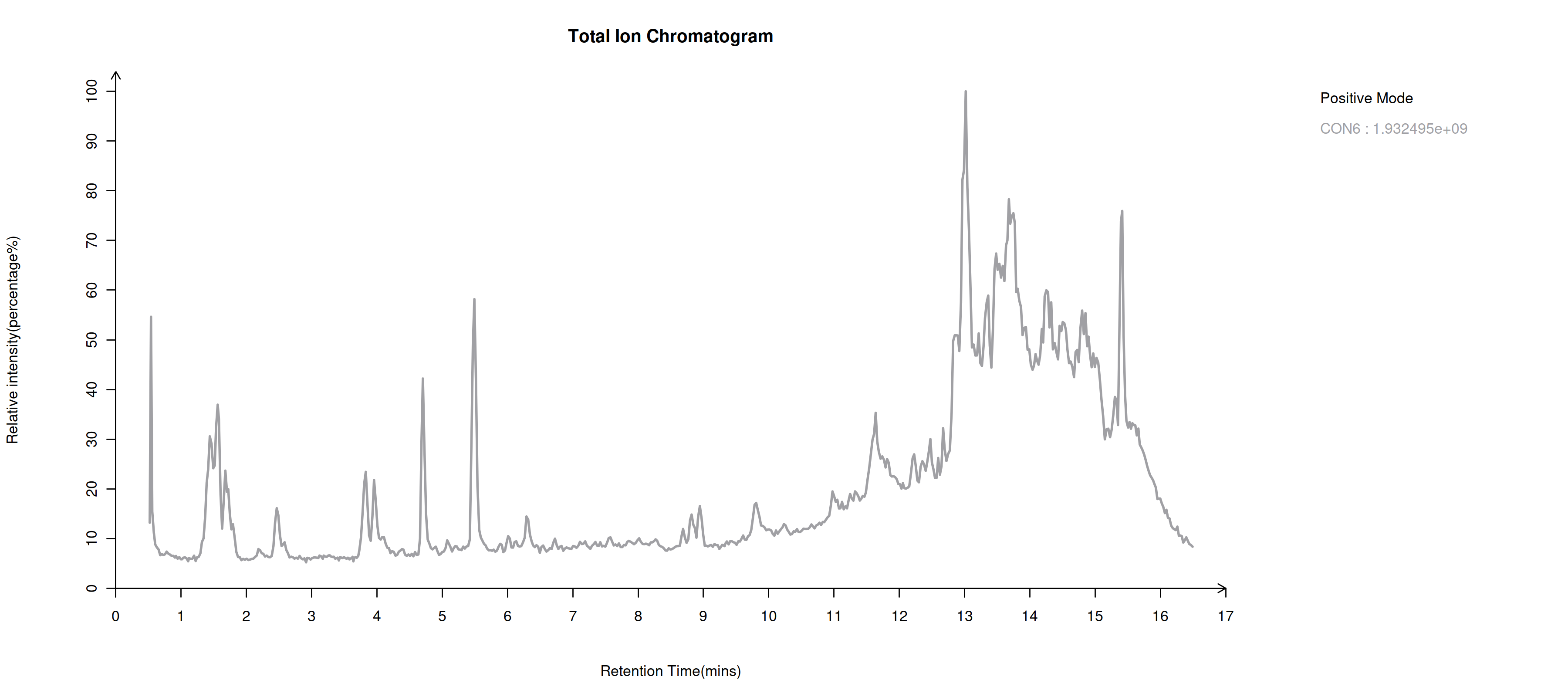

Supplement: Supplementary file 5 [file DataSheet1.ZIP › 1 TIC diagrams of all samples/Positive mode/CON6.png]

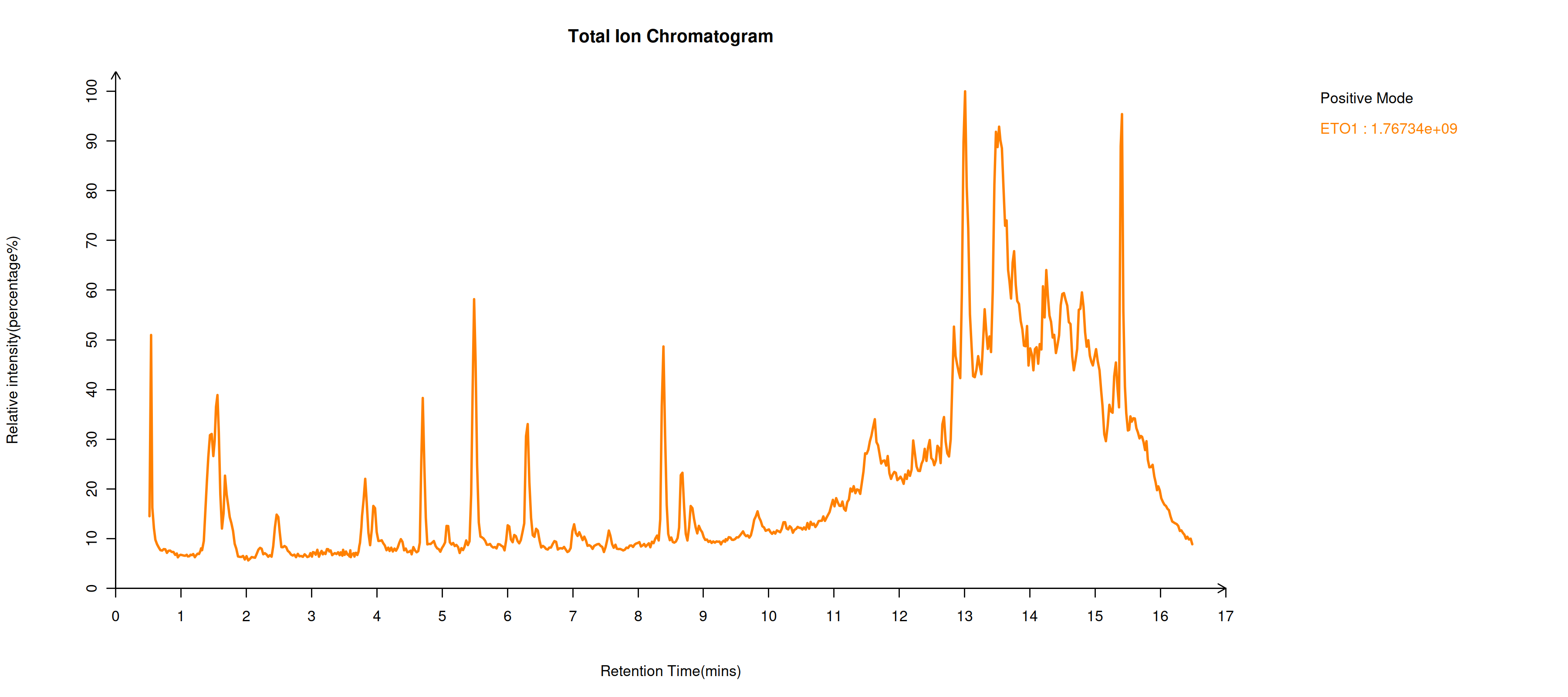

Supplement: Supplementary file 5 [file DataSheet1.ZIP › 1 TIC diagrams of all samples/Positive mode/ETO1.png]

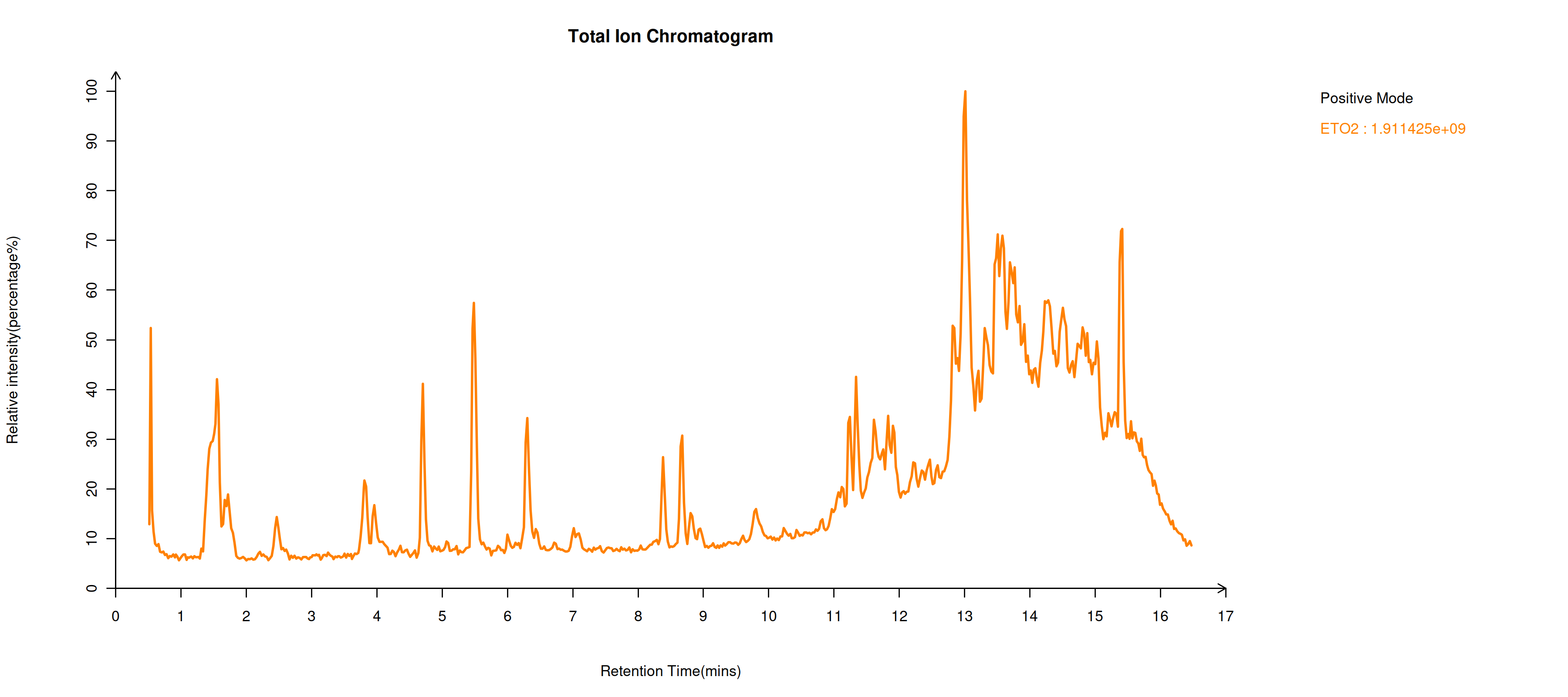

Supplement: Supplementary file 5 [file DataSheet1.ZIP › 1 TIC diagrams of all samples/Positive mode/ETO2.png]

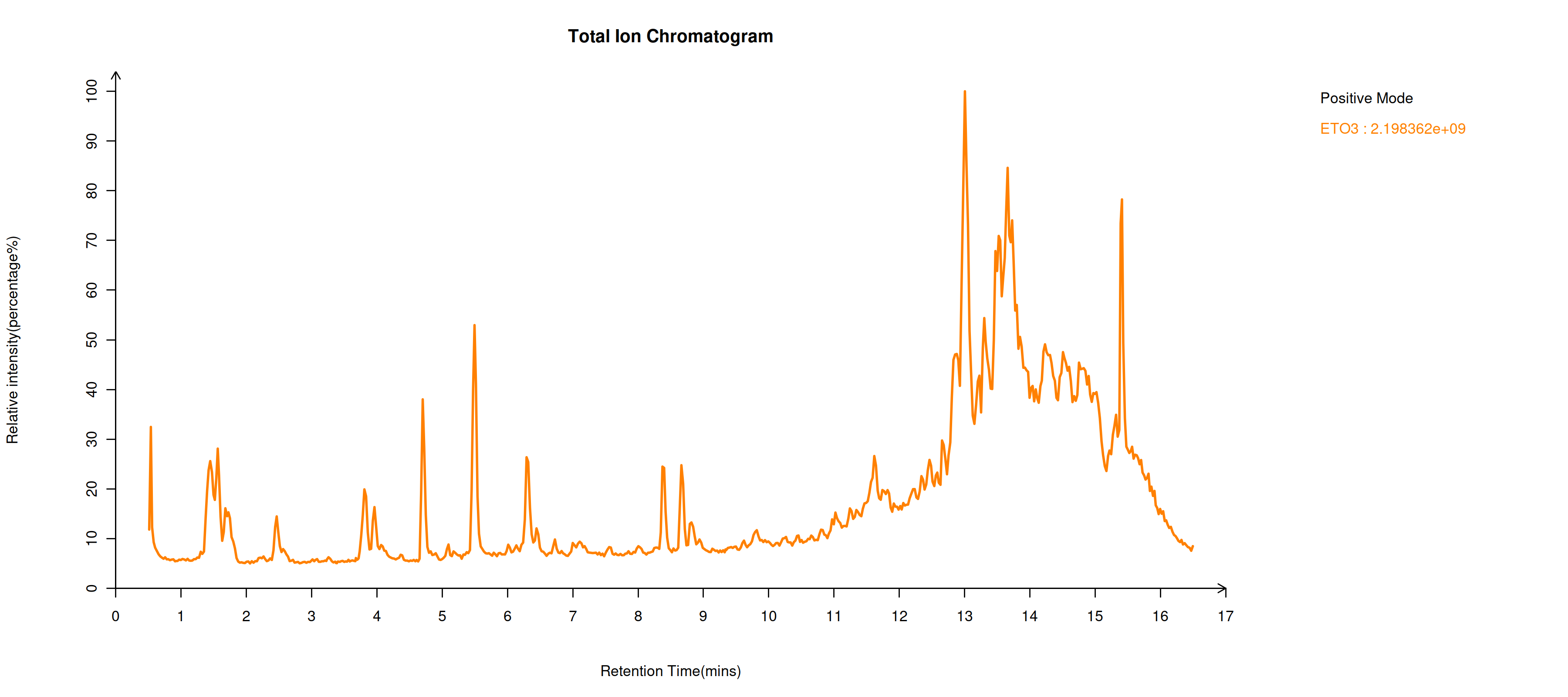

Supplement: Supplementary file 5 [file DataSheet1.ZIP › 1 TIC diagrams of all samples/Positive mode/ETO3.png]

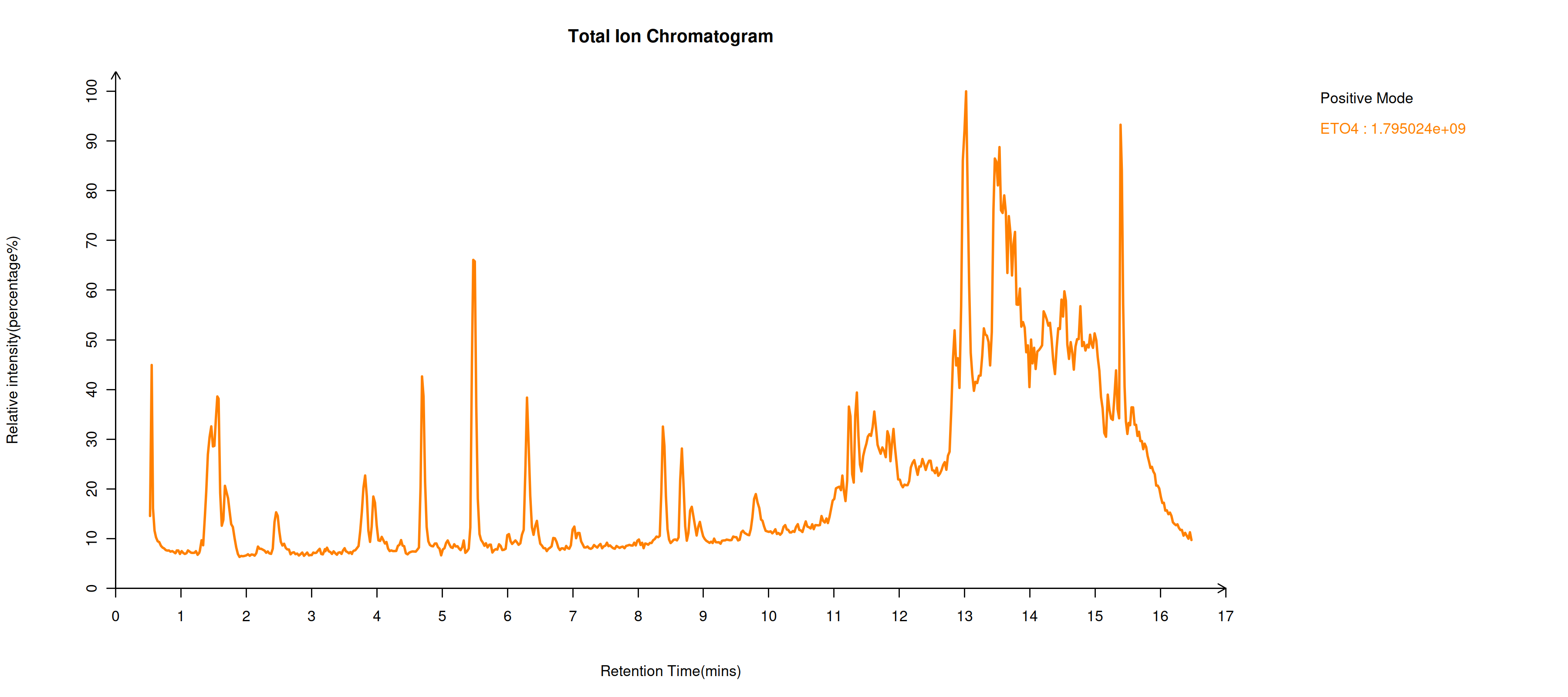

Supplement: Supplementary file 5 [file DataSheet1.ZIP › 1 TIC diagrams of all samples/Positive mode/ETO4.png]

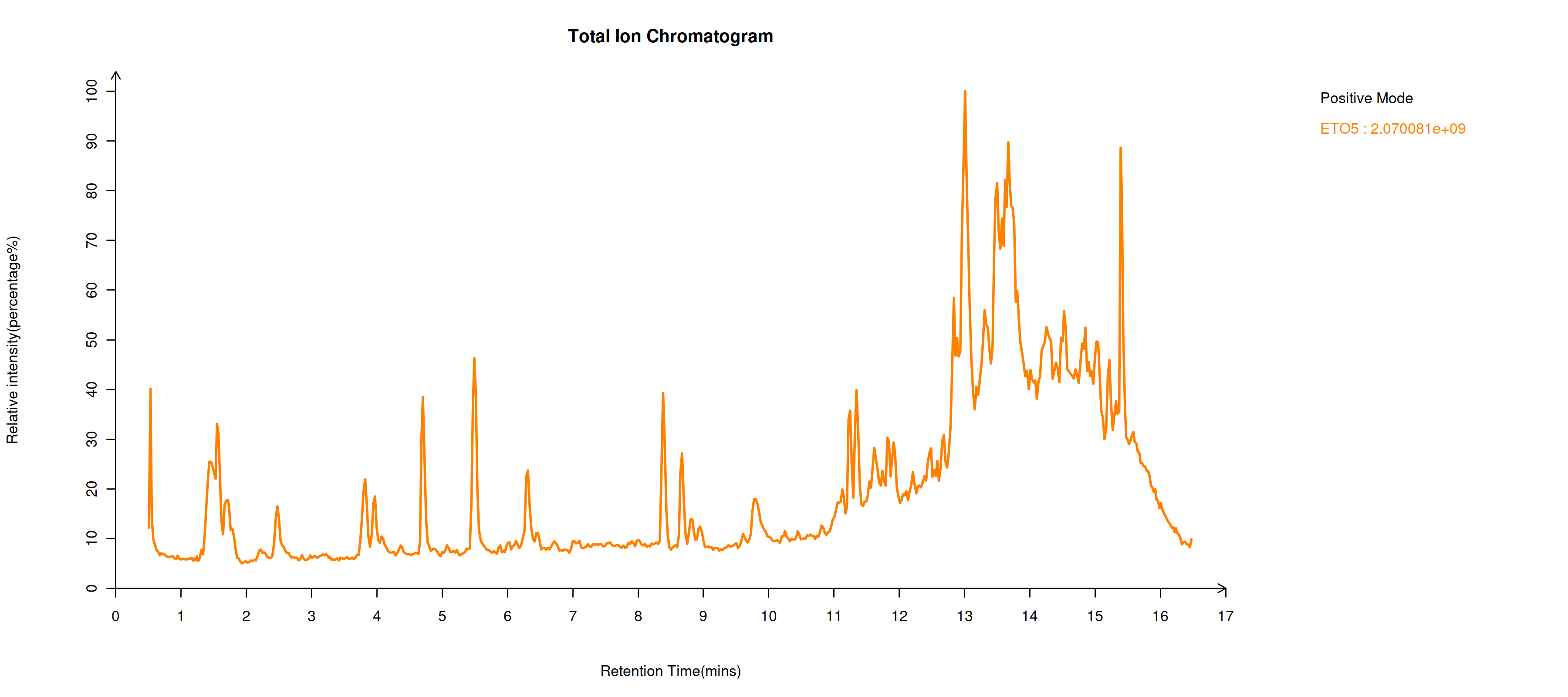

Supplement: Supplementary file 5 [file DataSheet1.ZIP › 1 TIC diagrams of all samples/Positive mode/ETO5.png]

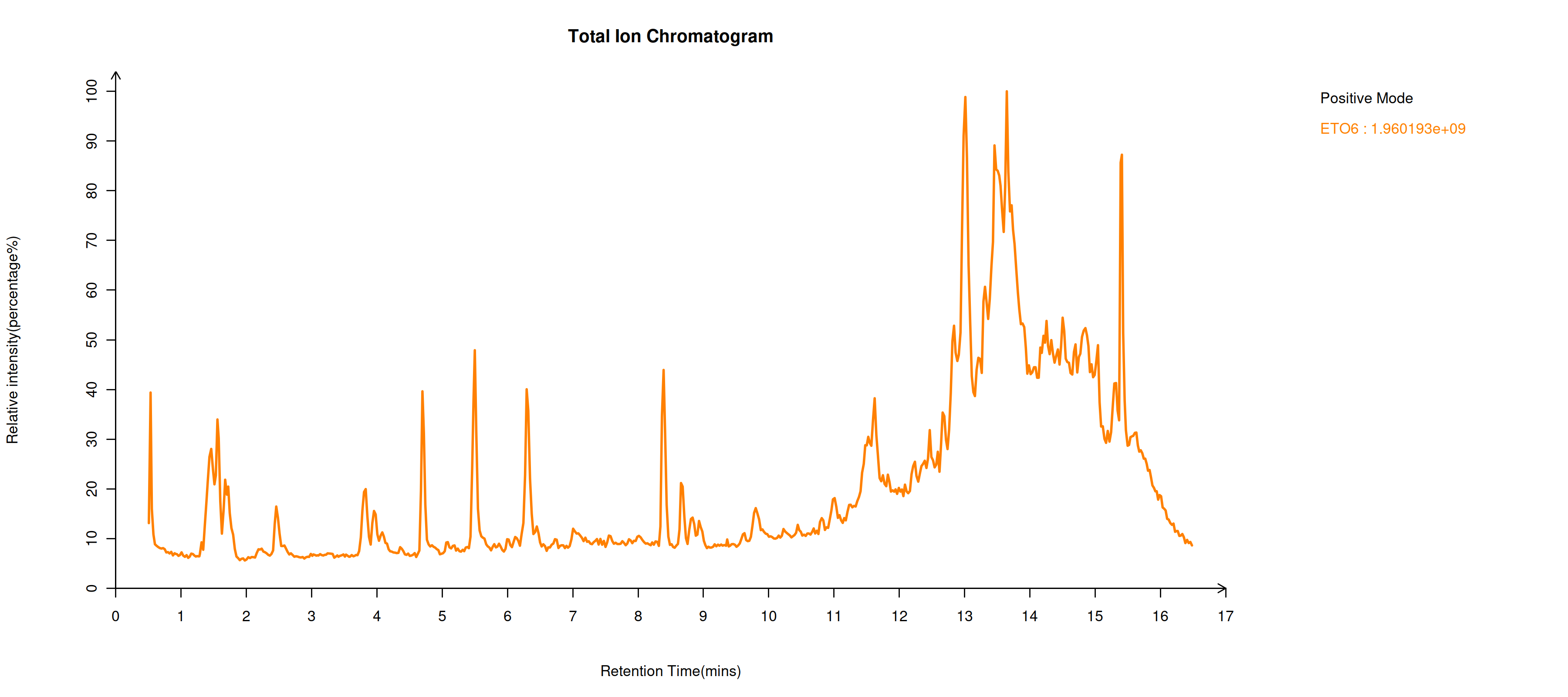

Supplement: Supplementary file 5 [file DataSheet1.ZIP › 1 TIC diagrams of all samples/Positive mode/ETO6.png]

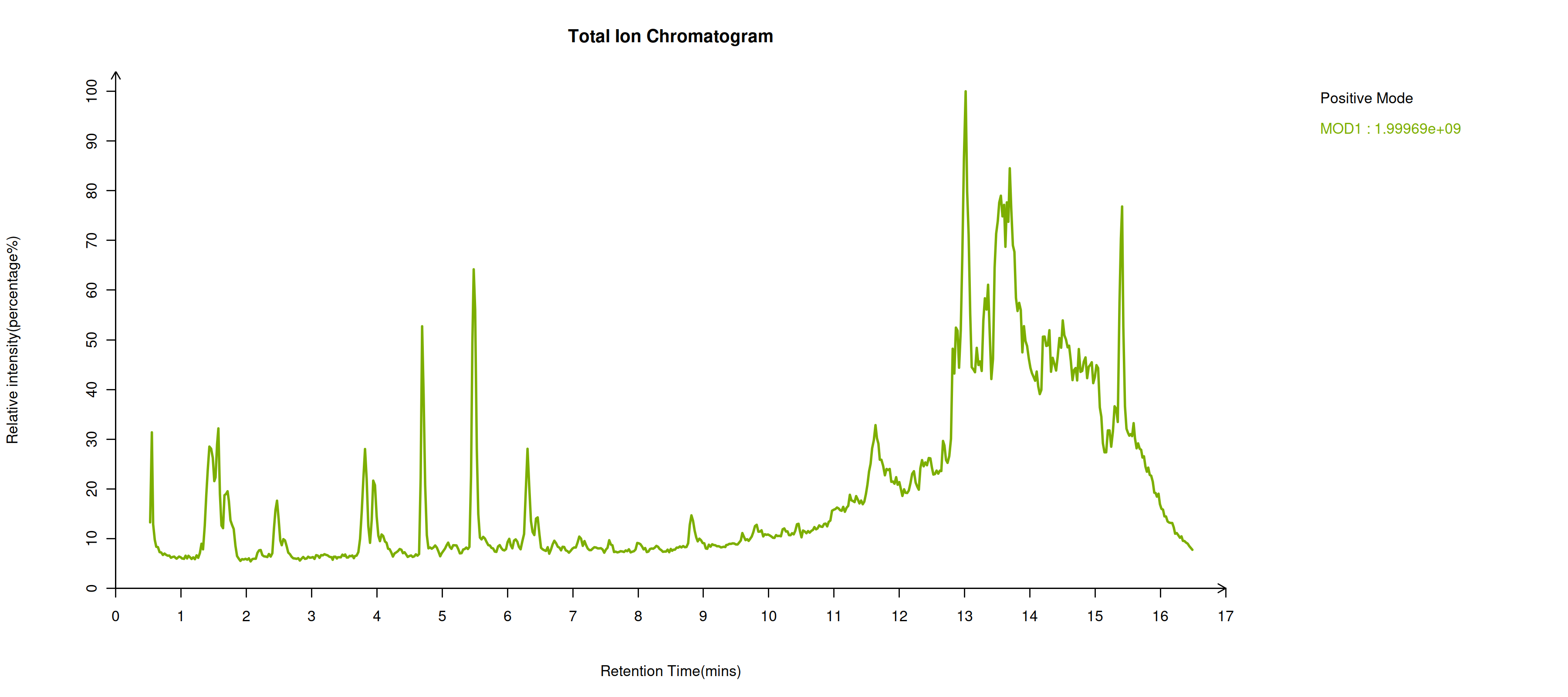

Supplement: Supplementary file 5 [file DataSheet1.ZIP › 1 TIC diagrams of all samples/Positive mode/MOD1.png]

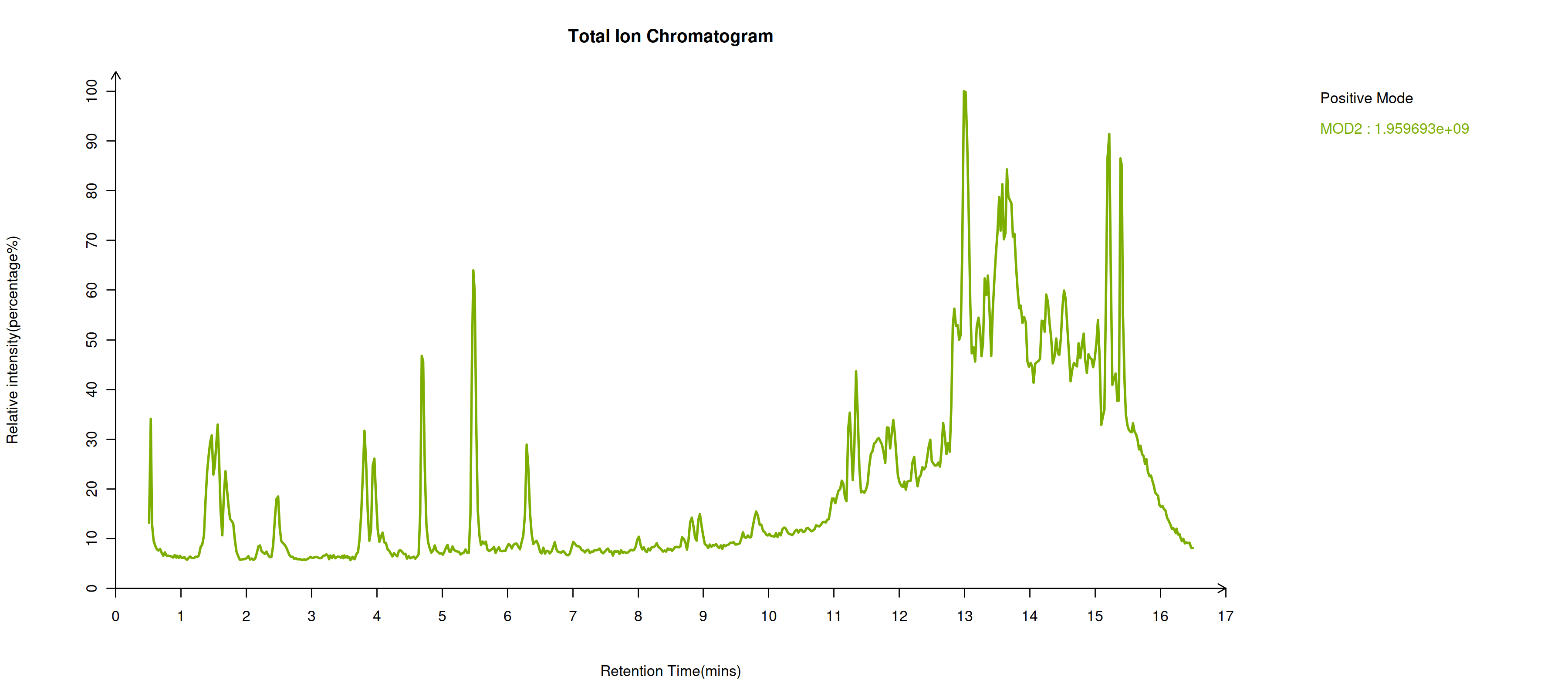

Supplement: Supplementary file 5 [file DataSheet1.ZIP › 1 TIC diagrams of all samples/Positive mode/MOD2.png]

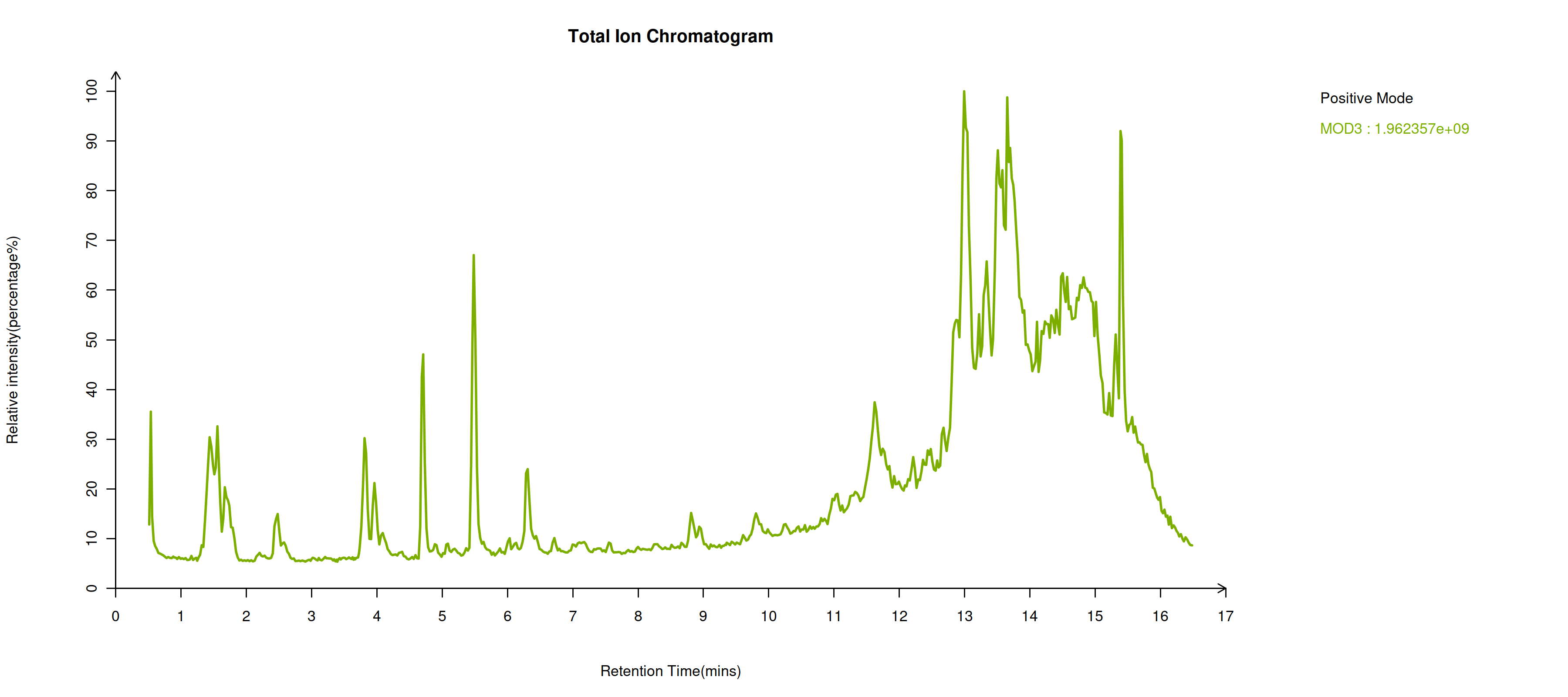

Supplement: Supplementary file 5 [file DataSheet1.ZIP › 1 TIC diagrams of all samples/Positive mode/MOD3.png]

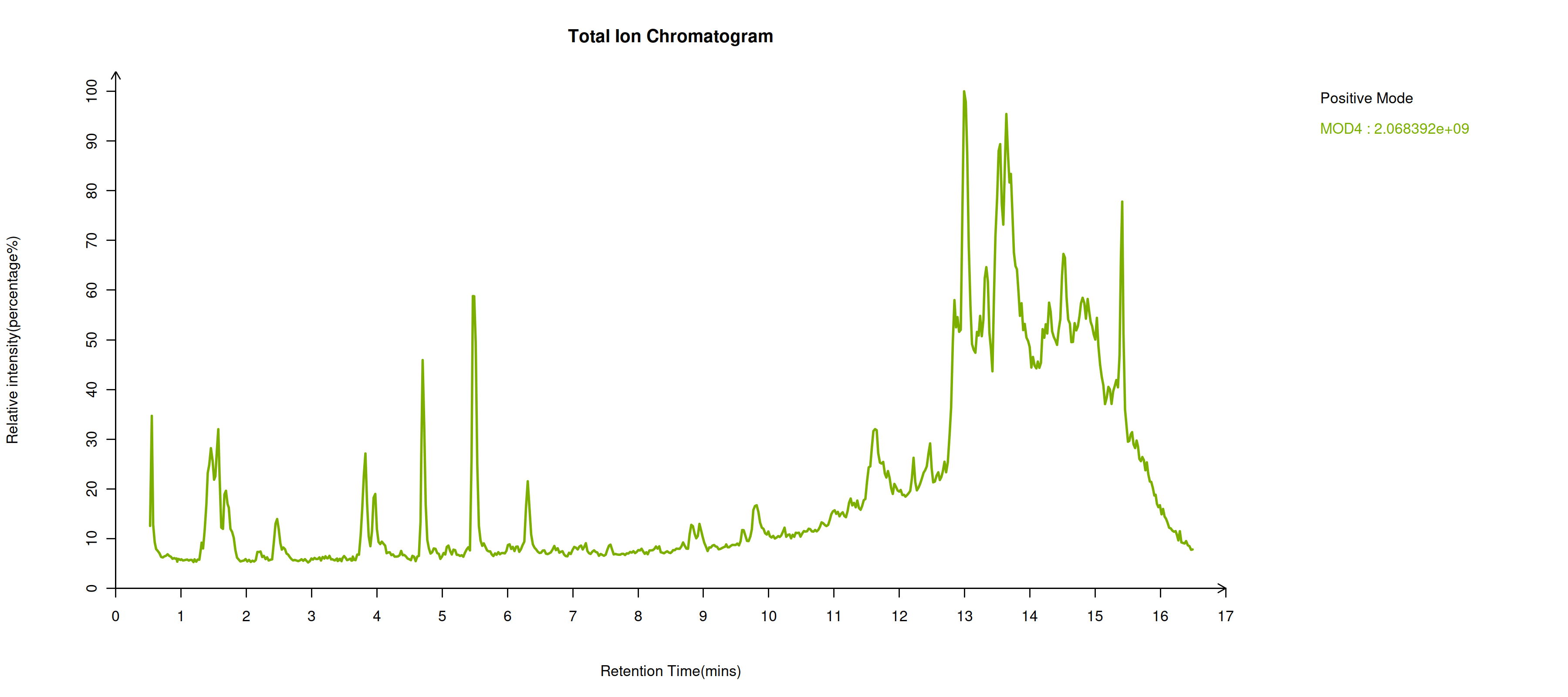

Supplement: Supplementary file 5 [file DataSheet1.ZIP › 1 TIC diagrams of all samples/Positive mode/MOD4.png]

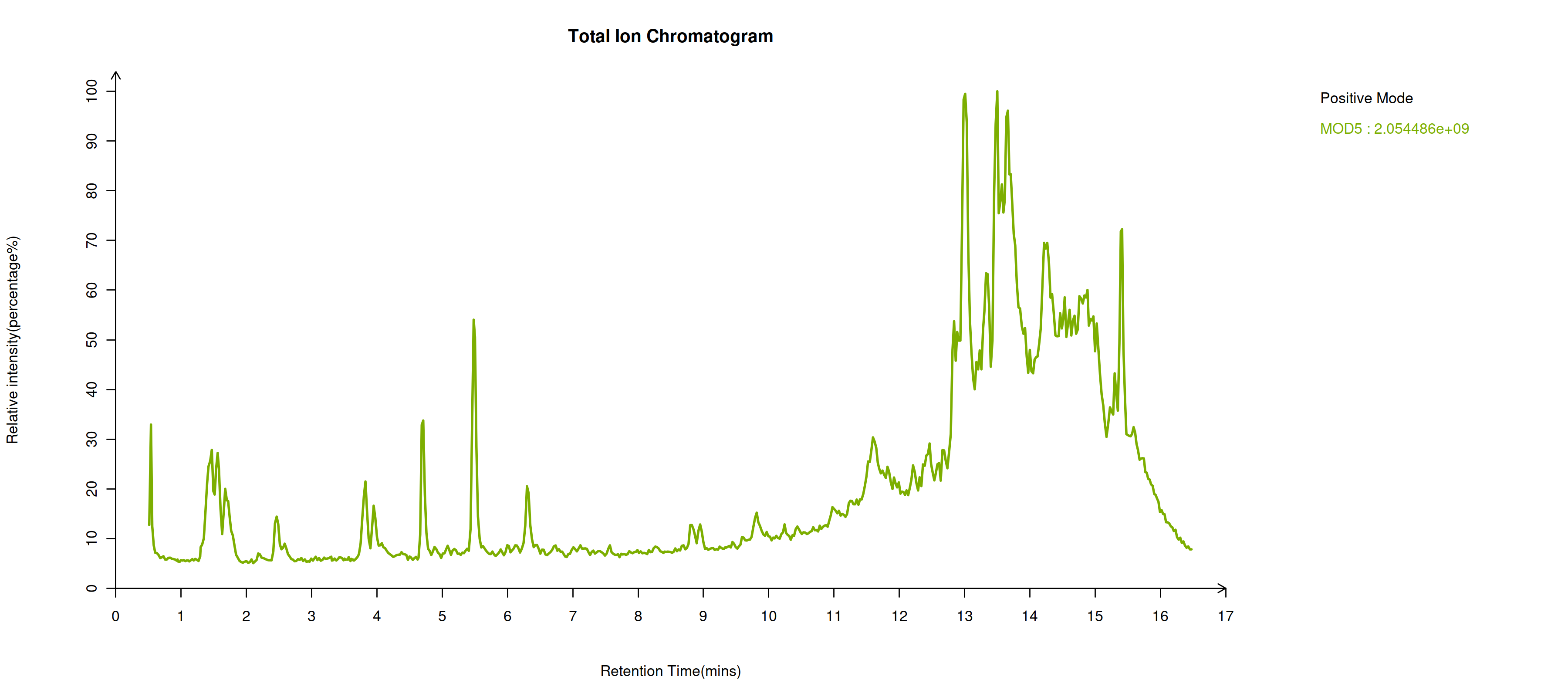

Supplement: Supplementary file 5 [file DataSheet1.ZIP › 1 TIC diagrams of all samples/Positive mode/MOD5.png]

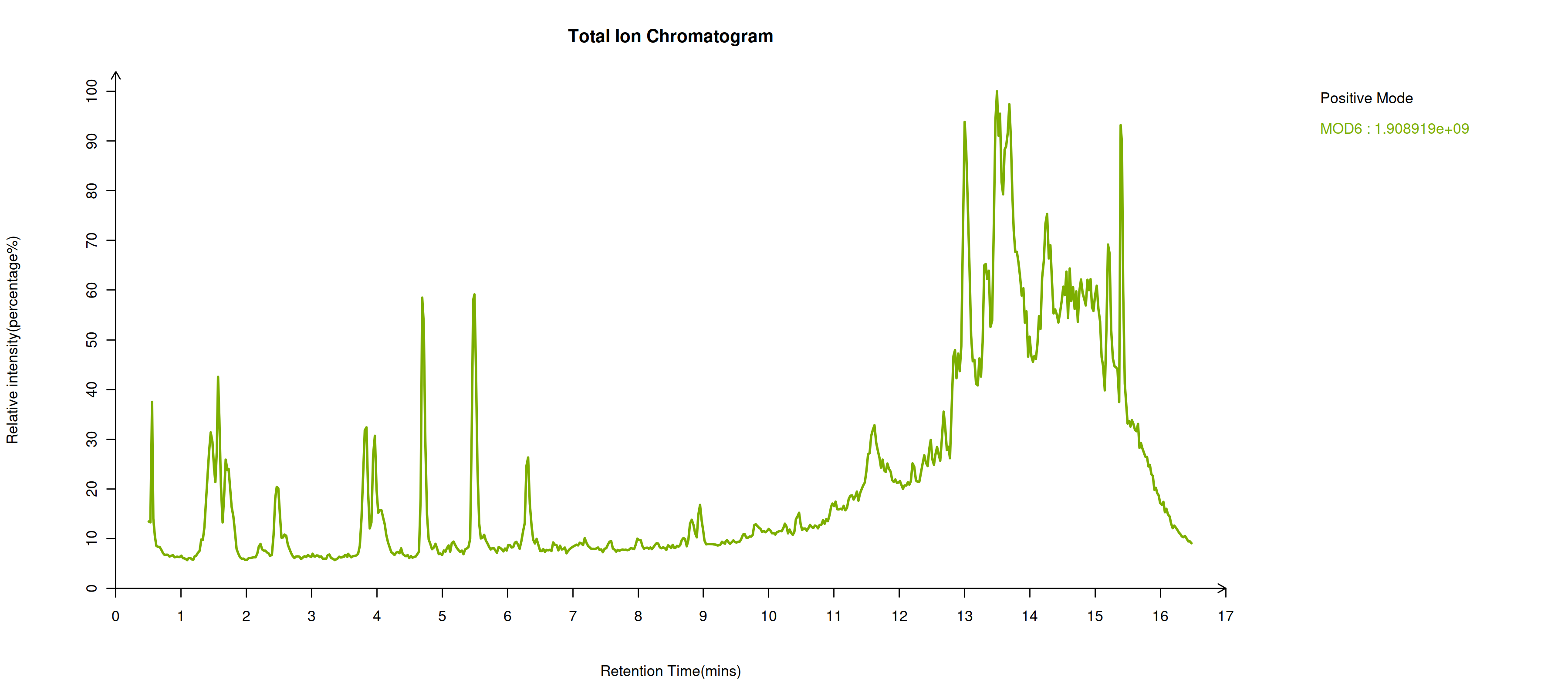

Supplement: Supplementary file 5 [file DataSheet1.ZIP › 1 TIC diagrams of all samples/Positive mode/MOD6.png]

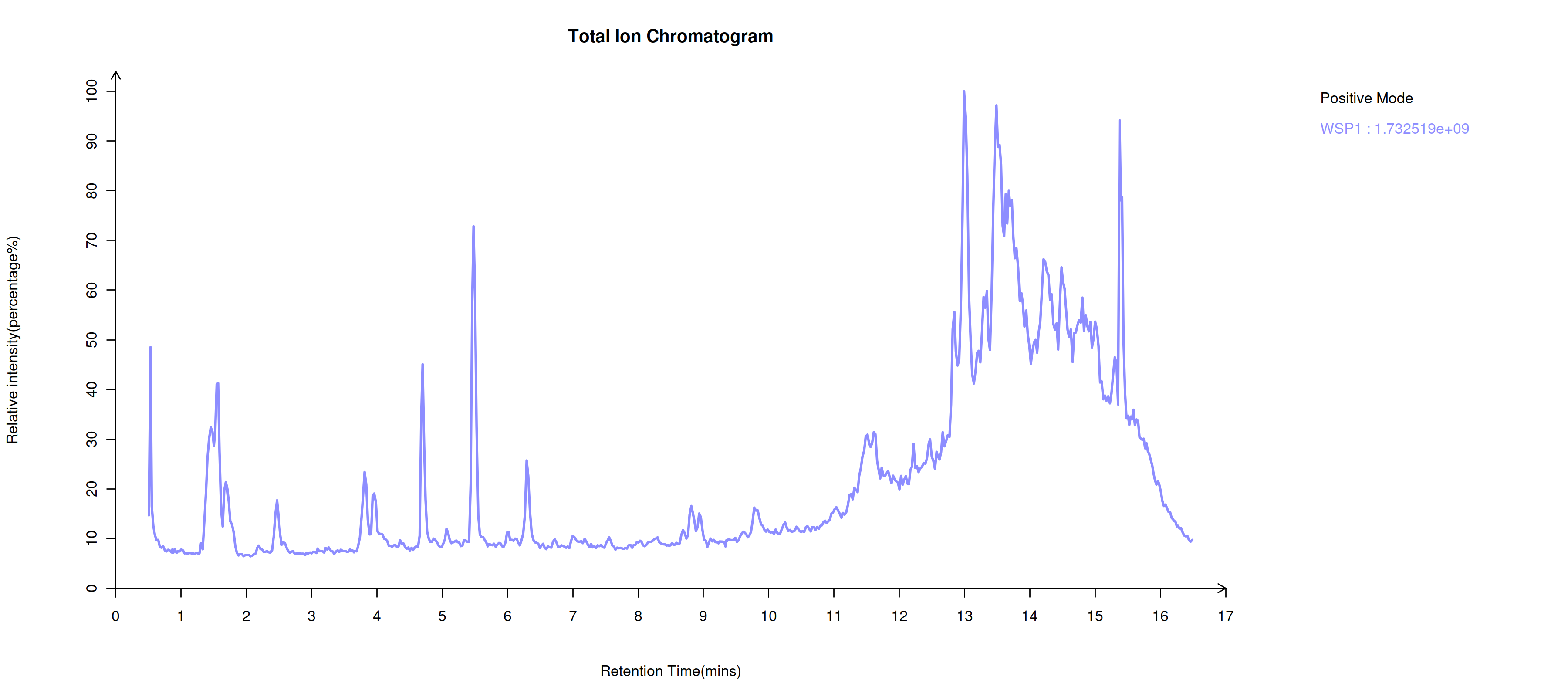

Supplement: Supplementary file 5 [file DataSheet1.ZIP › 1 TIC diagrams of all samples/Positive mode/WSP1.png]

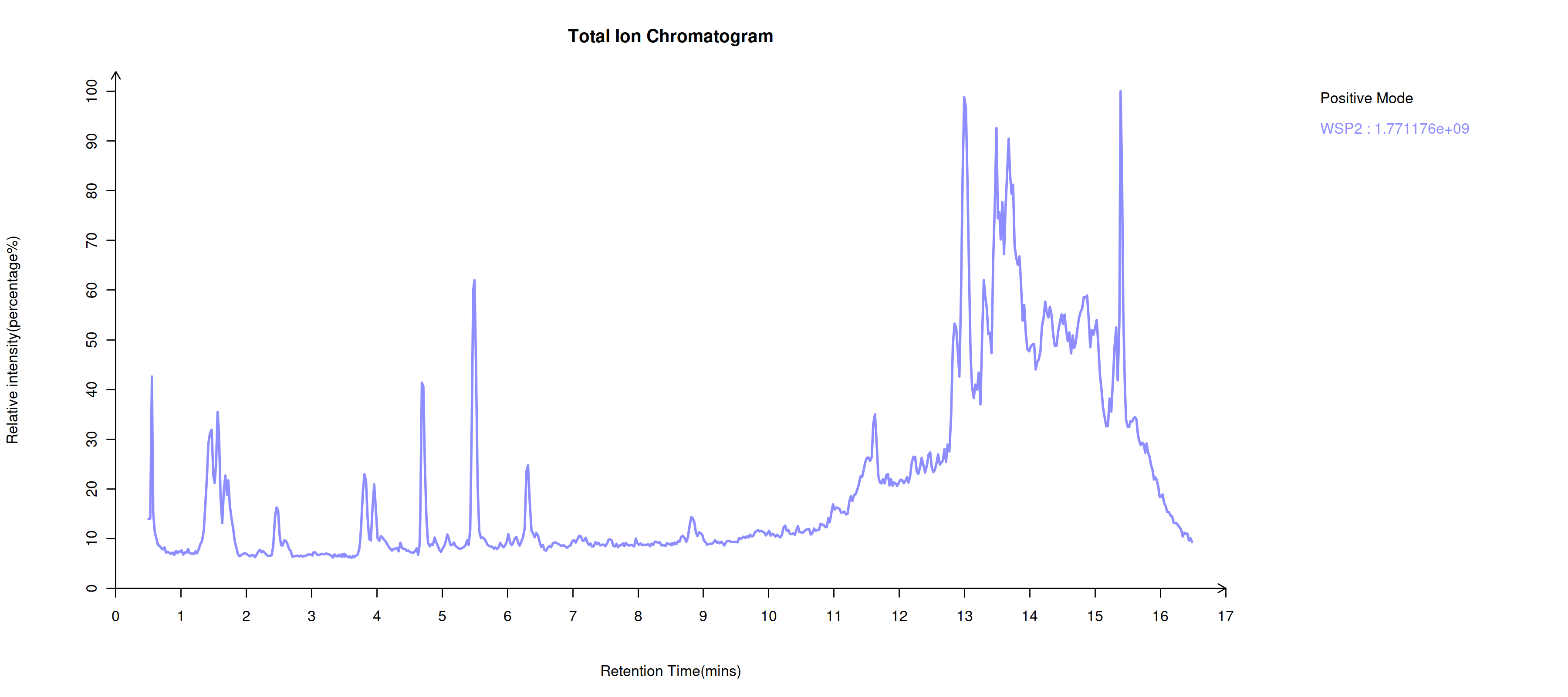

Supplement: Supplementary file 5 [file DataSheet1.ZIP › 1 TIC diagrams of all samples/Positive mode/WSP2.png]

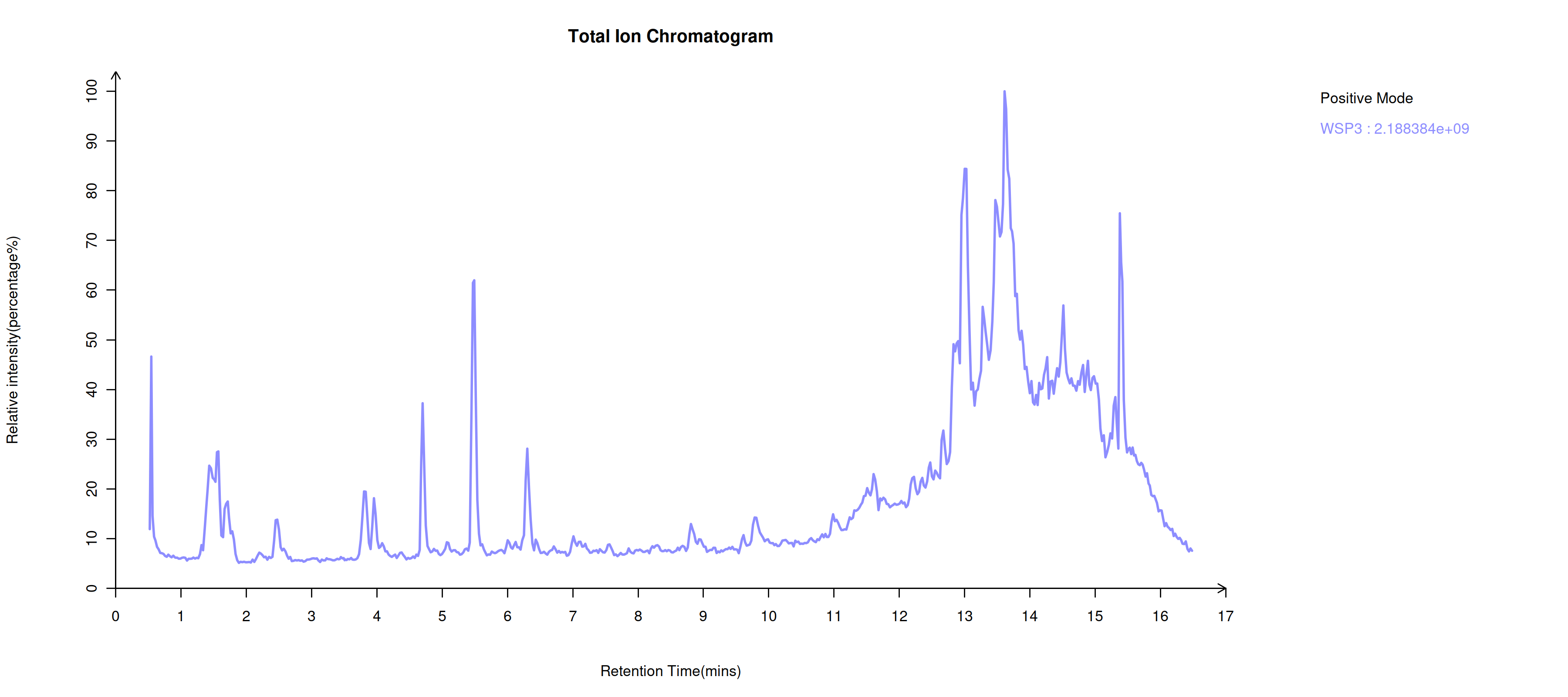

Supplement: Supplementary file 5 [file DataSheet1.ZIP › 1 TIC diagrams of all samples/Positive mode/WSP3.png]

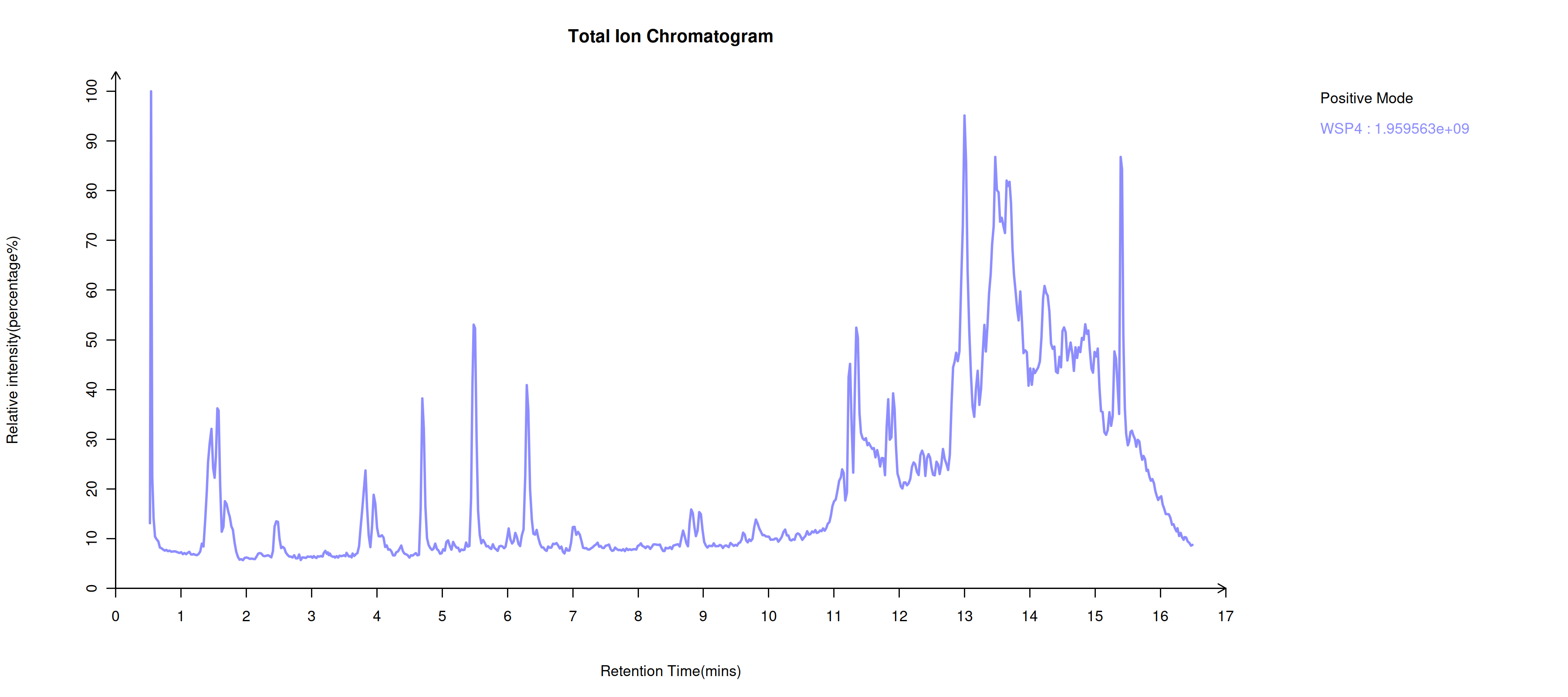

Supplement: Supplementary file 5 [file DataSheet1.ZIP › 1 TIC diagrams of all samples/Positive mode/WSP4.png]

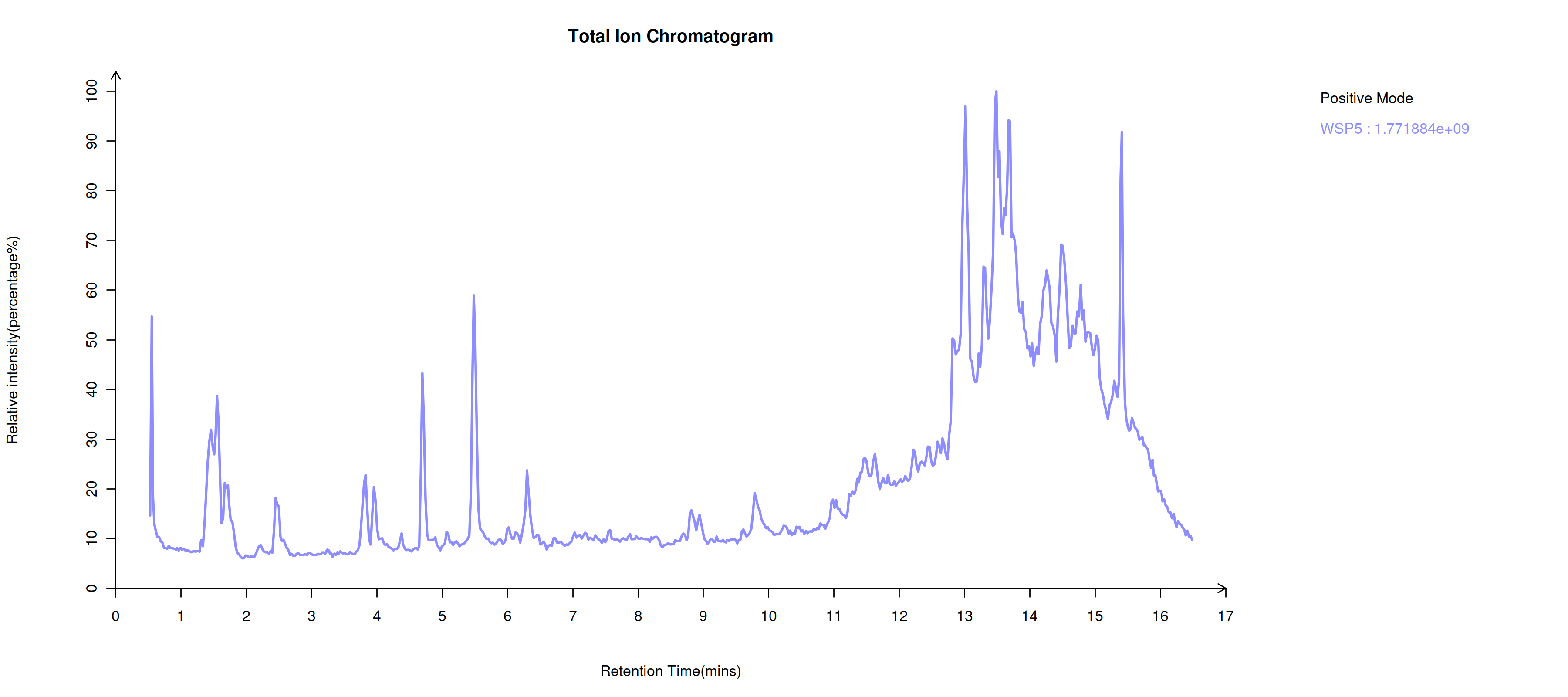

Supplement: Supplementary file 5 [file DataSheet1.ZIP › 1 TIC diagrams of all samples/Positive mode/WSP5.png]

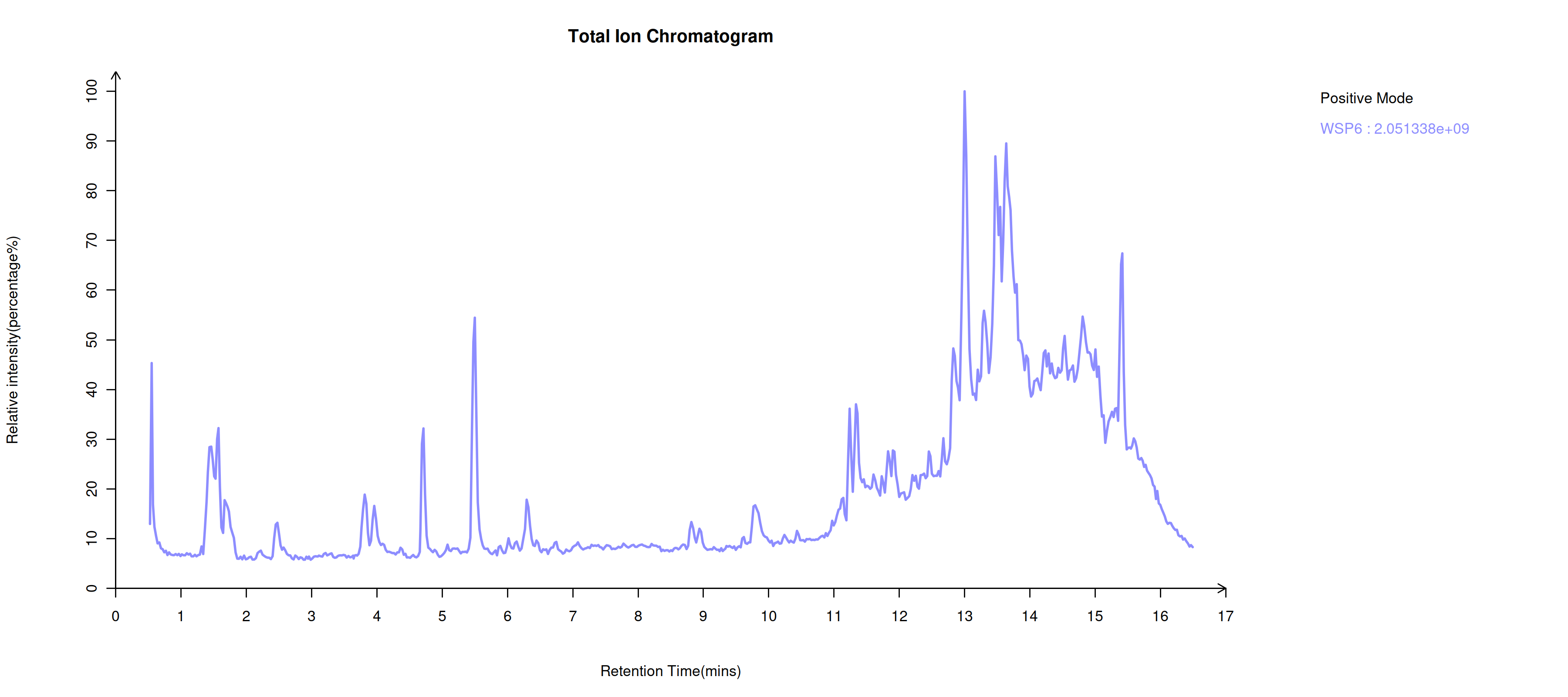

Supplement: Supplementary file 5 [file DataSheet1.ZIP › 1 TIC diagrams of all samples/Positive mode/WSP6.png]

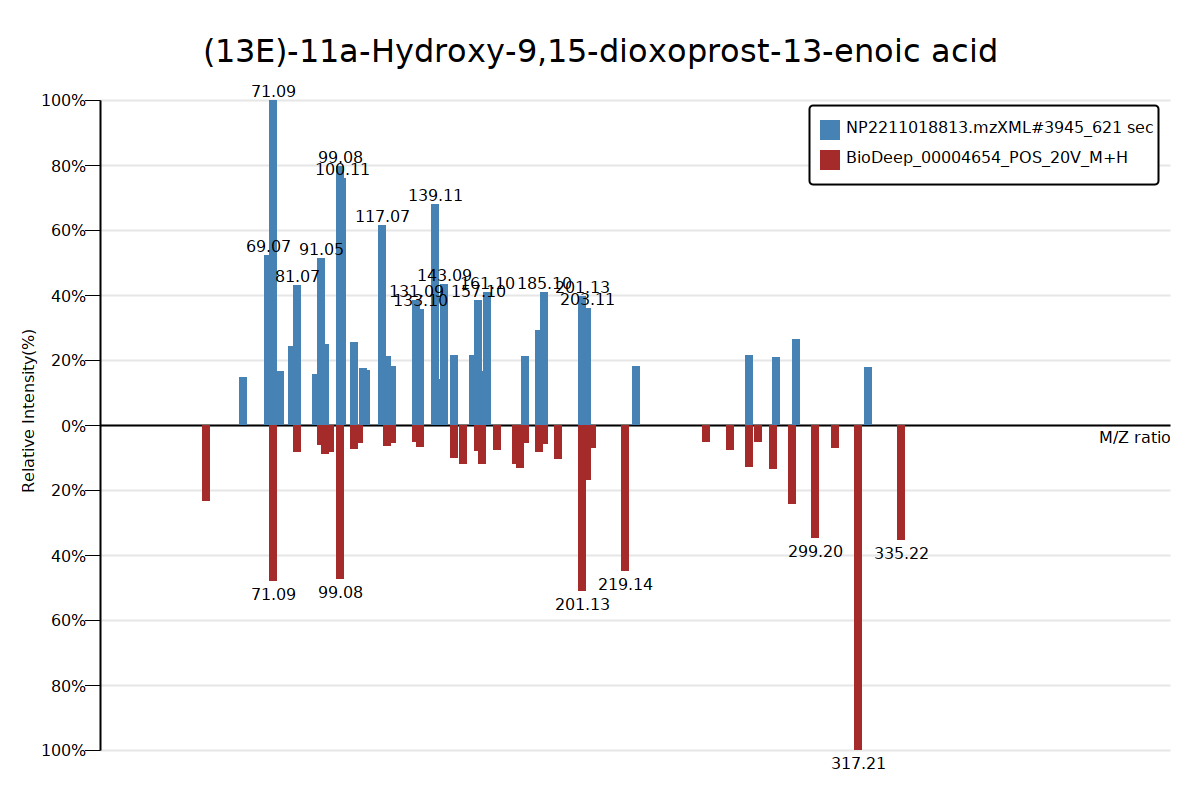

Supplement: Supplementary file 5 [file DataSheet1.ZIP › 2 result graphs between the MSMS secondary fragments of each metabolite and the MSMS secondary fragments of the standard substance in the database/(13E)-11a-Hydroxy-9,15-dioxoprost-13-enoic acid.png]

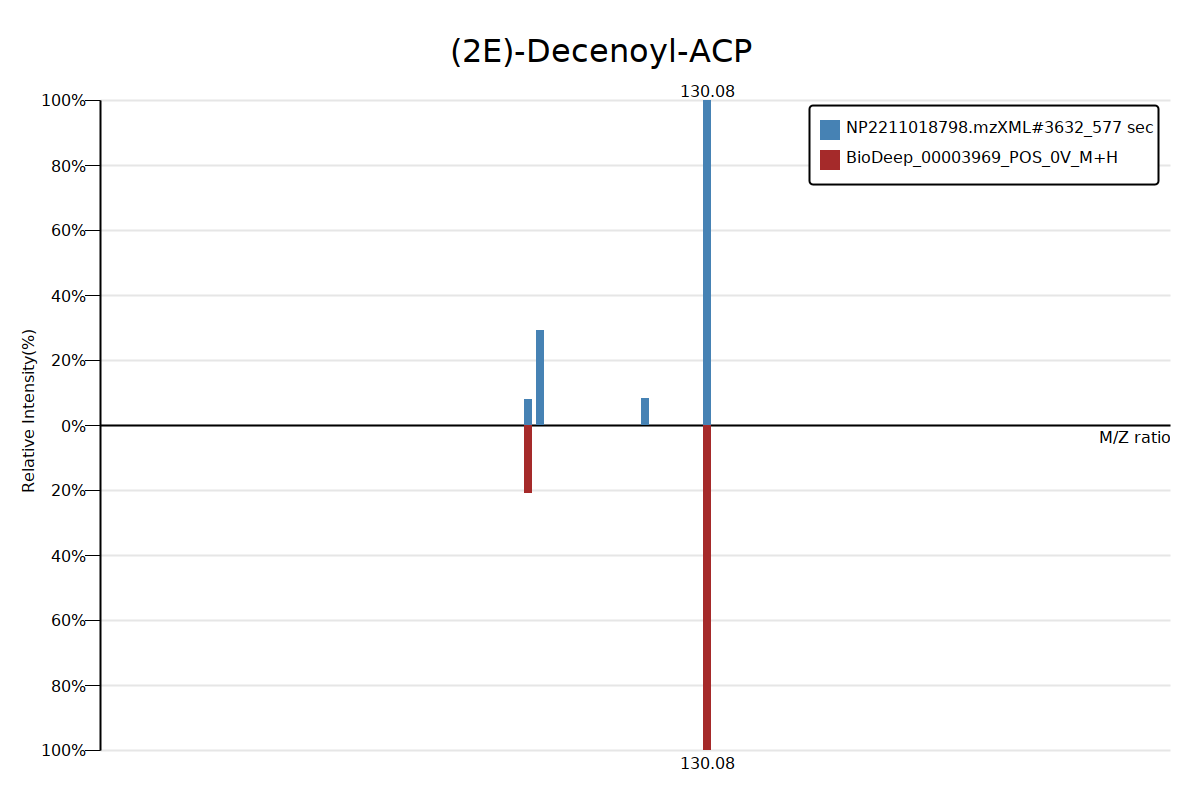

Supplement: Supplementary file 5 [file DataSheet1.ZIP › 2 result graphs between the MSMS secondary fragments of each metabolite and the MSMS secondary fragments of the standard substance in the database/(2E)-Decenoyl-ACP.png]

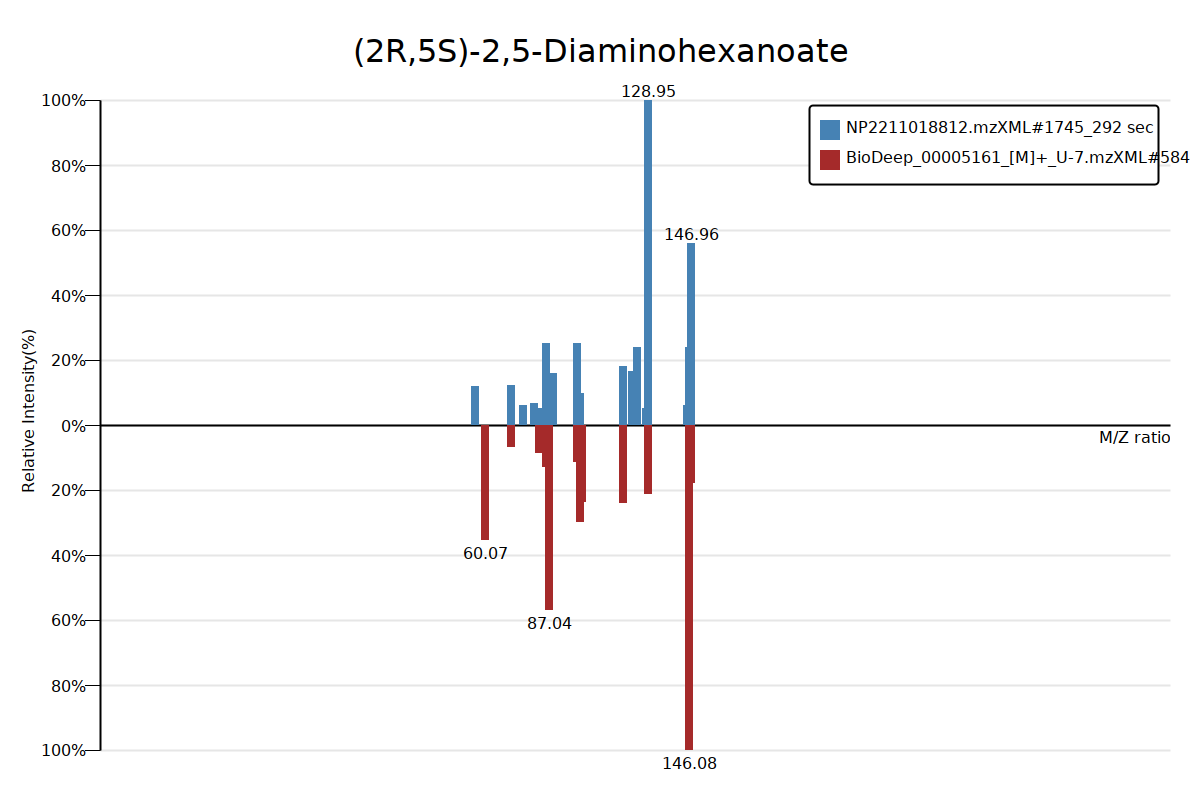

Supplement: Supplementary file 5 [file DataSheet1.ZIP › 2 result graphs between the MSMS secondary fragments of each metabolite and the MSMS secondary fragments of the standard substance in the database/(2R,5S)-2,5-Diaminohexanoate.png]

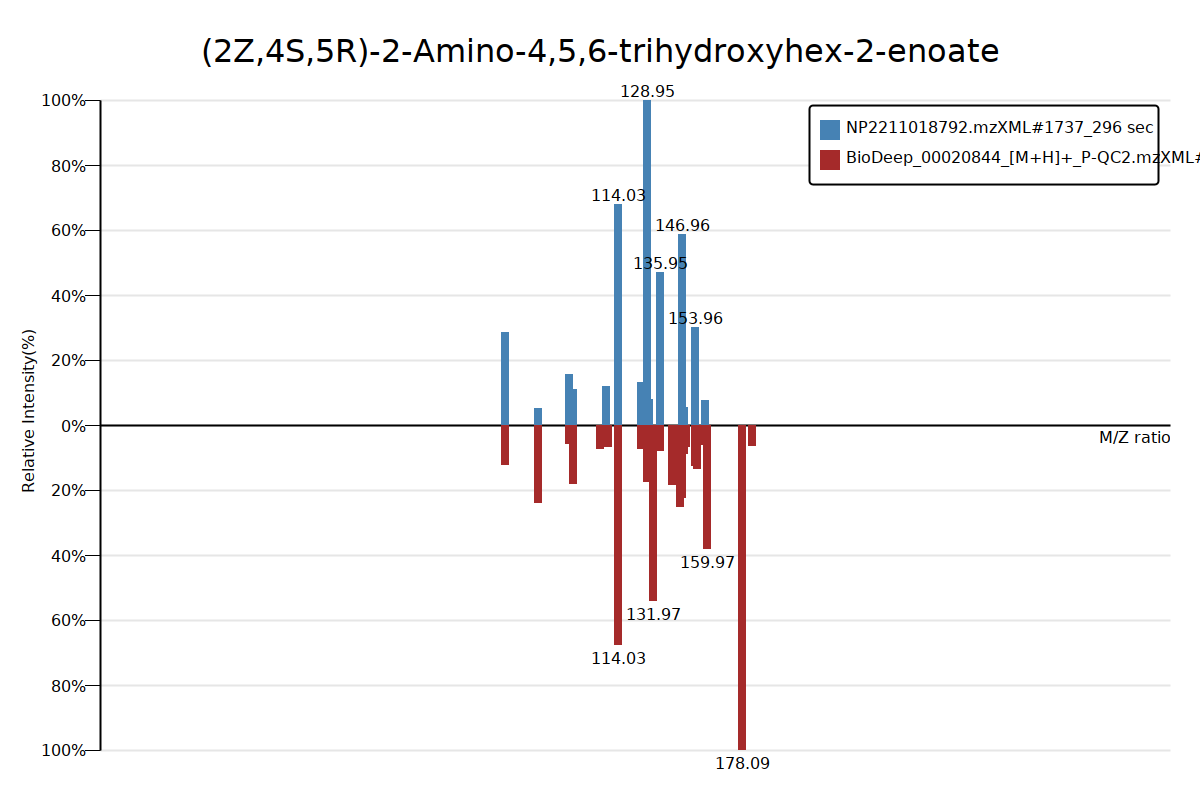

Supplement: Supplementary file 5 [file DataSheet1.ZIP › 2 result graphs between the MSMS secondary fragments of each metabolite and the MSMS secondary fragments of the standard substance in the database/(2Z,4S,5R)-2-Amino-4,5,6-trihydroxyhex-2-enoate.png]

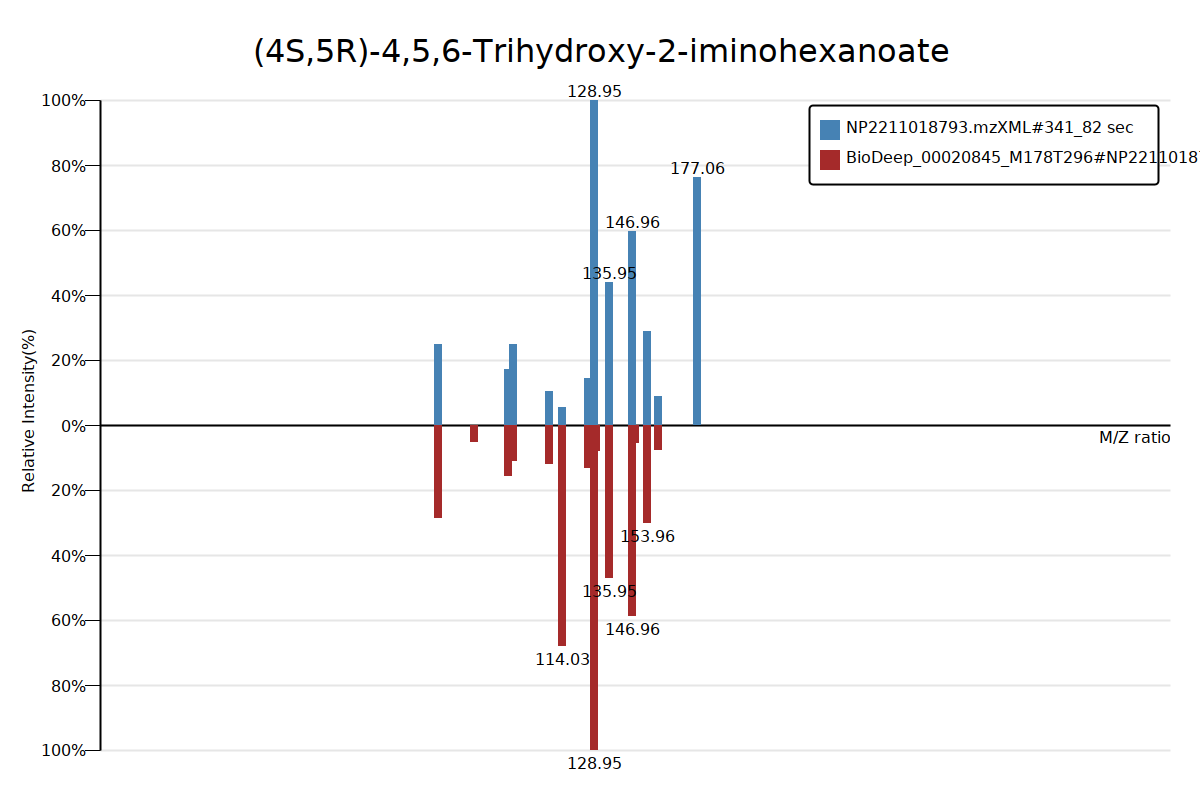

Supplement: Supplementary file 5 [file DataSheet1.ZIP › 2 result graphs between the MSMS secondary fragments of each metabolite and the MSMS secondary fragments of the standard substance in the database/(4S,5R)-4,5,6-Trihydroxy-2-iminohexanoate.png]

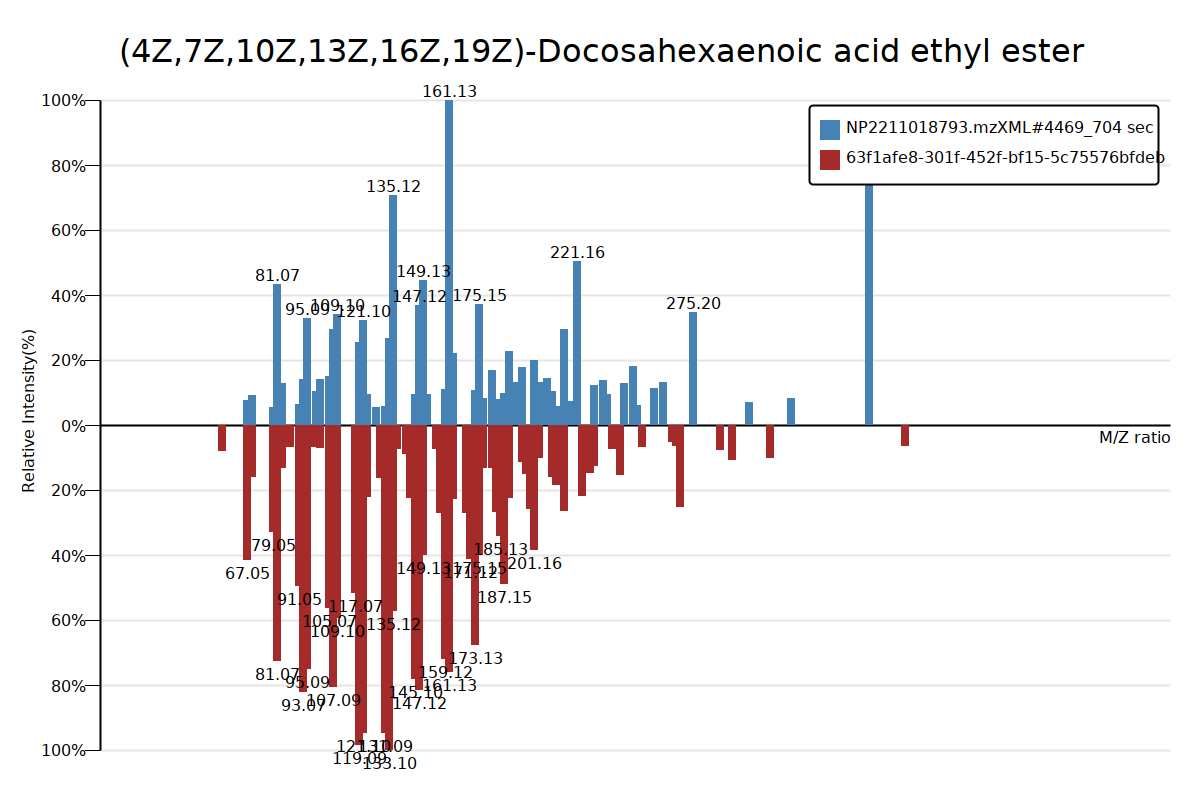

Supplement: Supplementary file 5 [file DataSheet1.ZIP › 2 result graphs between the MSMS secondary fragments of each metabolite and the MSMS secondary fragments of the standard substance in the database/(4Z,7Z,10Z,13Z,16Z,19Z)-Docosahexaenoic acid ethyl ester.png]

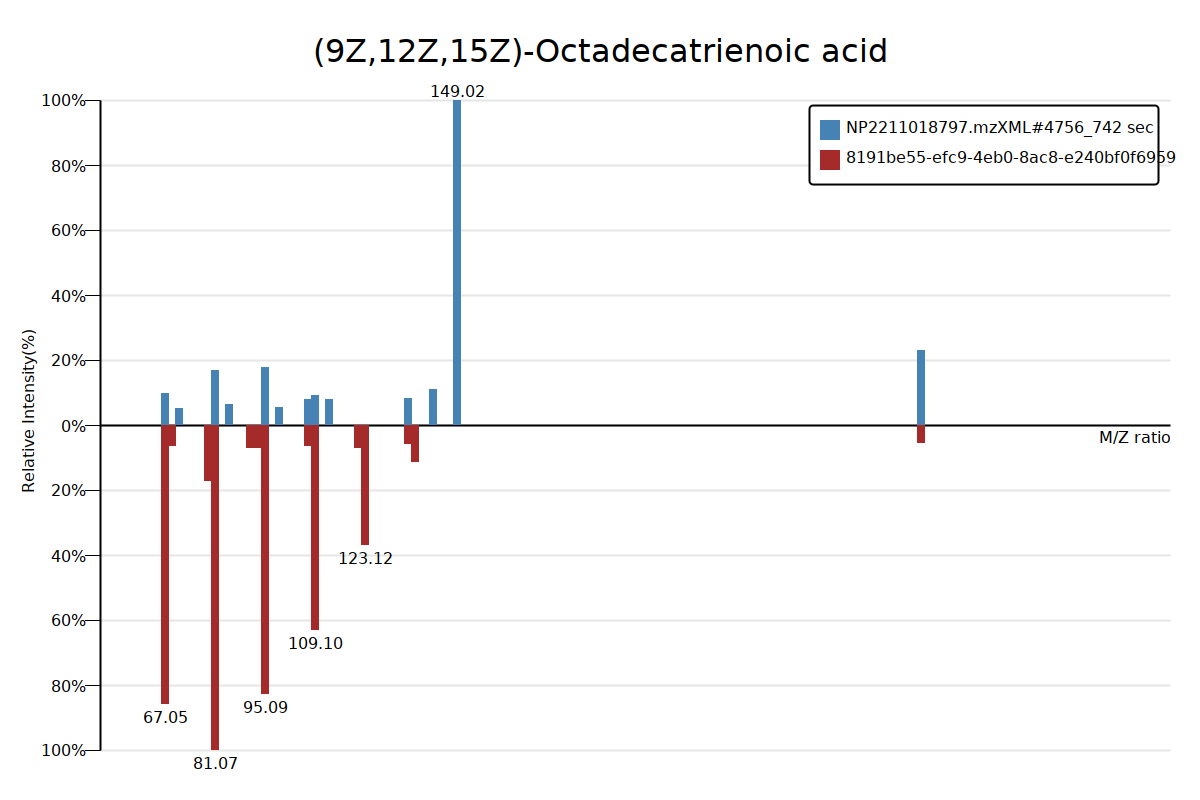

Supplement: Supplementary file 5 [file DataSheet1.ZIP › 2 result graphs between the MSMS secondary fragments of each metabolite and the MSMS secondary fragments of the standard substance in the database/(9Z,12Z,15Z)-Octadecatrienoic acid.png]

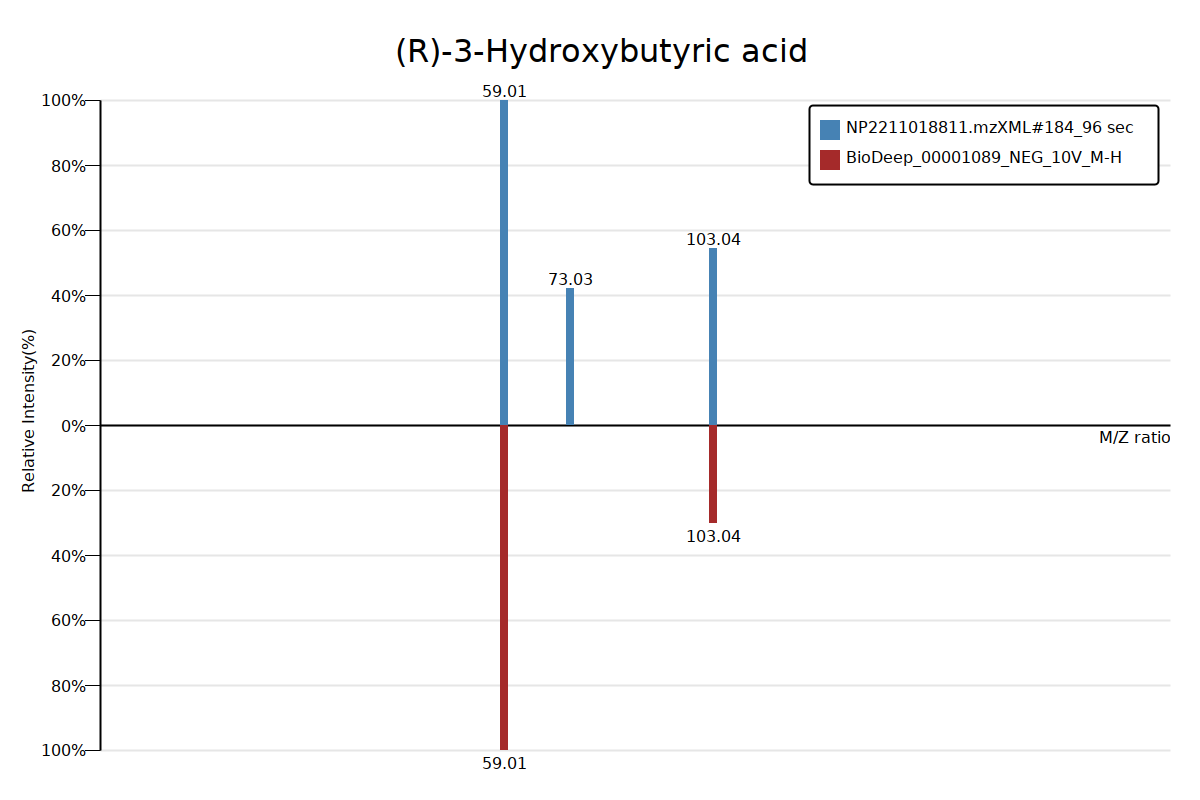

Supplement: Supplementary file 5 [file DataSheet1.ZIP › 2 result graphs between the MSMS secondary fragments of each metabolite and the MSMS secondary fragments of the standard substance in the database/(R)-3-Hydroxybutyric acid.png]

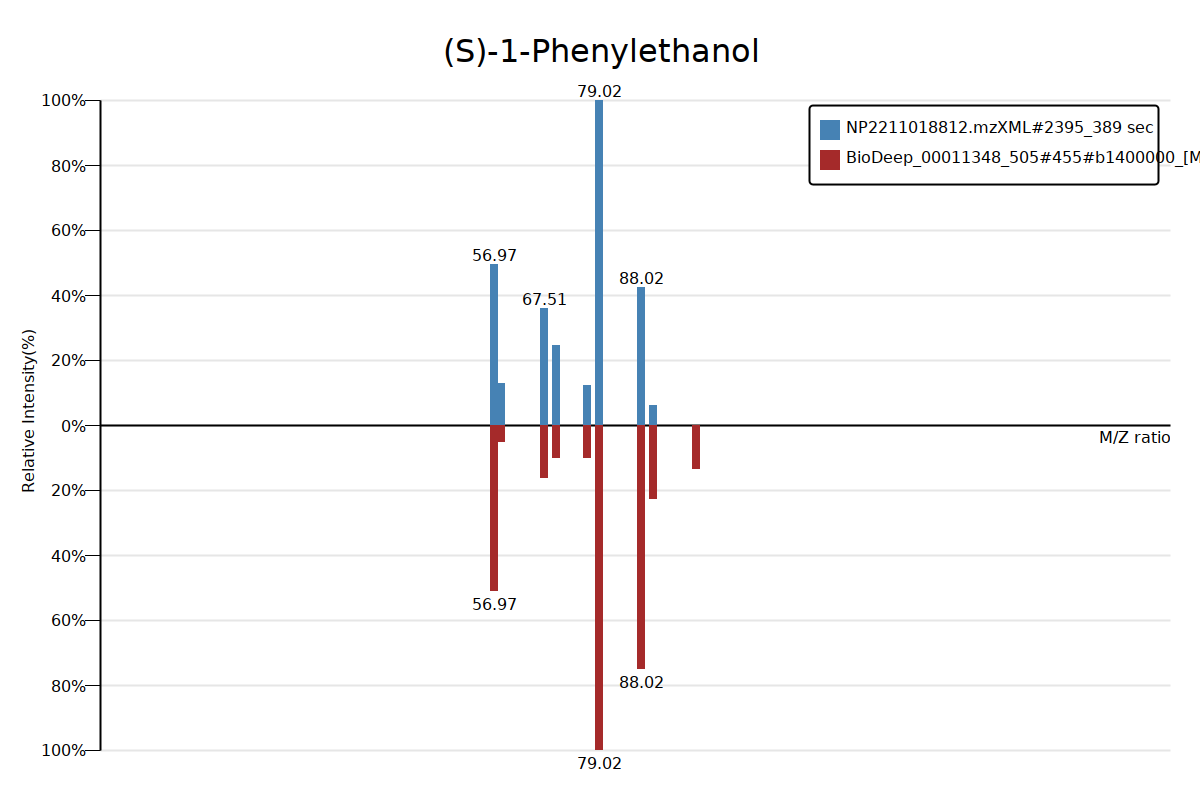

Supplement: Supplementary file 5 [file DataSheet1.ZIP › 2 result graphs between the MSMS secondary fragments of each metabolite and the MSMS secondary fragments of the standard substance in the database/(S)-1-Phenylethanol.png]

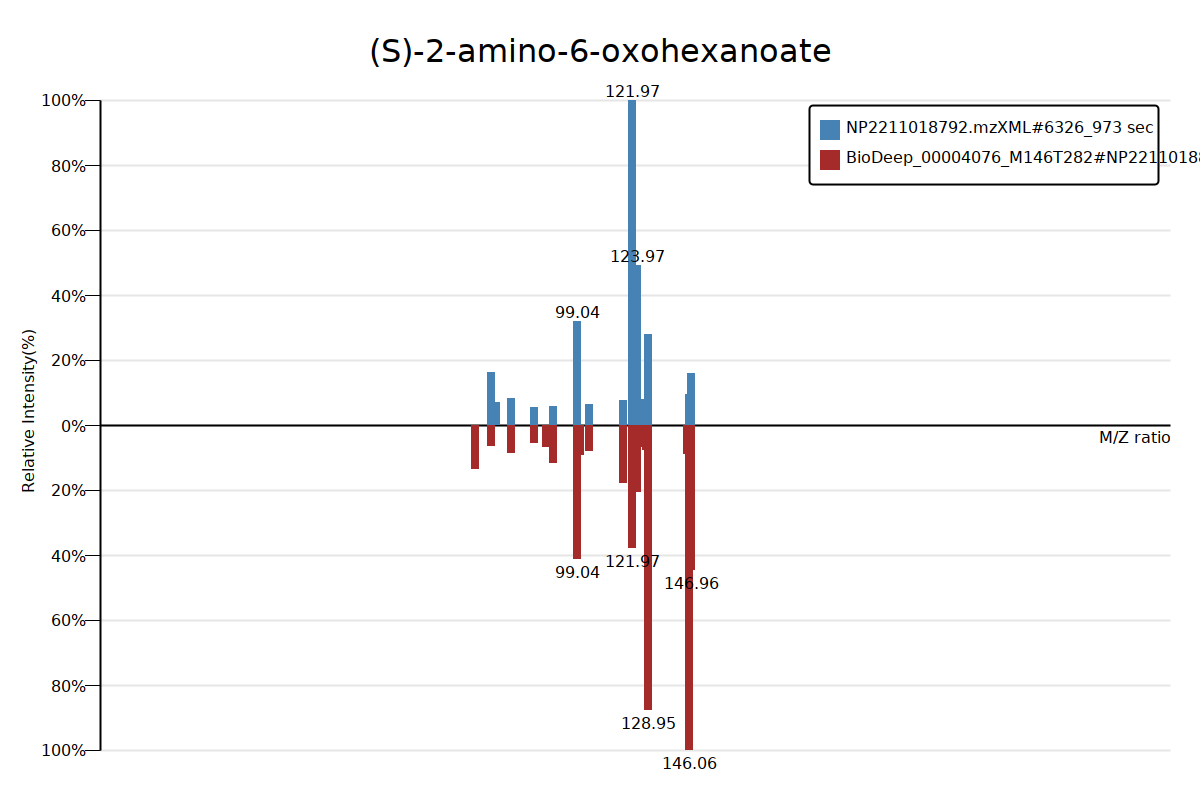

Supplement: Supplementary file 5 [file DataSheet1.ZIP › 2 result graphs between the MSMS secondary fragments of each metabolite and the MSMS secondary fragments of the standard substance in the database/(S)-2-amino-6-oxohexanoate.png]

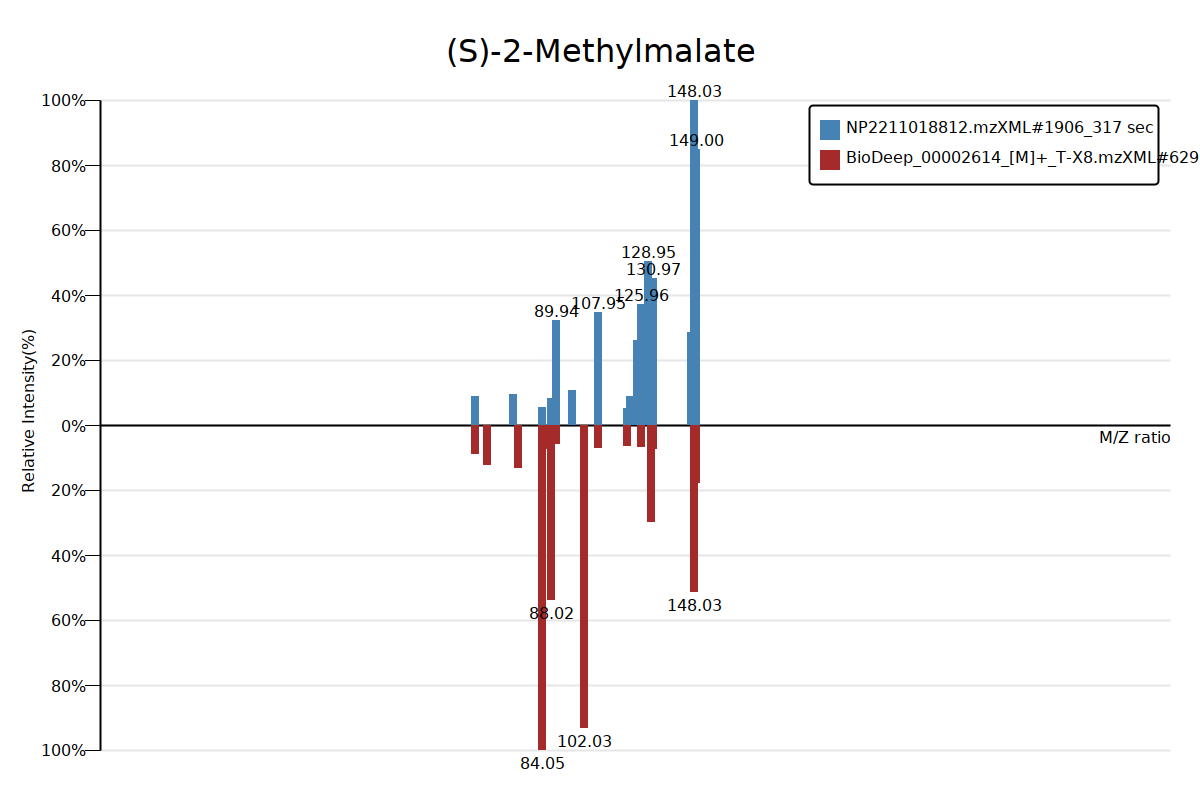

Supplement: Supplementary file 5 [file DataSheet1.ZIP › 2 result graphs between the MSMS secondary fragments of each metabolite and the MSMS secondary fragments of the standard substance in the database/(S)-2-Methylmalate.png]

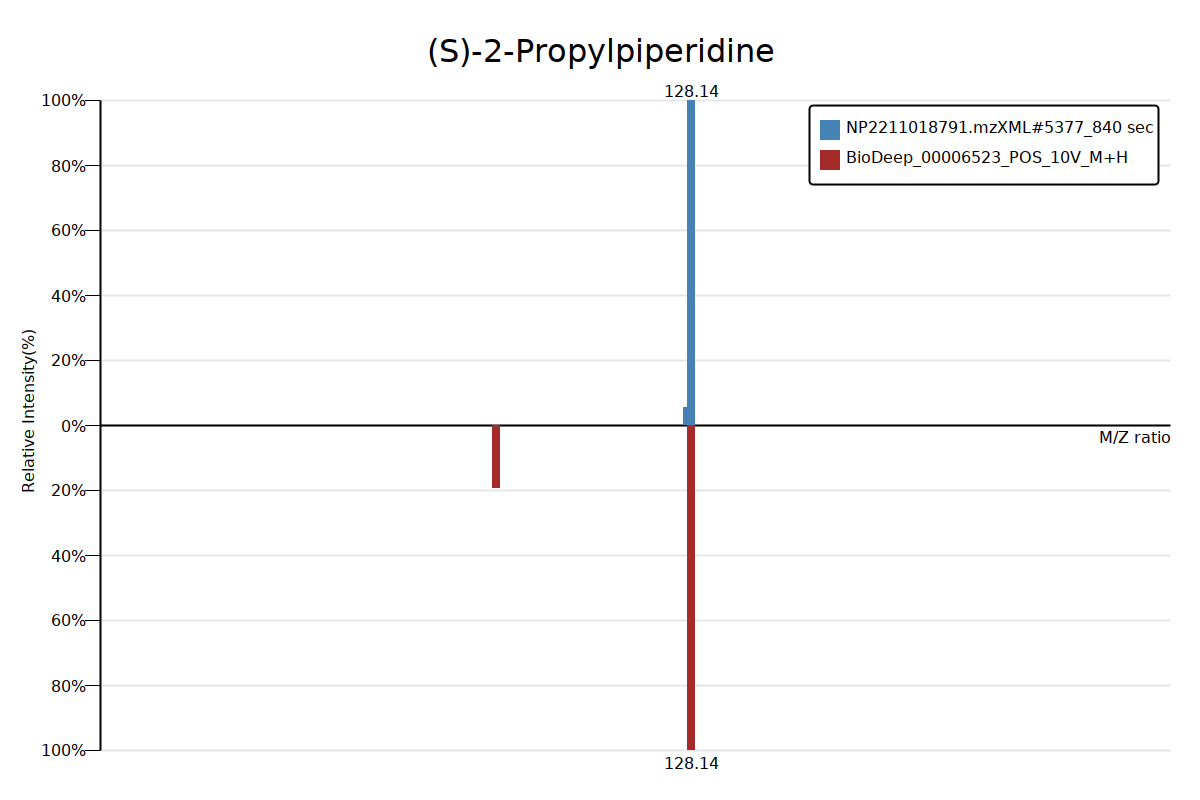

Supplement: Supplementary file 5 [file DataSheet1.ZIP › 2 result graphs between the MSMS secondary fragments of each metabolite and the MSMS secondary fragments of the standard substance in the database/(S)-2-Propylpiperidine.png]

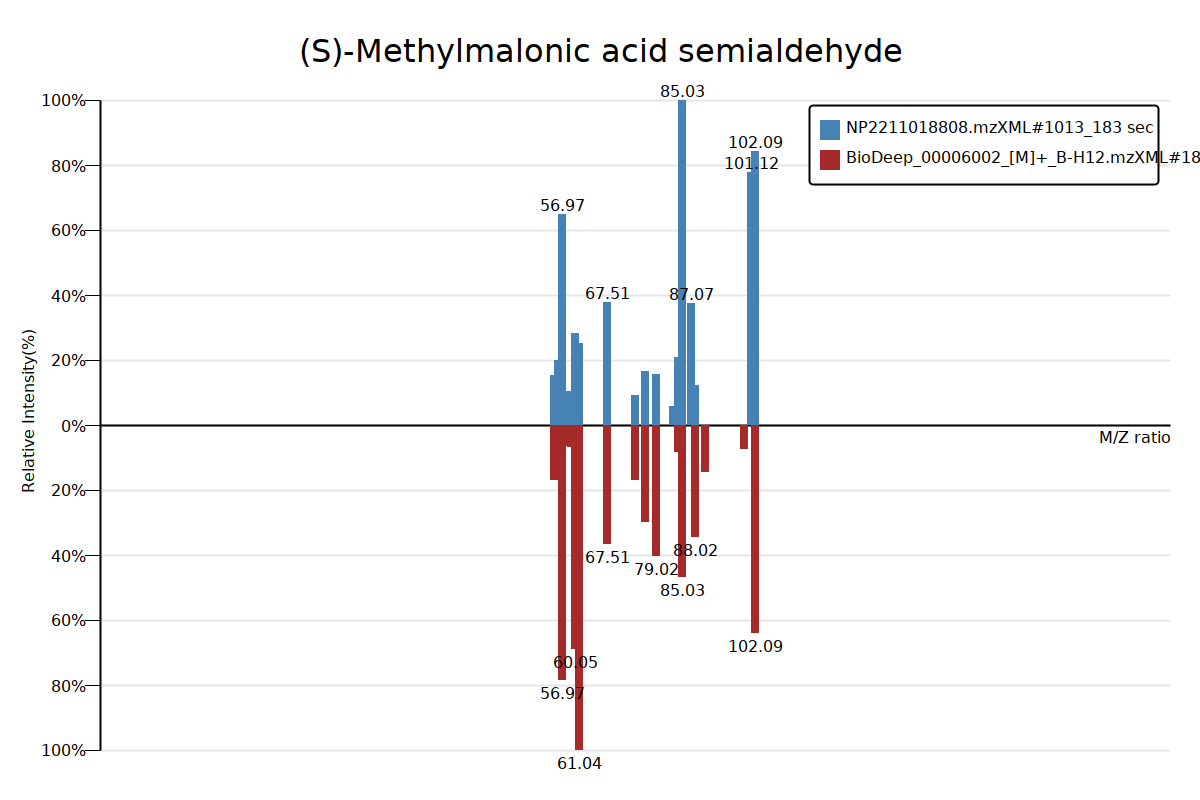

Supplement: Supplementary file 5 [file DataSheet1.ZIP › 2 result graphs between the MSMS secondary fragments of each metabolite and the MSMS secondary fragments of the standard substance in the database/(S)-Methylmalonic acid semialdehyde.png]

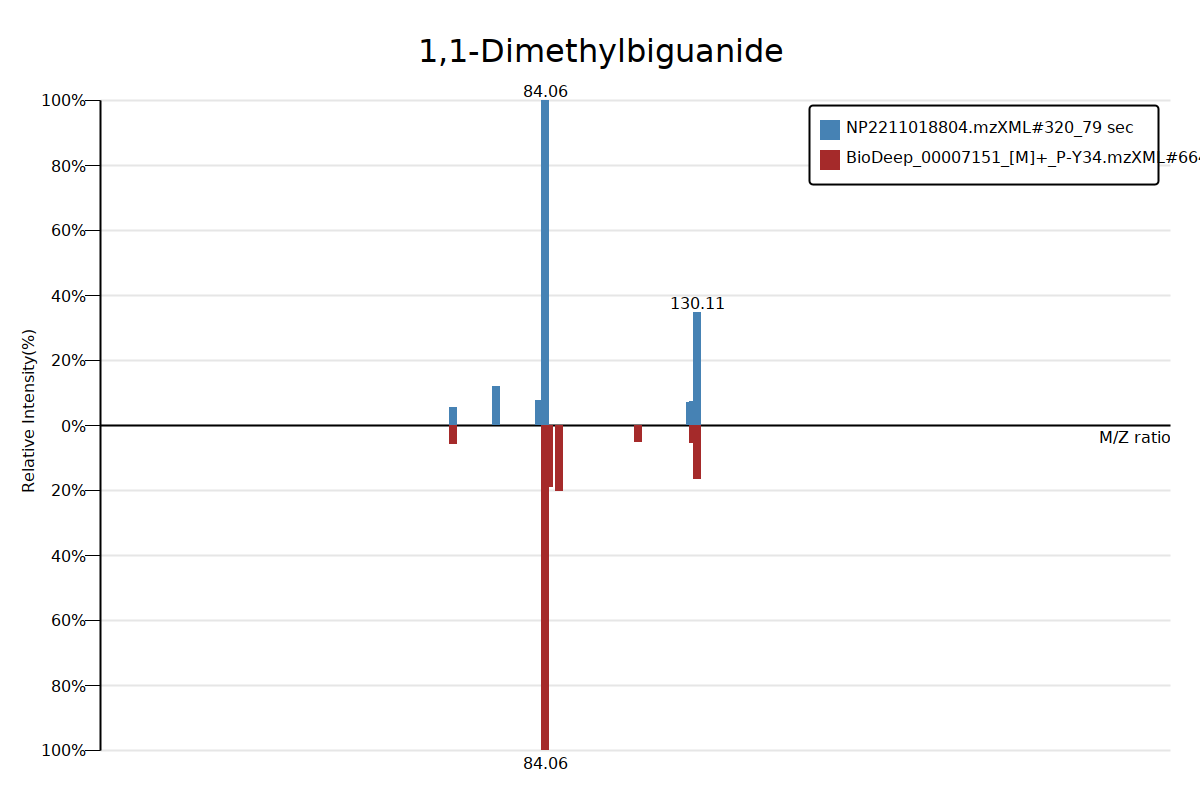

Supplement: Supplementary file 5 [file DataSheet1.ZIP › 2 result graphs between the MSMS secondary fragments of each metabolite and the MSMS secondary fragments of the standard substance in the database/1,1-Dimethylbiguanide.png]

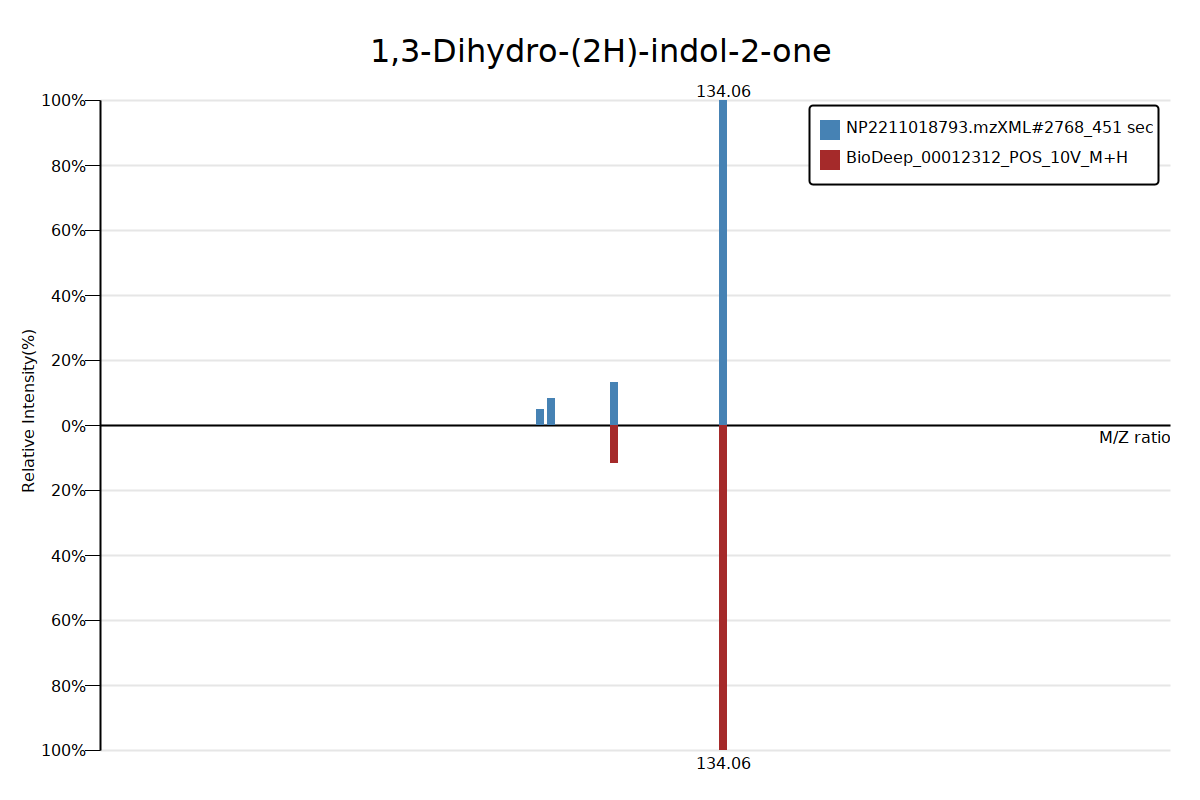

Supplement: Supplementary file 5 [file DataSheet1.ZIP › 2 result graphs between the MSMS secondary fragments of each metabolite and the MSMS secondary fragments of the standard substance in the database/1,3-Dihydro-(2H)-indol-2-one.png]

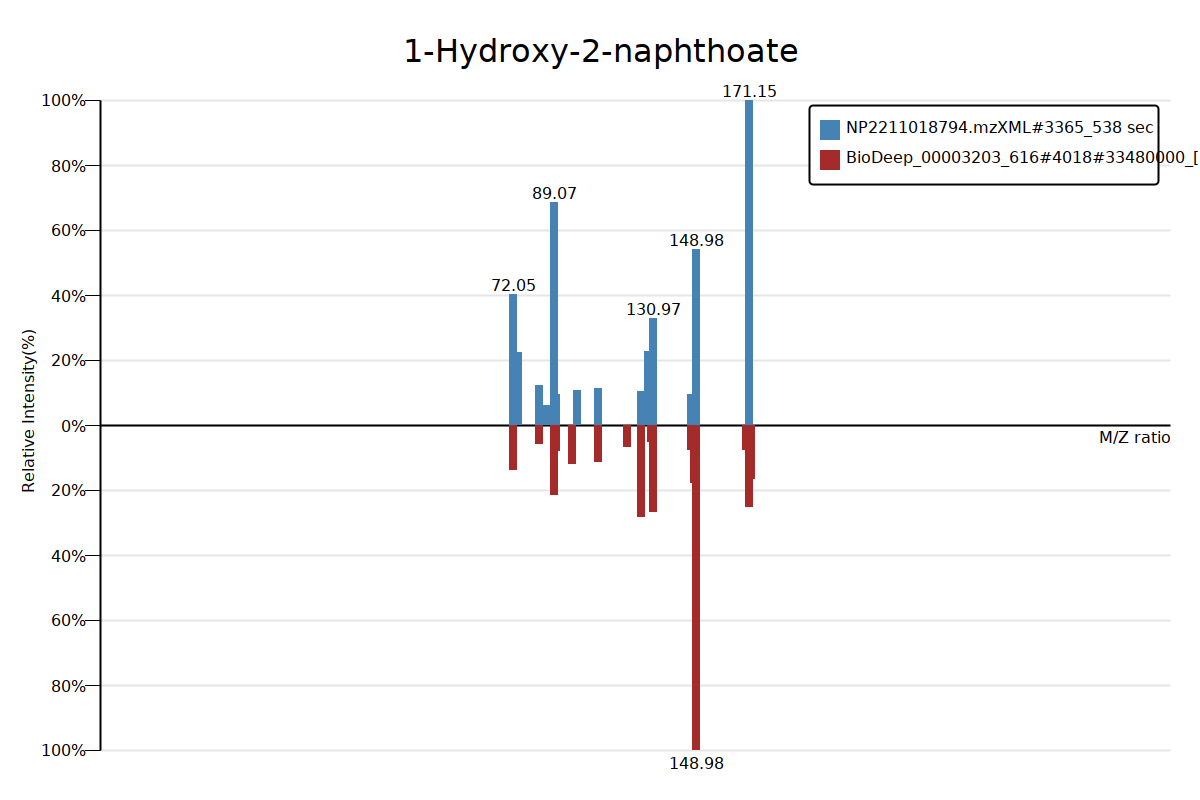

Supplement: Supplementary file 5 [file DataSheet1.ZIP › 2 result graphs between the MSMS secondary fragments of each metabolite and the MSMS secondary fragments of the standard substance in the database/1-Hydroxy-2-naphthoate.png]

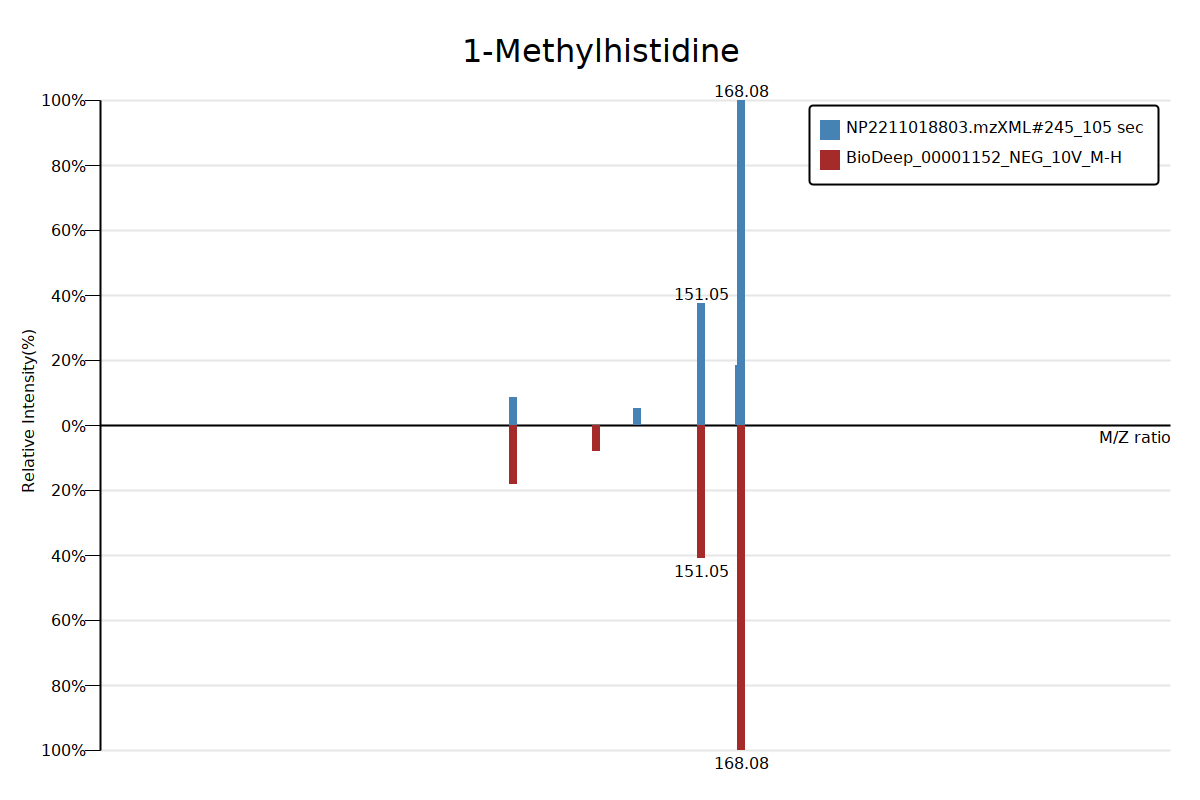

Supplement: Supplementary file 5 [file DataSheet1.ZIP › 2 result graphs between the MSMS secondary fragments of each metabolite and the MSMS secondary fragments of the standard substance in the database/1-Methylhistidine.png]

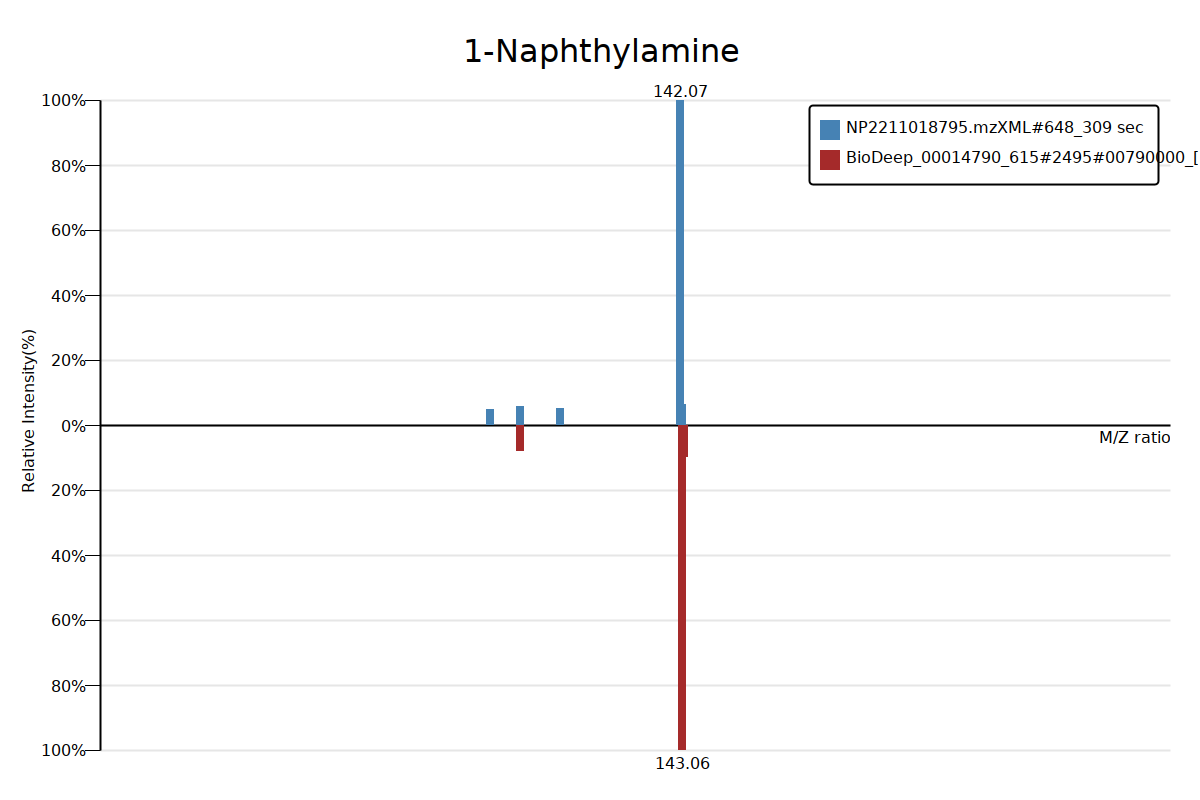

Supplement: Supplementary file 5 [file DataSheet1.ZIP › 2 result graphs between the MSMS secondary fragments of each metabolite and the MSMS secondary fragments of the standard substance in the database/1-Naphthylamine.png]

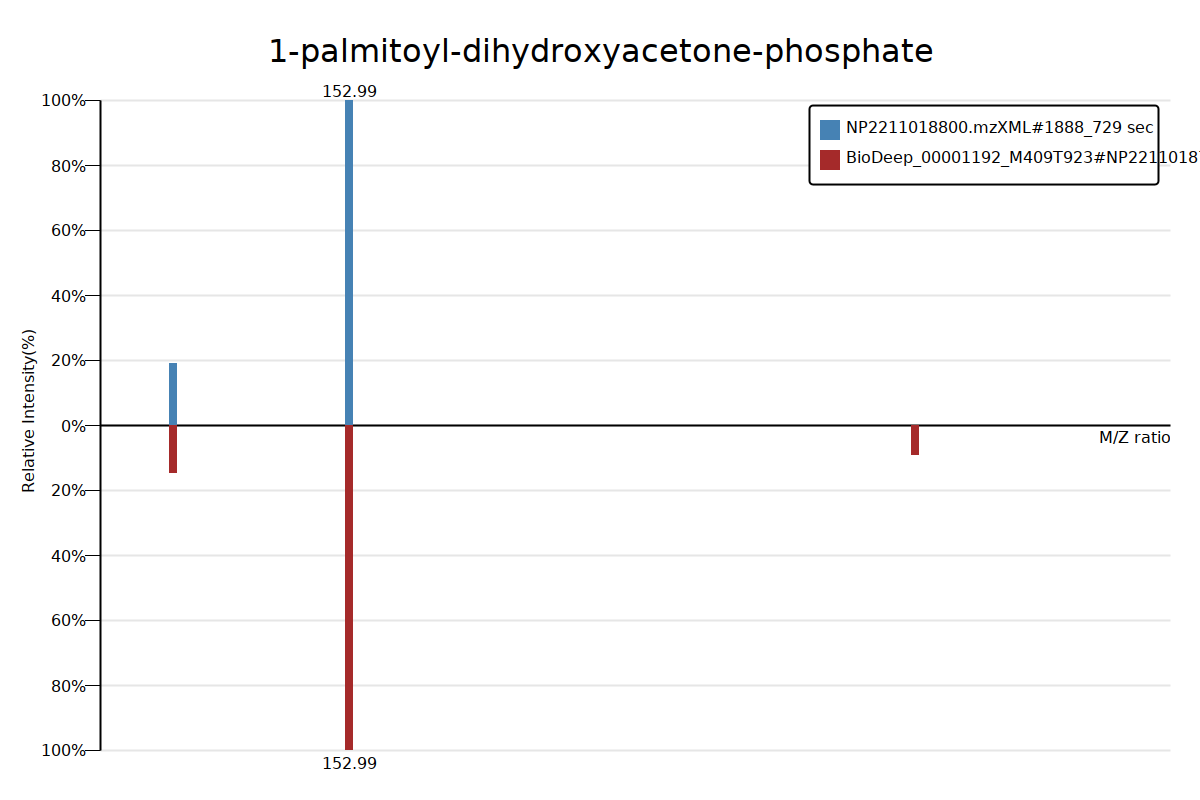

Supplement: Supplementary file 5 [file DataSheet1.ZIP › 2 result graphs between the MSMS secondary fragments of each metabolite and the MSMS secondary fragments of the standard substance in the database/1-palmitoyl-dihydroxyacetone-phosphate.png]

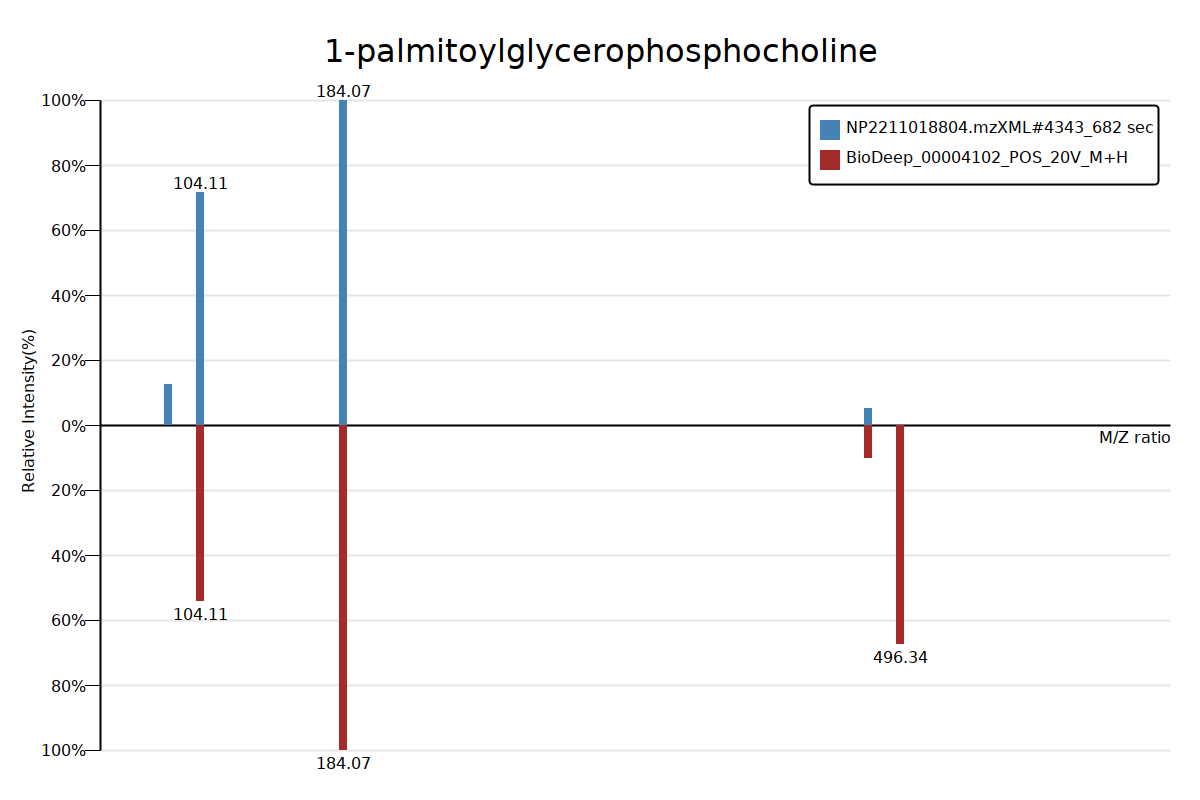

Supplement: Supplementary file 5 [file DataSheet1.ZIP › 2 result graphs between the MSMS secondary fragments of each metabolite and the MSMS secondary fragments of the standard substance in the database/1-palmitoylglycerophosphocholine.png]

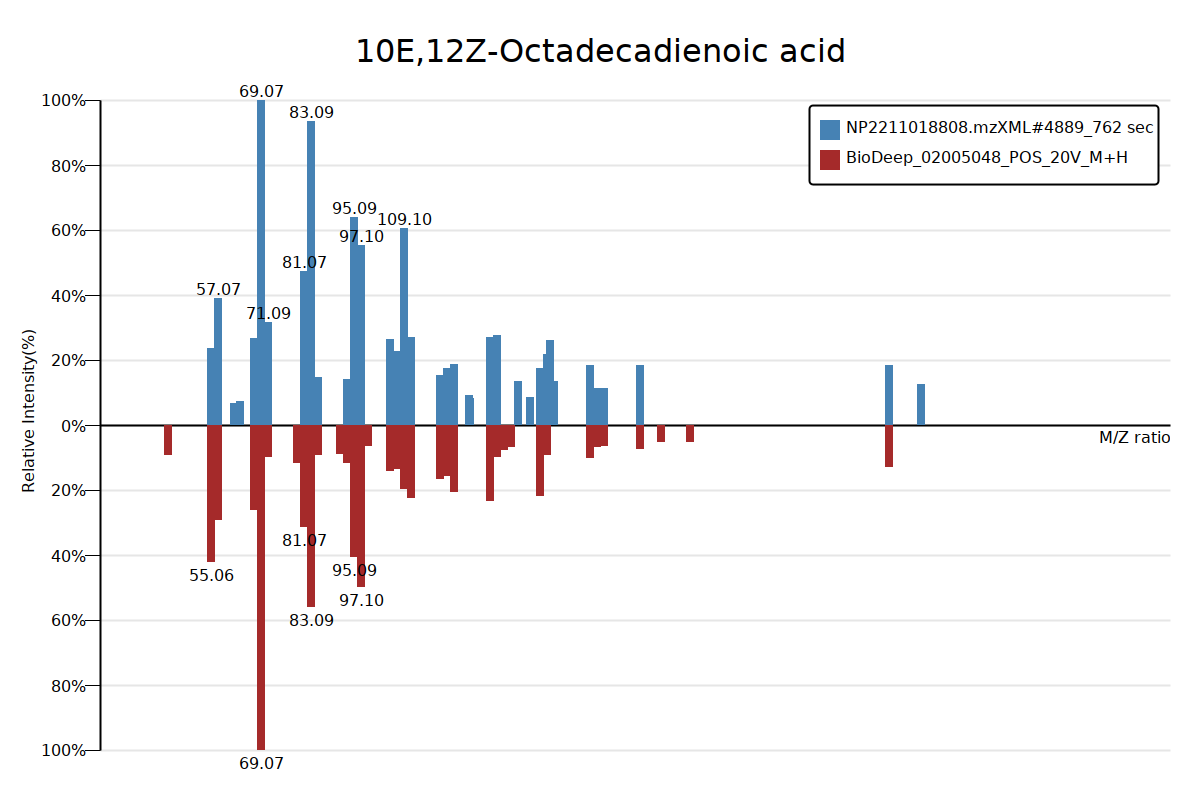

Supplement: Supplementary file 5 [file DataSheet1.ZIP › 2 result graphs between the MSMS secondary fragments of each metabolite and the MSMS secondary fragments of the standard substance in the database/10E,12Z-Octadecadienoic acid.png]

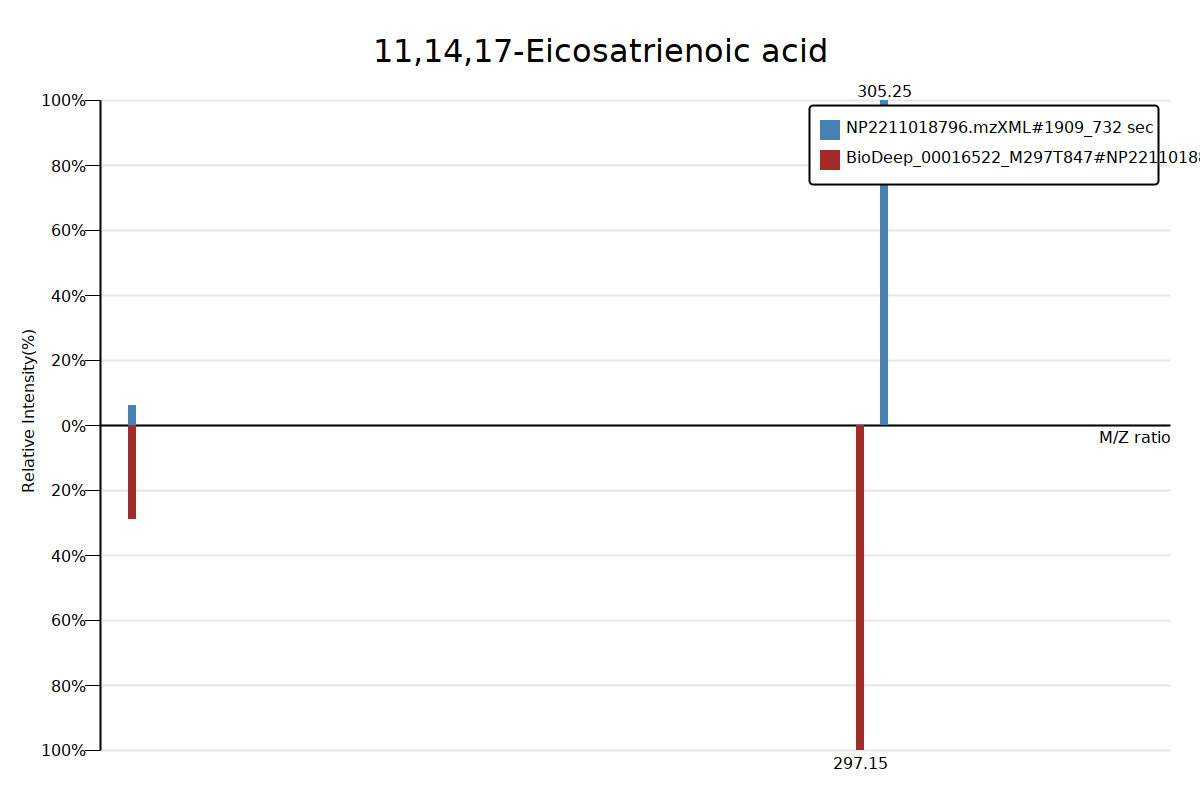

Supplement: Supplementary file 5 [file DataSheet1.ZIP › 2 result graphs between the MSMS secondary fragments of each metabolite and the MSMS secondary fragments of the standard substance in the database/11,14,17-Eicosatrienoic acid.png]

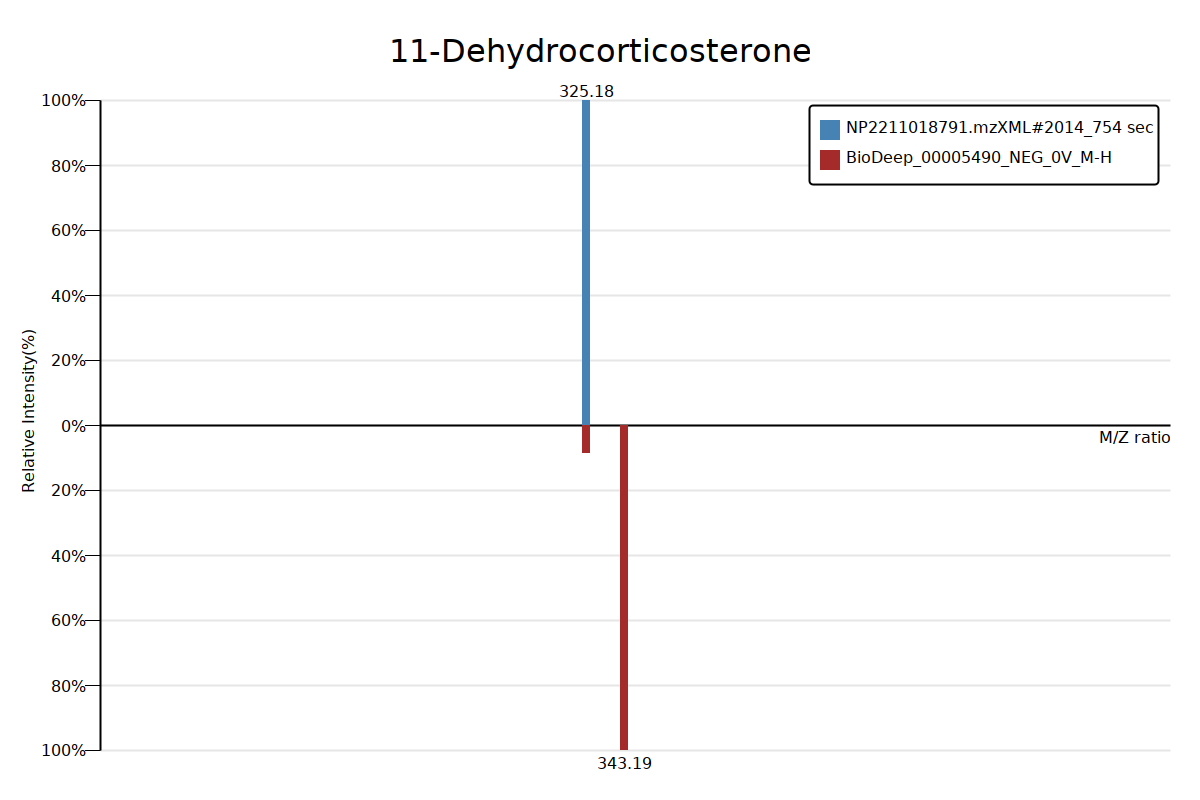

Supplement: Supplementary file 5 [file DataSheet1.ZIP › 2 result graphs between the MSMS secondary fragments of each metabolite and the MSMS secondary fragments of the standard substance in the database/11-Dehydrocorticosterone.png]

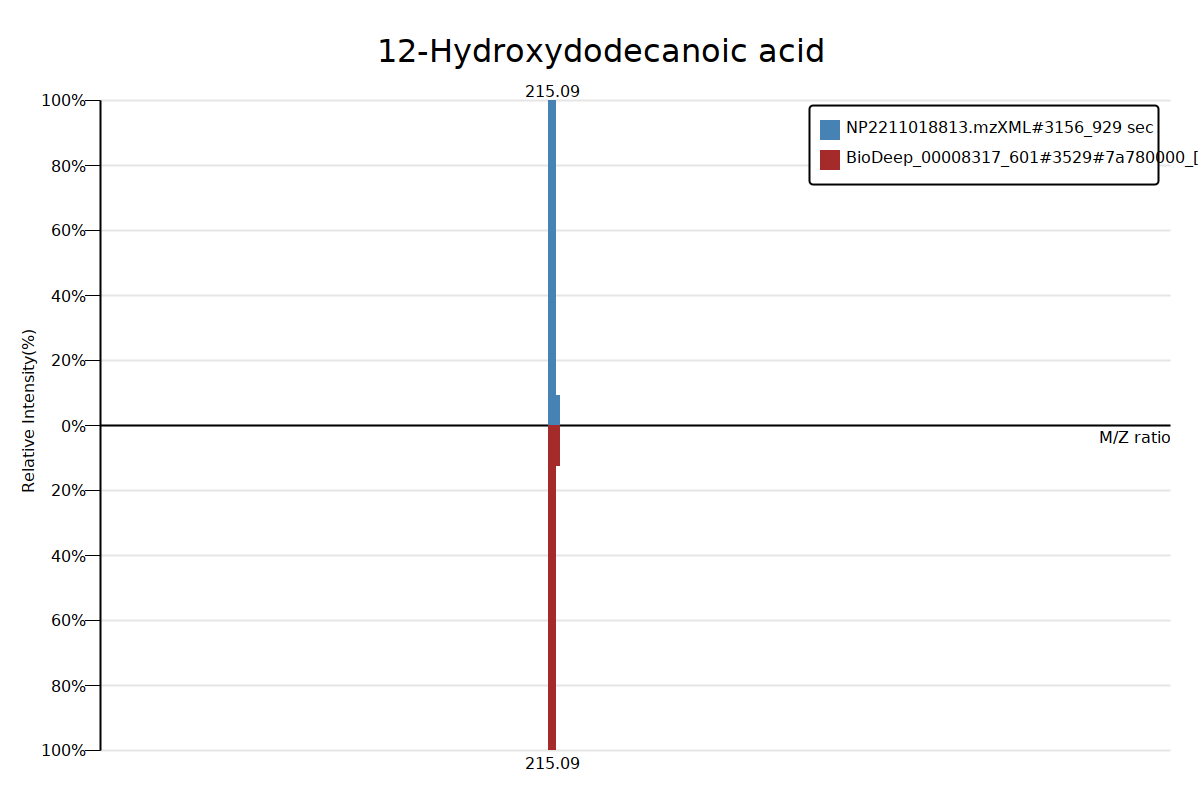

Supplement: Supplementary file 5 [file DataSheet1.ZIP › 2 result graphs between the MSMS secondary fragments of each metabolite and the MSMS secondary fragments of the standard substance in the database/12-Hydroxydodecanoic acid.png]

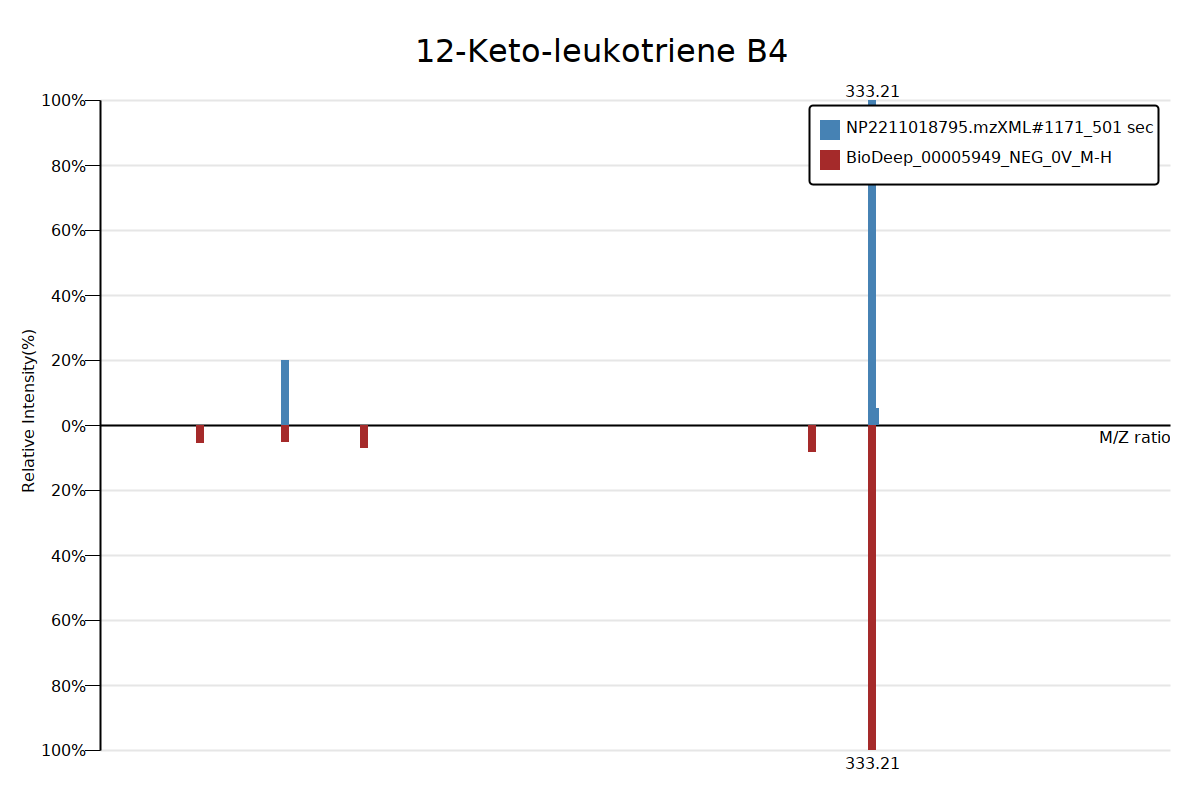

Supplement: Supplementary file 5 [file DataSheet1.ZIP › 2 result graphs between the MSMS secondary fragments of each metabolite and the MSMS secondary fragments of the standard substance in the database/12-Keto-leukotriene B4.png]

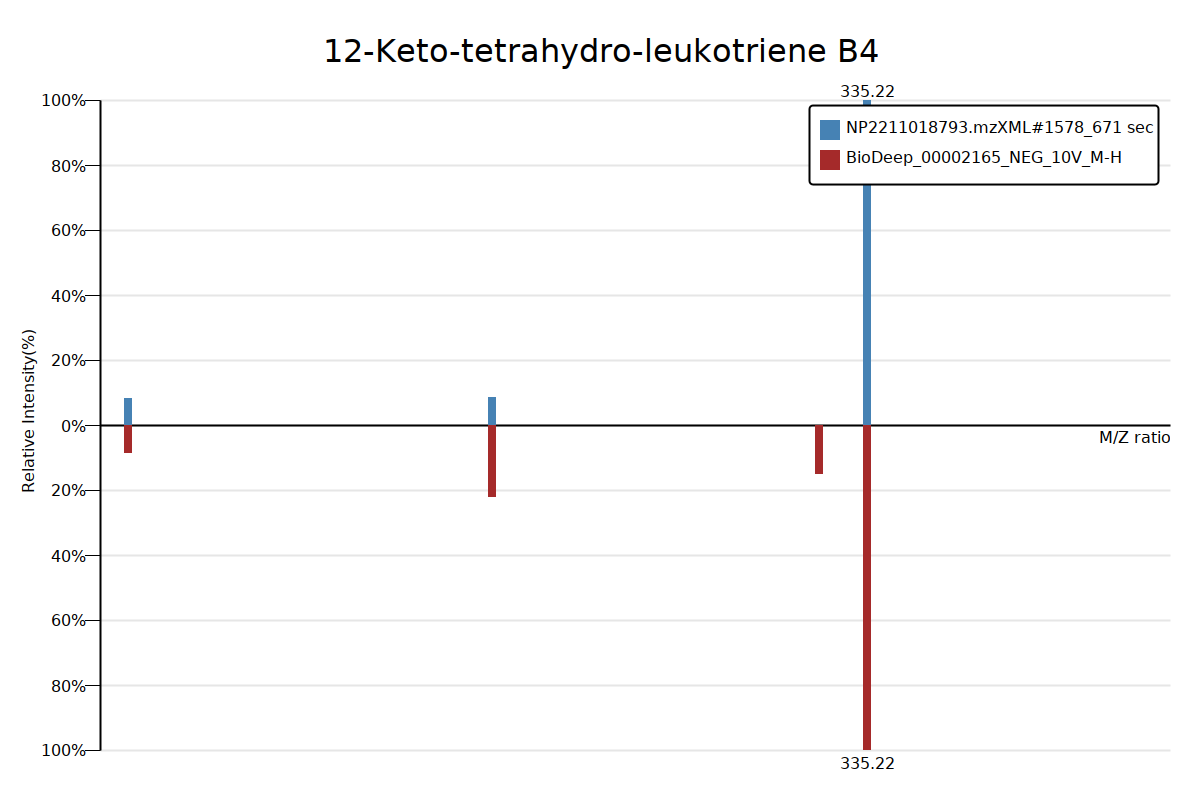

Supplement: Supplementary file 5 [file DataSheet1.ZIP › 2 result graphs between the MSMS secondary fragments of each metabolite and the MSMS secondary fragments of the standard substance in the database/12-Keto-tetrahydro-leukotriene B4.png]

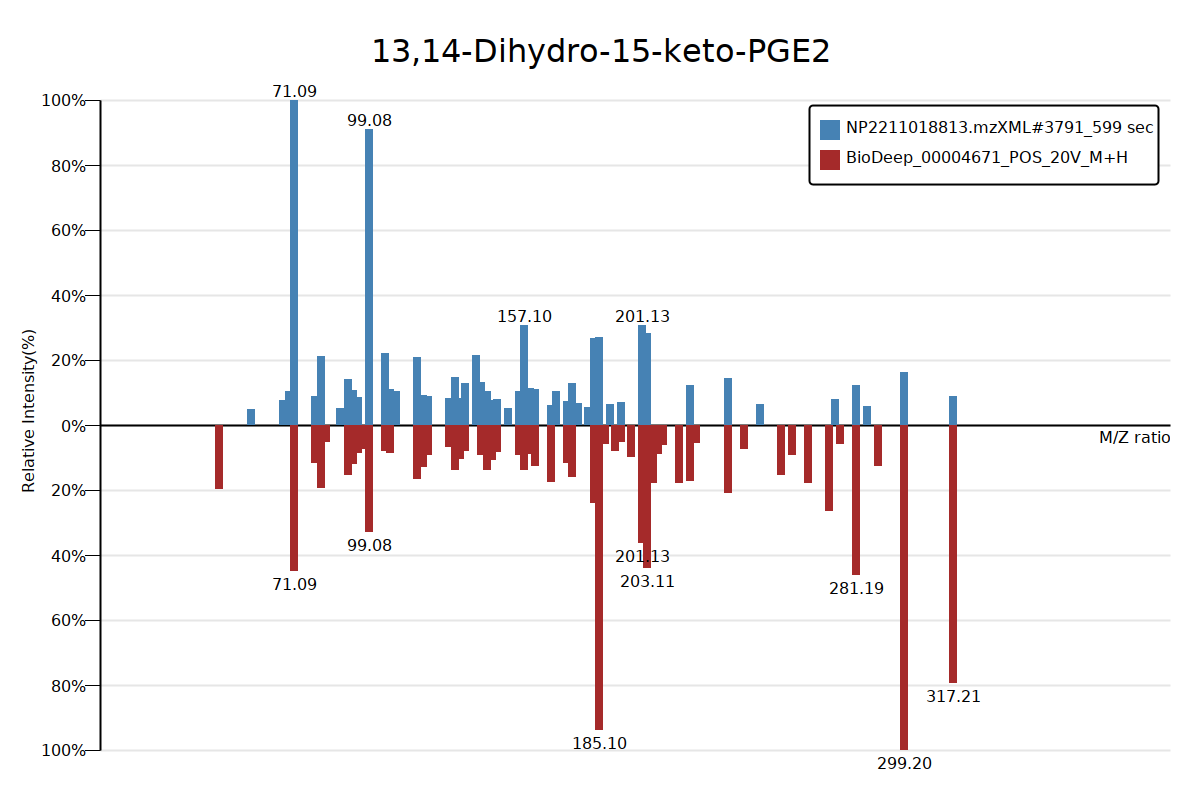

Supplement: Supplementary file 5 [file DataSheet1.ZIP › 2 result graphs between the MSMS secondary fragments of each metabolite and the MSMS secondary fragments of the standard substance in the database/13,14-Dihydro-15-keto-PGE2.png]

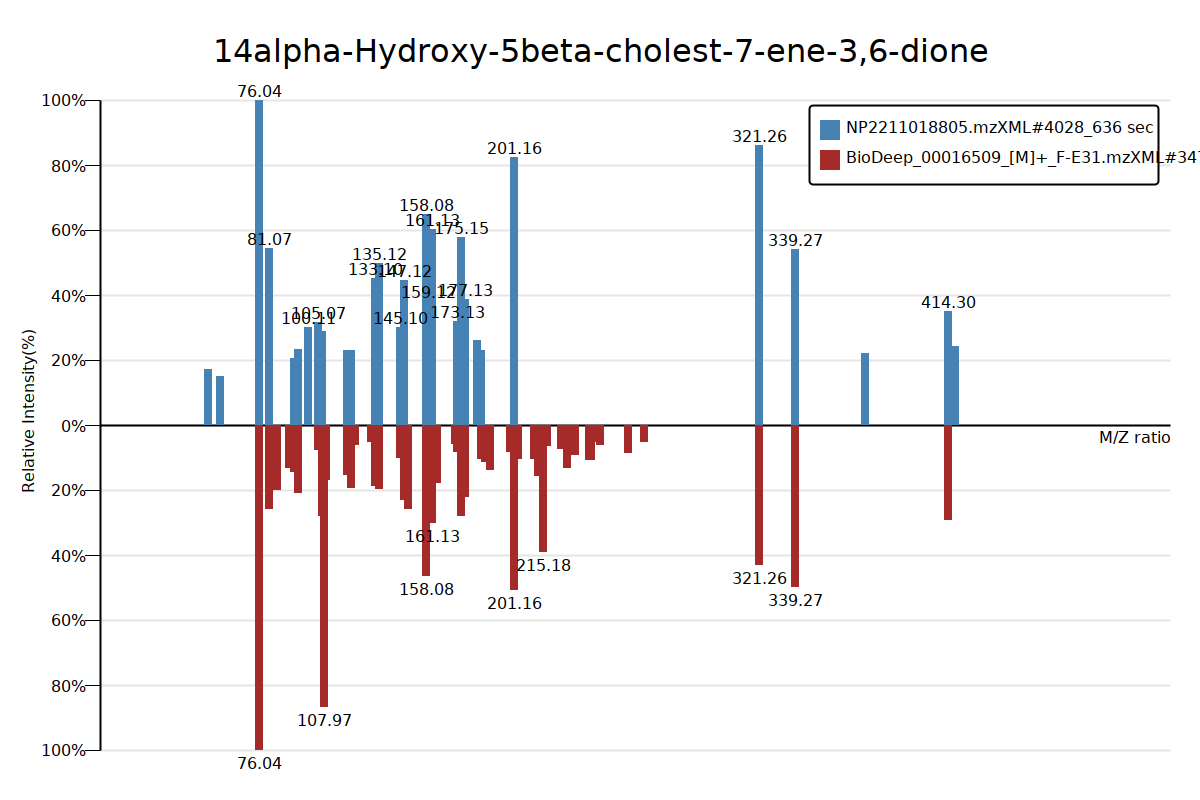

Supplement: Supplementary file 5 [file DataSheet1.ZIP › 2 result graphs between the MSMS secondary fragments of each metabolite and the MSMS secondary fragments of the standard substance in the database/14alpha-Hydroxy-5beta-cholest-7-ene-3,6-dione.png]

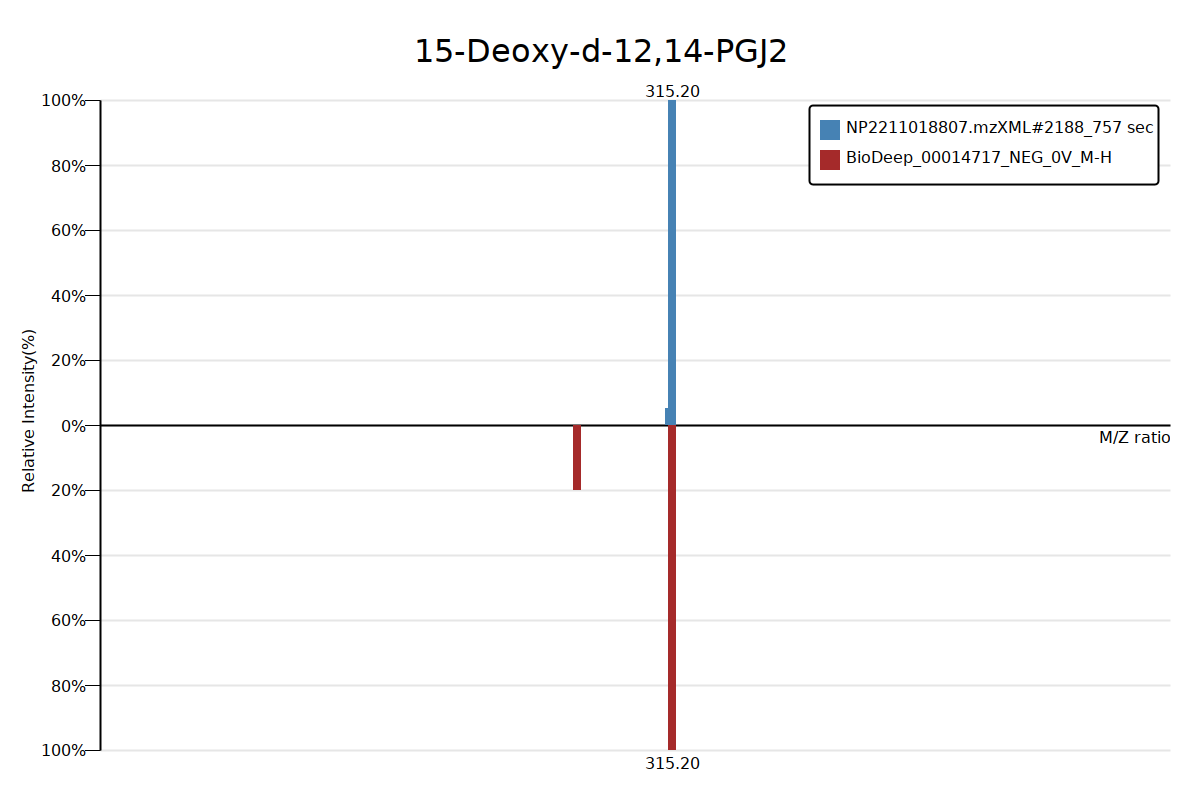

Supplement: Supplementary file 5 [file DataSheet1.ZIP › 2 result graphs between the MSMS secondary fragments of each metabolite and the MSMS secondary fragments of the standard substance in the database/15-Deoxy-d-12,14-PGJ2.png]

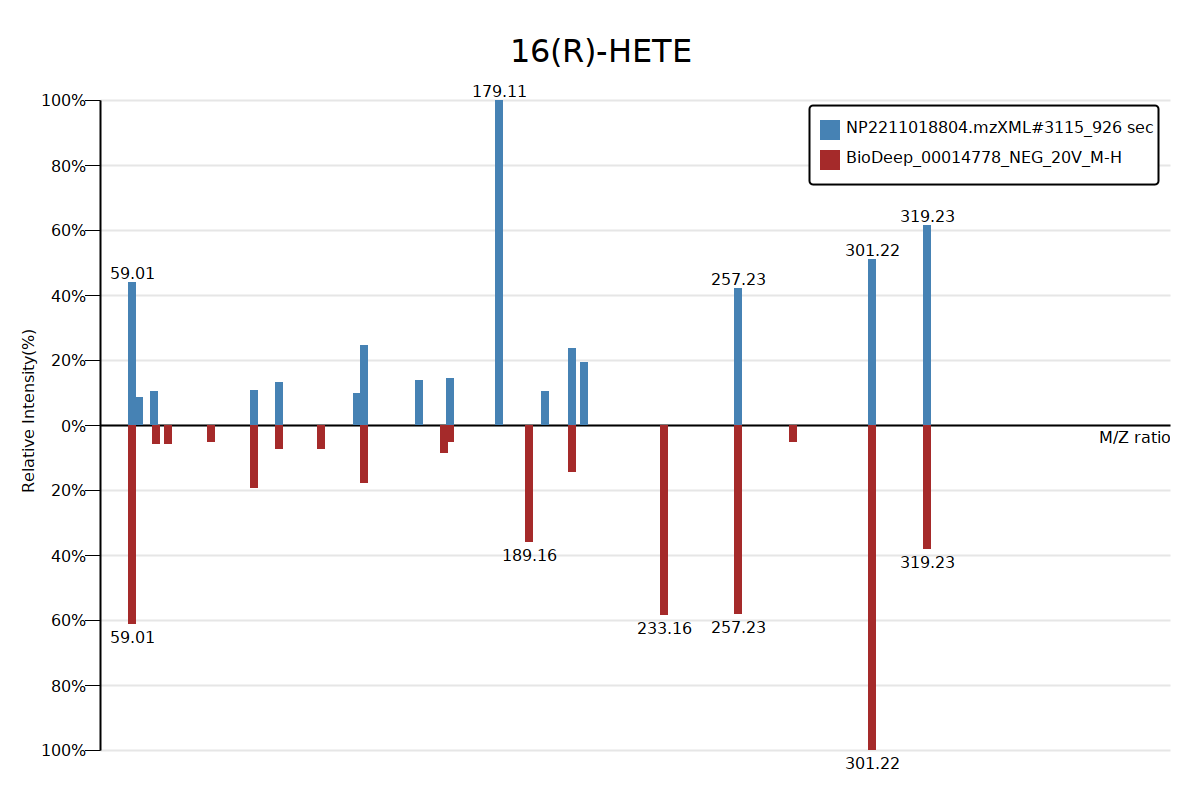

Supplement: Supplementary file 5 [file DataSheet1.ZIP › 2 result graphs between the MSMS secondary fragments of each metabolite and the MSMS secondary fragments of the standard substance in the database/16(R)-HETE.png]

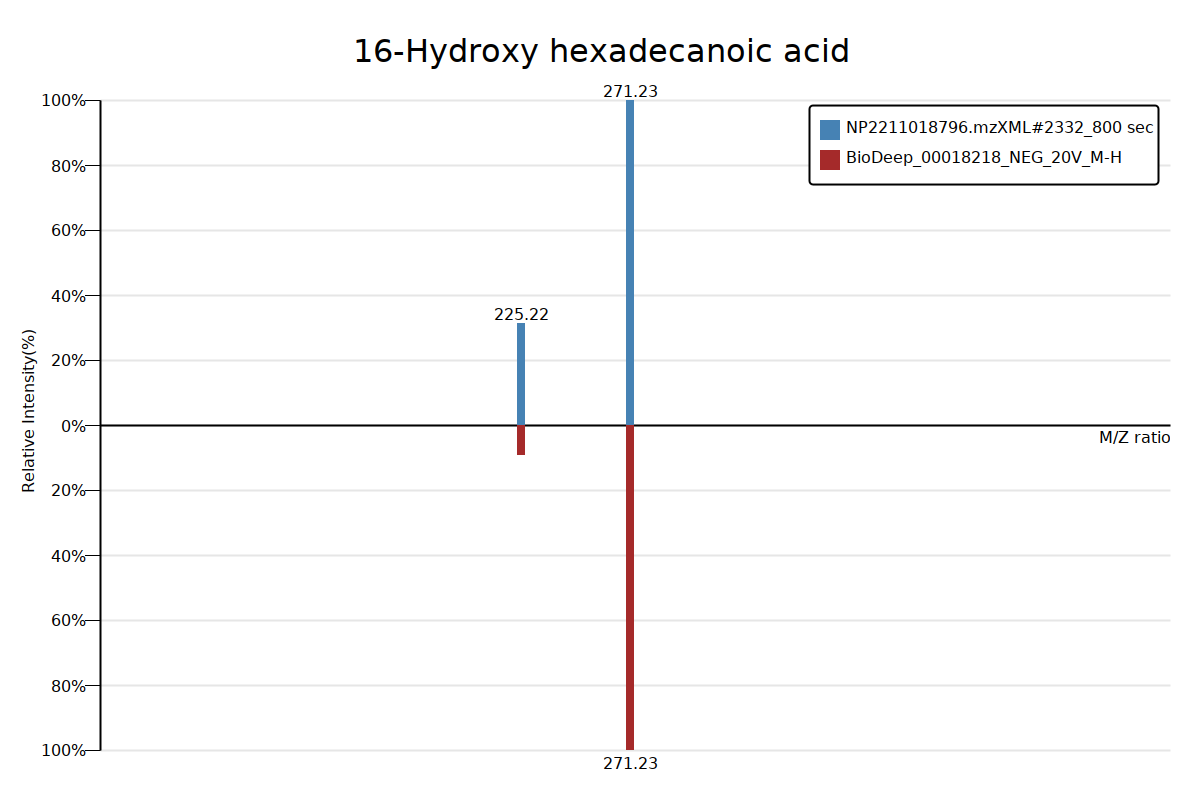

Supplement: Supplementary file 5 [file DataSheet1.ZIP › 2 result graphs between the MSMS secondary fragments of each metabolite and the MSMS secondary fragments of the standard substance in the database/16-Hydroxy hexadecanoic acid.png]

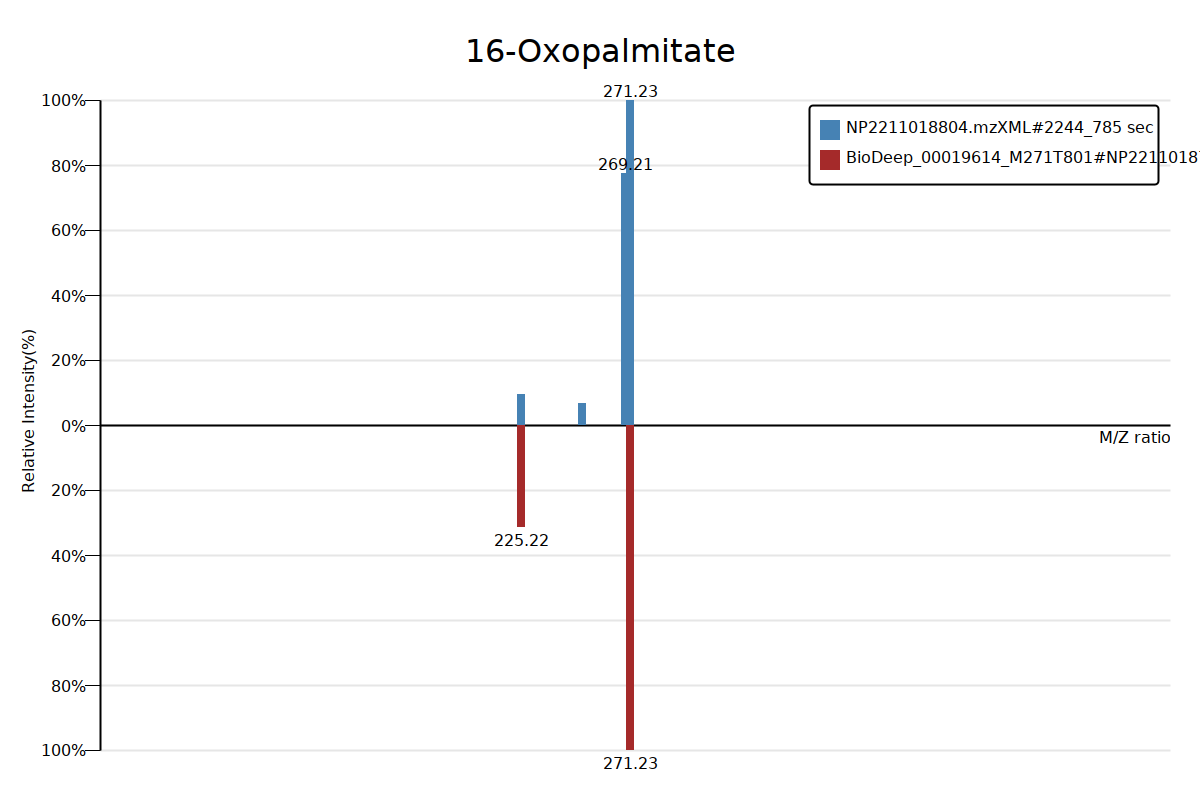

Supplement: Supplementary file 5 [file DataSheet1.ZIP › 2 result graphs between the MSMS secondary fragments of each metabolite and the MSMS secondary fragments of the standard substance in the database/16-Oxopalmitate.png]

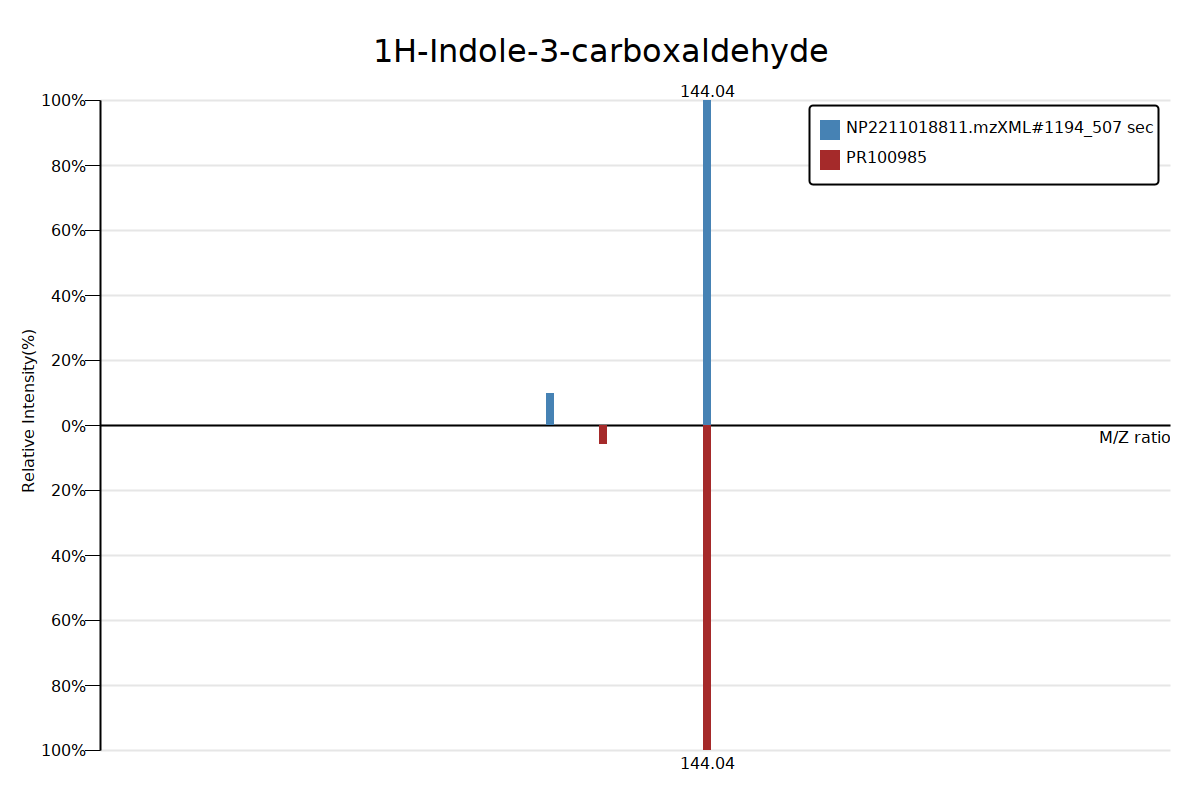

Supplement: Supplementary file 5 [file DataSheet1.ZIP › 2 result graphs between the MSMS secondary fragments of each metabolite and the MSMS secondary fragments of the standard substance in the database/1H-Indole-3-carboxaldehyde.png]

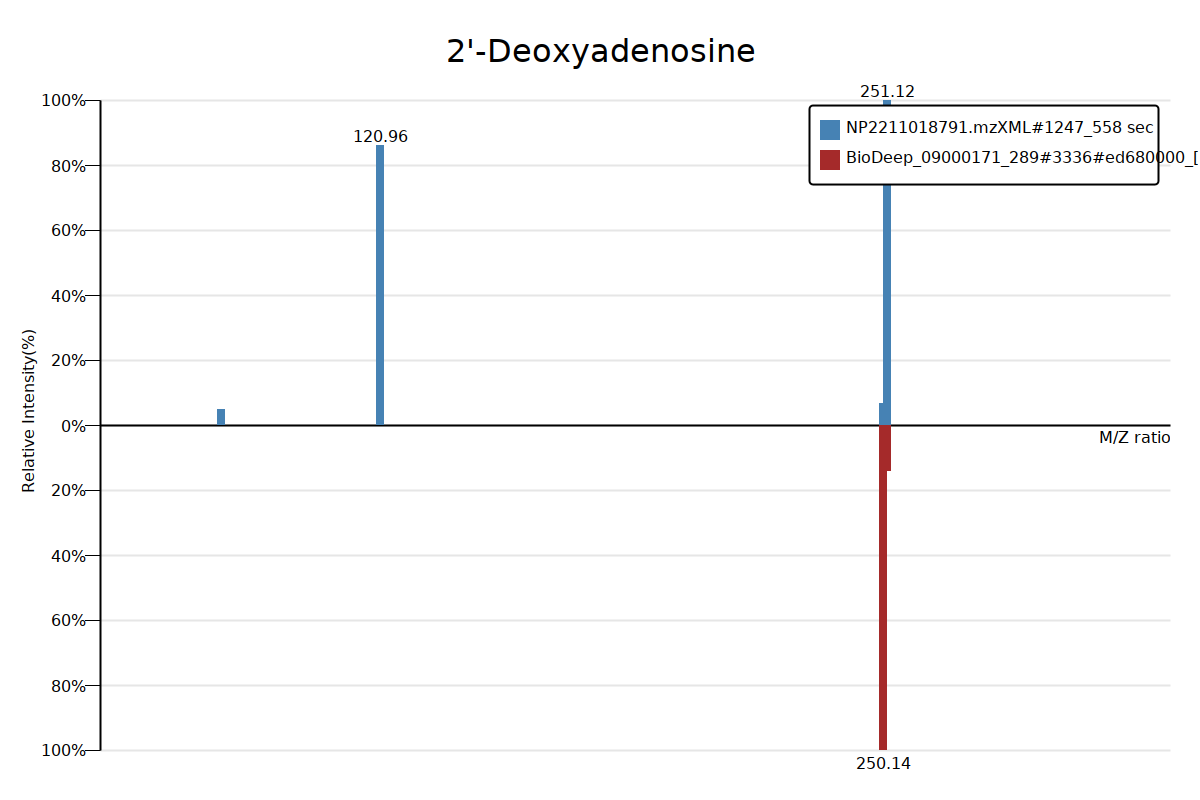

Supplement: Supplementary file 5 [file DataSheet1.ZIP › 2 result graphs between the MSMS secondary fragments of each metabolite and the MSMS secondary fragments of the standard substance in the database/2'-Deoxyadenosine.png]

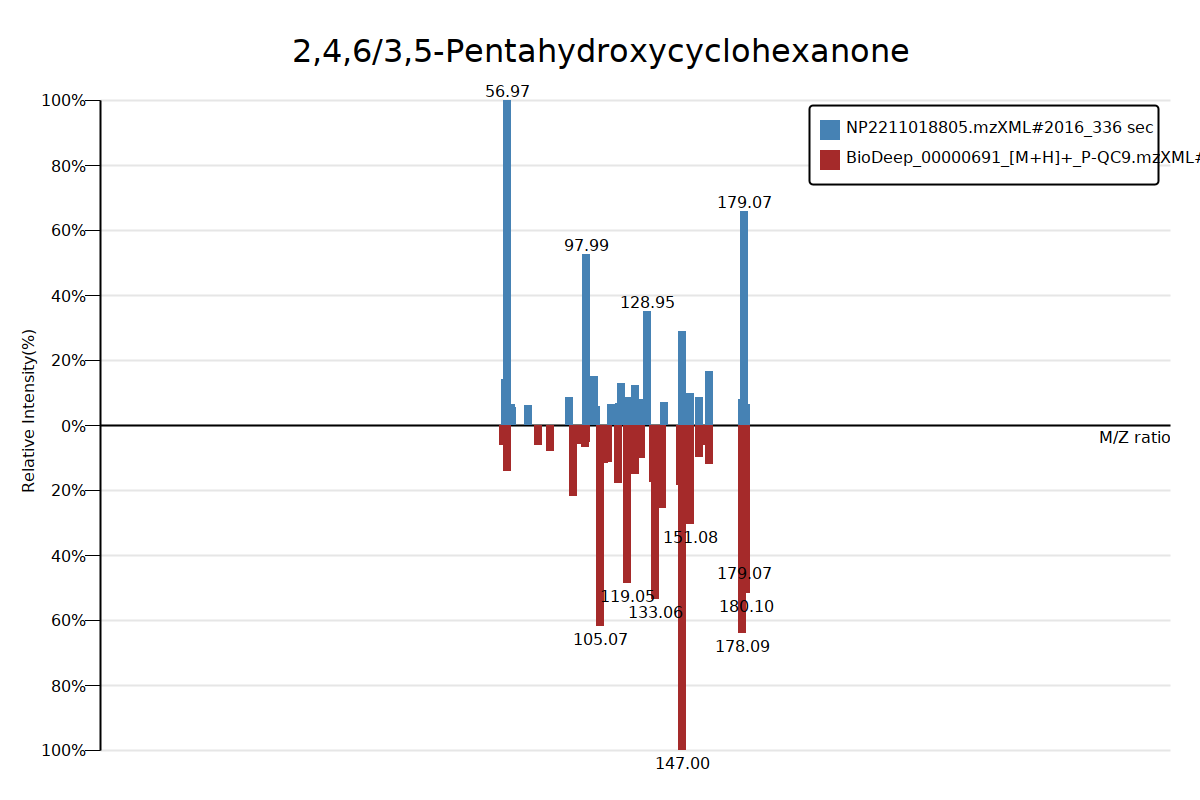

Supplement: Supplementary file 5 [file DataSheet1.ZIP › 2 result graphs between the MSMS secondary fragments of each metabolite and the MSMS secondary fragments of the standard substance in the database/2,4,6_3,5-Pentahydroxycyclohexanone.png]

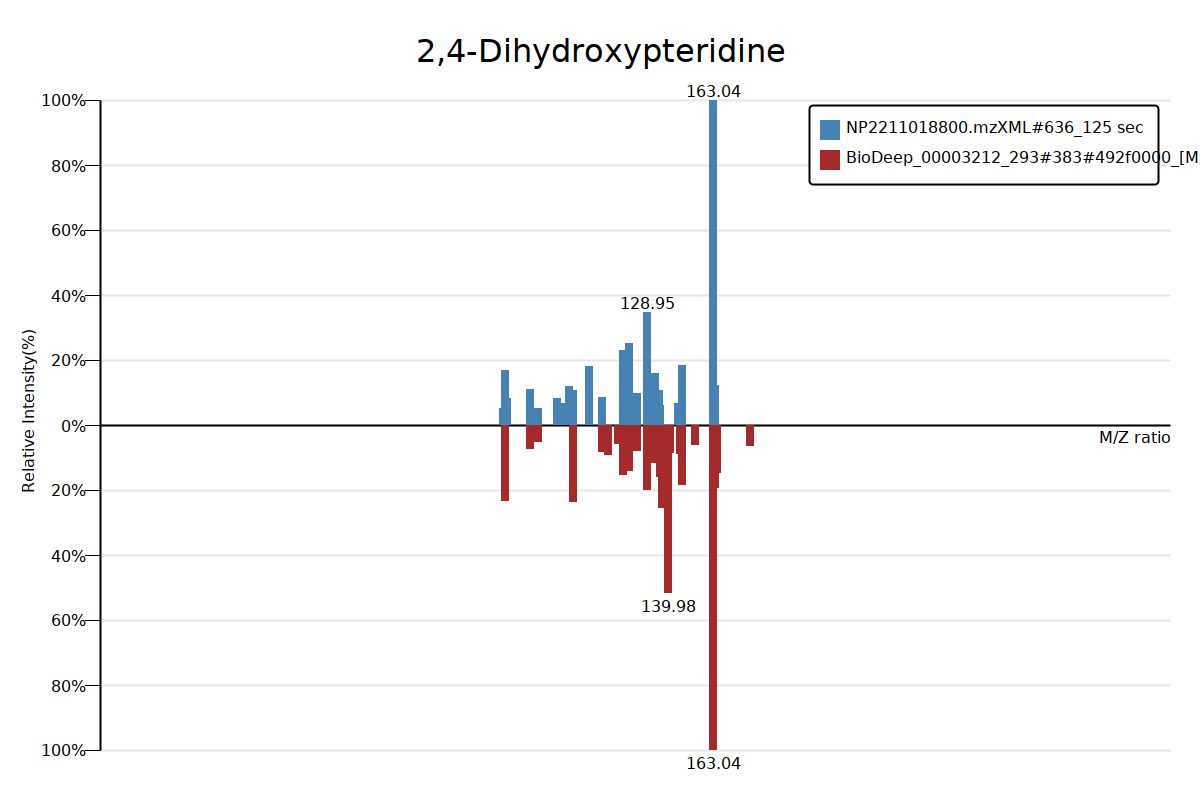

Supplement: Supplementary file 5 [file DataSheet1.ZIP › 2 result graphs between the MSMS secondary fragments of each metabolite and the MSMS secondary fragments of the standard substance in the database/2,4-Dihydroxypteridine.png]

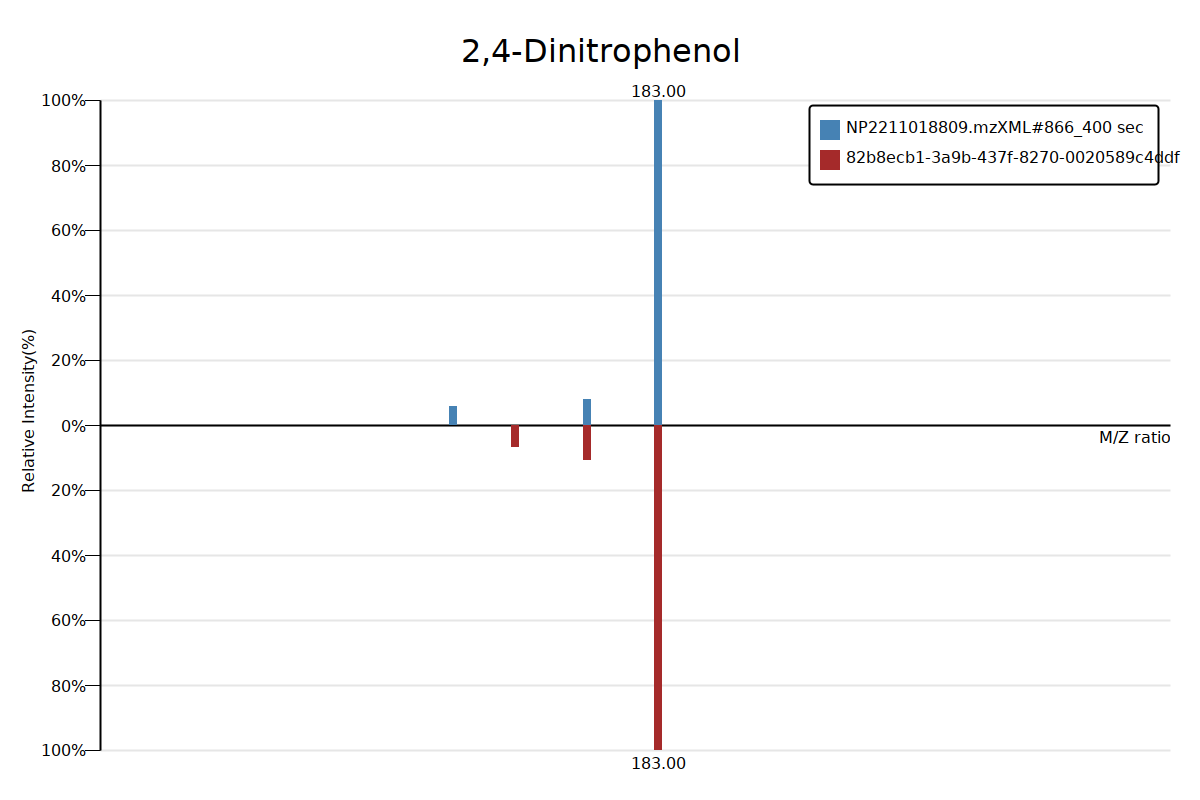

Supplement: Supplementary file 5 [file DataSheet1.ZIP › 2 result graphs between the MSMS secondary fragments of each metabolite and the MSMS secondary fragments of the standard substance in the database/2,4-Dinitrophenol.png]

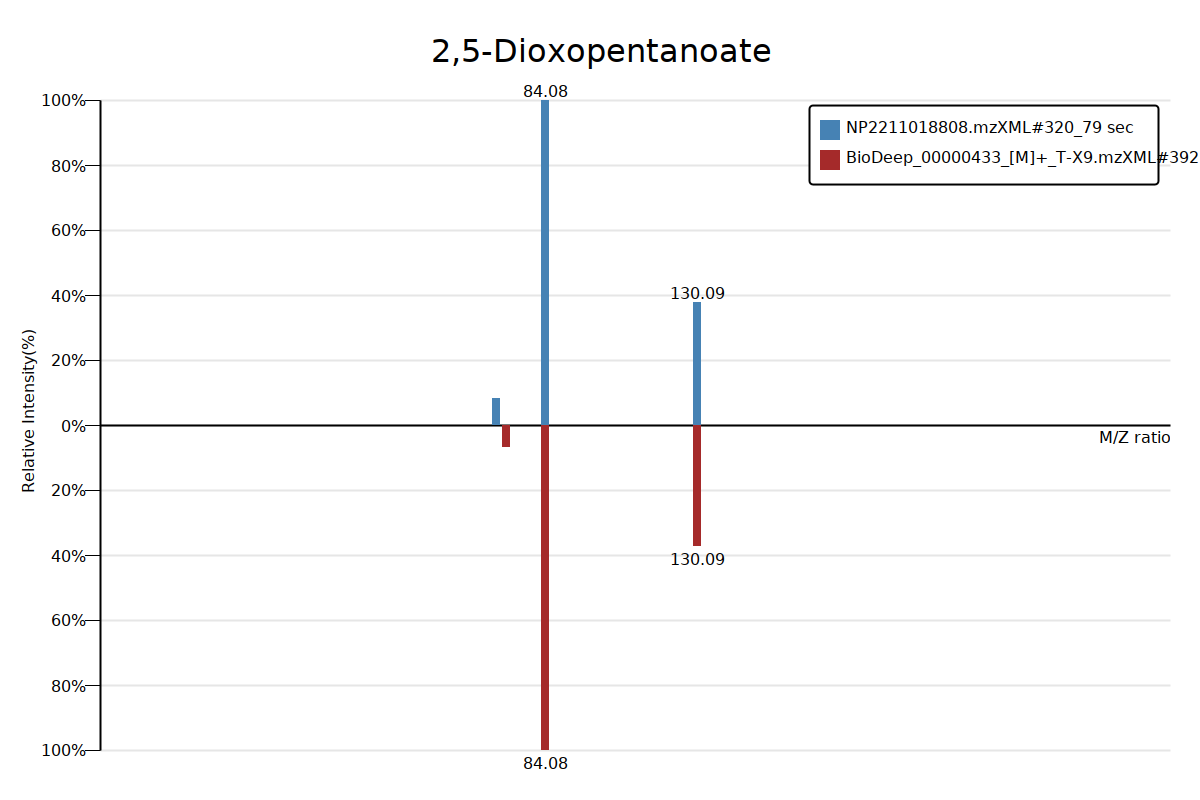

Supplement: Supplementary file 5 [file DataSheet1.ZIP › 2 result graphs between the MSMS secondary fragments of each metabolite and the MSMS secondary fragments of the standard substance in the database/2,5-Dioxopentanoate.png]

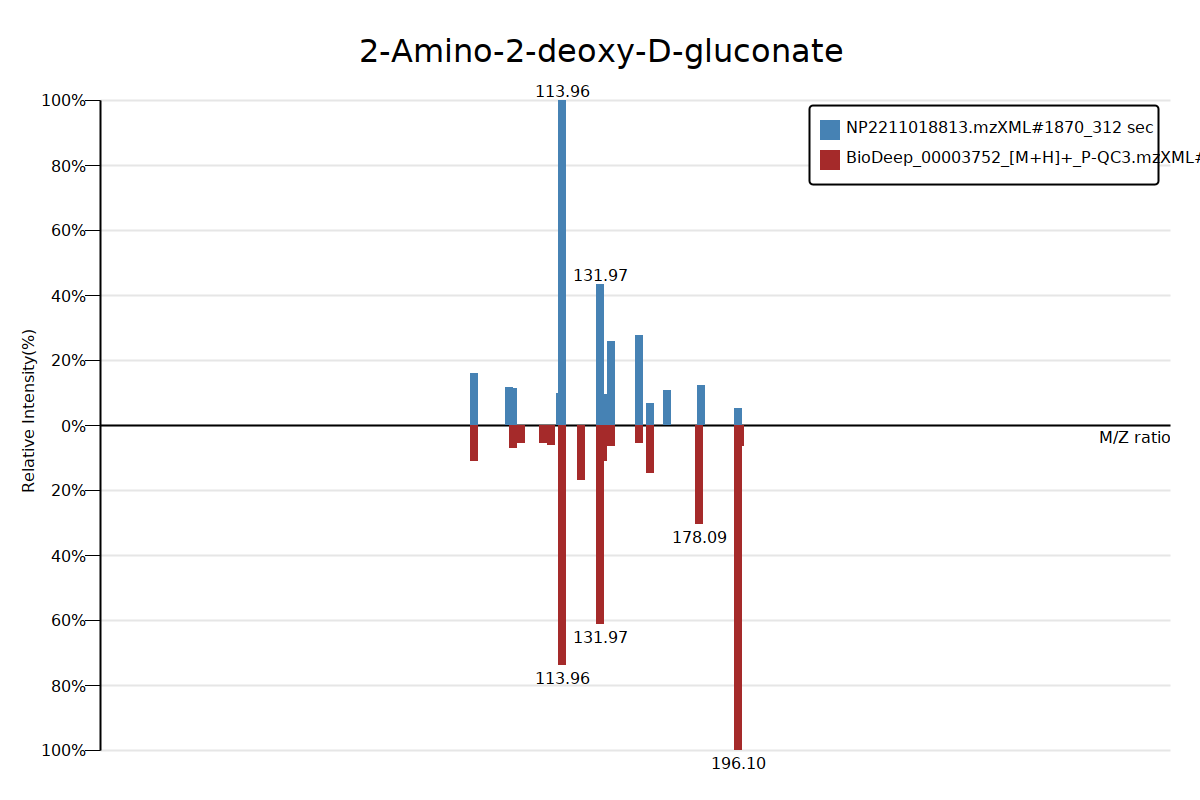

Supplement: Supplementary file 5 [file DataSheet1.ZIP › 2 result graphs between the MSMS secondary fragments of each metabolite and the MSMS secondary fragments of the standard substance in the database/2-Amino-2-deoxy-D-gluconate.png]

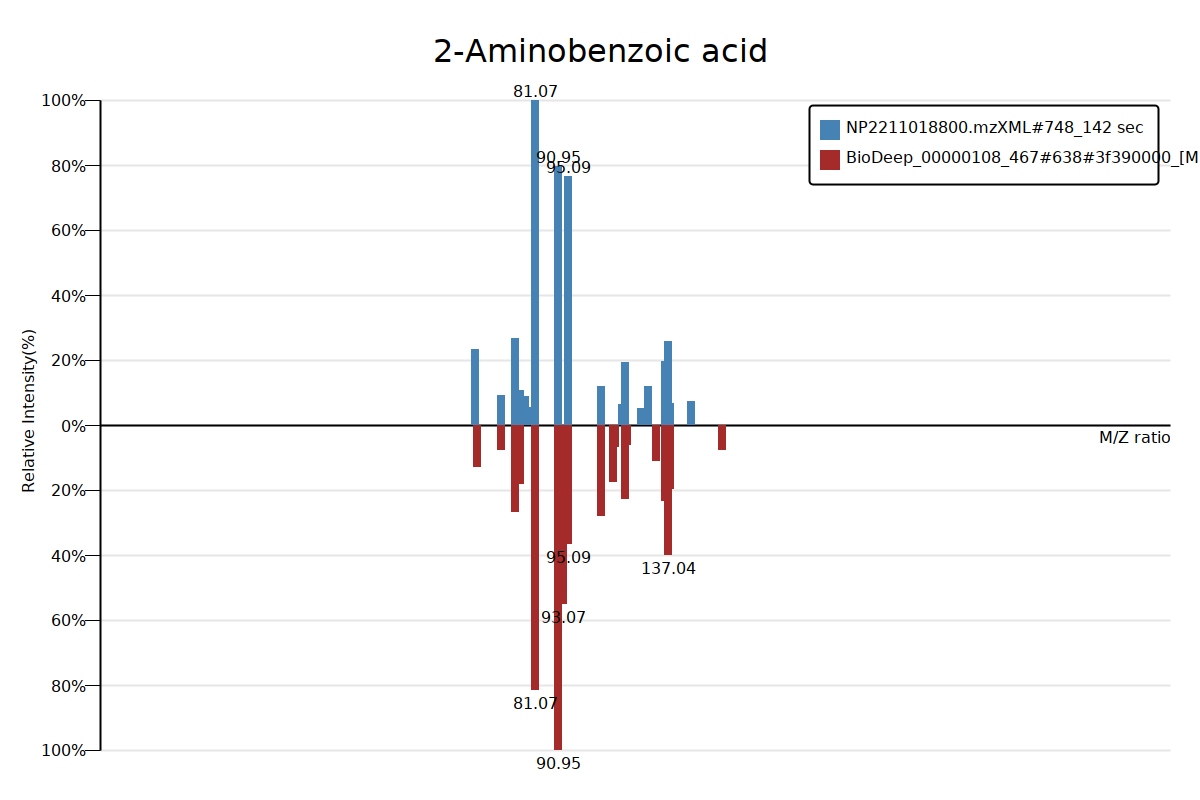

Supplement: Supplementary file 5 [file DataSheet1.ZIP › 2 result graphs between the MSMS secondary fragments of each metabolite and the MSMS secondary fragments of the standard substance in the database/2-Aminobenzoic acid.png]

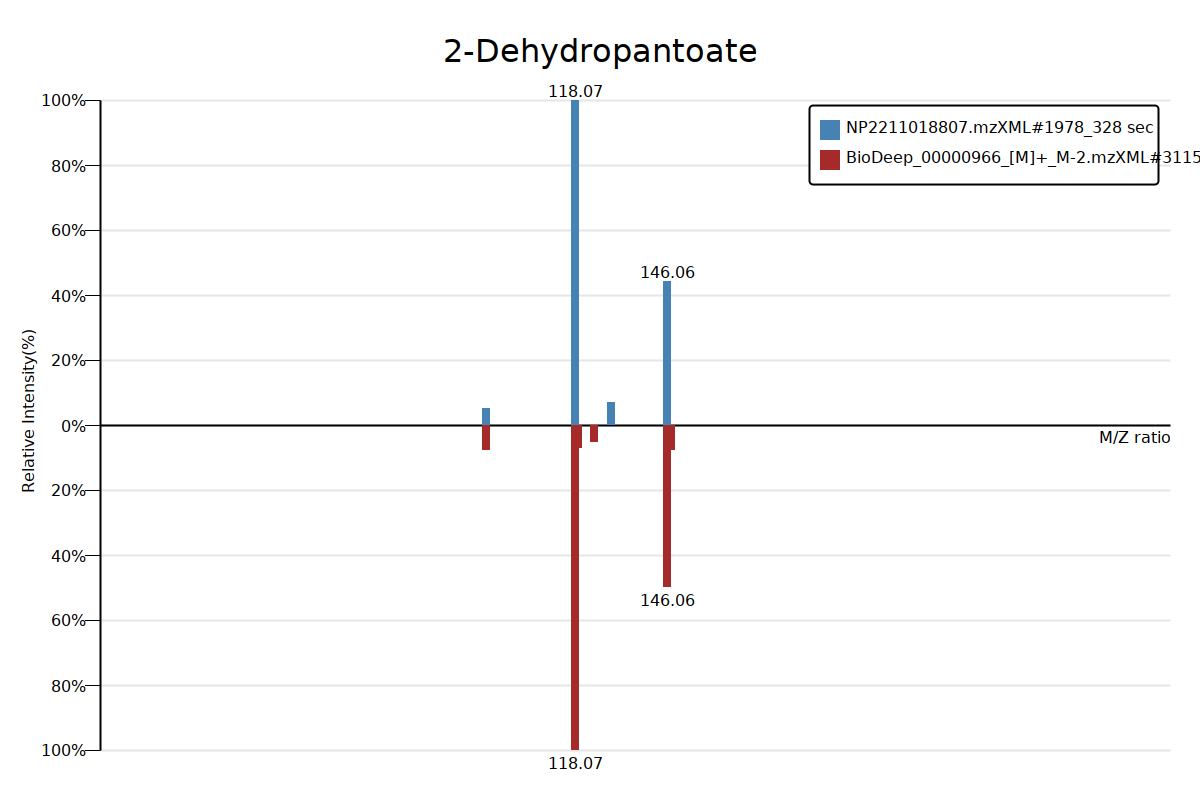

Supplement: Supplementary file 5 [file DataSheet1.ZIP › 2 result graphs between the MSMS secondary fragments of each metabolite and the MSMS secondary fragments of the standard substance in the database/2-Dehydropantoate.png]

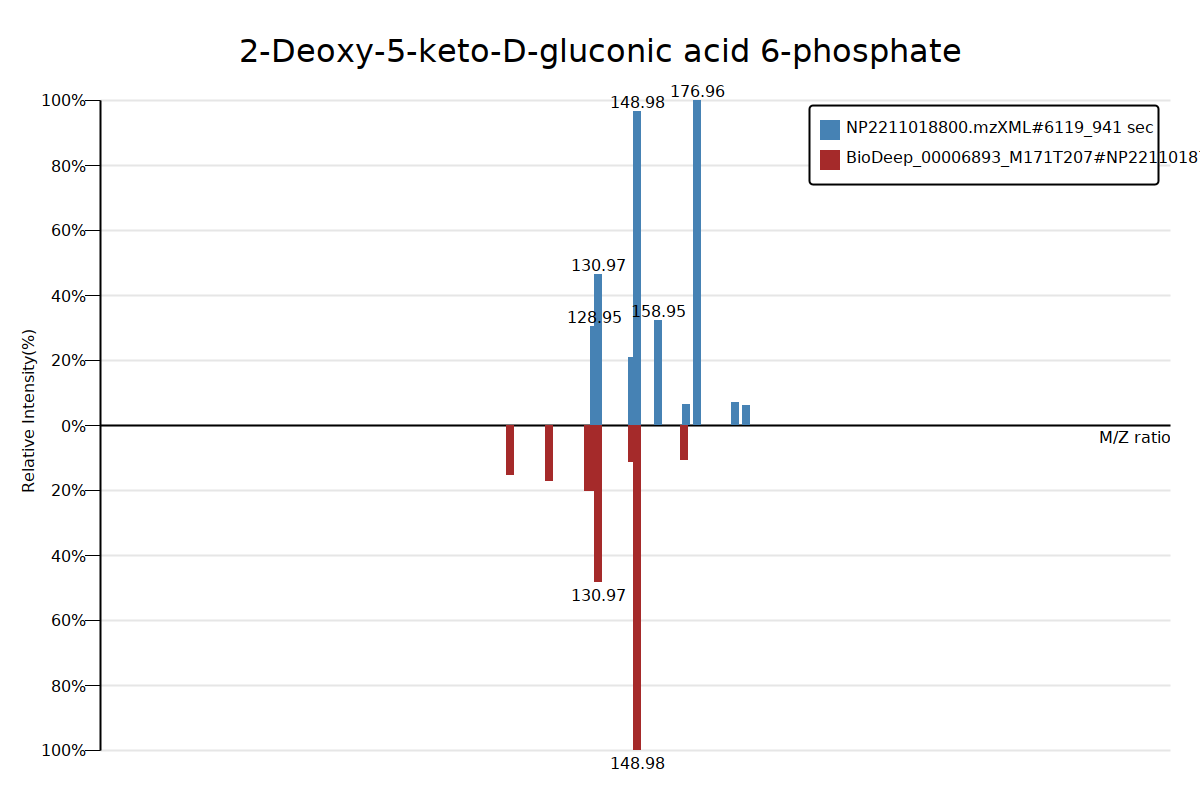

Supplement: Supplementary file 5 [file DataSheet1.ZIP › 2 result graphs between the MSMS secondary fragments of each metabolite and the MSMS secondary fragments of the standard substance in the database/2-Deoxy-5-keto-D-gluconic acid 6-phosphate.png]

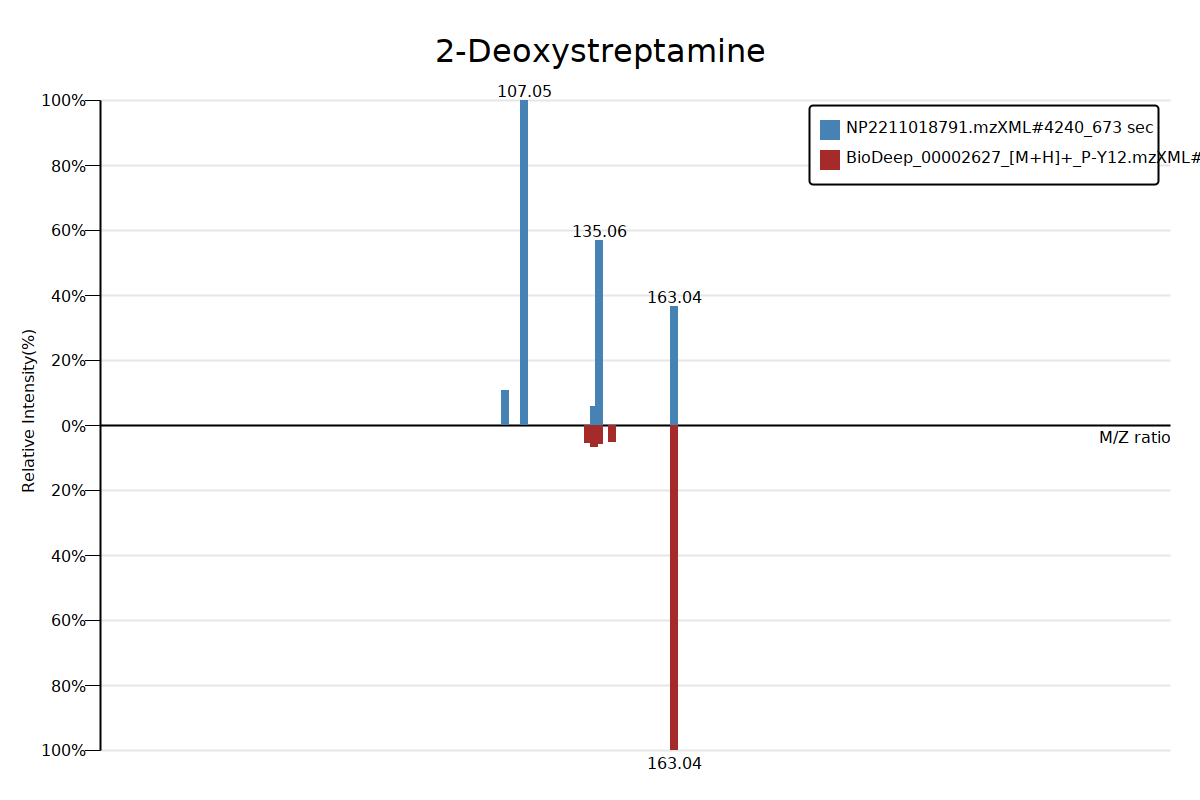

Supplement: Supplementary file 5 [file DataSheet1.ZIP › 2 result graphs between the MSMS secondary fragments of each metabolite and the MSMS secondary fragments of the standard substance in the database/2-Deoxystreptamine.png]

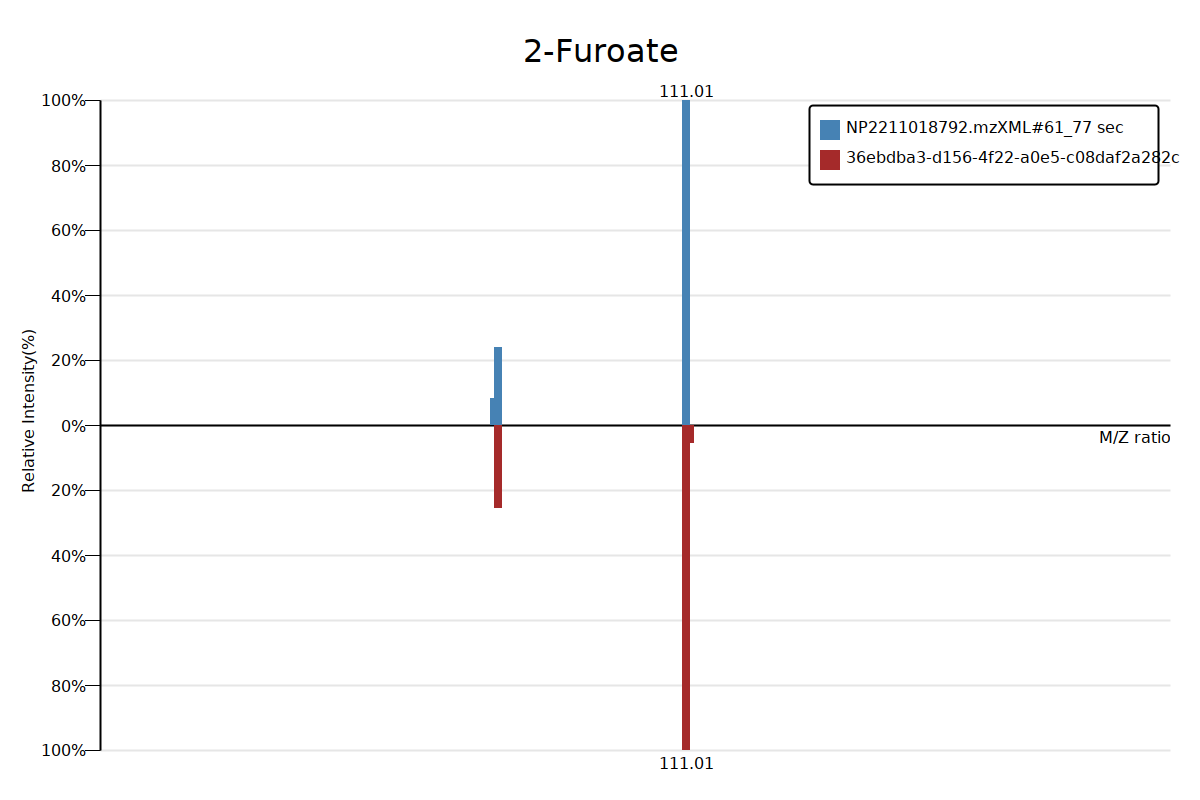

Supplement: Supplementary file 5 [file DataSheet1.ZIP › 2 result graphs between the MSMS secondary fragments of each metabolite and the MSMS secondary fragments of the standard substance in the database/2-Furoate.png]

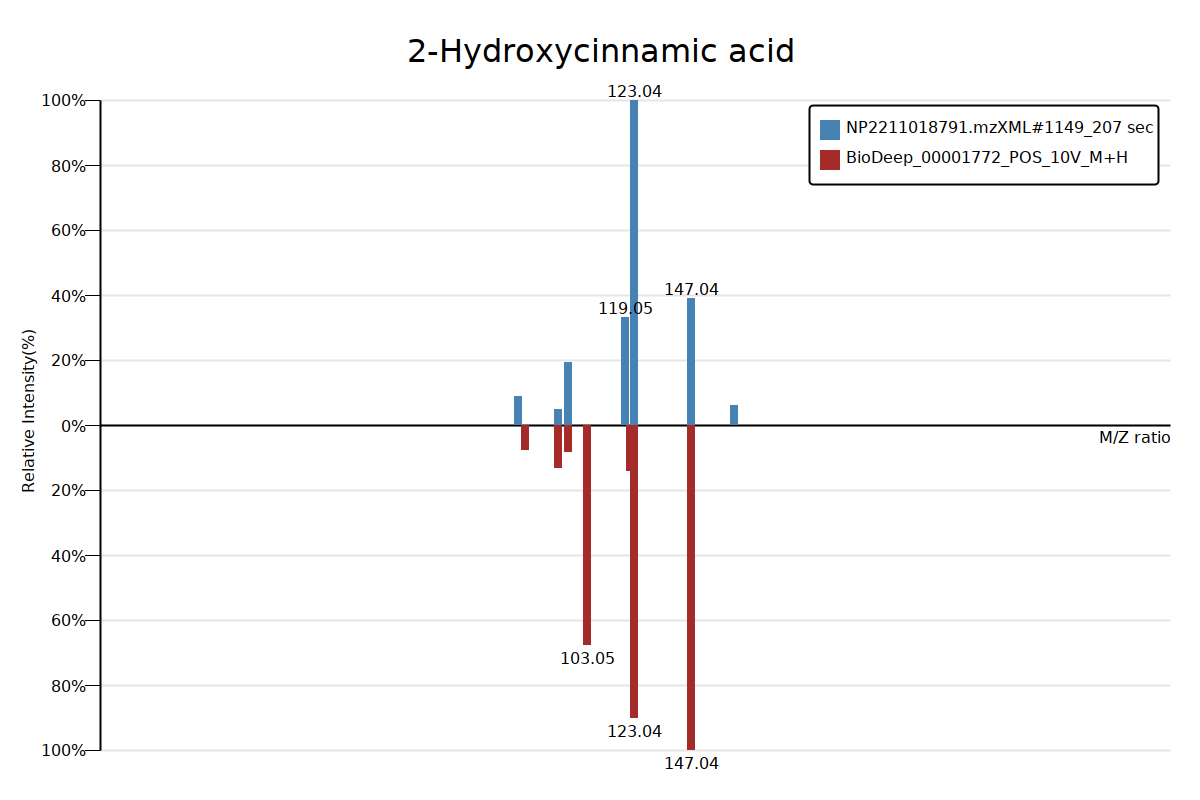

Supplement: Supplementary file 5 [file DataSheet1.ZIP › 2 result graphs between the MSMS secondary fragments of each metabolite and the MSMS secondary fragments of the standard substance in the database/2-Hydroxycinnamic acid.png]

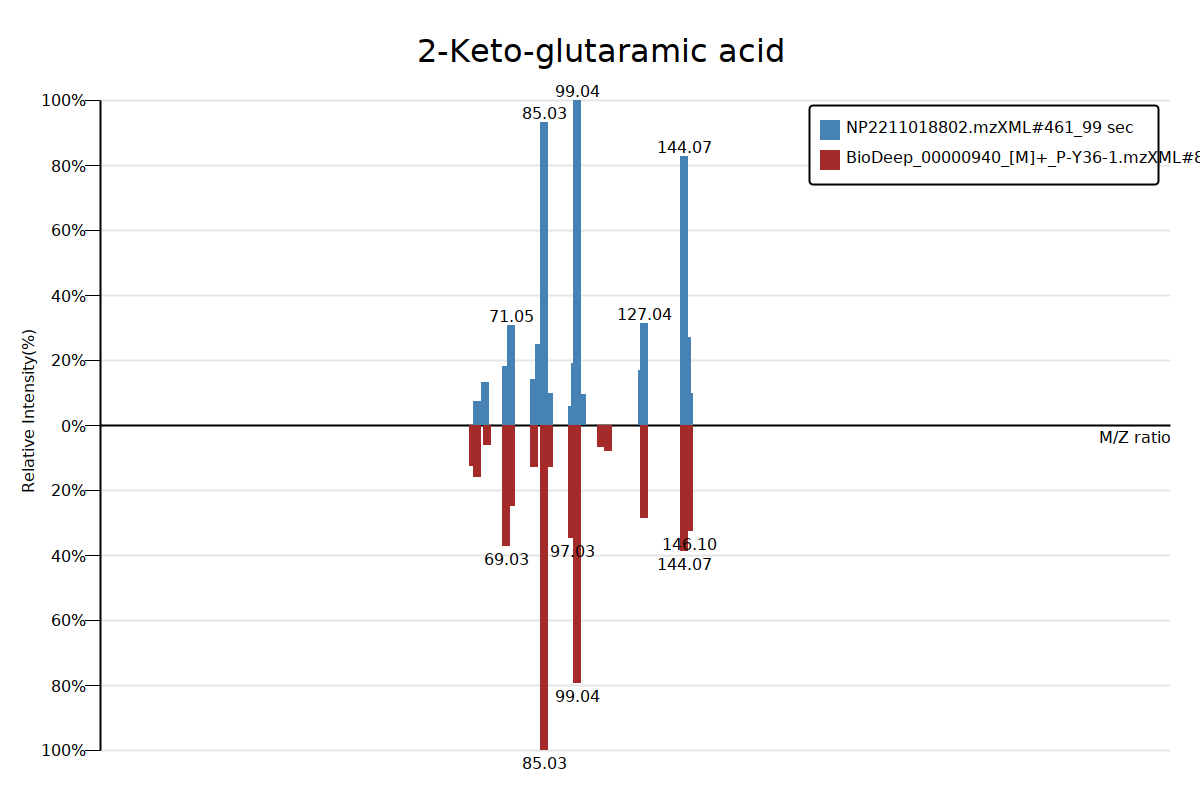

Supplement: Supplementary file 5 [file DataSheet1.ZIP › 2 result graphs between the MSMS secondary fragments of each metabolite and the MSMS secondary fragments of the standard substance in the database/2-Keto-glutaramic acid.png]

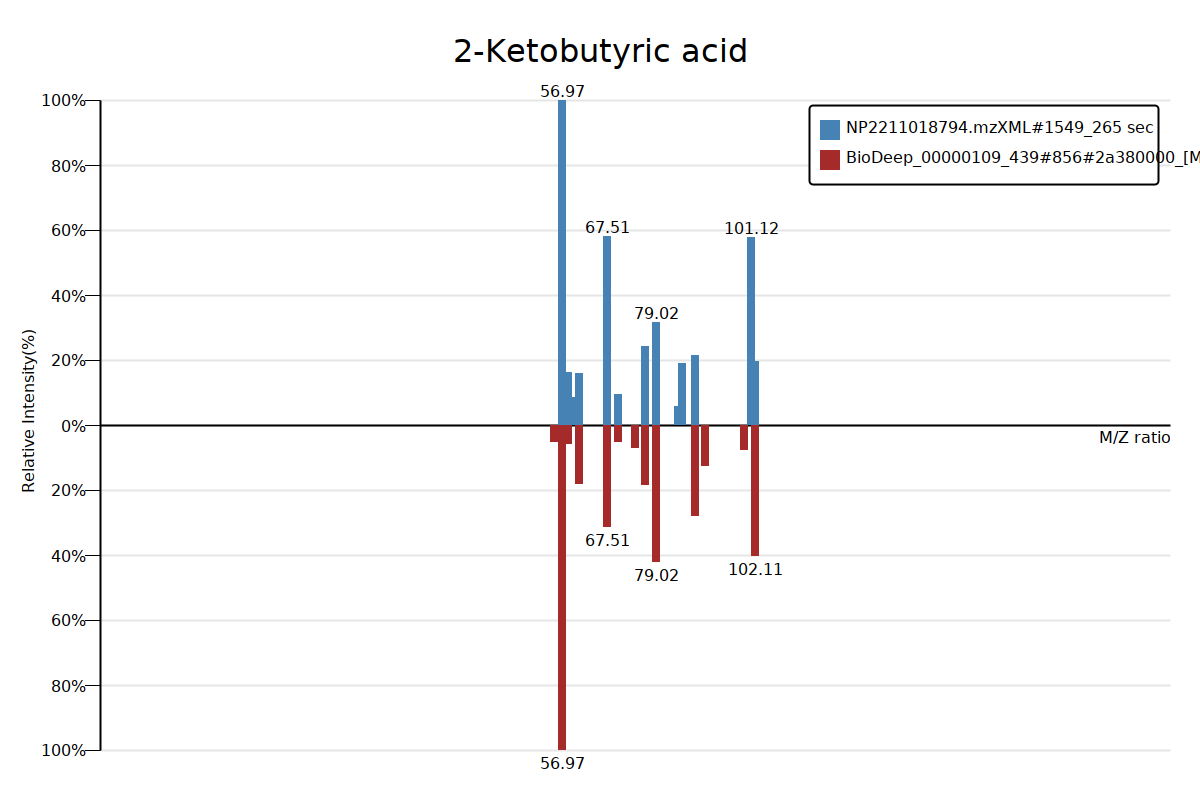

Supplement: Supplementary file 5 [file DataSheet1.ZIP › 2 result graphs between the MSMS secondary fragments of each metabolite and the MSMS secondary fragments of the standard substance in the database/2-Ketobutyric acid.png]

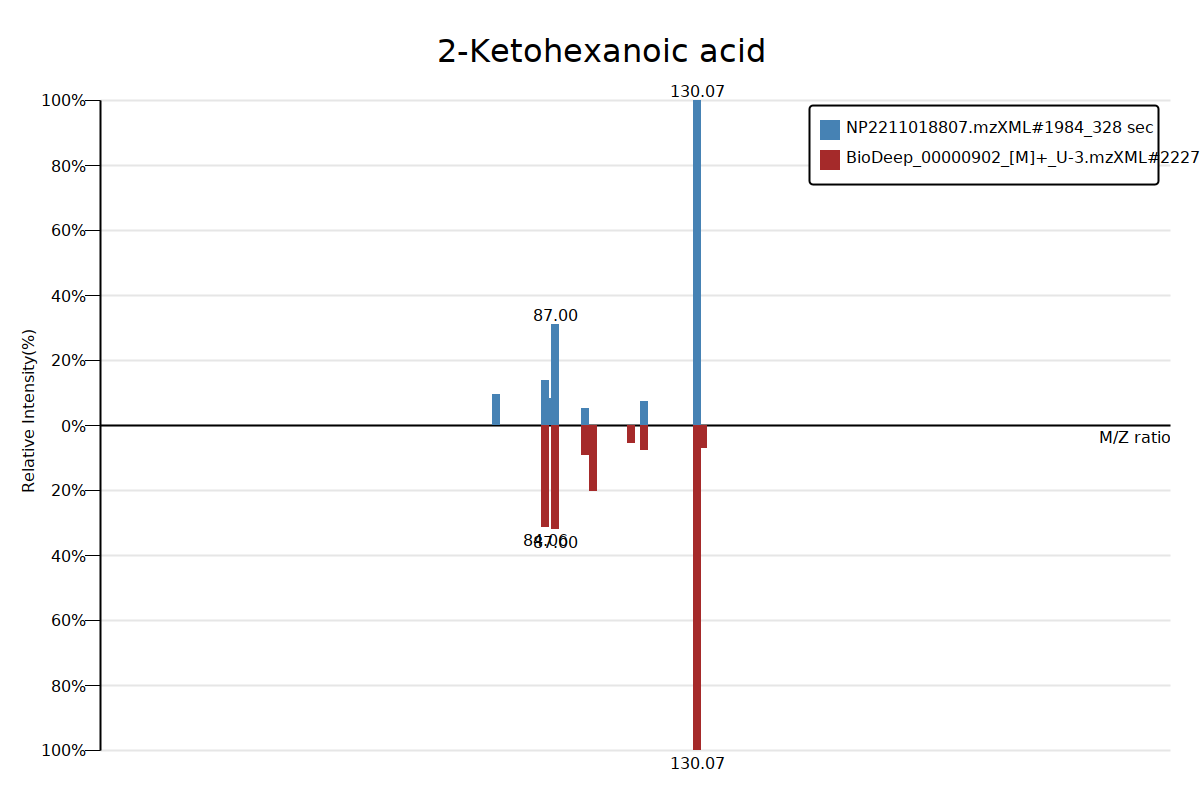

Supplement: Supplementary file 5 [file DataSheet1.ZIP › 2 result graphs between the MSMS secondary fragments of each metabolite and the MSMS secondary fragments of the standard substance in the database/2-Ketohexanoic acid.png]

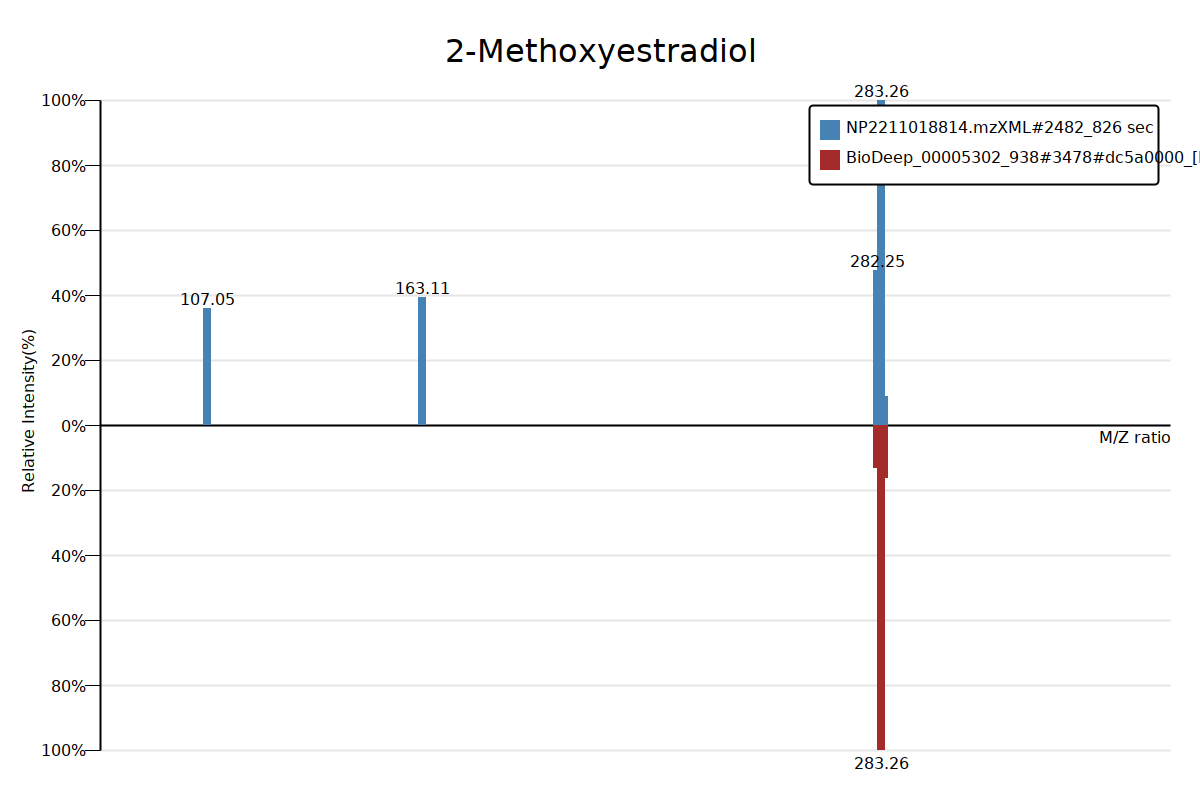

Supplement: Supplementary file 5 [file DataSheet1.ZIP › 2 result graphs between the MSMS secondary fragments of each metabolite and the MSMS secondary fragments of the standard substance in the database/2-Methoxyestradiol.png]

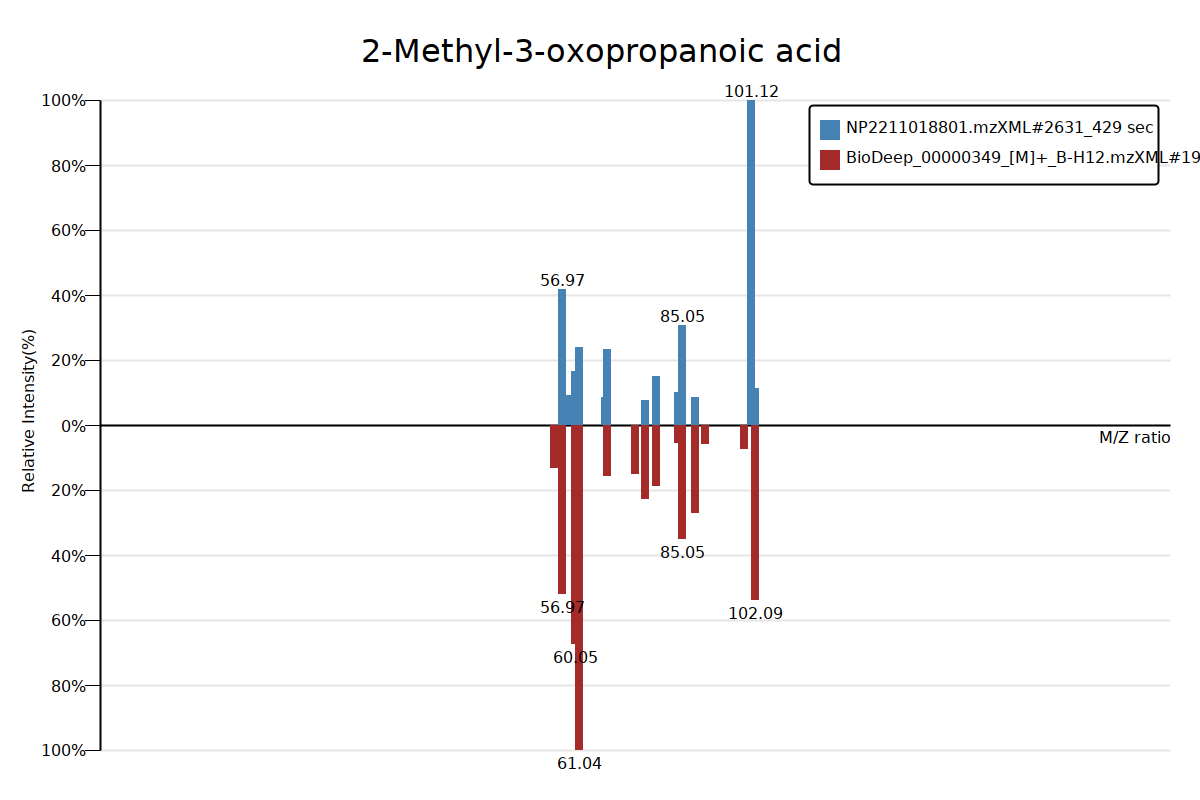

Supplement: Supplementary file 5 [file DataSheet1.ZIP › 2 result graphs between the MSMS secondary fragments of each metabolite and the MSMS secondary fragments of the standard substance in the database/2-Methyl-3-oxopropanoic acid.png]

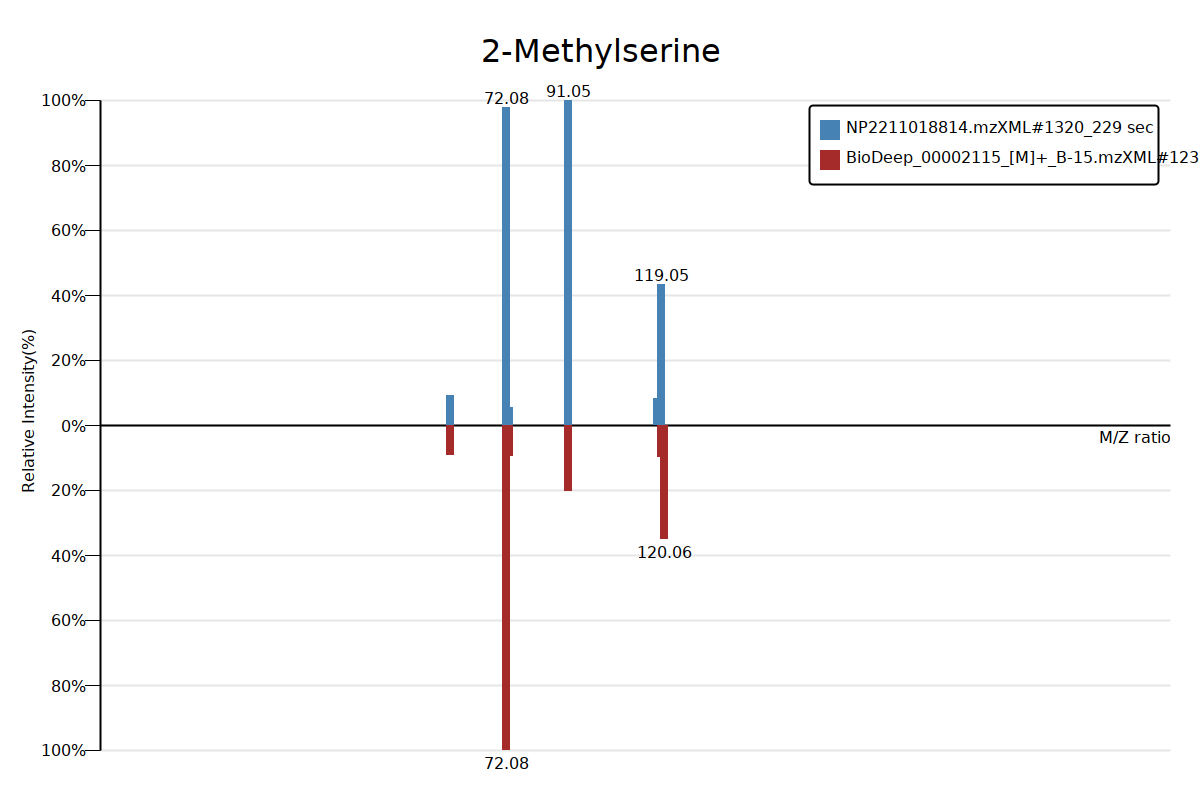

Supplement: Supplementary file 5 [file DataSheet1.ZIP › 2 result graphs between the MSMS secondary fragments of each metabolite and the MSMS secondary fragments of the standard substance in the database/2-Methylserine.png]
