# Supplementary figures and images for: LC-MS-based metabolomics reveals the mechanism of anti-gouty arthritis effect of Wuwei Shexiang pill (part 3 of 3)
Source: Front Pharmacol. 2023 Aug 11;14:1213602. doi: 10.3389/fphar.2023.1213602 (PMC10450745; doi:10.3389/fphar.2023.1213602)

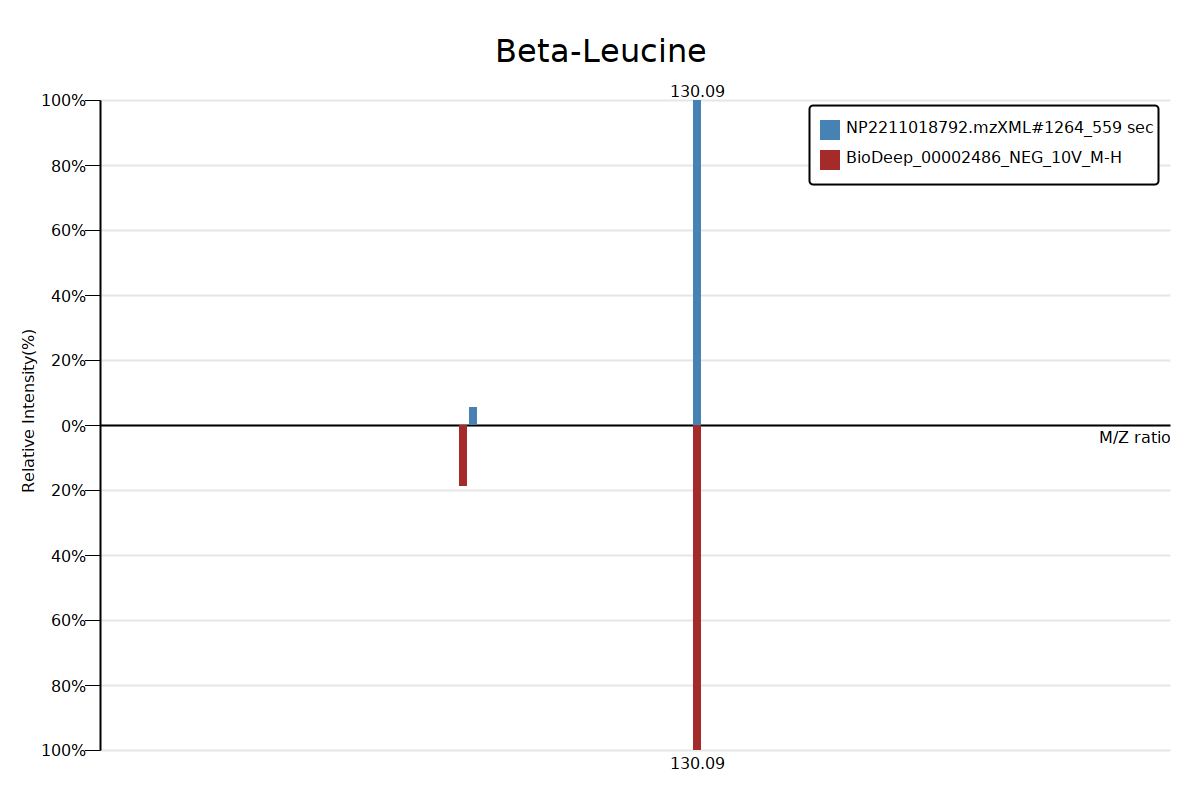

Supplement: Supplementary file 5 [file DataSheet1.ZIP › 2 result graphs between the MSMS secondary fragments of each metabolite and the MSMS secondary fragments of the standard substance in the database/Beta-Leucine.png]

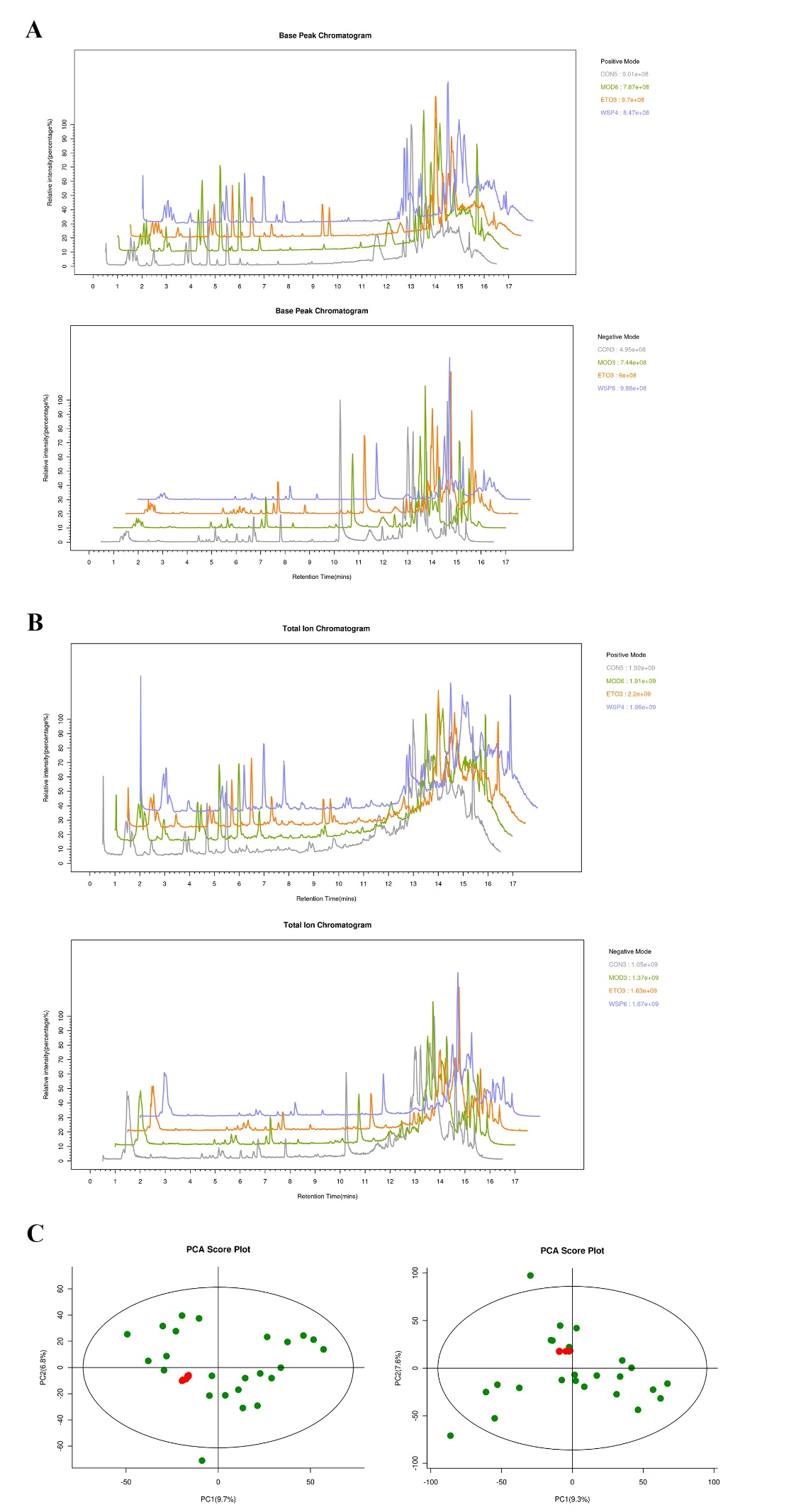


Supplementary figure 1 (A) Base peak chromatogram. (B) Total ion chromatogram. (C) QC samples.

Supplement: Supplementary file 6 [file DataSheet2.docx]
