# Supplementary figures and images for: LC-MS-based metabolomics reveals the mechanism of anti-gouty arthritis effect of Wuwei Shexiang pill (part 2 of 3)
Source: Front Pharmacol. 2023 Aug 11;14:1213602. doi: 10.3389/fphar.2023.1213602 (PMC10450745; doi:10.3389/fphar.2023.1213602)

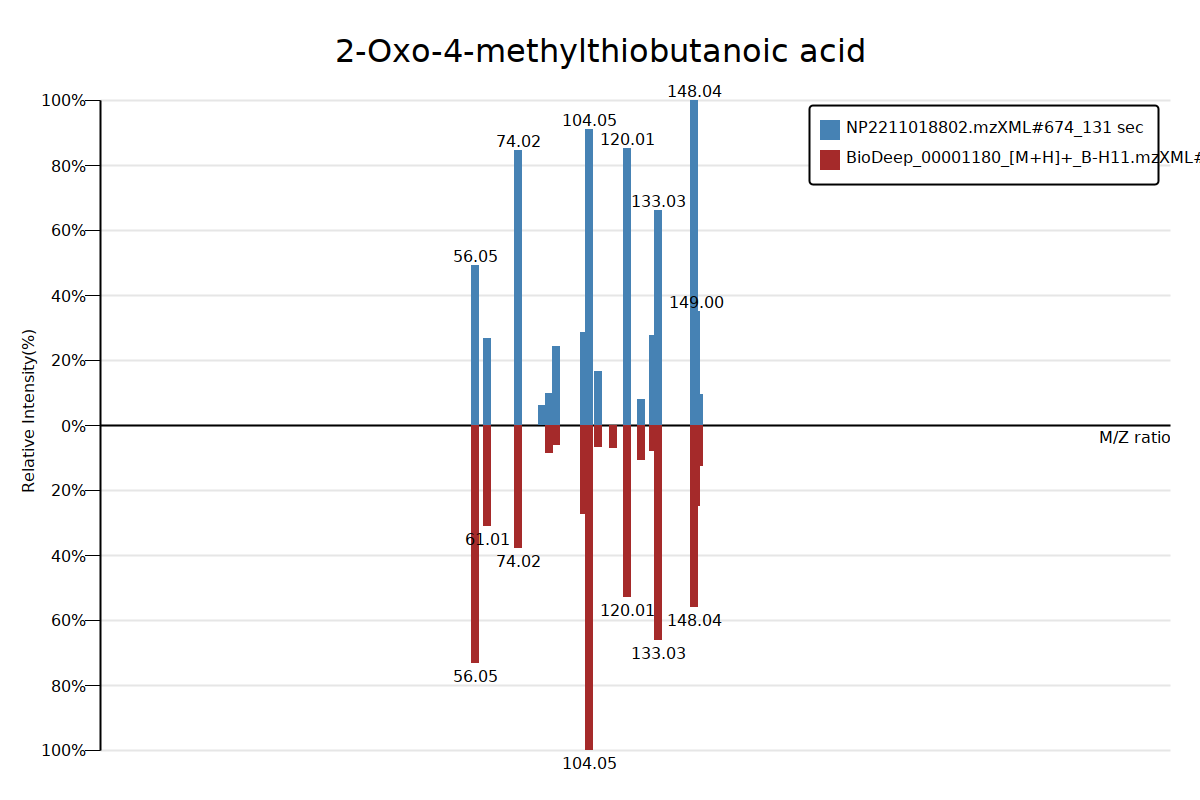

Supplement: Supplementary file 5 [file DataSheet1.ZIP › 2 result graphs between the MSMS secondary fragments of each metabolite and the MSMS secondary fragments of the standard substance in the database/2-Oxo-4-methylthiobutanoic acid.png]

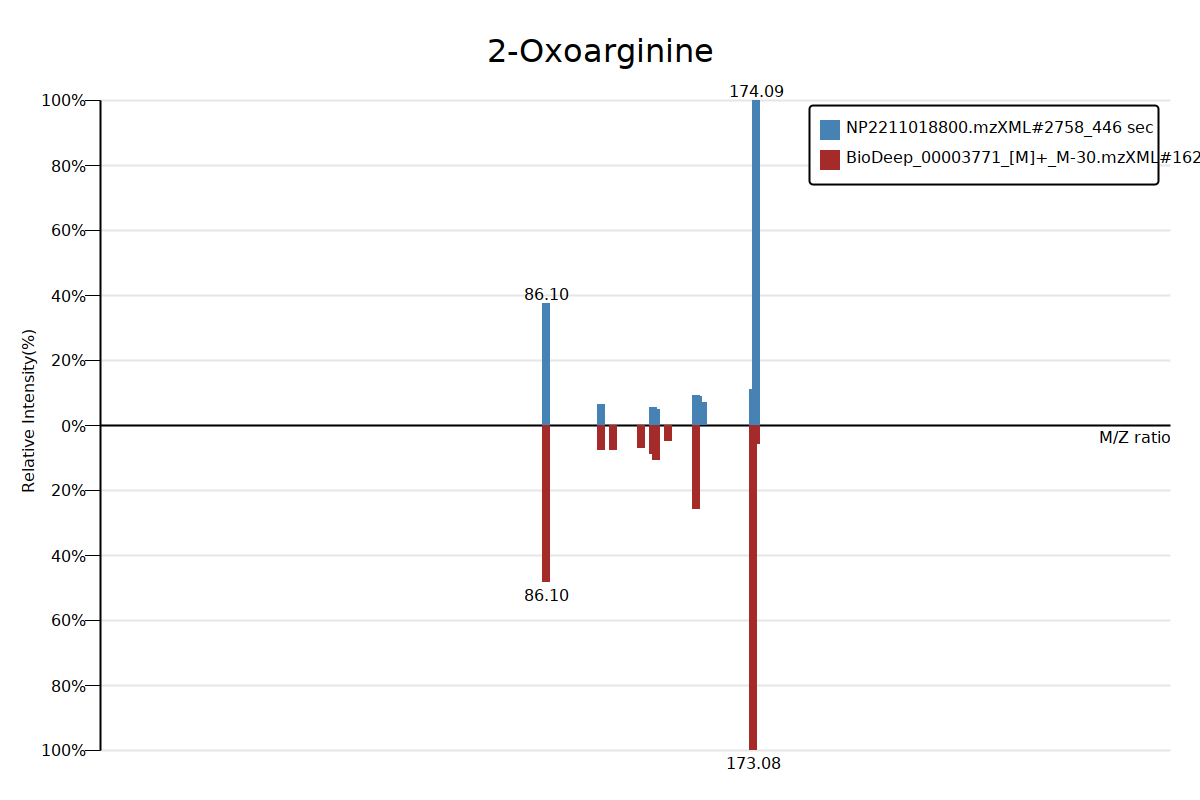

Supplement: Supplementary file 5 [file DataSheet1.ZIP › 2 result graphs between the MSMS secondary fragments of each metabolite and the MSMS secondary fragments of the standard substance in the database/2-Oxoarginine.png]

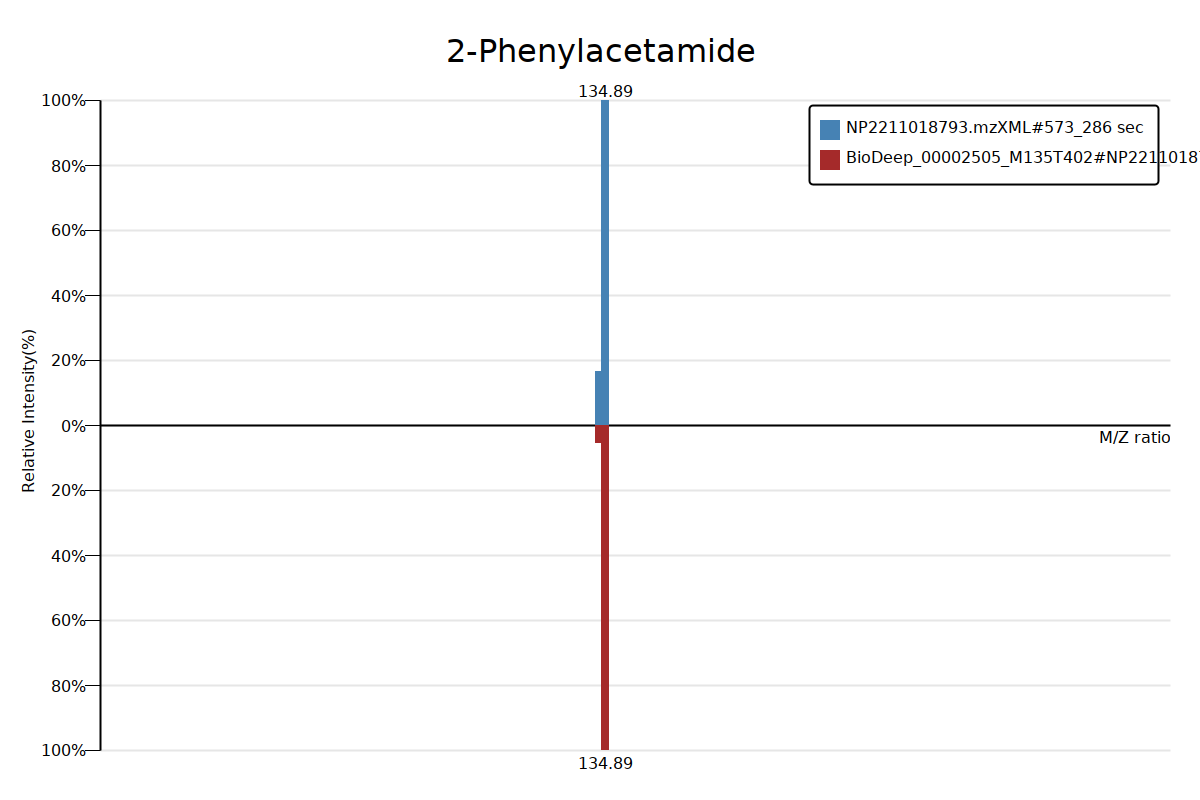

Supplement: Supplementary file 5 [file DataSheet1.ZIP › 2 result graphs between the MSMS secondary fragments of each metabolite and the MSMS secondary fragments of the standard substance in the database/2-Phenylacetamide.png]

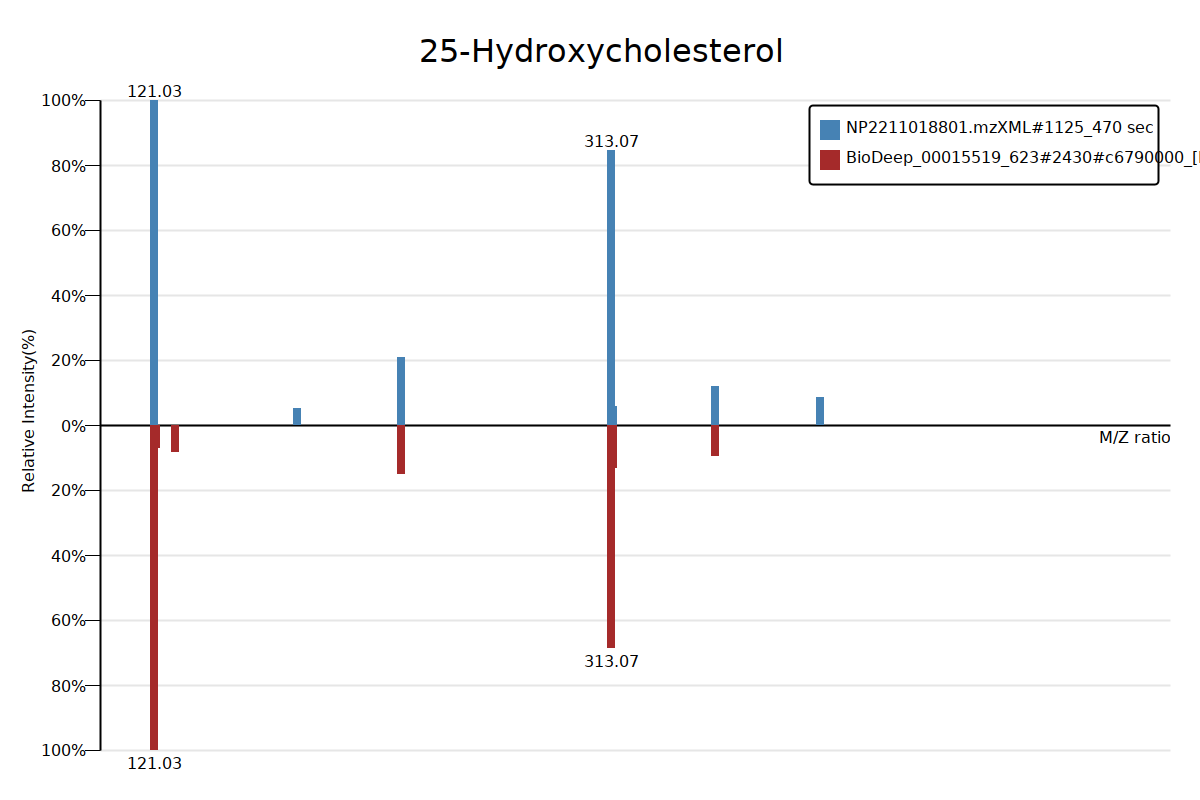

Supplement: Supplementary file 5 [file DataSheet1.ZIP › 2 result graphs between the MSMS secondary fragments of each metabolite and the MSMS secondary fragments of the standard substance in the database/25-Hydroxycholesterol.png]

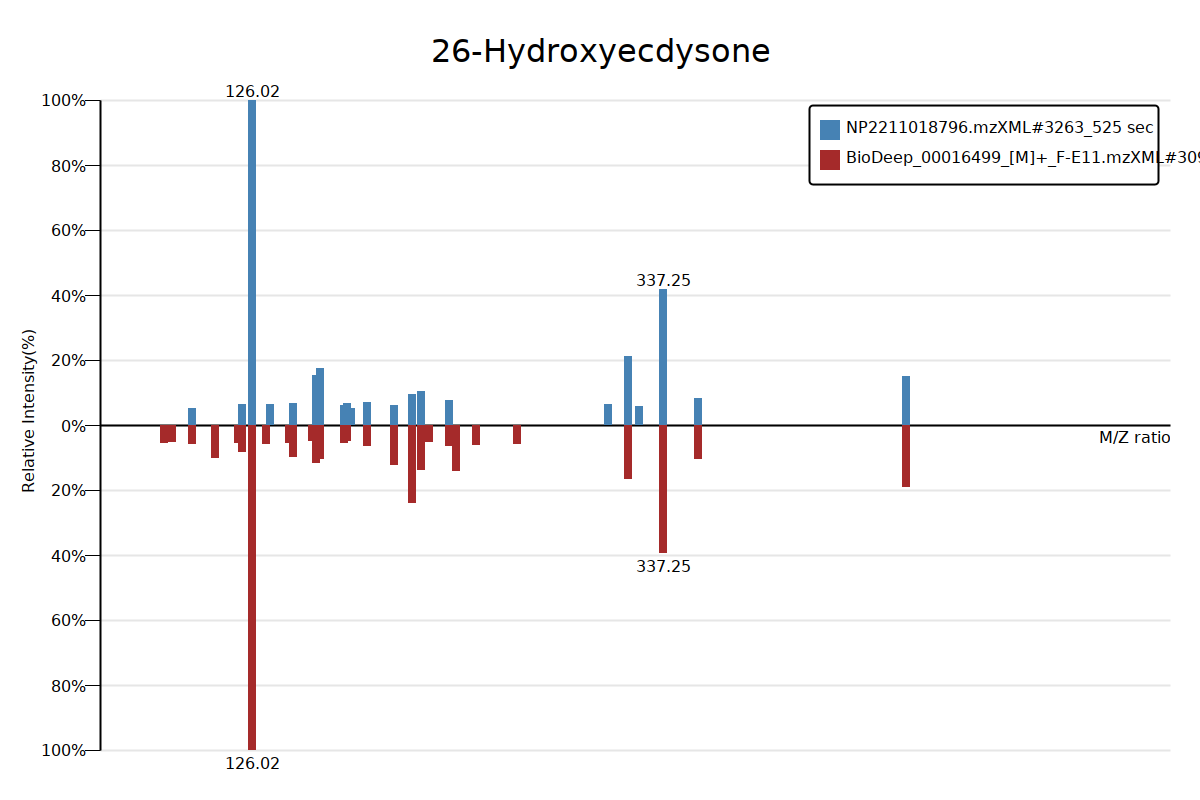

Supplement: Supplementary file 5 [file DataSheet1.ZIP › 2 result graphs between the MSMS secondary fragments of each metabolite and the MSMS secondary fragments of the standard substance in the database/26-Hydroxyecdysone.png]

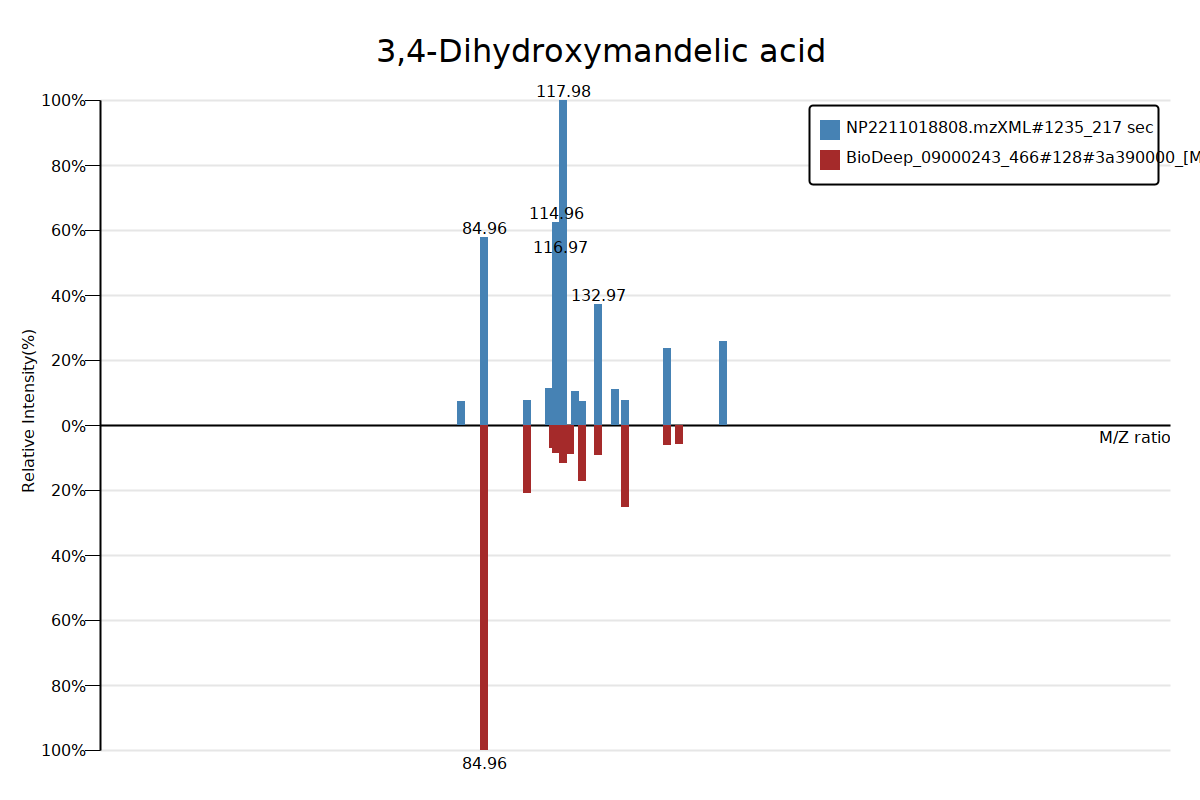

Supplement: Supplementary file 5 [file DataSheet1.ZIP › 2 result graphs between the MSMS secondary fragments of each metabolite and the MSMS secondary fragments of the standard substance in the database/3,4-Dihydroxymandelic acid.png]

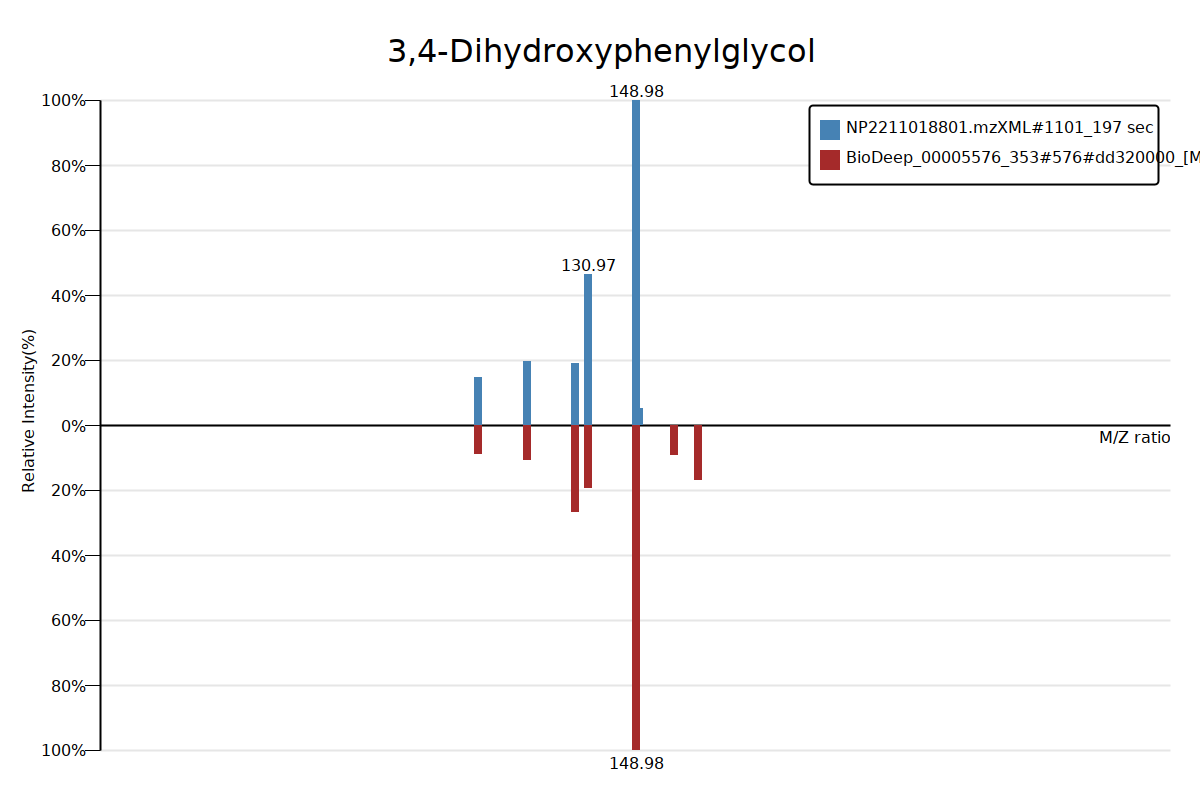

Supplement: Supplementary file 5 [file DataSheet1.ZIP › 2 result graphs between the MSMS secondary fragments of each metabolite and the MSMS secondary fragments of the standard substance in the database/3,4-Dihydroxyphenylglycol.png]

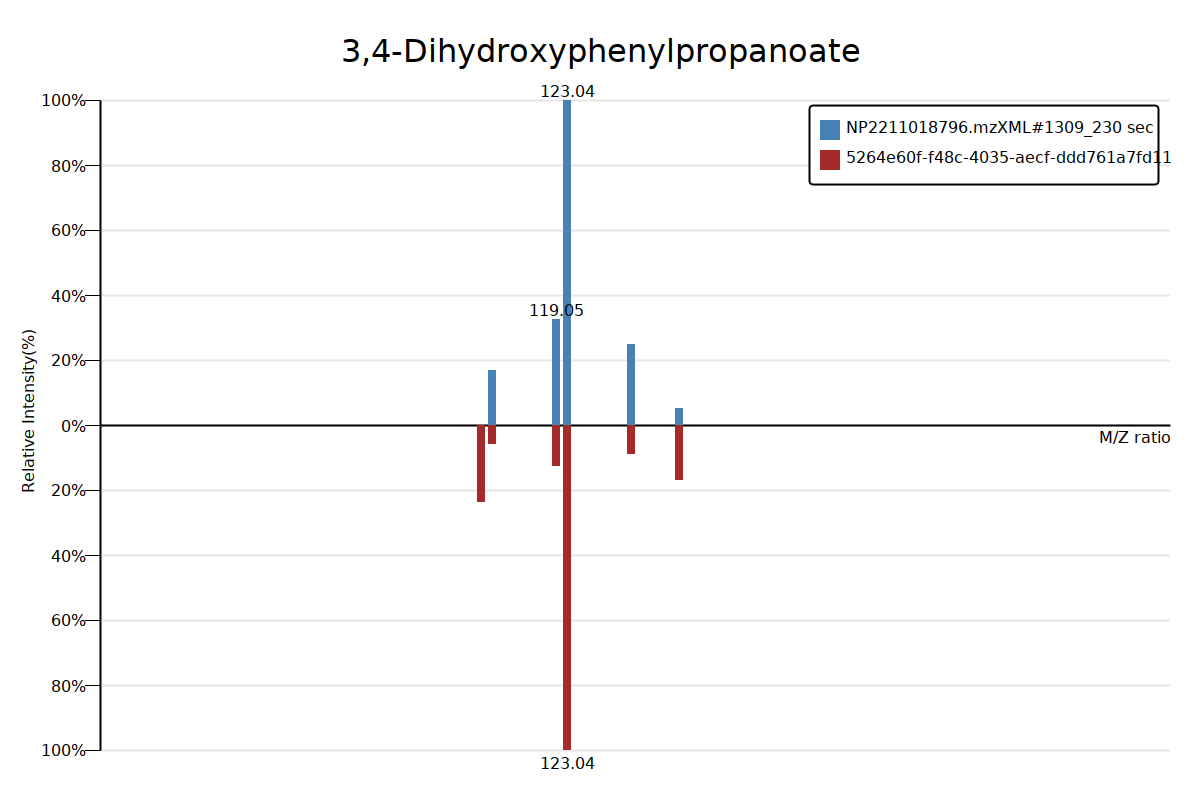

Supplement: Supplementary file 5 [file DataSheet1.ZIP › 2 result graphs between the MSMS secondary fragments of each metabolite and the MSMS secondary fragments of the standard substance in the database/3,4-Dihydroxyphenylpropanoate.png]

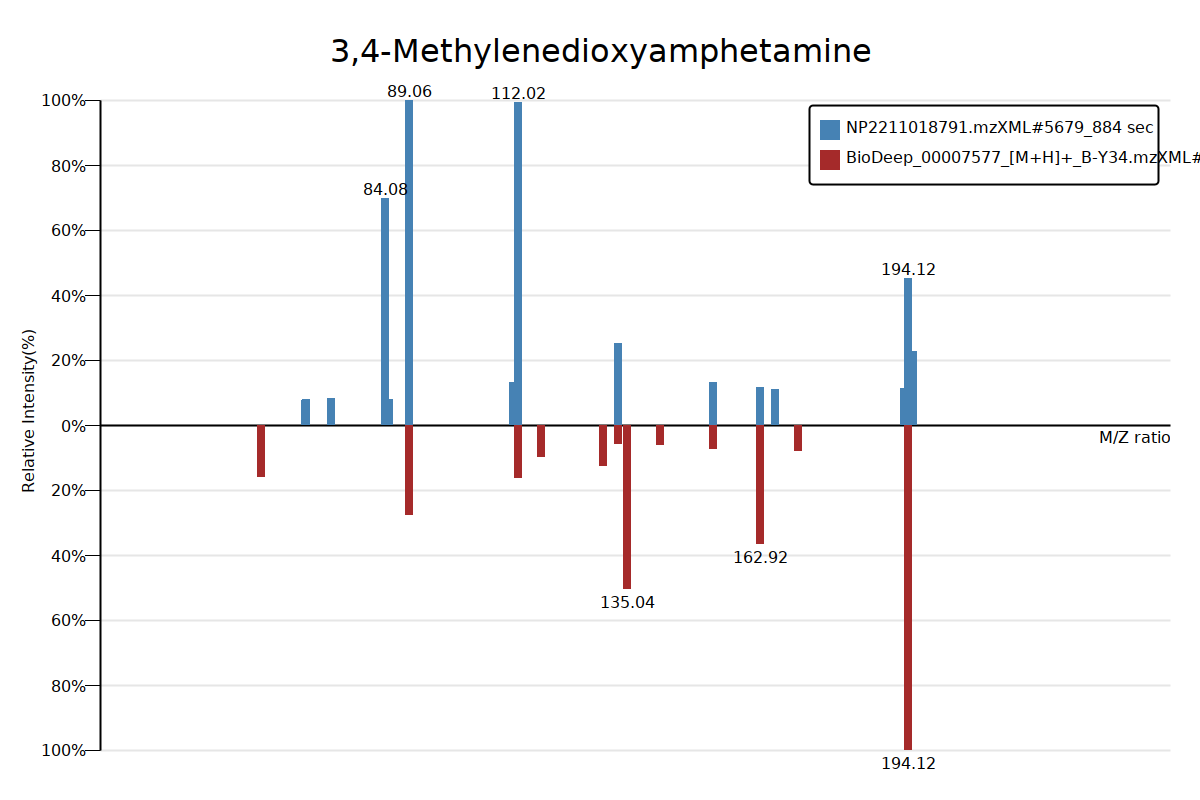

Supplement: Supplementary file 5 [file DataSheet1.ZIP › 2 result graphs between the MSMS secondary fragments of each metabolite and the MSMS secondary fragments of the standard substance in the database/3,4-Methylenedioxyamphetamine.png]

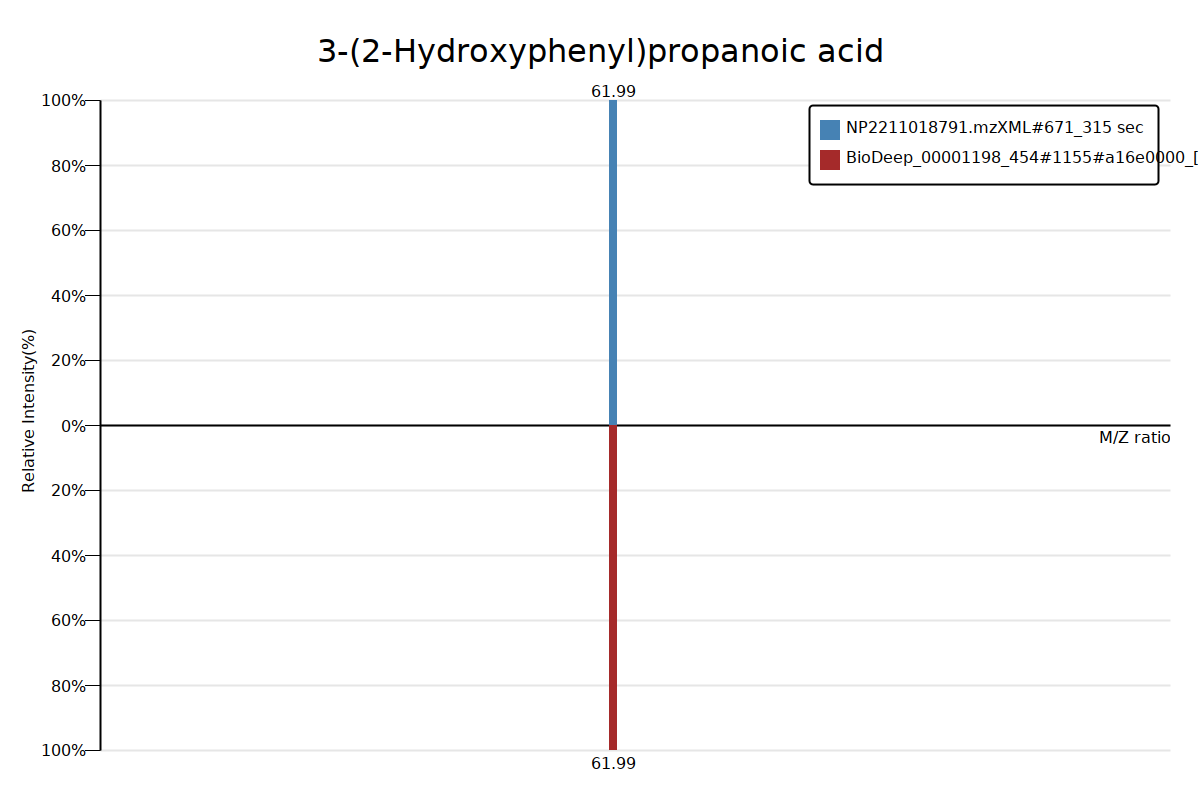

Supplement: Supplementary file 5 [file DataSheet1.ZIP › 2 result graphs between the MSMS secondary fragments of each metabolite and the MSMS secondary fragments of the standard substance in the database/3-(2-Hydroxyphenyl)propanoic acid.png]

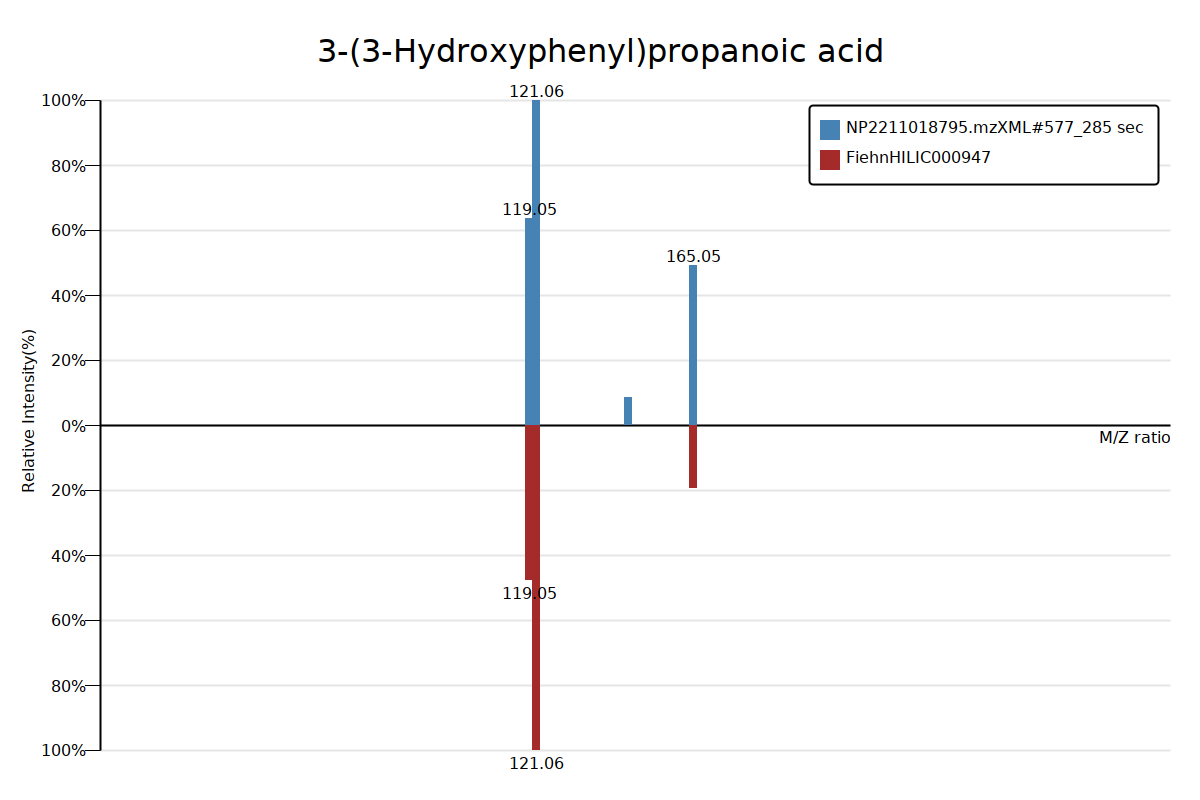

Supplement: Supplementary file 5 [file DataSheet1.ZIP › 2 result graphs between the MSMS secondary fragments of each metabolite and the MSMS secondary fragments of the standard substance in the database/3-(3-Hydroxyphenyl)propanoic acid.png]

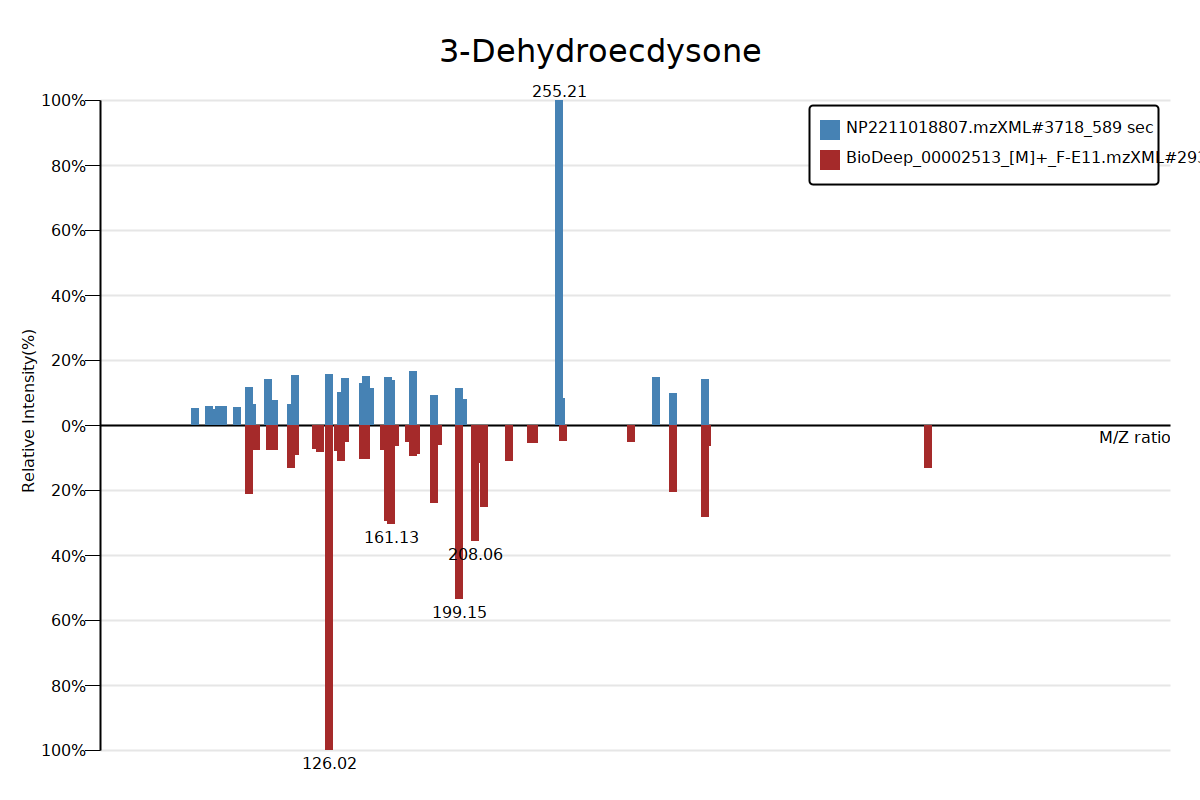

Supplement: Supplementary file 5 [file DataSheet1.ZIP › 2 result graphs between the MSMS secondary fragments of each metabolite and the MSMS secondary fragments of the standard substance in the database/3-Dehydroecdysone.png]

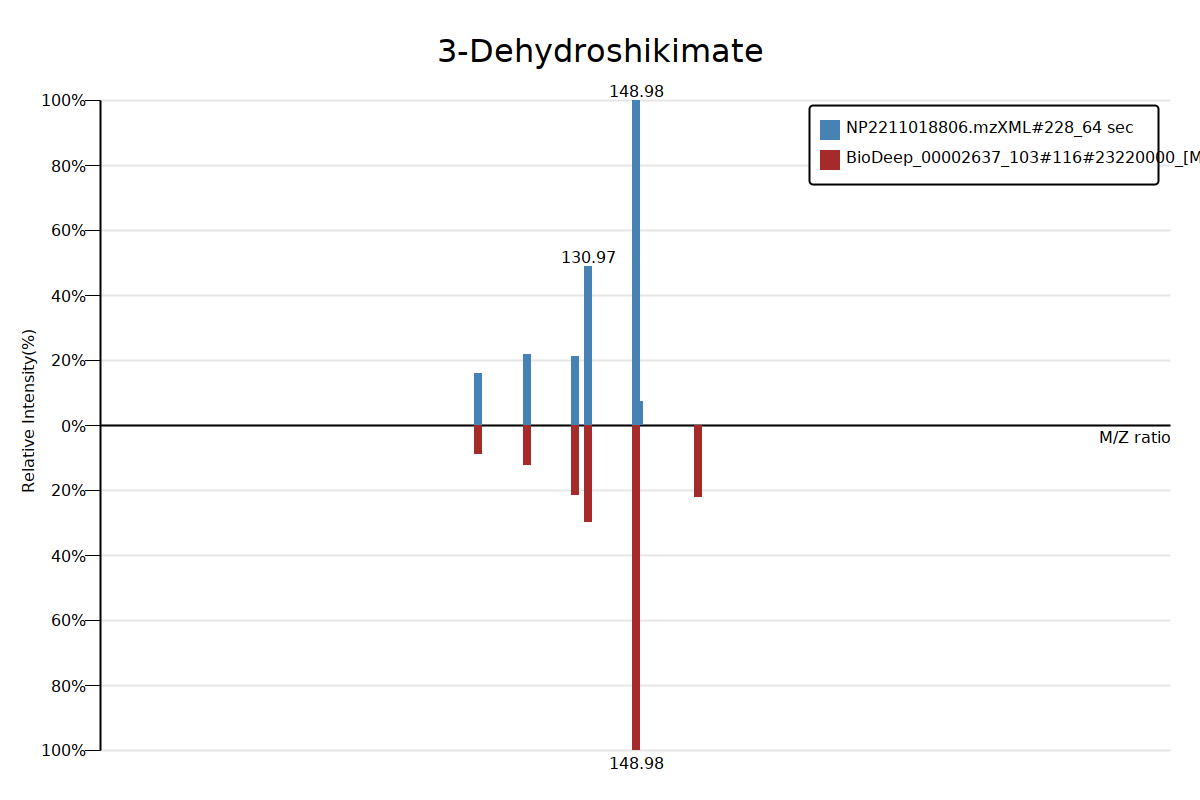

Supplement: Supplementary file 5 [file DataSheet1.ZIP › 2 result graphs between the MSMS secondary fragments of each metabolite and the MSMS secondary fragments of the standard substance in the database/3-Dehydroshikimate.png]

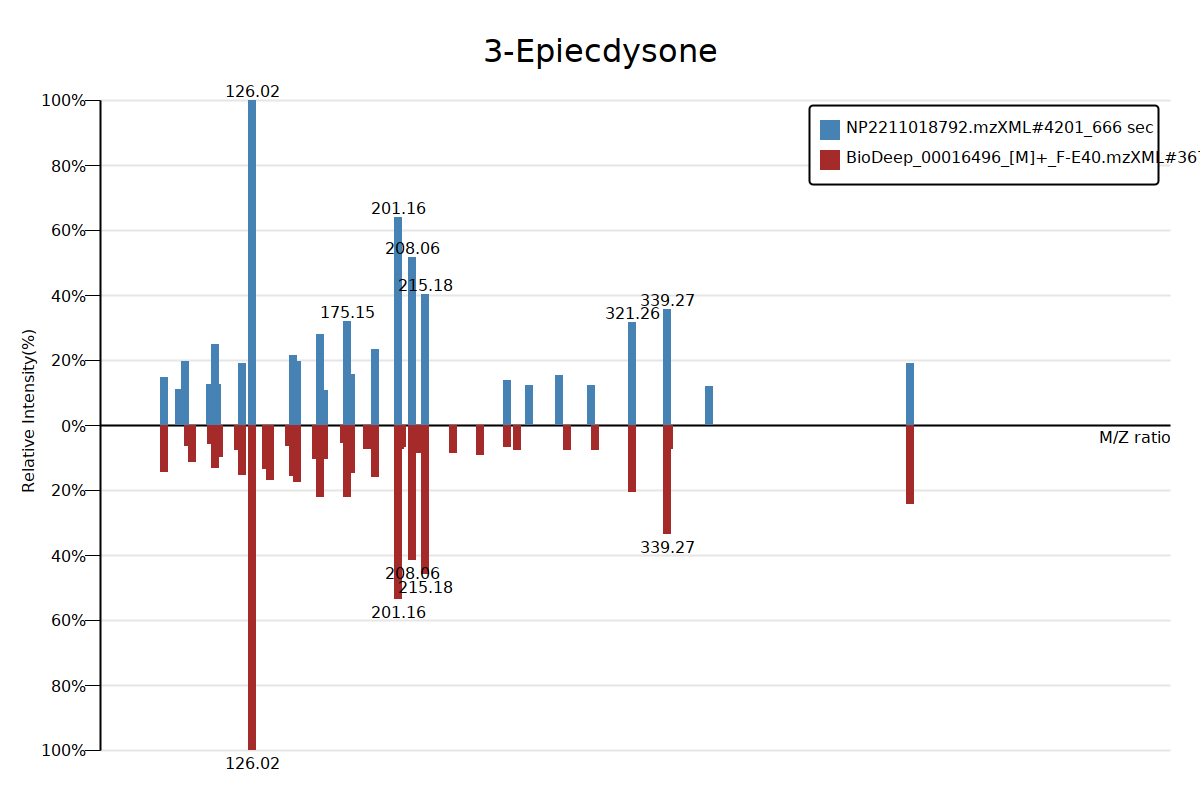

Supplement: Supplementary file 5 [file DataSheet1.ZIP › 2 result graphs between the MSMS secondary fragments of each metabolite and the MSMS secondary fragments of the standard substance in the database/3-Epiecdysone.png]

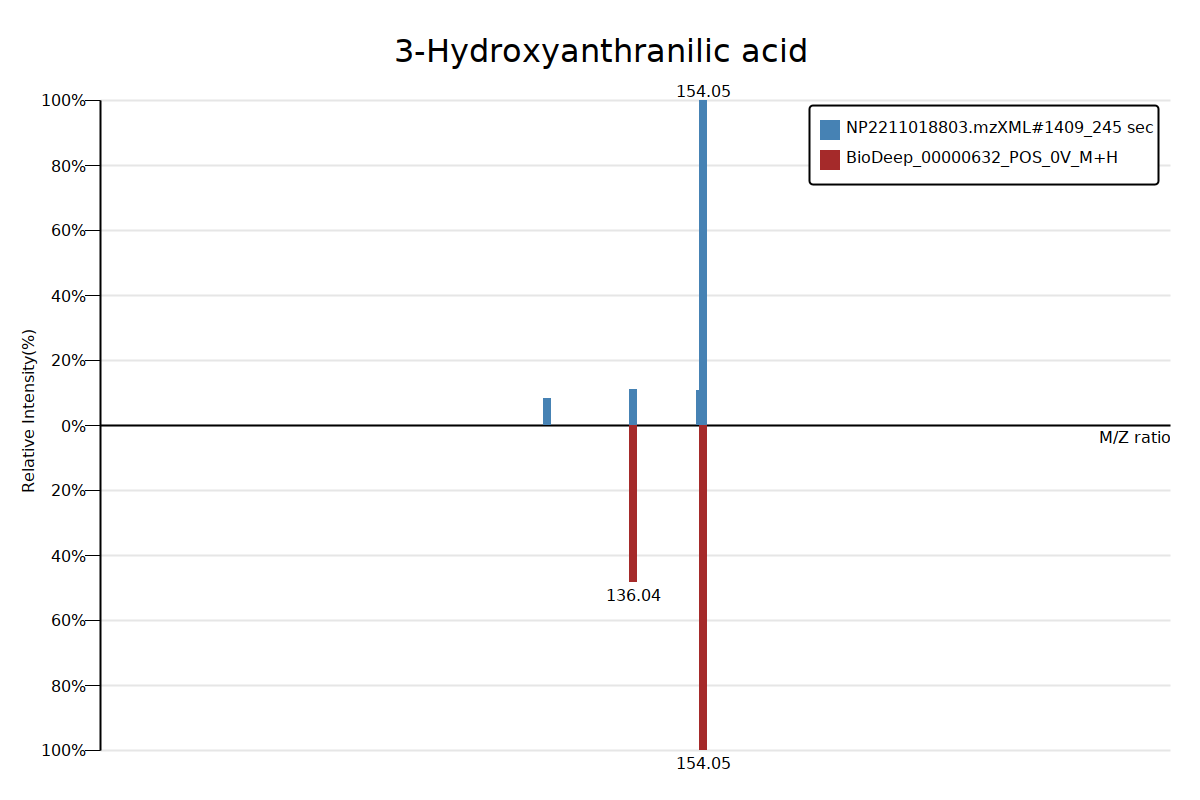

Supplement: Supplementary file 5 [file DataSheet1.ZIP › 2 result graphs between the MSMS secondary fragments of each metabolite and the MSMS secondary fragments of the standard substance in the database/3-Hydroxyanthranilic acid.png]

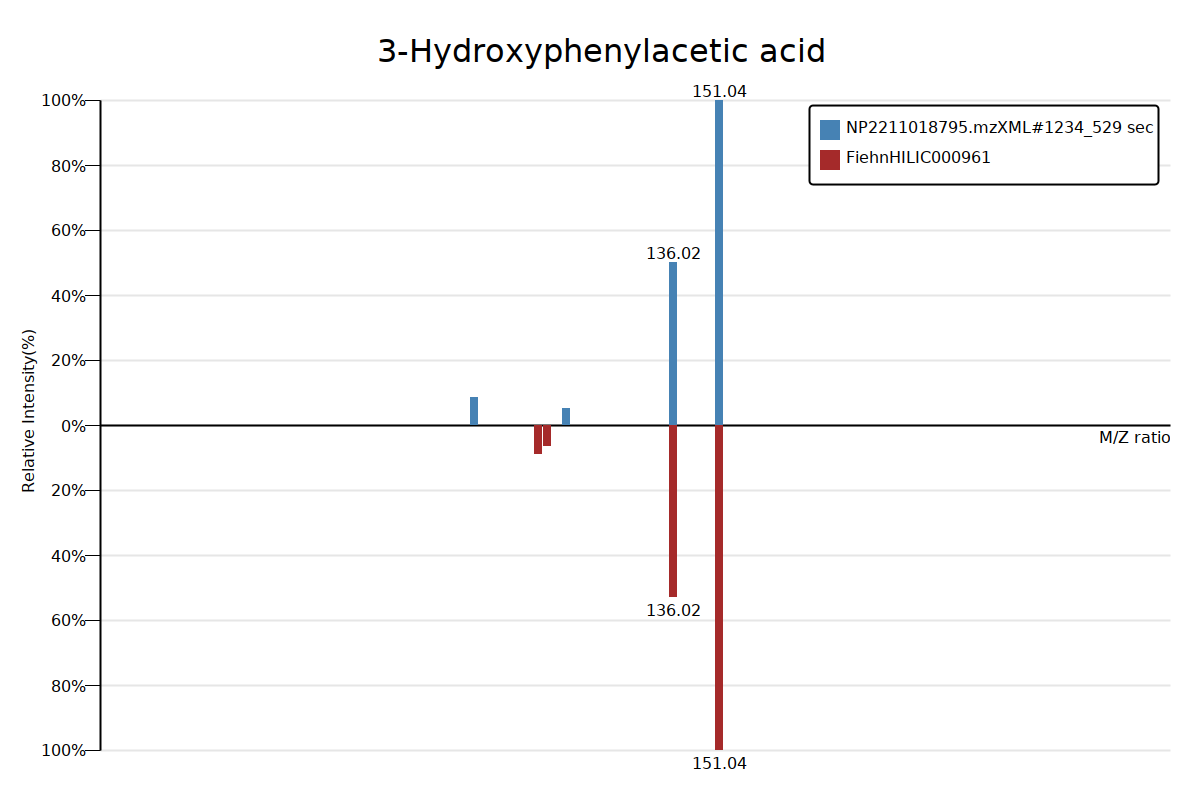

Supplement: Supplementary file 5 [file DataSheet1.ZIP › 2 result graphs between the MSMS secondary fragments of each metabolite and the MSMS secondary fragments of the standard substance in the database/3-Hydroxyphenylacetic acid.png]

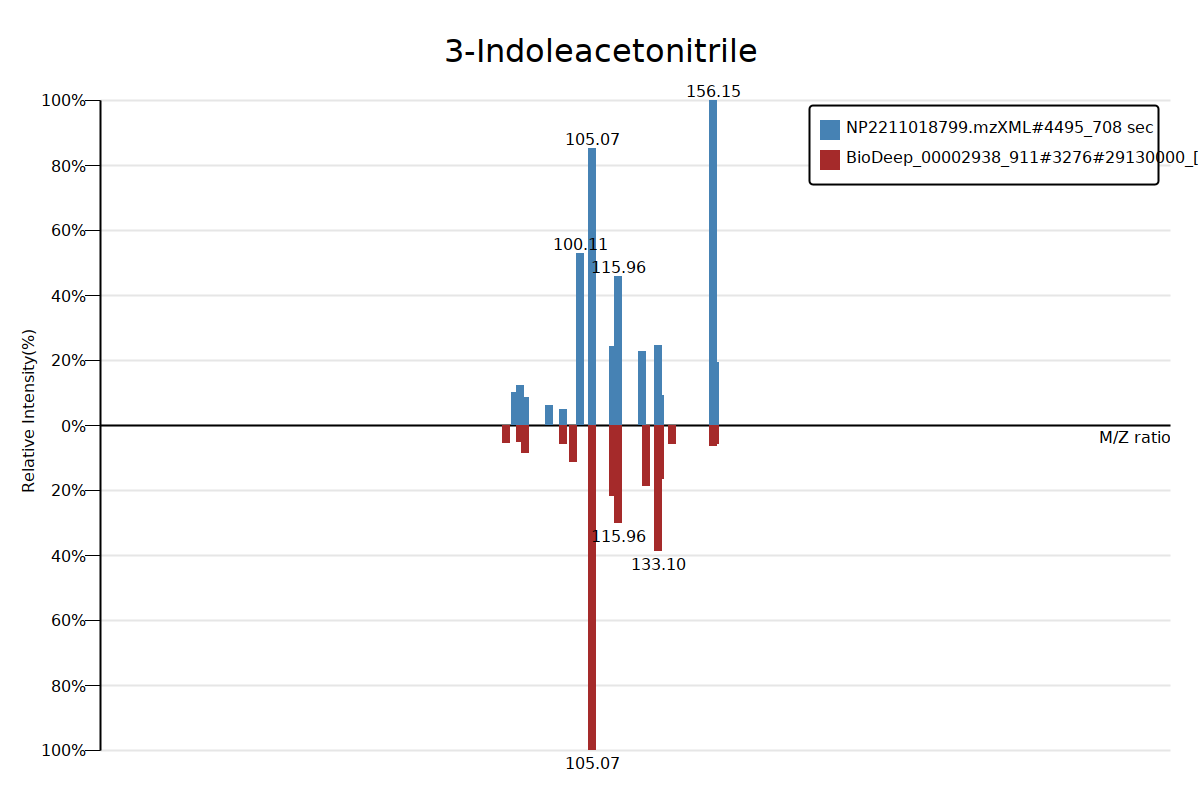

Supplement: Supplementary file 5 [file DataSheet1.ZIP › 2 result graphs between the MSMS secondary fragments of each metabolite and the MSMS secondary fragments of the standard substance in the database/3-Indoleacetonitrile.png]

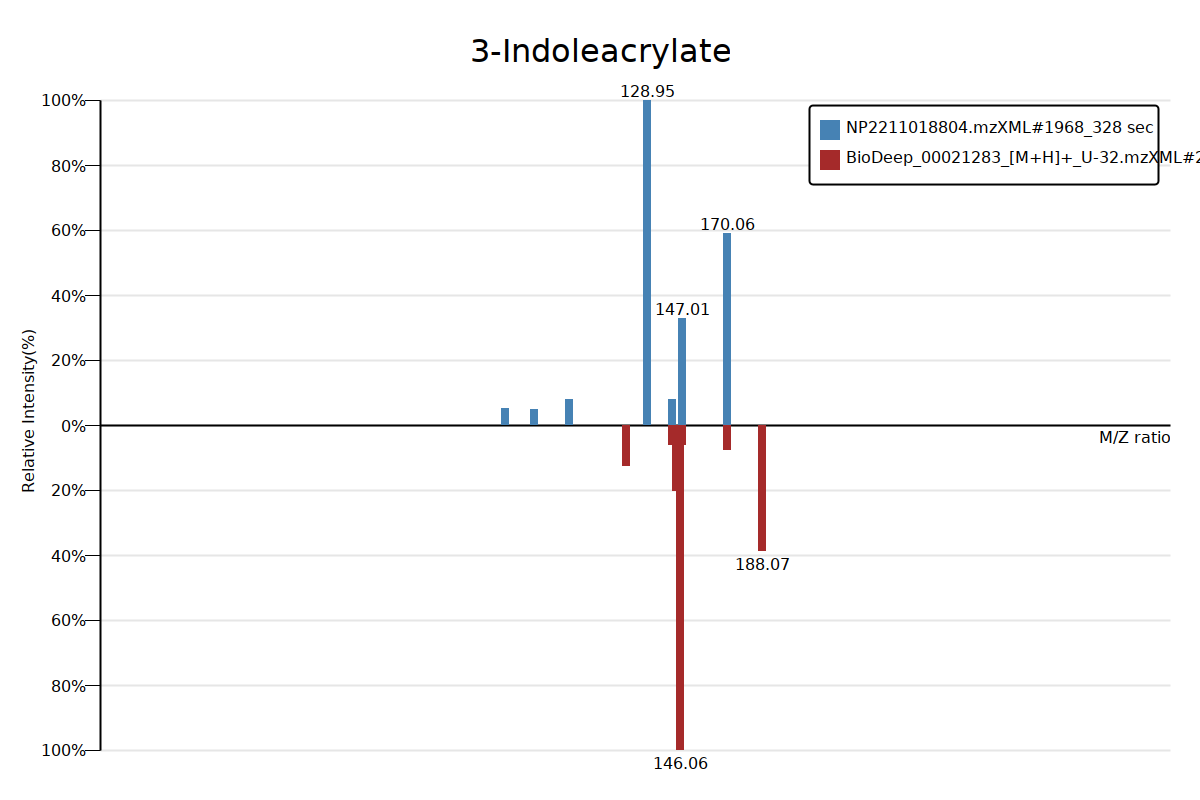

Supplement: Supplementary file 5 [file DataSheet1.ZIP › 2 result graphs between the MSMS secondary fragments of each metabolite and the MSMS secondary fragments of the standard substance in the database/3-Indoleacrylate.png]

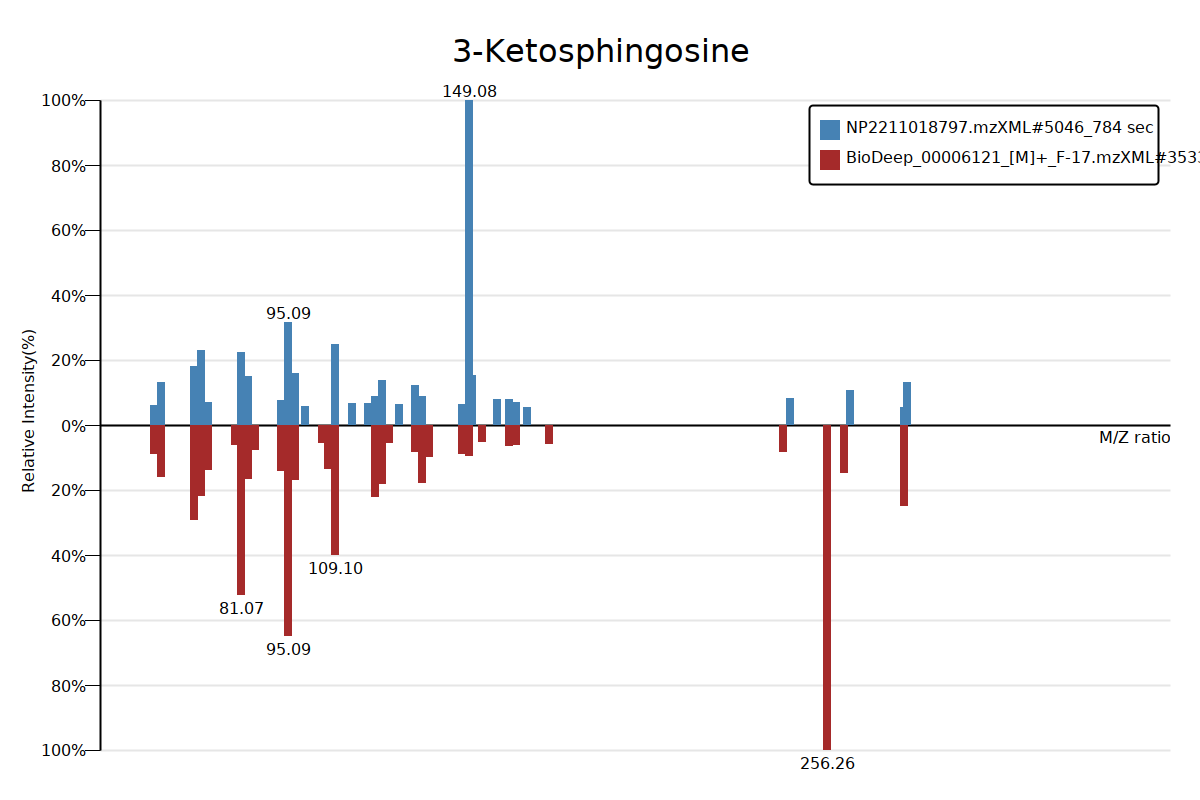

Supplement: Supplementary file 5 [file DataSheet1.ZIP › 2 result graphs between the MSMS secondary fragments of each metabolite and the MSMS secondary fragments of the standard substance in the database/3-Ketosphingosine.png]

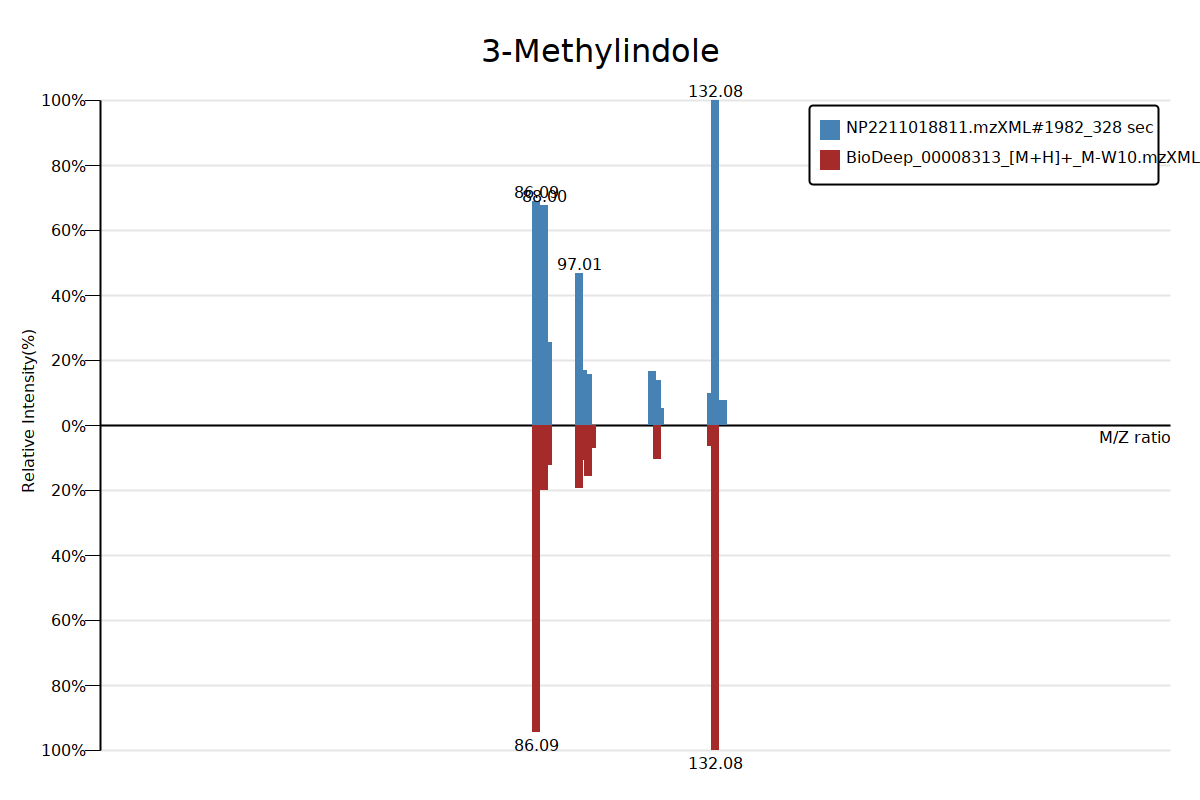

Supplement: Supplementary file 5 [file DataSheet1.ZIP › 2 result graphs between the MSMS secondary fragments of each metabolite and the MSMS secondary fragments of the standard substance in the database/3-Methylindole.png]

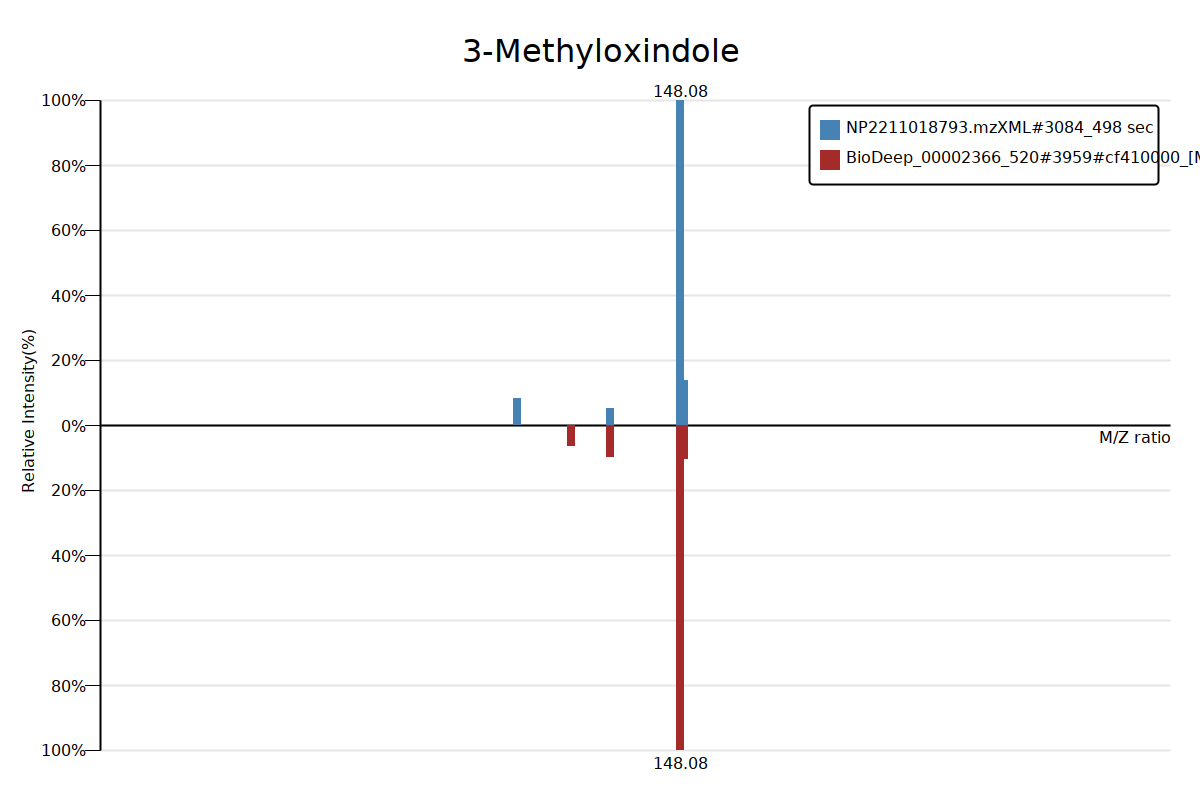

Supplement: Supplementary file 5 [file DataSheet1.ZIP › 2 result graphs between the MSMS secondary fragments of each metabolite and the MSMS secondary fragments of the standard substance in the database/3-Methyloxindole.png]

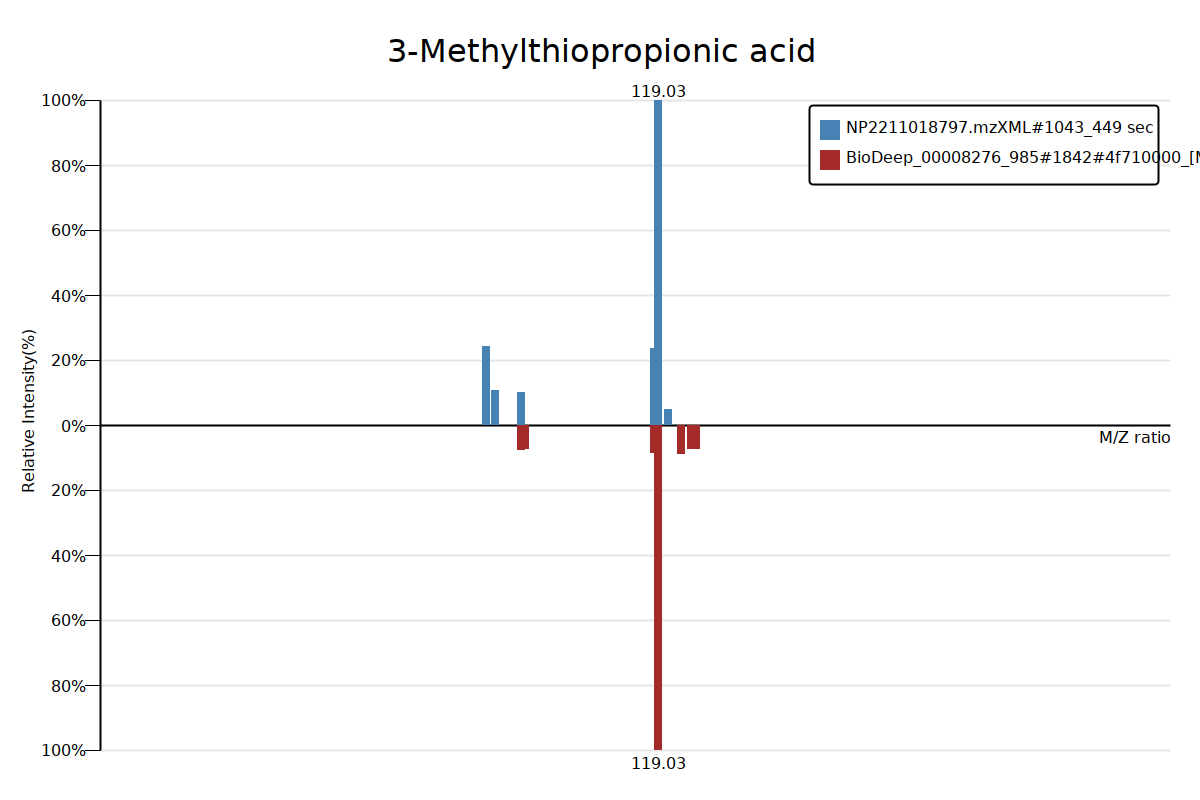

Supplement: Supplementary file 5 [file DataSheet1.ZIP › 2 result graphs between the MSMS secondary fragments of each metabolite and the MSMS secondary fragments of the standard substance in the database/3-Methylthiopropionic acid.png]

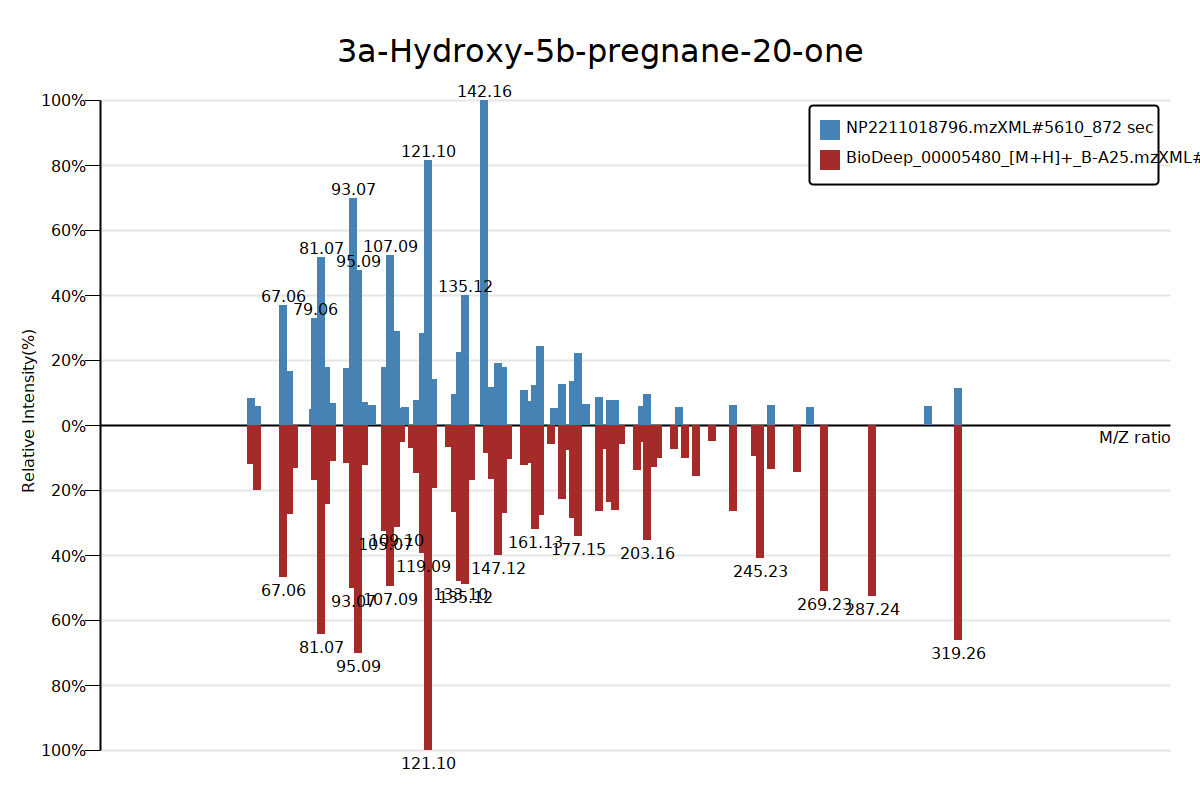

Supplement: Supplementary file 5 [file DataSheet1.ZIP › 2 result graphs between the MSMS secondary fragments of each metabolite and the MSMS secondary fragments of the standard substance in the database/3a-Hydroxy-5b-pregnane-20-one.png]

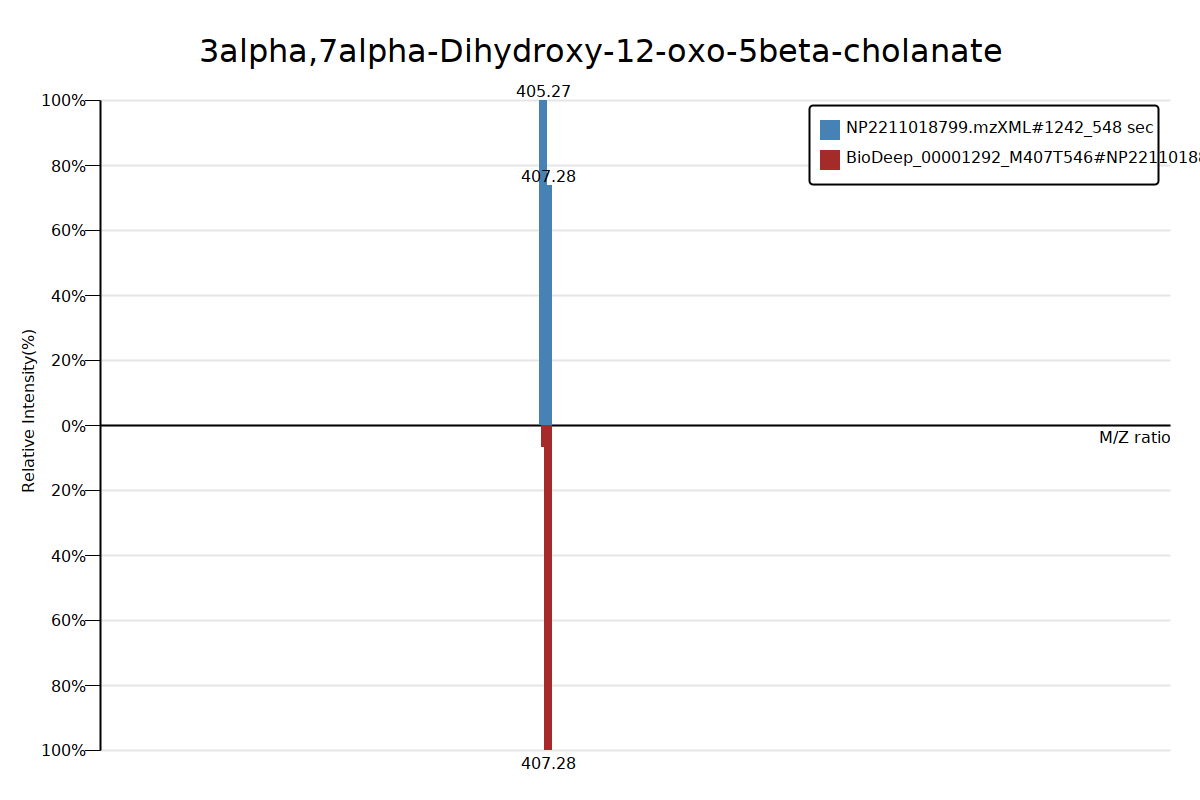

Supplement: Supplementary file 5 [file DataSheet1.ZIP › 2 result graphs between the MSMS secondary fragments of each metabolite and the MSMS secondary fragments of the standard substance in the database/3alpha,7alpha-Dihydroxy-12-oxo-5beta-cholanate.png]

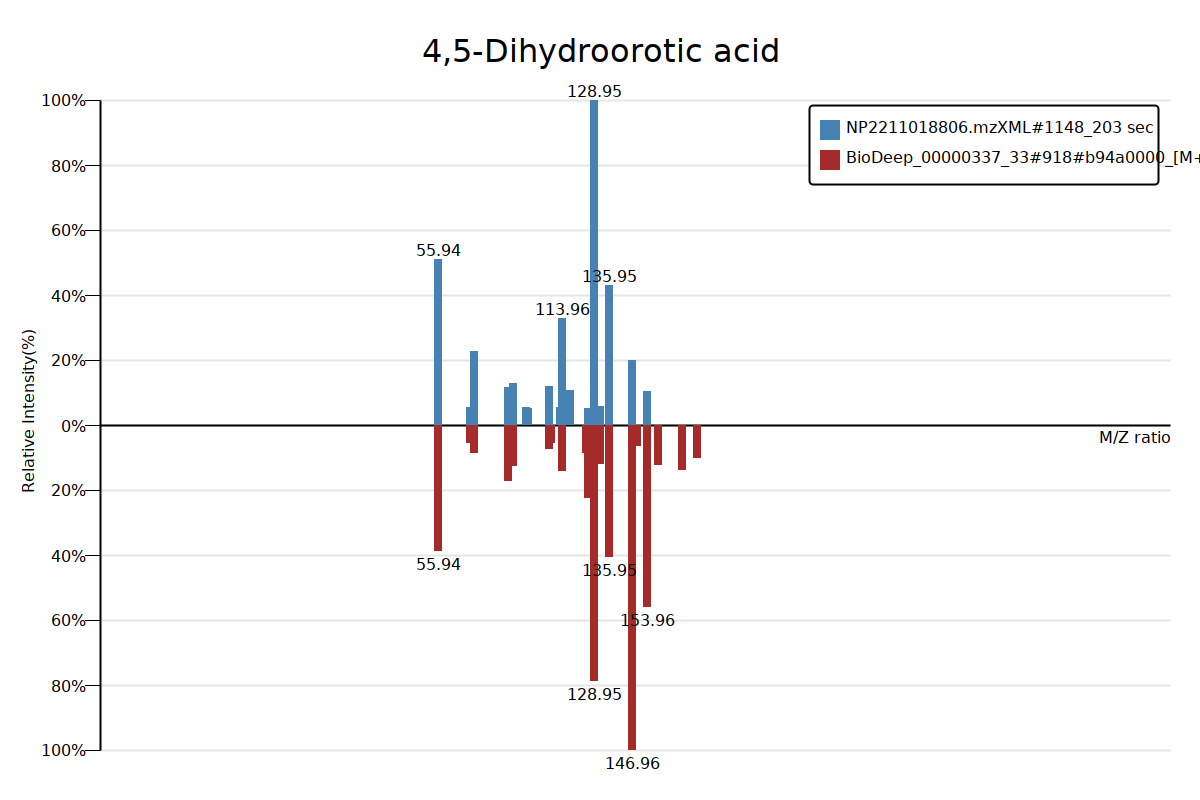

Supplement: Supplementary file 5 [file DataSheet1.ZIP › 2 result graphs between the MSMS secondary fragments of each metabolite and the MSMS secondary fragments of the standard substance in the database/4,5-Dihydroorotic acid.png]

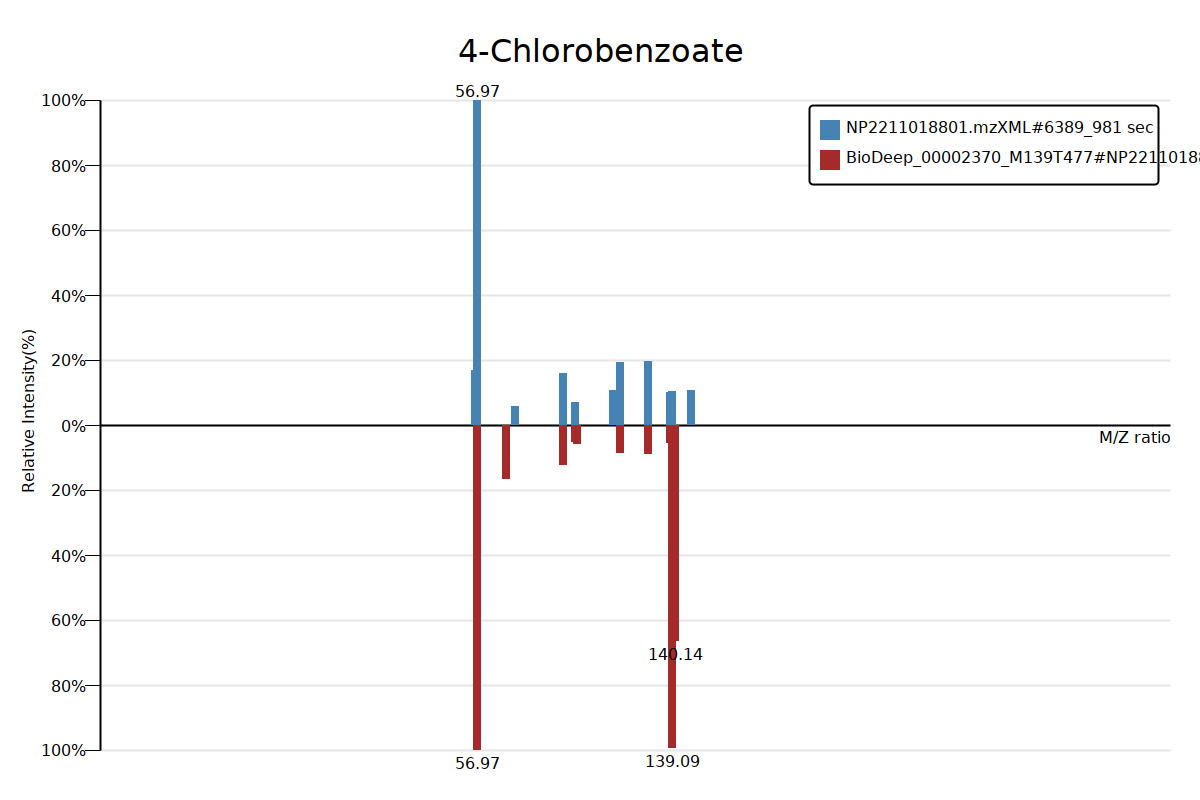

Supplement: Supplementary file 5 [file DataSheet1.ZIP › 2 result graphs between the MSMS secondary fragments of each metabolite and the MSMS secondary fragments of the standard substance in the database/4-Chlorobenzoate.png]

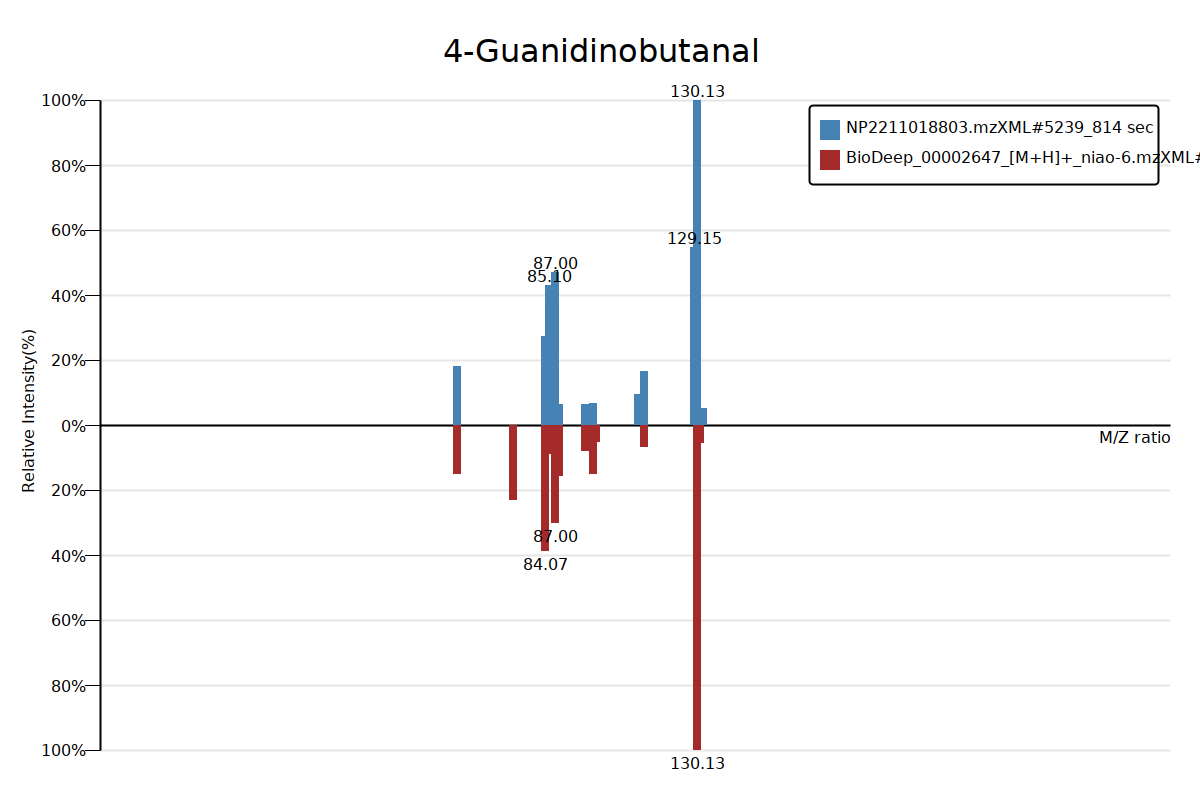

Supplement: Supplementary file 5 [file DataSheet1.ZIP › 2 result graphs between the MSMS secondary fragments of each metabolite and the MSMS secondary fragments of the standard substance in the database/4-Guanidinobutanal.png]

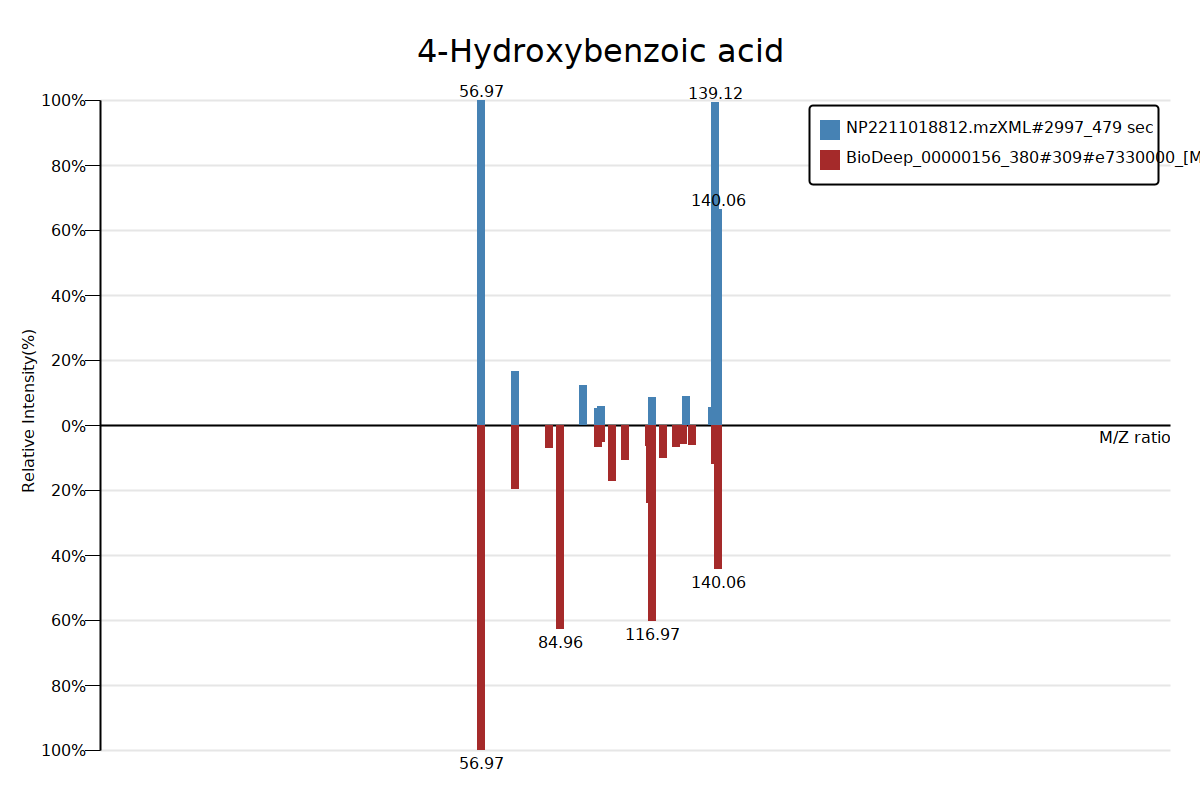

Supplement: Supplementary file 5 [file DataSheet1.ZIP › 2 result graphs between the MSMS secondary fragments of each metabolite and the MSMS secondary fragments of the standard substance in the database/4-Hydroxybenzoic acid.png]

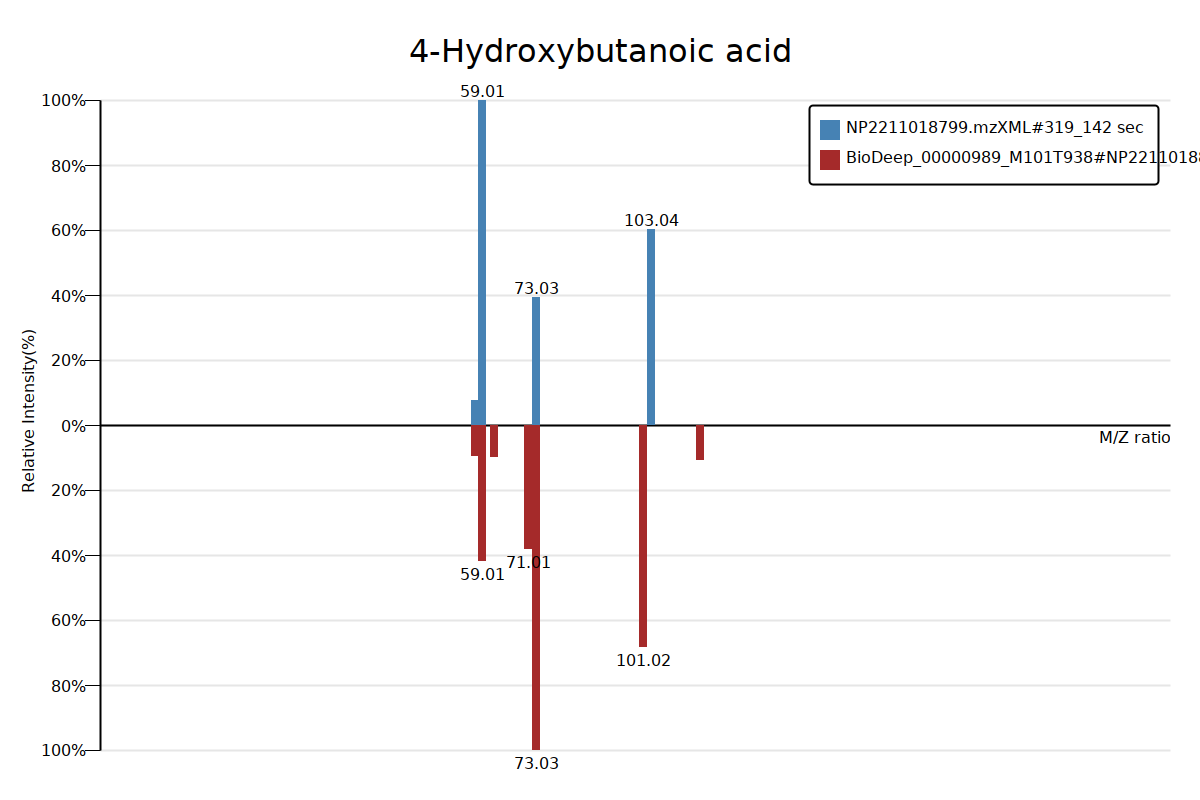

Supplement: Supplementary file 5 [file DataSheet1.ZIP › 2 result graphs between the MSMS secondary fragments of each metabolite and the MSMS secondary fragments of the standard substance in the database/4-Hydroxybutanoic acid.png]

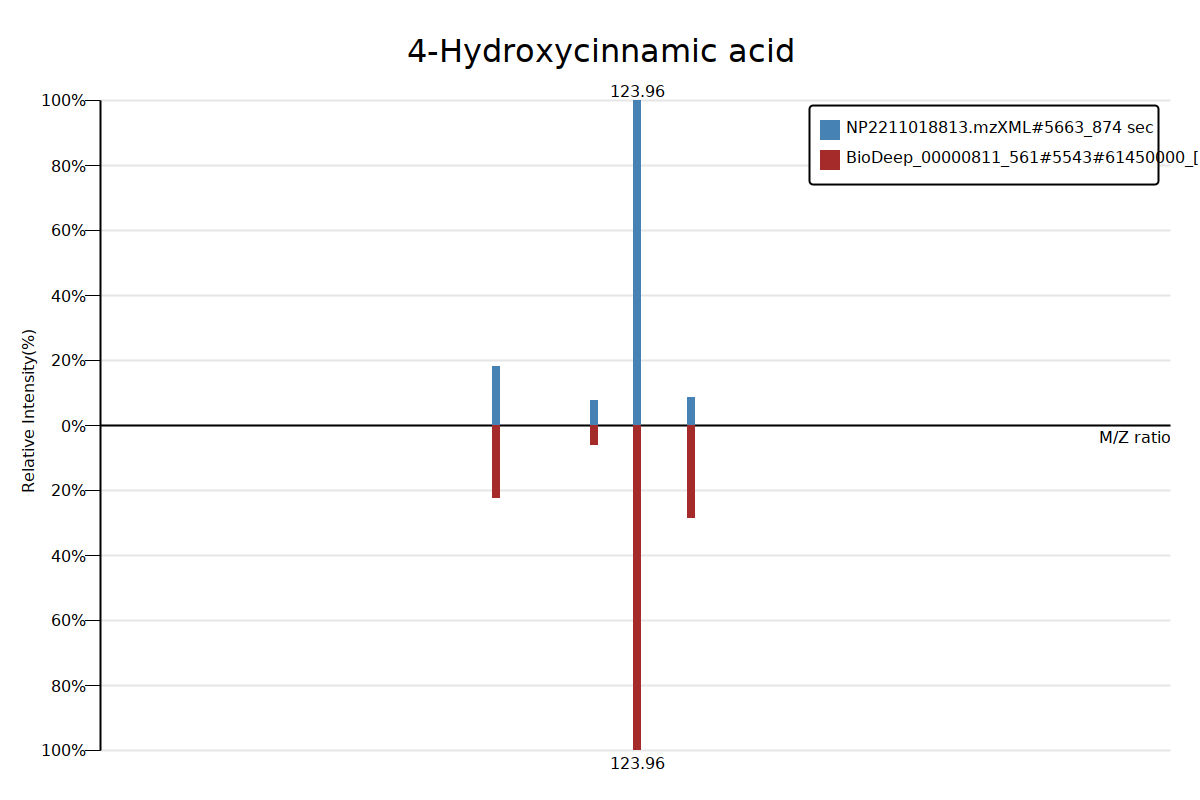

Supplement: Supplementary file 5 [file DataSheet1.ZIP › 2 result graphs between the MSMS secondary fragments of each metabolite and the MSMS secondary fragments of the standard substance in the database/4-Hydroxycinnamic acid.png]

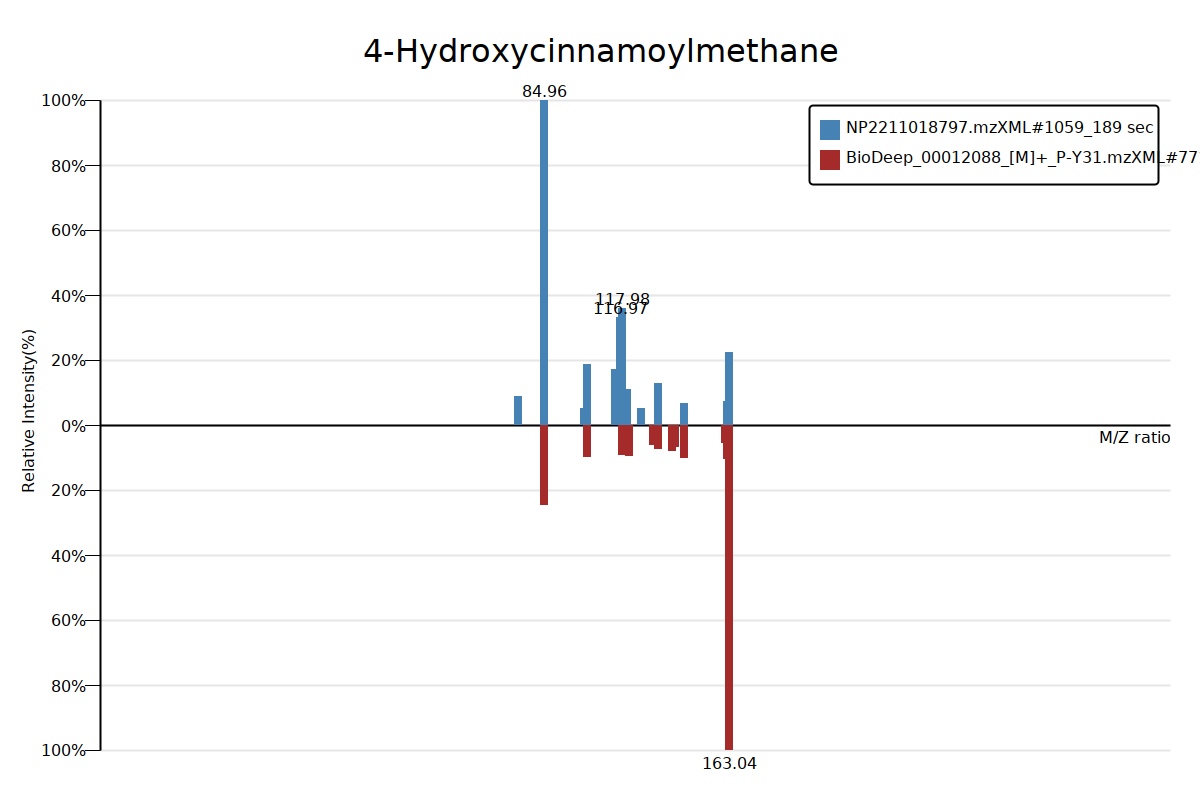

Supplement: Supplementary file 5 [file DataSheet1.ZIP › 2 result graphs between the MSMS secondary fragments of each metabolite and the MSMS secondary fragments of the standard substance in the database/4-Hydroxycinnamoylmethane.png]

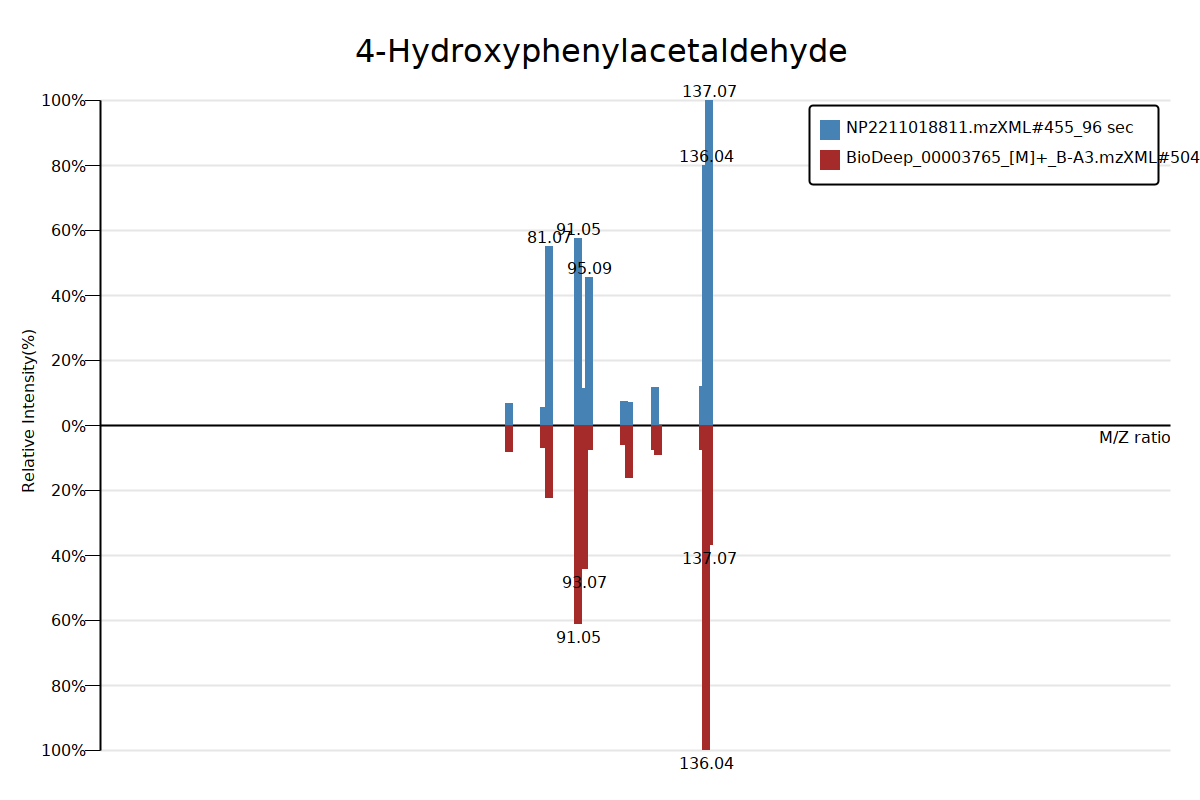

Supplement: Supplementary file 5 [file DataSheet1.ZIP › 2 result graphs between the MSMS secondary fragments of each metabolite and the MSMS secondary fragments of the standard substance in the database/4-Hydroxyphenylacetaldehyde.png]

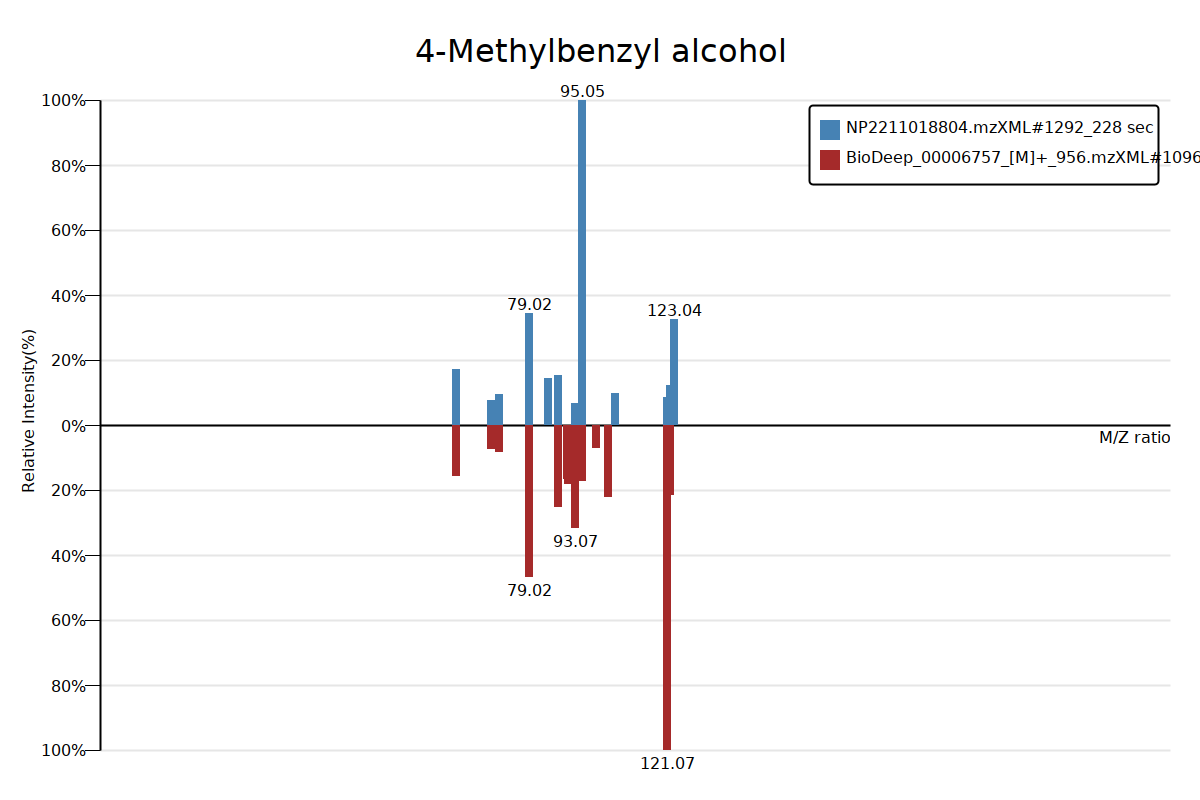

Supplement: Supplementary file 5 [file DataSheet1.ZIP › 2 result graphs between the MSMS secondary fragments of each metabolite and the MSMS secondary fragments of the standard substance in the database/4-Methylbenzyl alcohol.png]

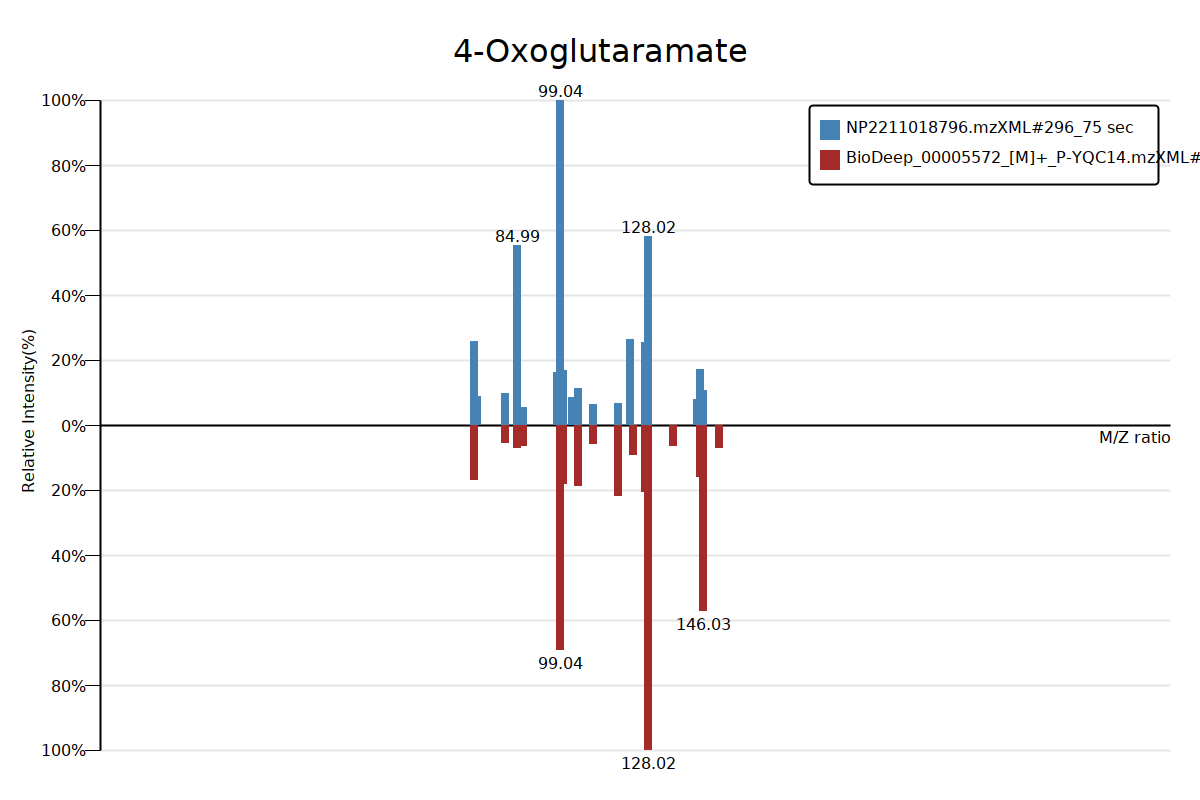

Supplement: Supplementary file 5 [file DataSheet1.ZIP › 2 result graphs between the MSMS secondary fragments of each metabolite and the MSMS secondary fragments of the standard substance in the database/4-Oxoglutaramate.png]

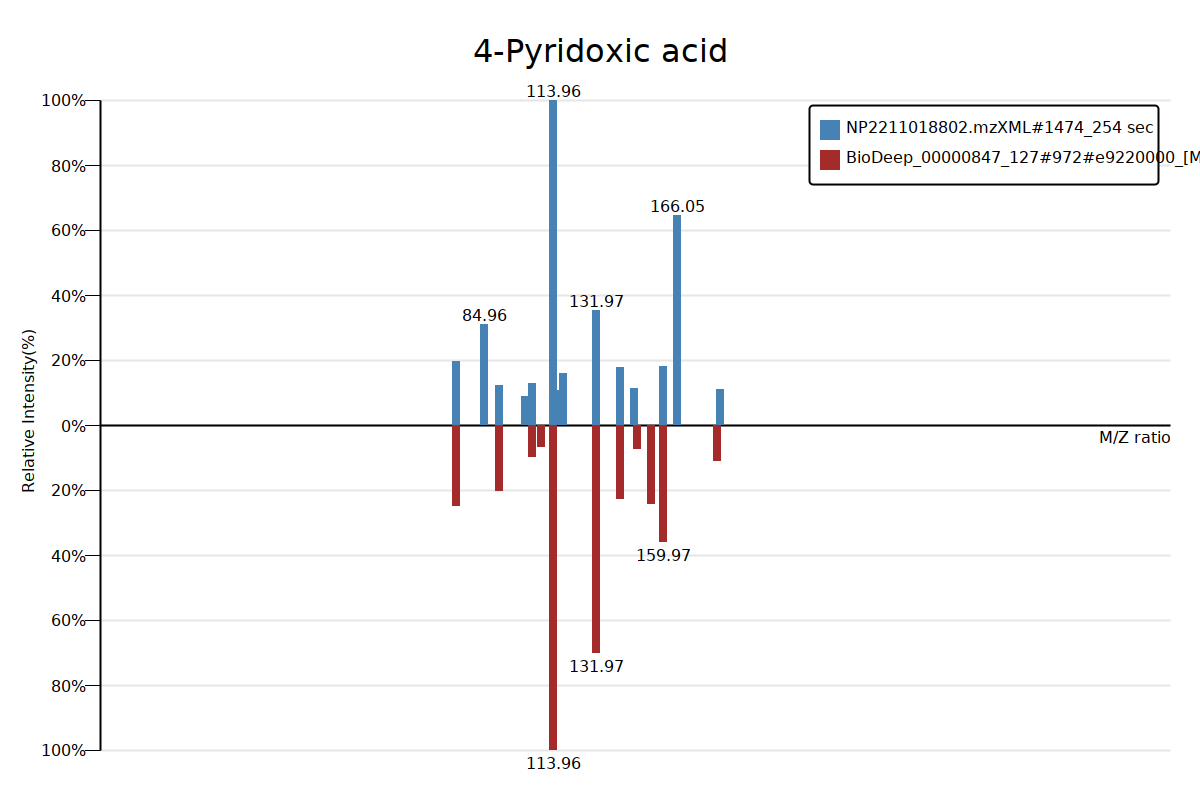

Supplement: Supplementary file 5 [file DataSheet1.ZIP › 2 result graphs between the MSMS secondary fragments of each metabolite and the MSMS secondary fragments of the standard substance in the database/4-Pyridoxic acid.png]

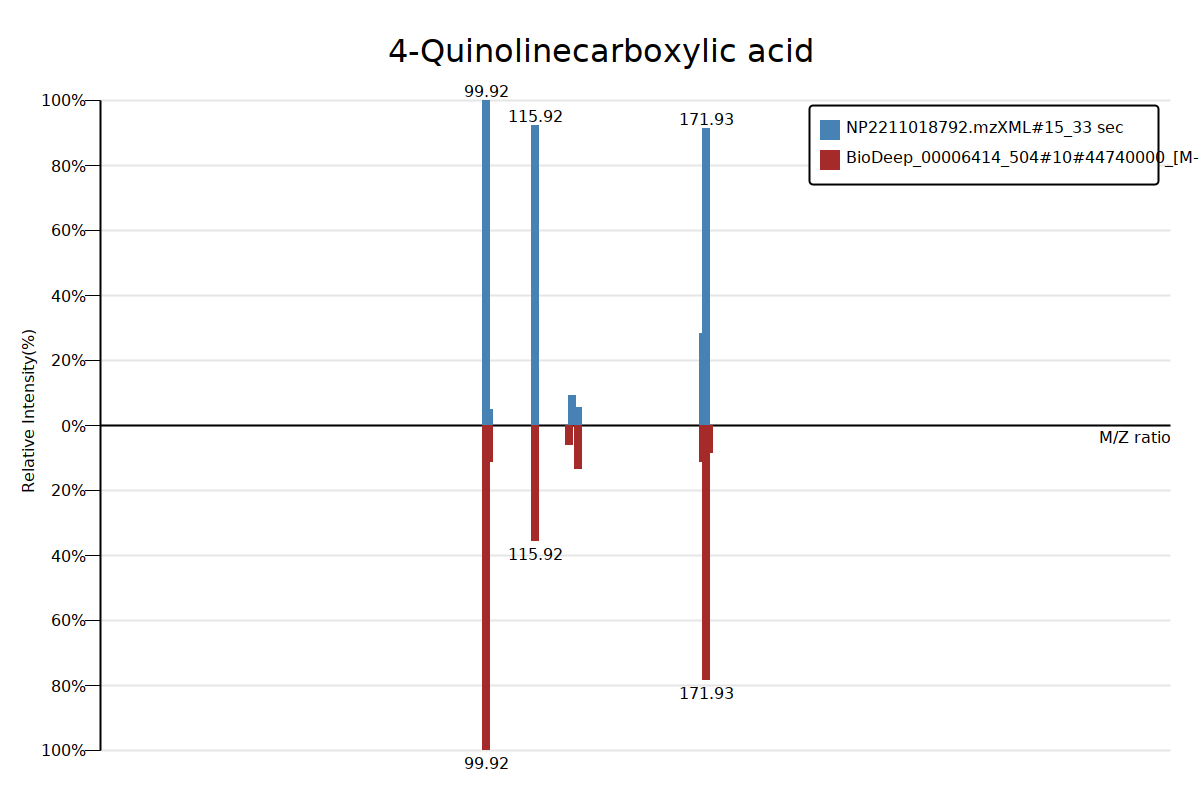

Supplement: Supplementary file 5 [file DataSheet1.ZIP › 2 result graphs between the MSMS secondary fragments of each metabolite and the MSMS secondary fragments of the standard substance in the database/4-Quinolinecarboxylic acid.png]

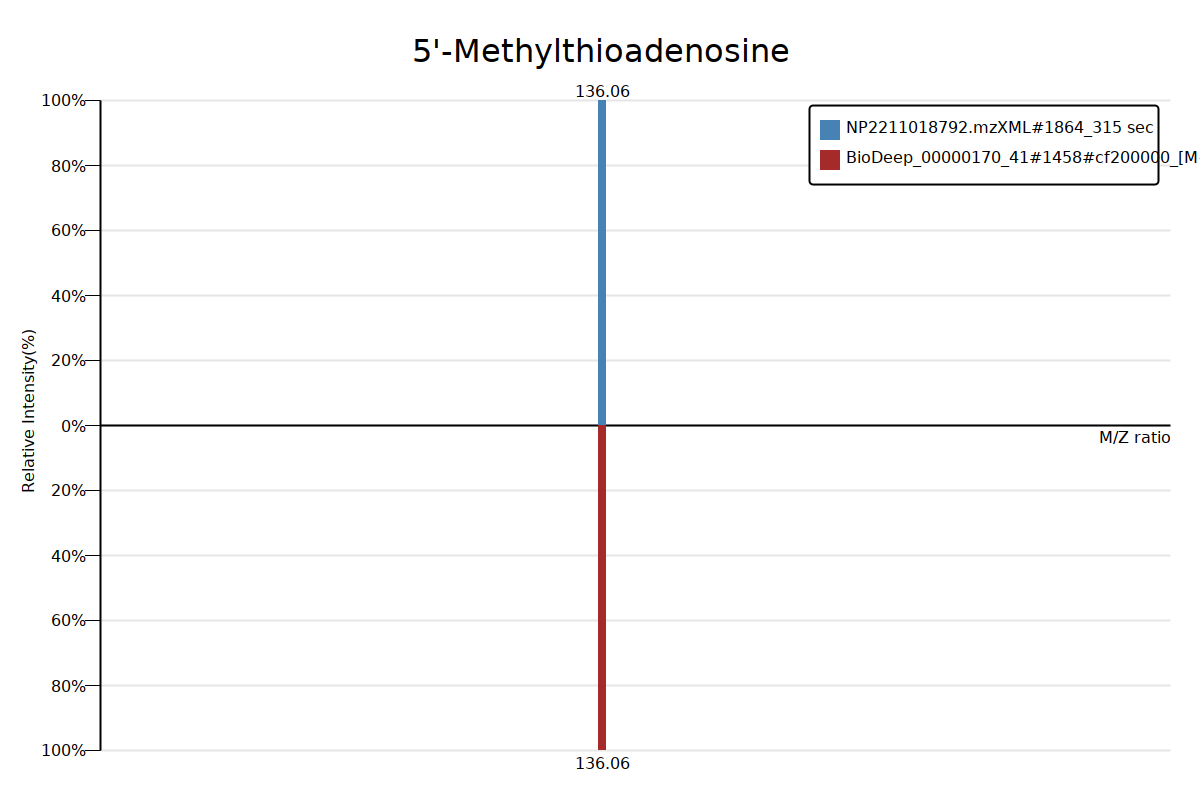

Supplement: Supplementary file 5 [file DataSheet1.ZIP › 2 result graphs between the MSMS secondary fragments of each metabolite and the MSMS secondary fragments of the standard substance in the database/5'-Methylthioadenosine.png]

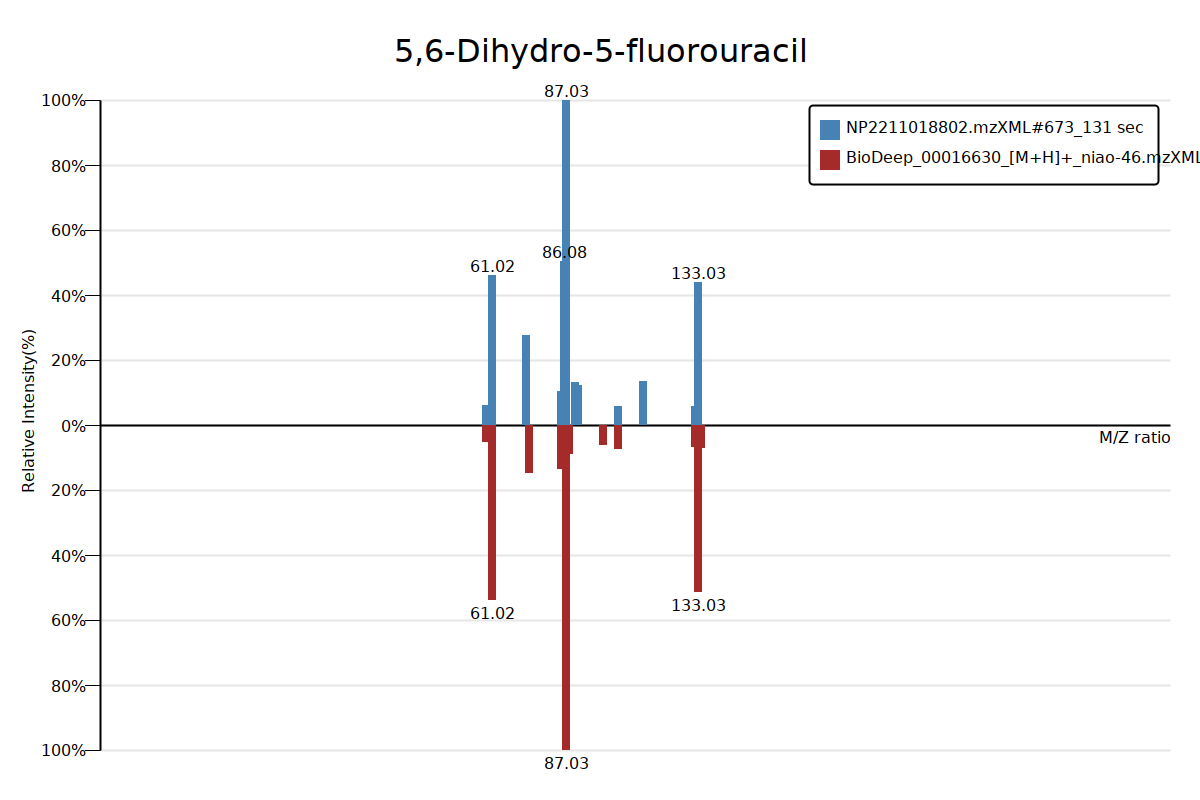

Supplement: Supplementary file 5 [file DataSheet1.ZIP › 2 result graphs between the MSMS secondary fragments of each metabolite and the MSMS secondary fragments of the standard substance in the database/5,6-Dihydro-5-fluorouracil.png]

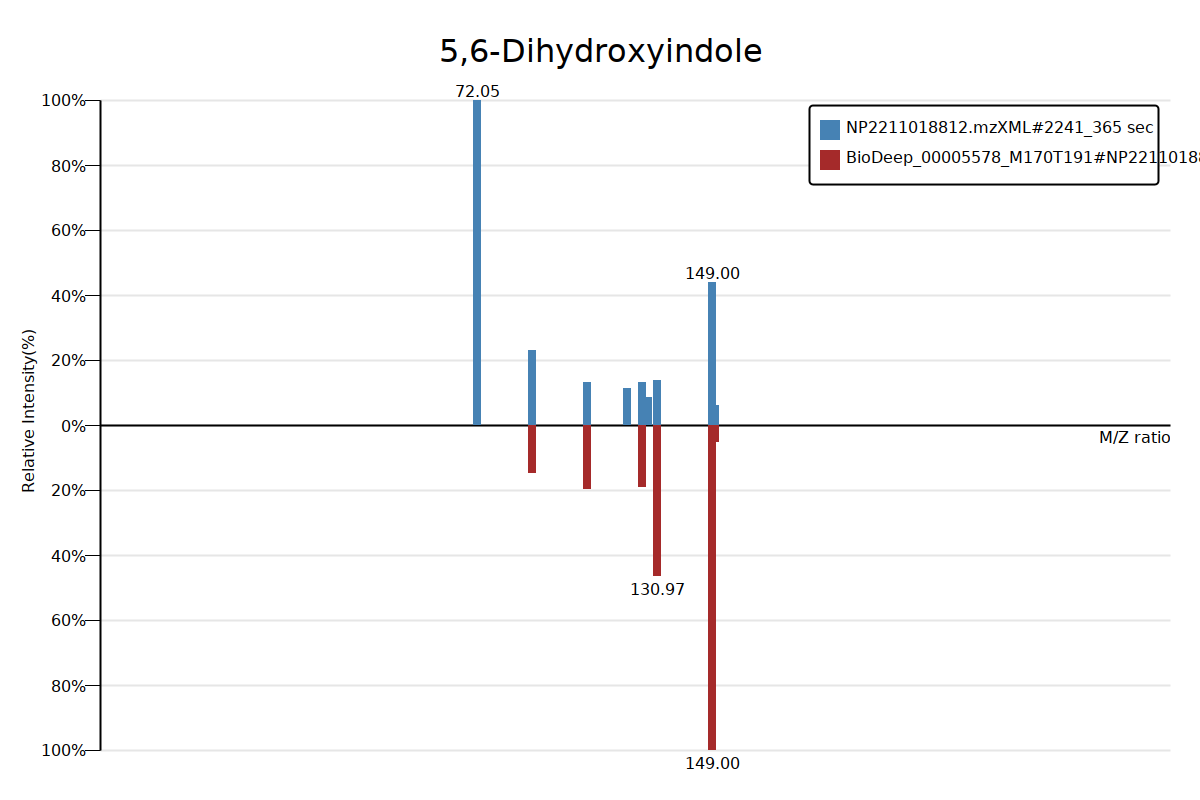

Supplement: Supplementary file 5 [file DataSheet1.ZIP › 2 result graphs between the MSMS secondary fragments of each metabolite and the MSMS secondary fragments of the standard substance in the database/5,6-Dihydroxyindole.png]

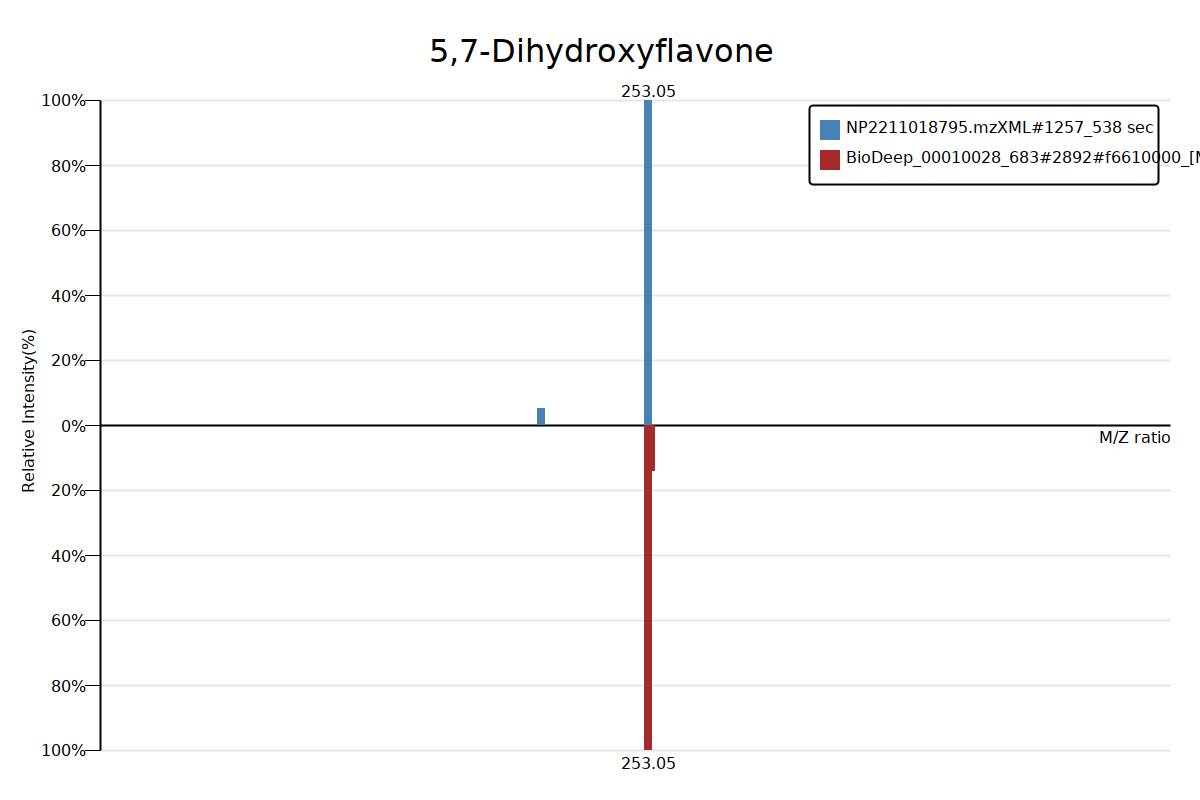

Supplement: Supplementary file 5 [file DataSheet1.ZIP › 2 result graphs between the MSMS secondary fragments of each metabolite and the MSMS secondary fragments of the standard substance in the database/5,7-Dihydroxyflavone.png]

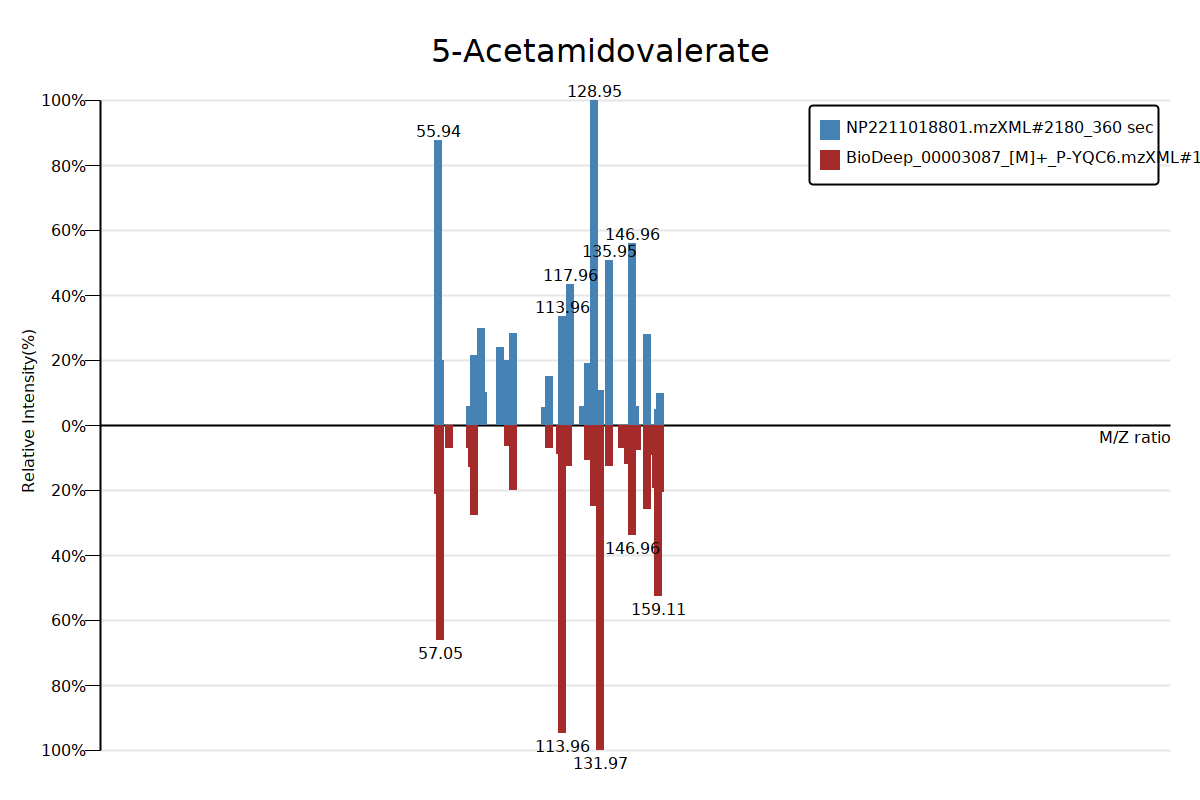

Supplement: Supplementary file 5 [file DataSheet1.ZIP › 2 result graphs between the MSMS secondary fragments of each metabolite and the MSMS secondary fragments of the standard substance in the database/5-Acetamidovalerate.png]

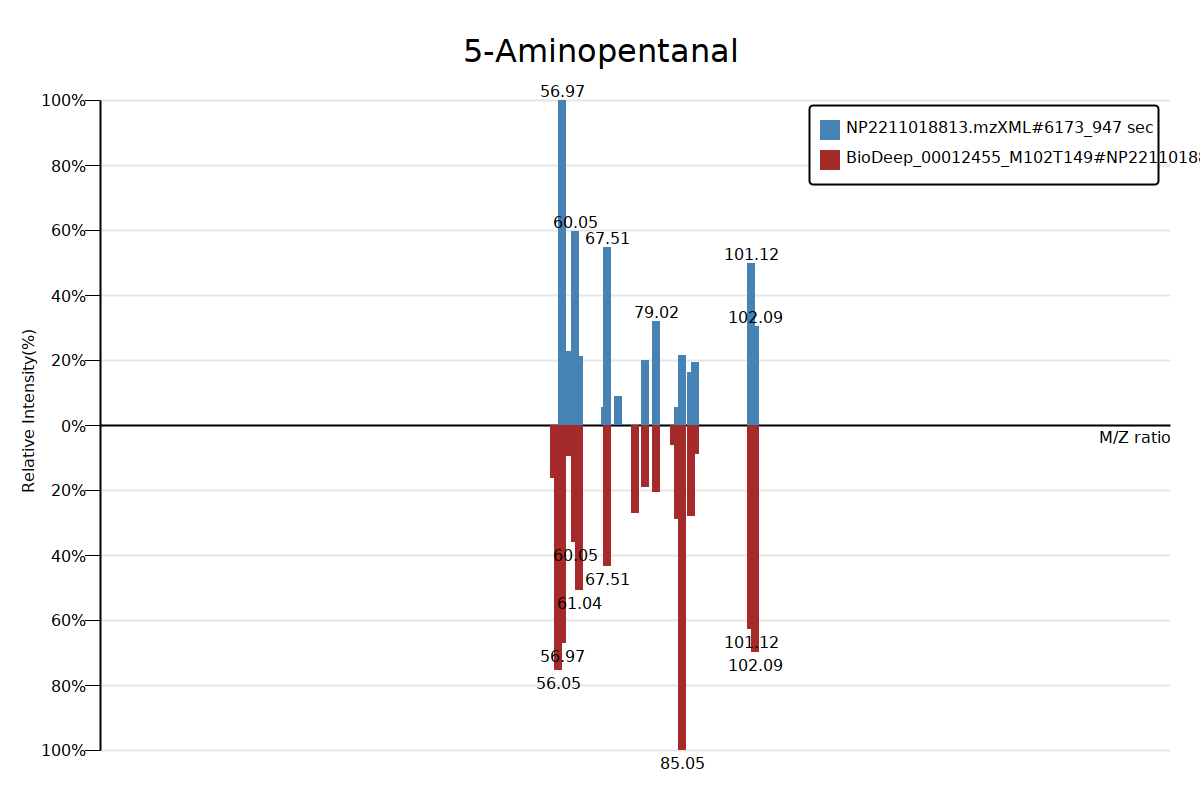

Supplement: Supplementary file 5 [file DataSheet1.ZIP › 2 result graphs between the MSMS secondary fragments of each metabolite and the MSMS secondary fragments of the standard substance in the database/5-Aminopentanal.png]

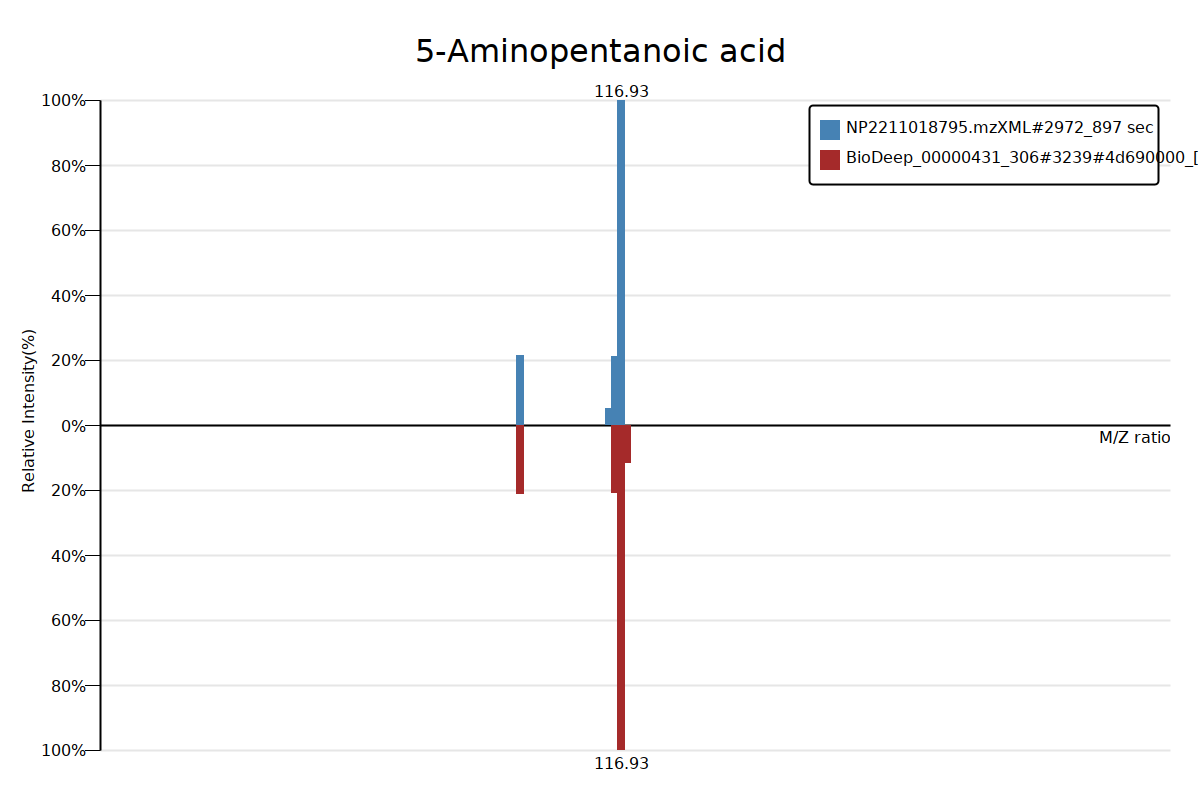

Supplement: Supplementary file 5 [file DataSheet1.ZIP › 2 result graphs between the MSMS secondary fragments of each metabolite and the MSMS secondary fragments of the standard substance in the database/5-Aminopentanoic acid.png]

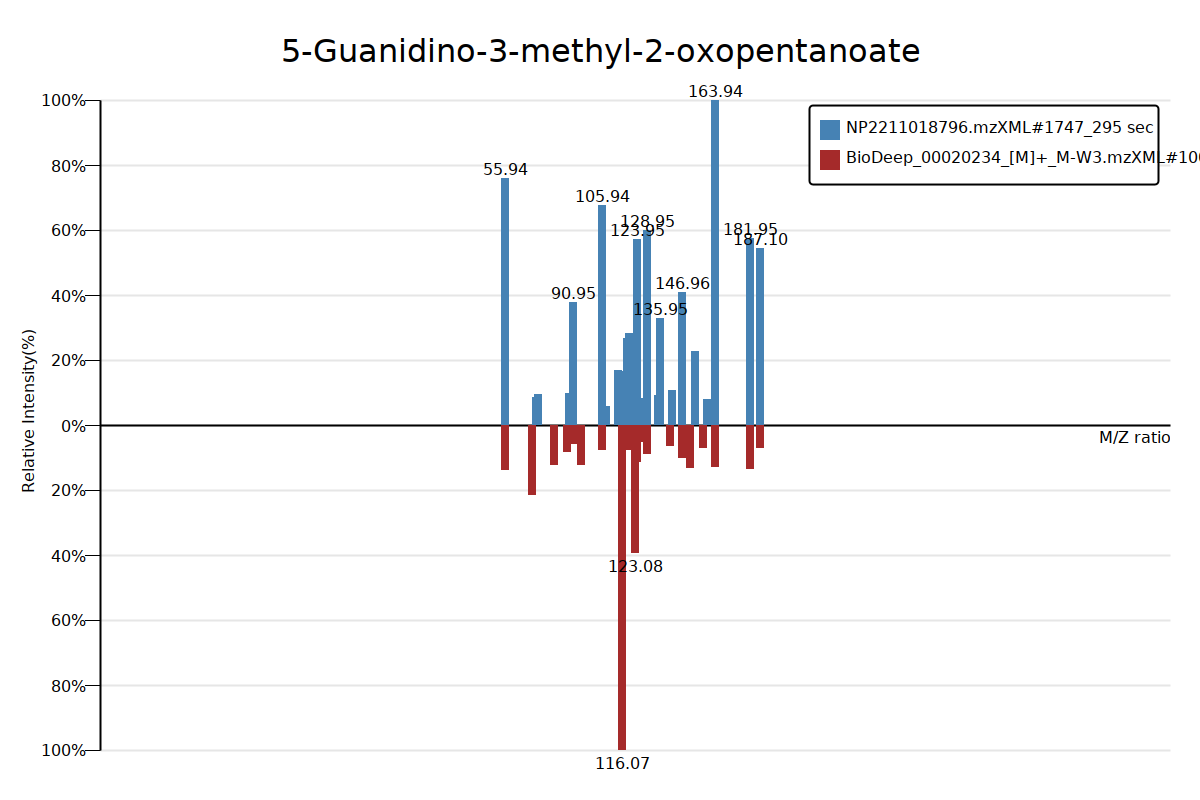

Supplement: Supplementary file 5 [file DataSheet1.ZIP › 2 result graphs between the MSMS secondary fragments of each metabolite and the MSMS secondary fragments of the standard substance in the database/5-Guanidino-3-methyl-2-oxopentanoate.png]

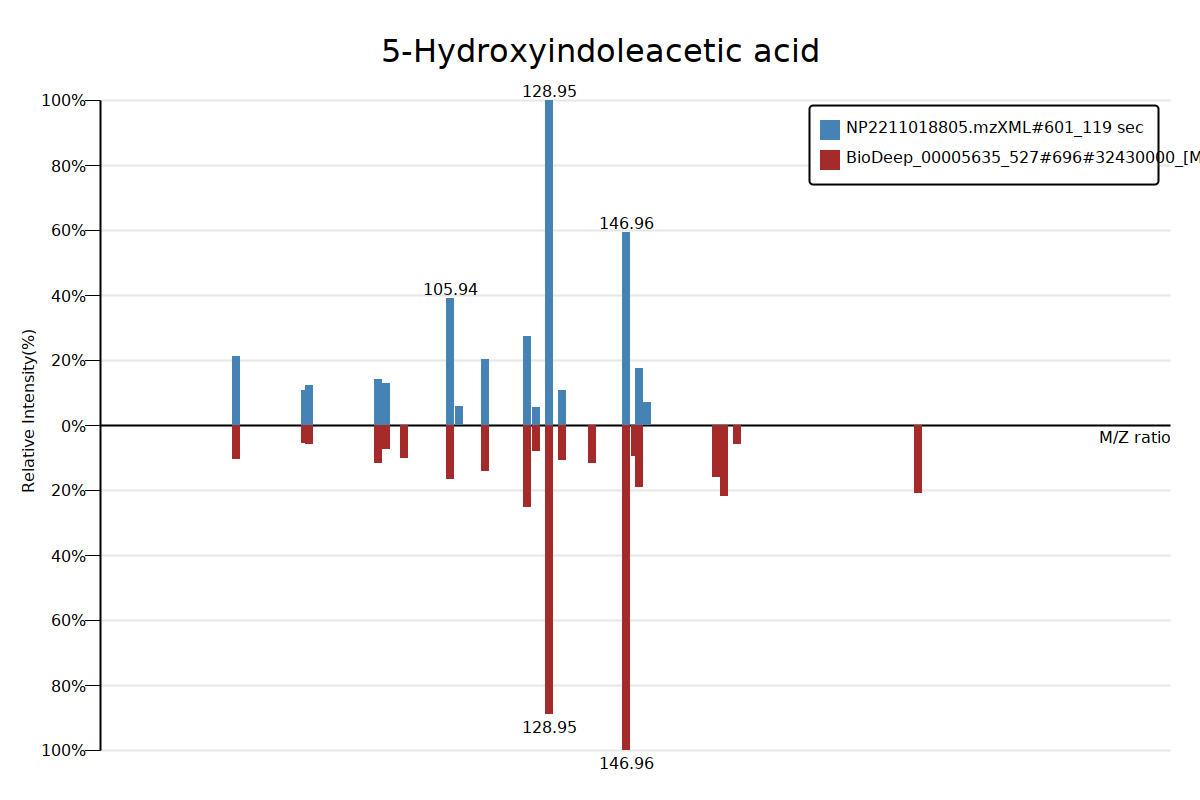

Supplement: Supplementary file 5 [file DataSheet1.ZIP › 2 result graphs between the MSMS secondary fragments of each metabolite and the MSMS secondary fragments of the standard substance in the database/5-Hydroxyindoleacetic acid.png]

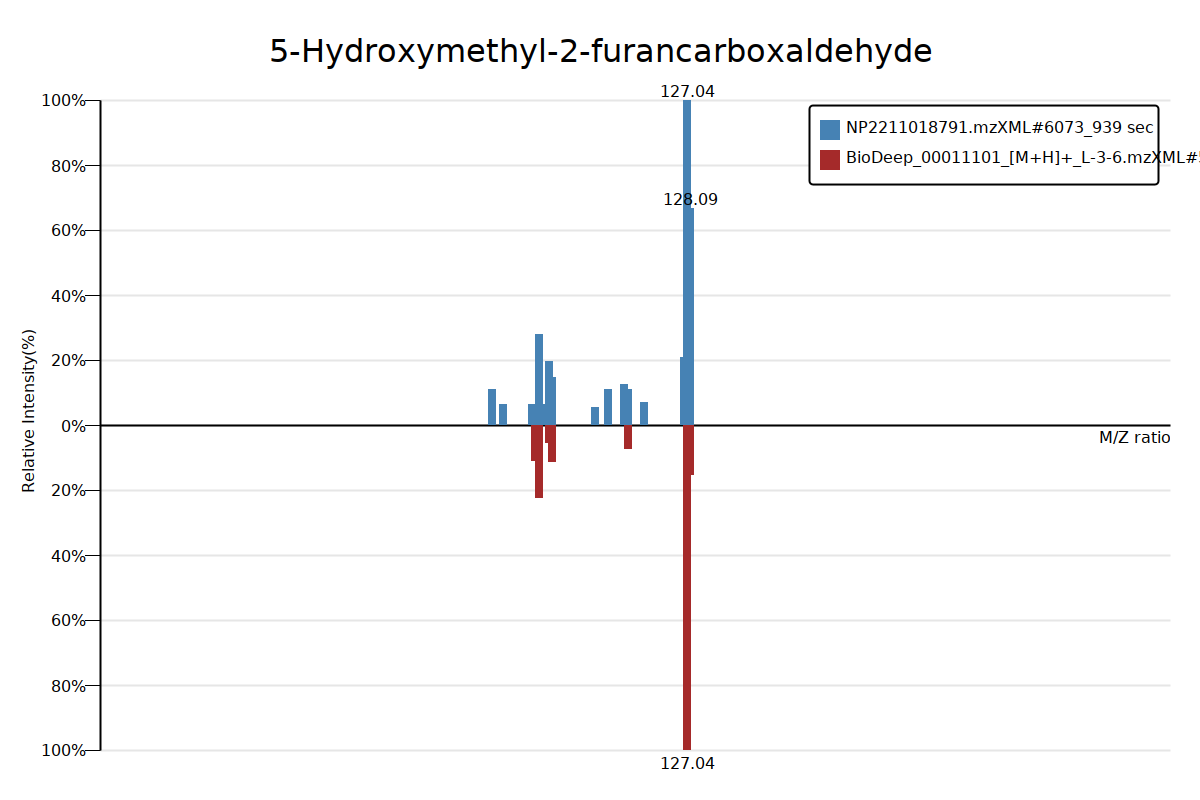

Supplement: Supplementary file 5 [file DataSheet1.ZIP › 2 result graphs between the MSMS secondary fragments of each metabolite and the MSMS secondary fragments of the standard substance in the database/5-Hydroxymethyl-2-furancarboxaldehyde.png]

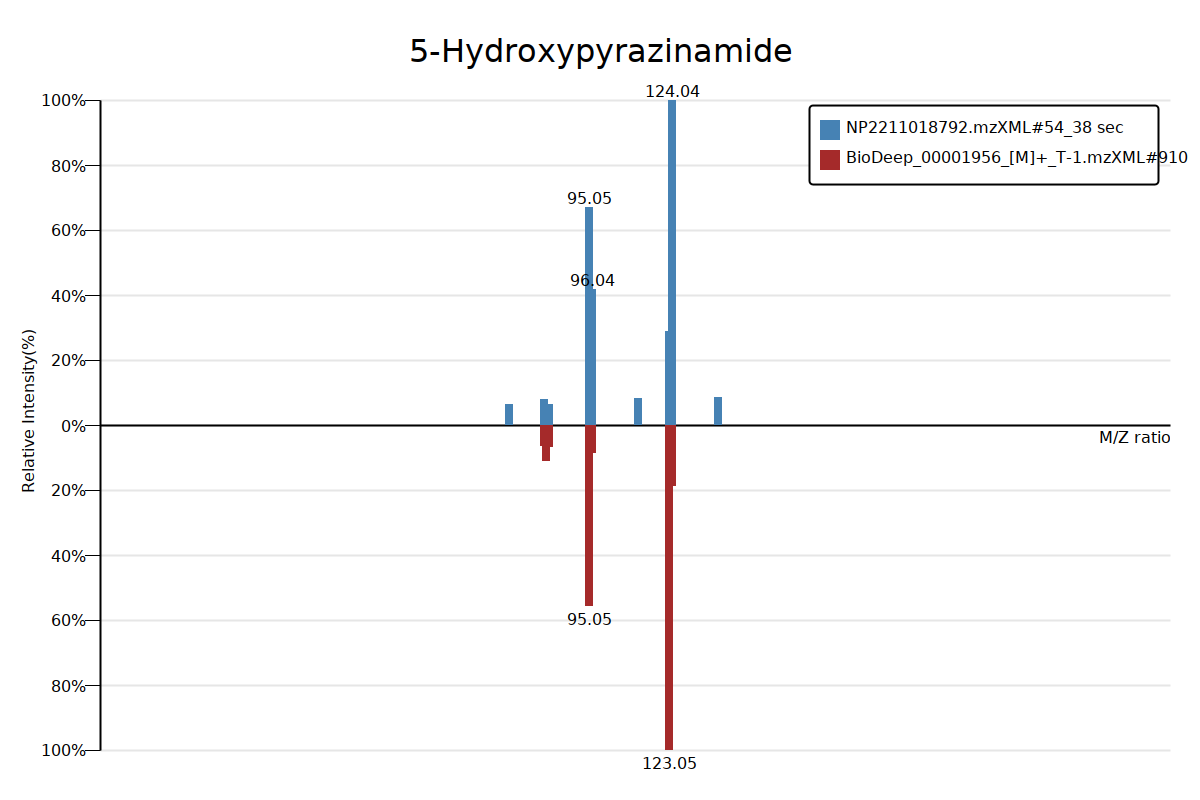

Supplement: Supplementary file 5 [file DataSheet1.ZIP › 2 result graphs between the MSMS secondary fragments of each metabolite and the MSMS secondary fragments of the standard substance in the database/5-Hydroxypyrazinamide.png]

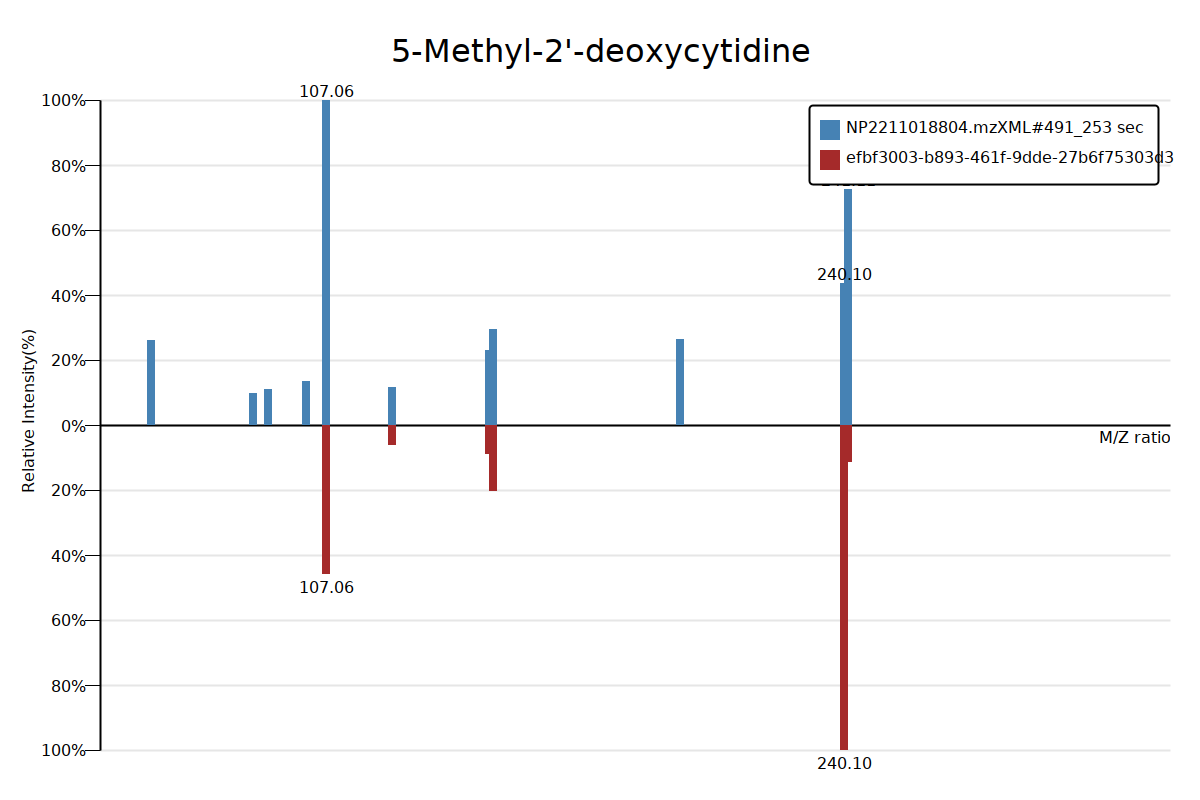

Supplement: Supplementary file 5 [file DataSheet1.ZIP › 2 result graphs between the MSMS secondary fragments of each metabolite and the MSMS secondary fragments of the standard substance in the database/5-Methyl-2'-deoxycytidine.png]

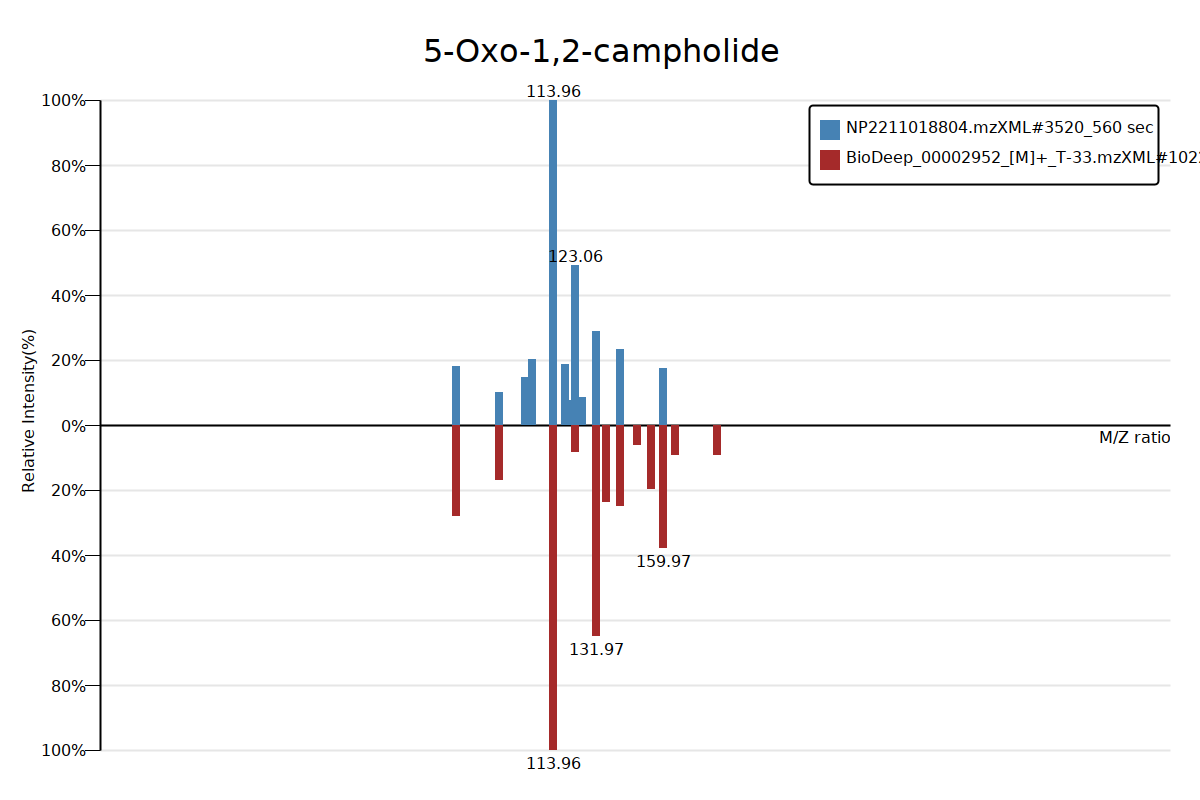

Supplement: Supplementary file 5 [file DataSheet1.ZIP › 2 result graphs between the MSMS secondary fragments of each metabolite and the MSMS secondary fragments of the standard substance in the database/5-Oxo-1,2-campholide.png]

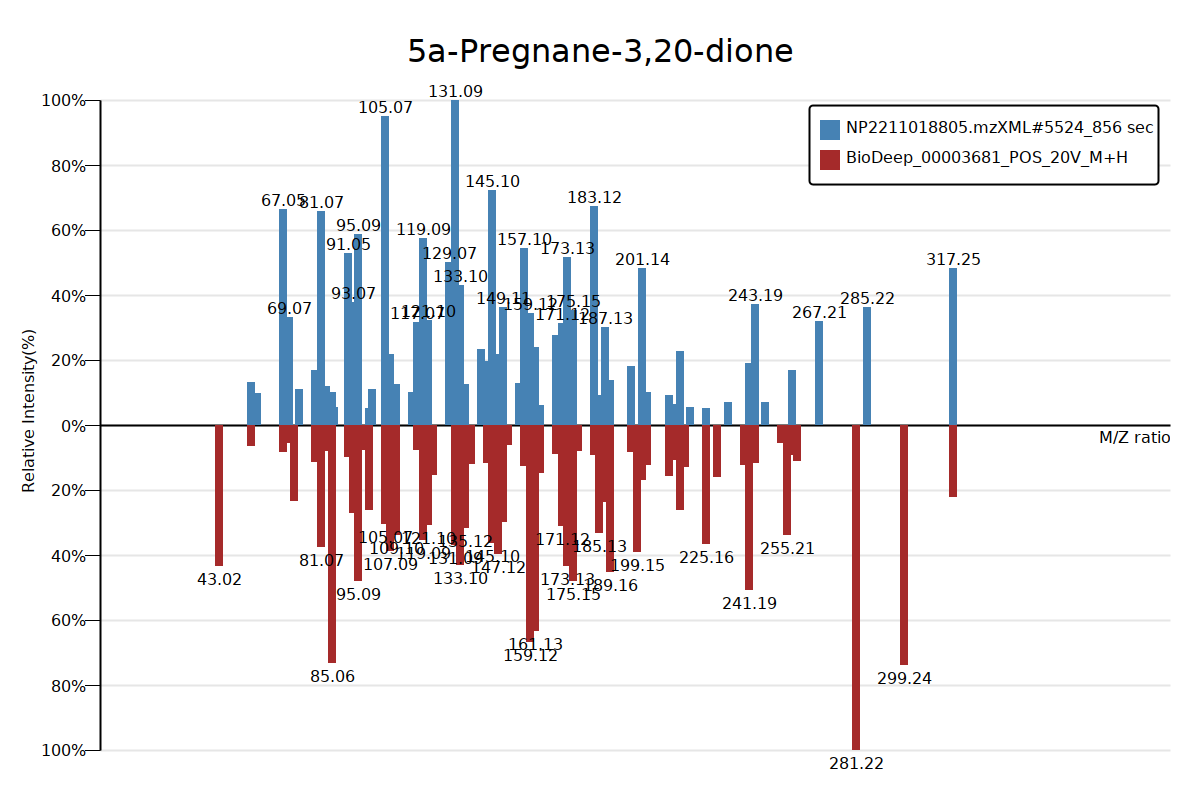

Supplement: Supplementary file 5 [file DataSheet1.ZIP › 2 result graphs between the MSMS secondary fragments of each metabolite and the MSMS secondary fragments of the standard substance in the database/5a-Pregnane-3,20-dione.png]

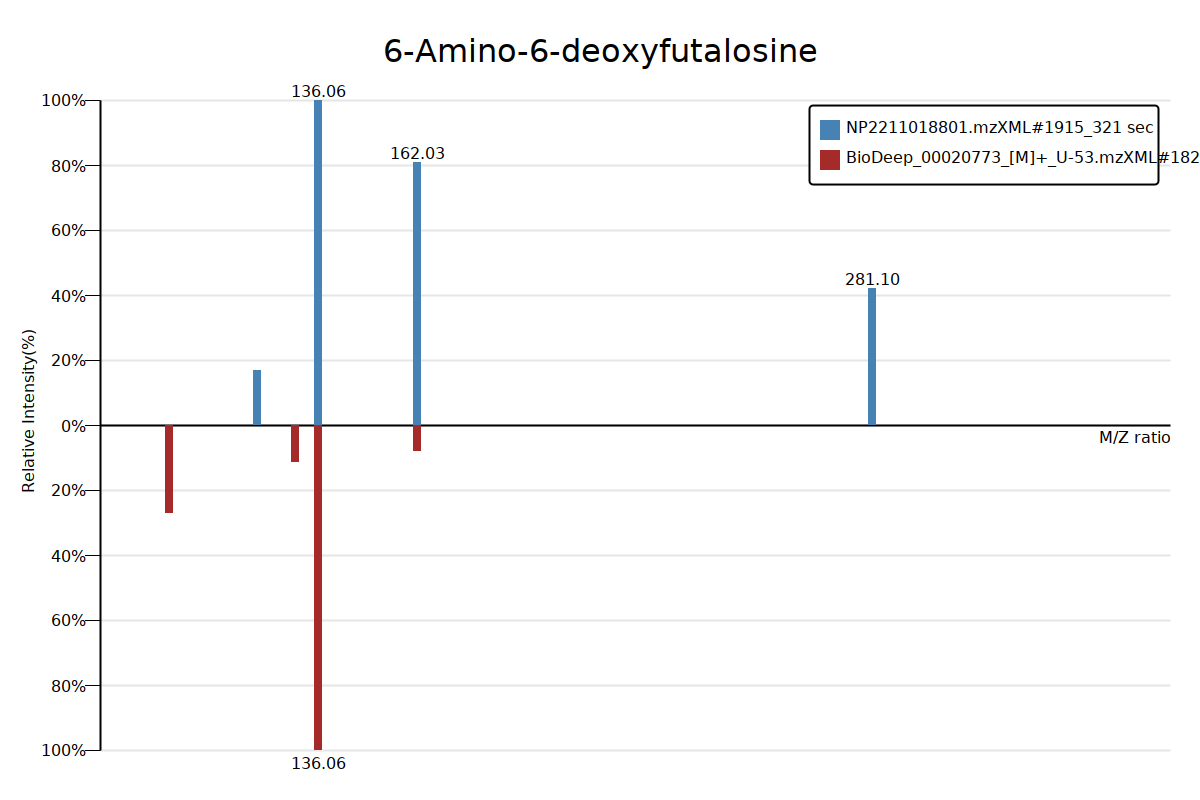

Supplement: Supplementary file 5 [file DataSheet1.ZIP › 2 result graphs between the MSMS secondary fragments of each metabolite and the MSMS secondary fragments of the standard substance in the database/6-Amino-6-deoxyfutalosine.png]

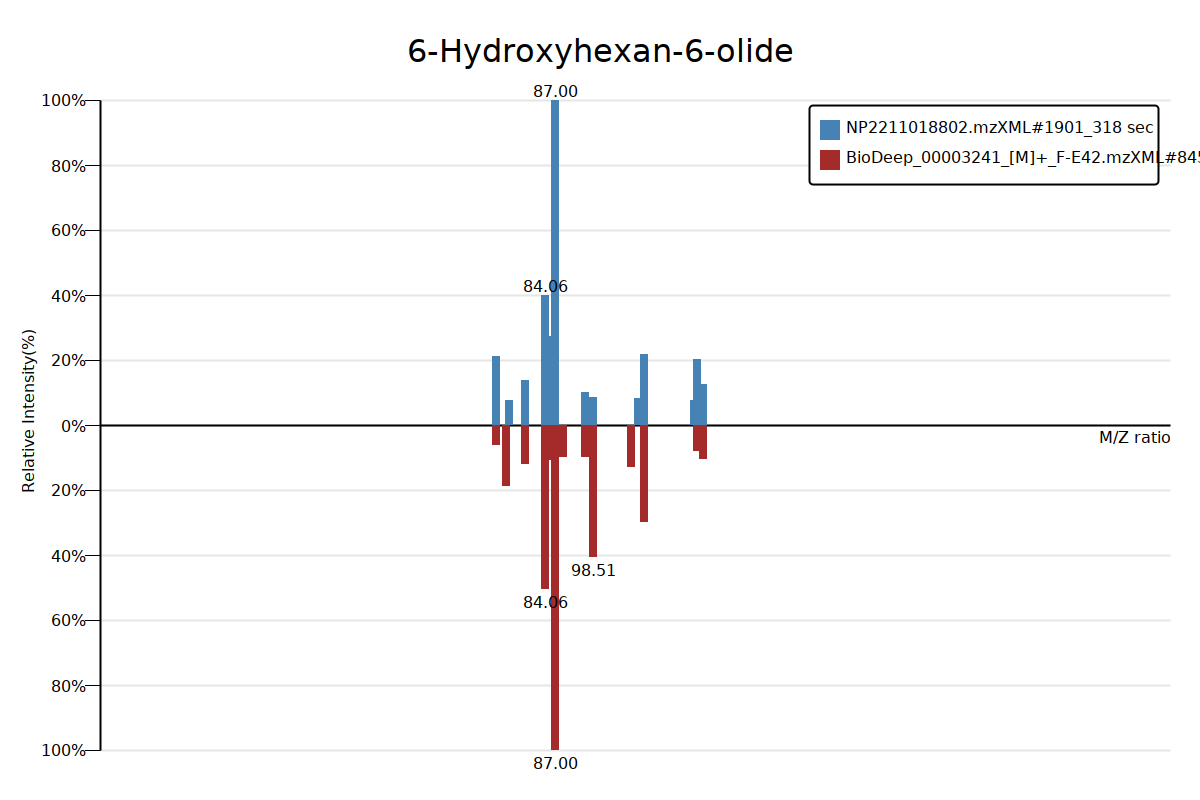

Supplement: Supplementary file 5 [file DataSheet1.ZIP › 2 result graphs between the MSMS secondary fragments of each metabolite and the MSMS secondary fragments of the standard substance in the database/6-Hydroxyhexan-6-olide.png]

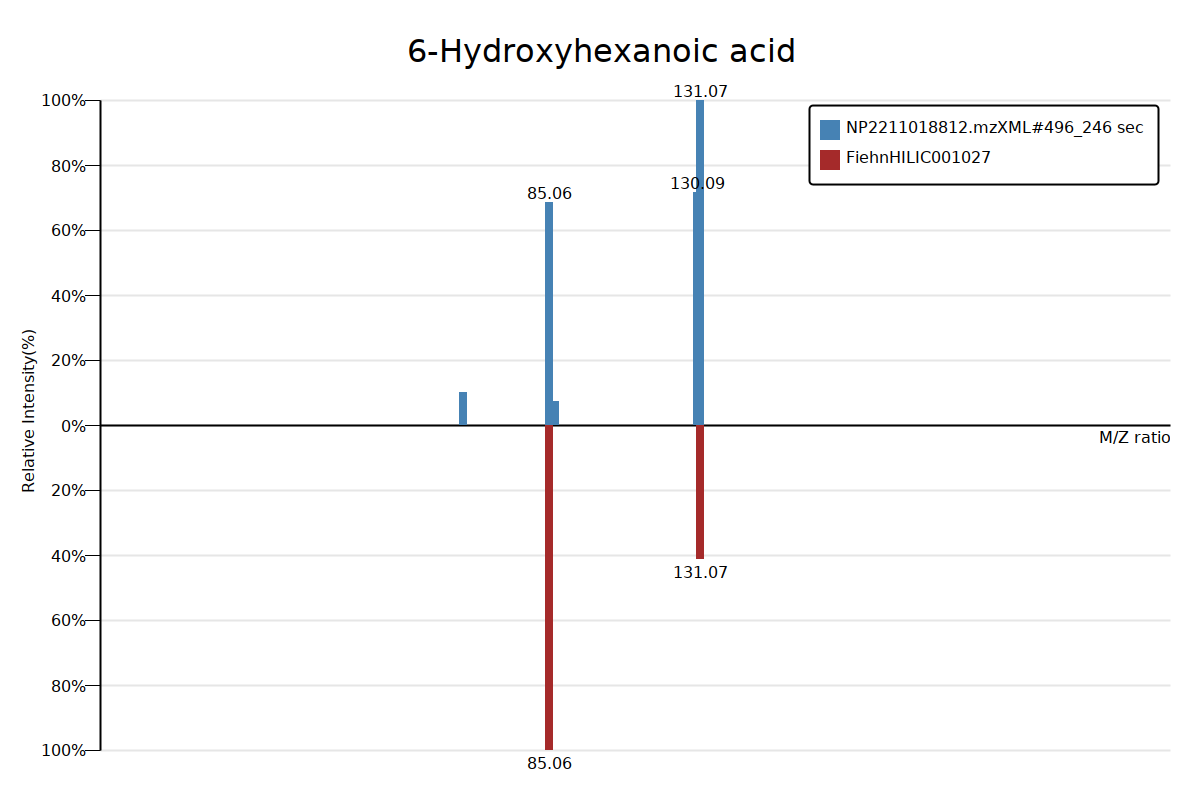

Supplement: Supplementary file 5 [file DataSheet1.ZIP › 2 result graphs between the MSMS secondary fragments of each metabolite and the MSMS secondary fragments of the standard substance in the database/6-Hydroxyhexanoic acid.png]

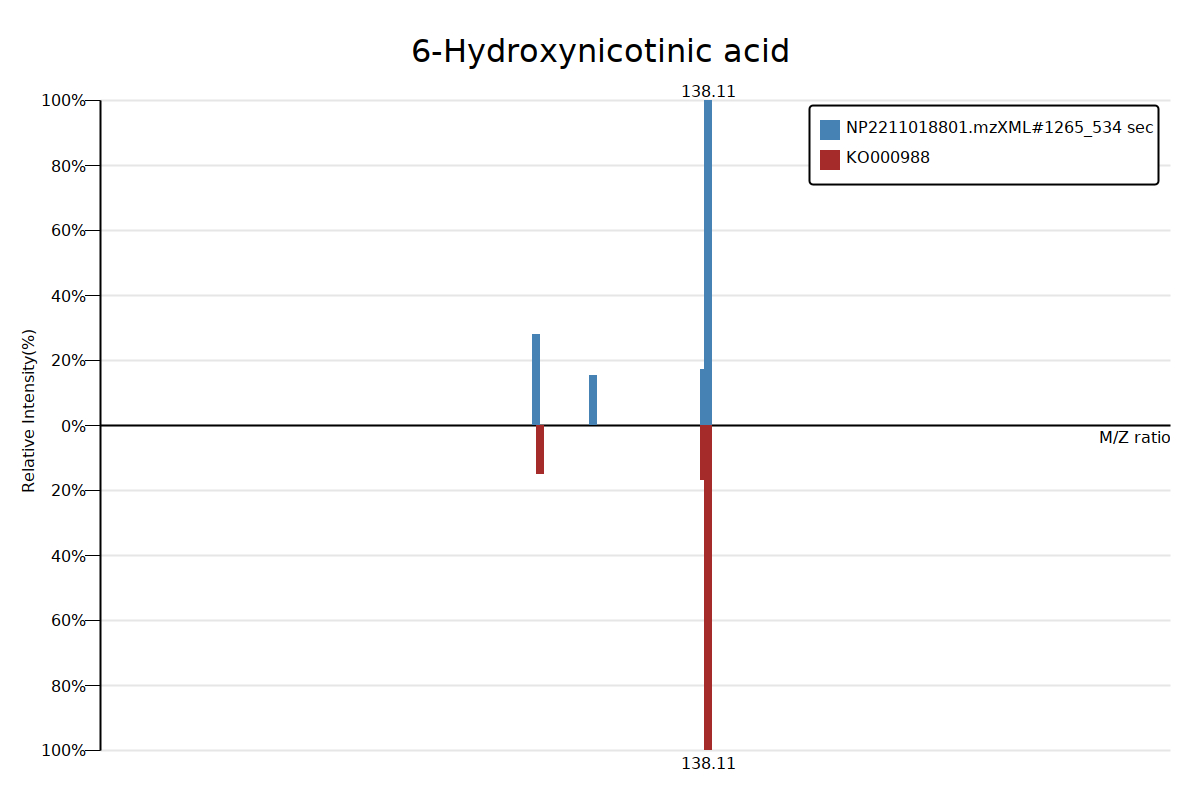

Supplement: Supplementary file 5 [file DataSheet1.ZIP › 2 result graphs between the MSMS secondary fragments of each metabolite and the MSMS secondary fragments of the standard substance in the database/6-Hydroxynicotinic acid.png]

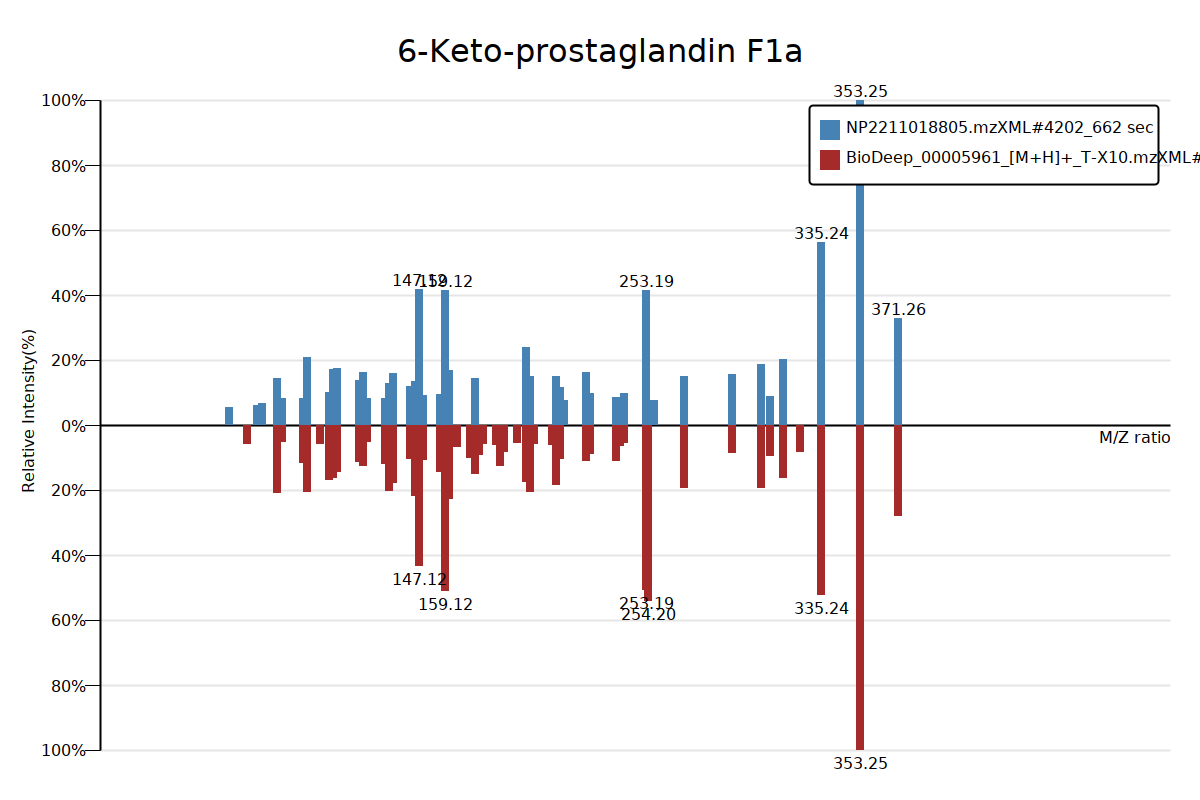

Supplement: Supplementary file 5 [file DataSheet1.ZIP › 2 result graphs between the MSMS secondary fragments of each metabolite and the MSMS secondary fragments of the standard substance in the database/6-Keto-prostaglandin F1a.png]

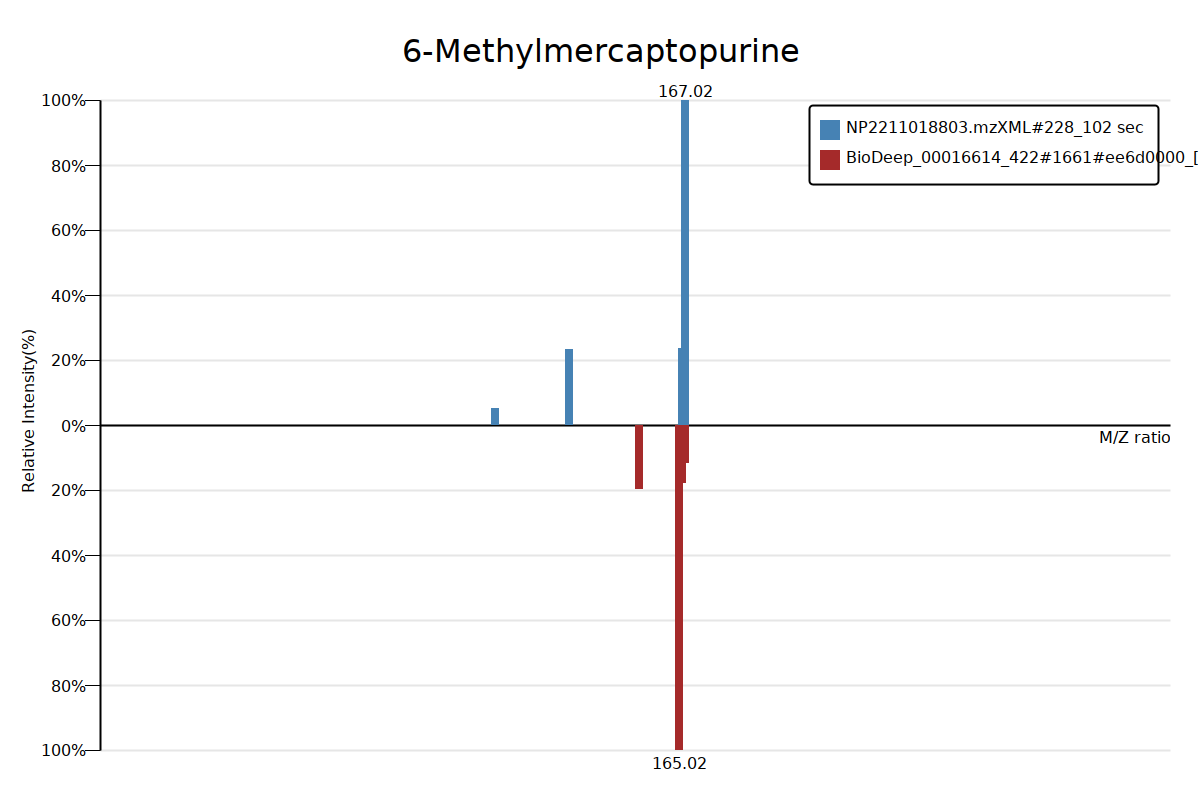

Supplement: Supplementary file 5 [file DataSheet1.ZIP › 2 result graphs between the MSMS secondary fragments of each metabolite and the MSMS secondary fragments of the standard substance in the database/6-Methylmercaptopurine.png]

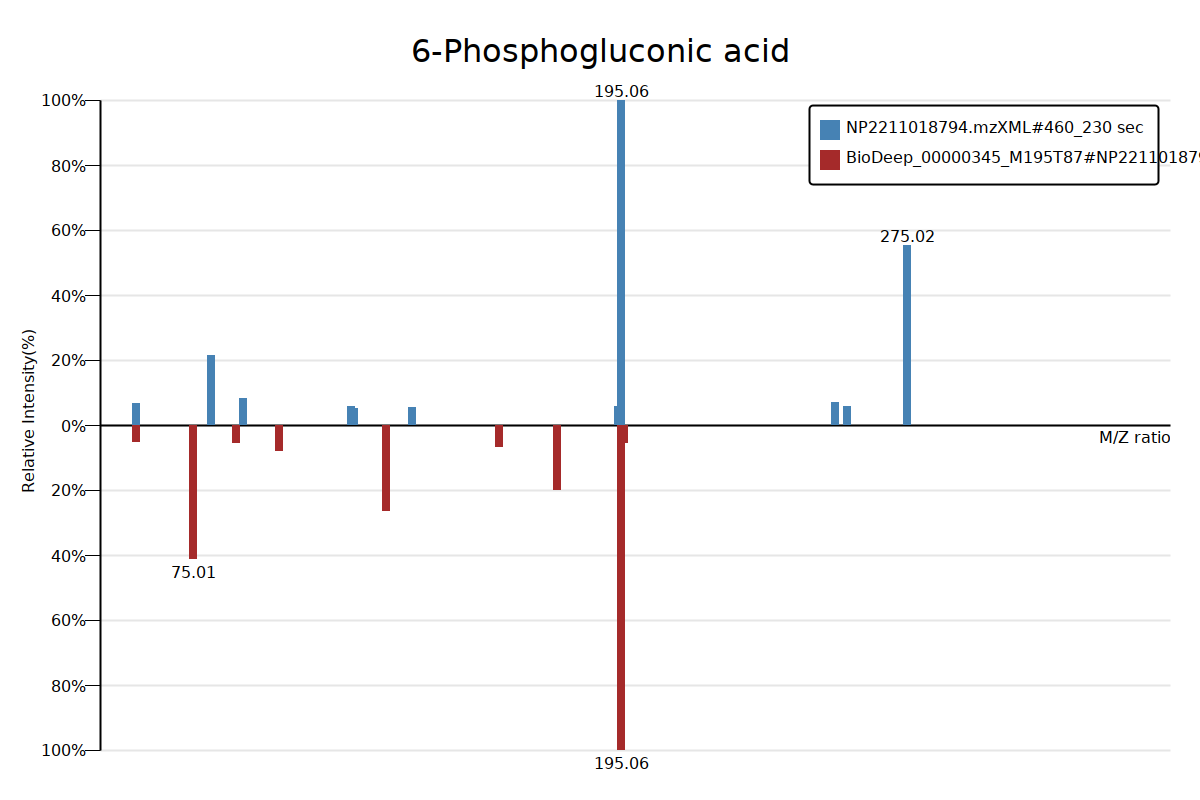

Supplement: Supplementary file 5 [file DataSheet1.ZIP › 2 result graphs between the MSMS secondary fragments of each metabolite and the MSMS secondary fragments of the standard substance in the database/6-Phosphogluconic acid.png]

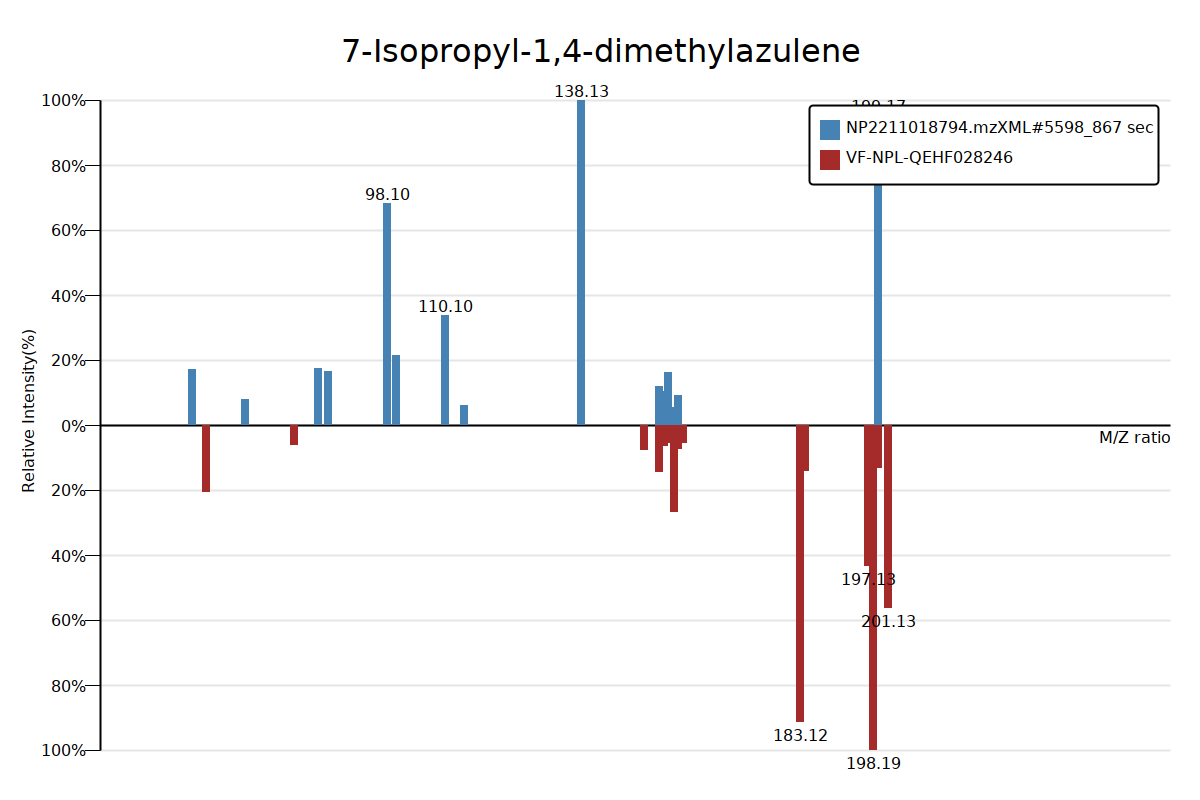

Supplement: Supplementary file 5 [file DataSheet1.ZIP › 2 result graphs between the MSMS secondary fragments of each metabolite and the MSMS secondary fragments of the standard substance in the database/7-Isopropyl-1,4-dimethylazulene.png]

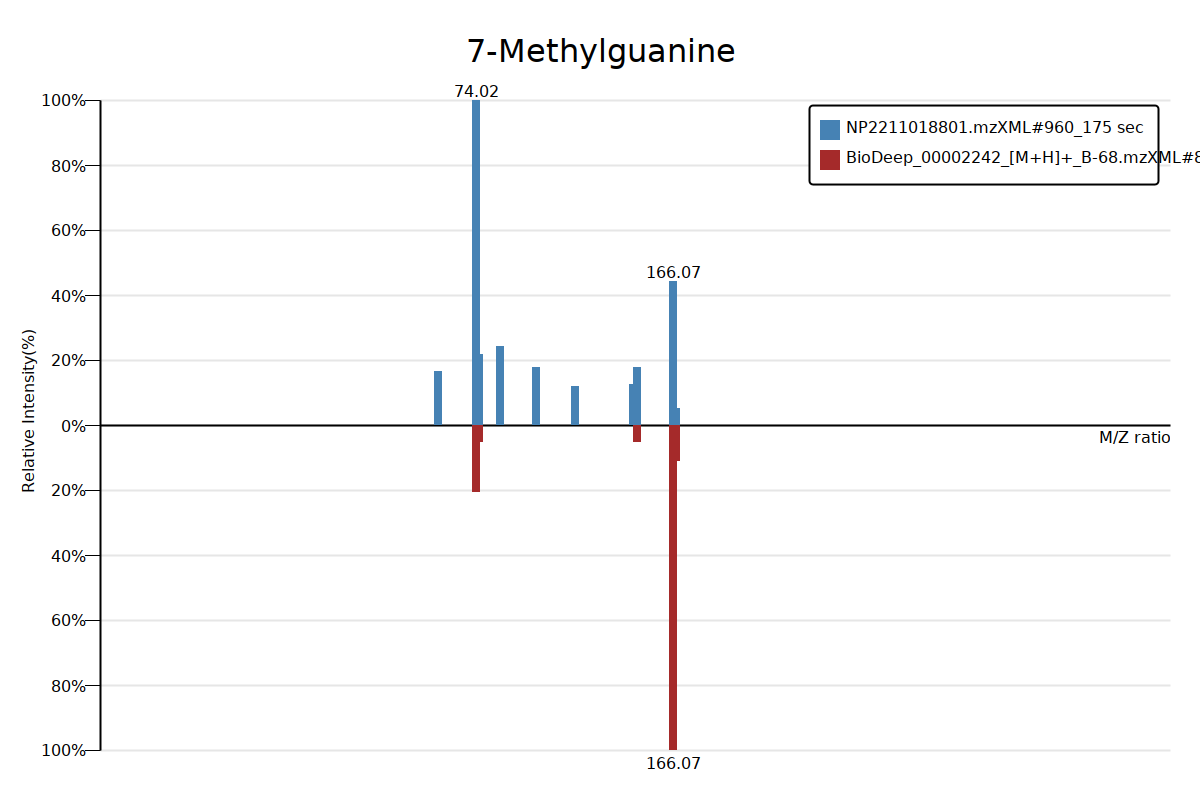

Supplement: Supplementary file 5 [file DataSheet1.ZIP › 2 result graphs between the MSMS secondary fragments of each metabolite and the MSMS secondary fragments of the standard substance in the database/7-Methylguanine.png]

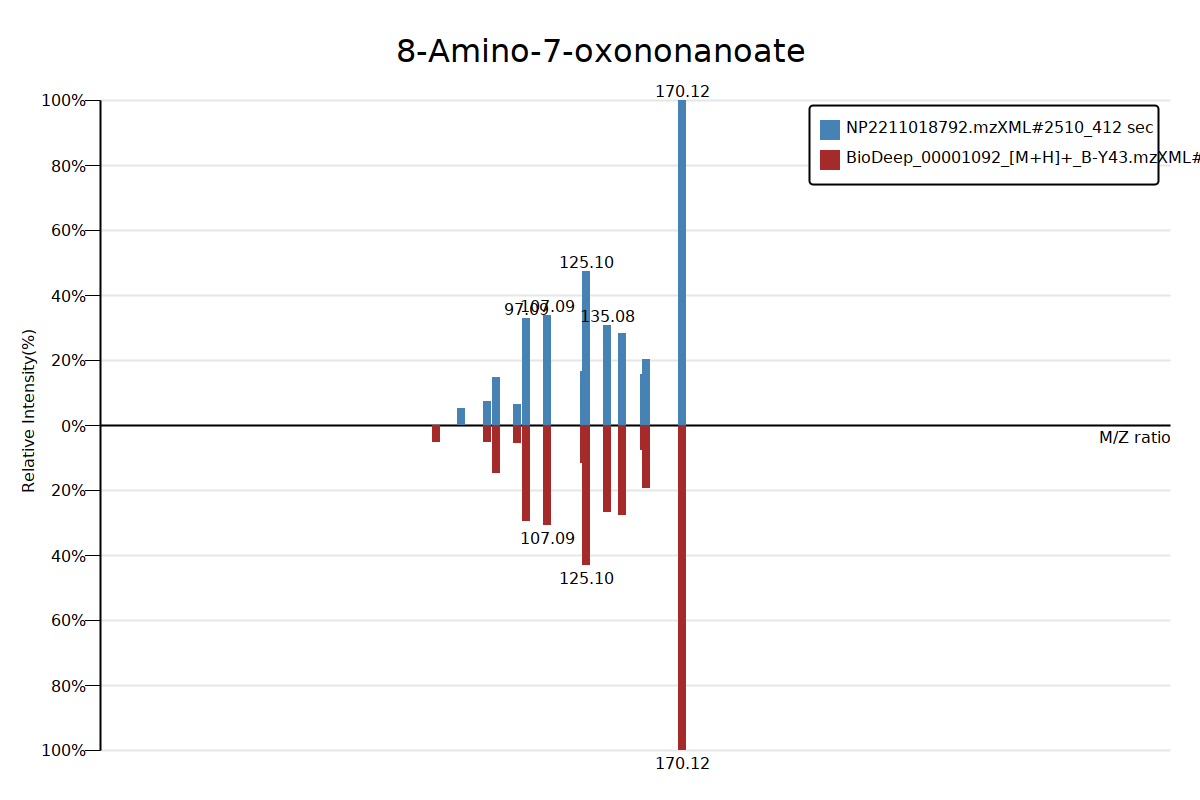

Supplement: Supplementary file 5 [file DataSheet1.ZIP › 2 result graphs between the MSMS secondary fragments of each metabolite and the MSMS secondary fragments of the standard substance in the database/8-Amino-7-oxononanoate.png]

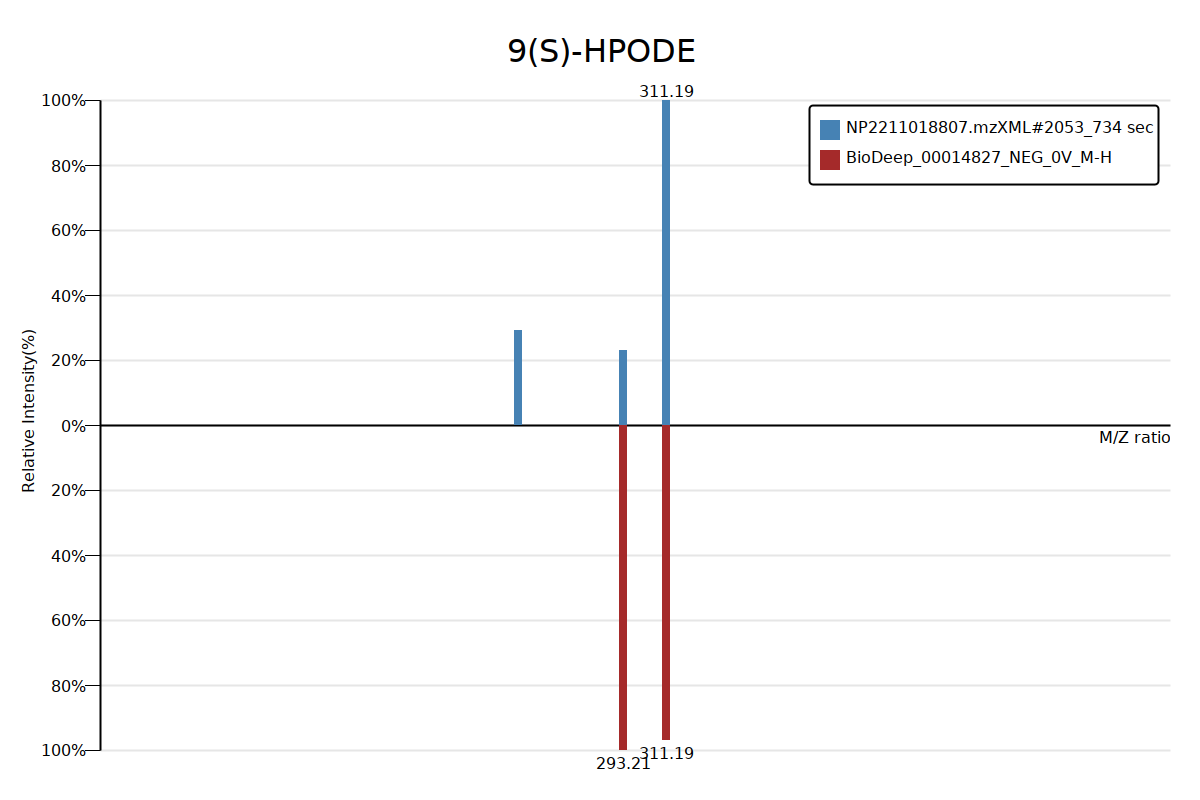

Supplement: Supplementary file 5 [file DataSheet1.ZIP › 2 result graphs between the MSMS secondary fragments of each metabolite and the MSMS secondary fragments of the standard substance in the database/9(S)-HPODE.png]

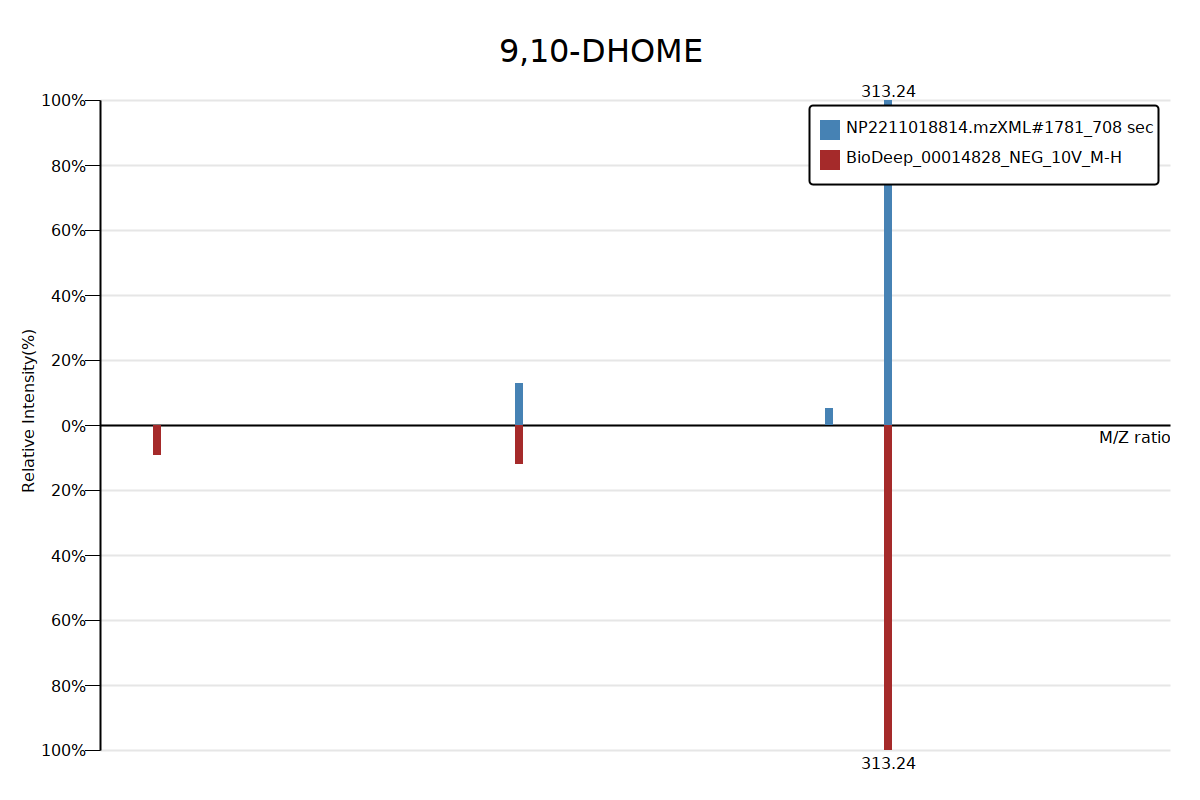

Supplement: Supplementary file 5 [file DataSheet1.ZIP › 2 result graphs between the MSMS secondary fragments of each metabolite and the MSMS secondary fragments of the standard substance in the database/9,10-DHOME.png]

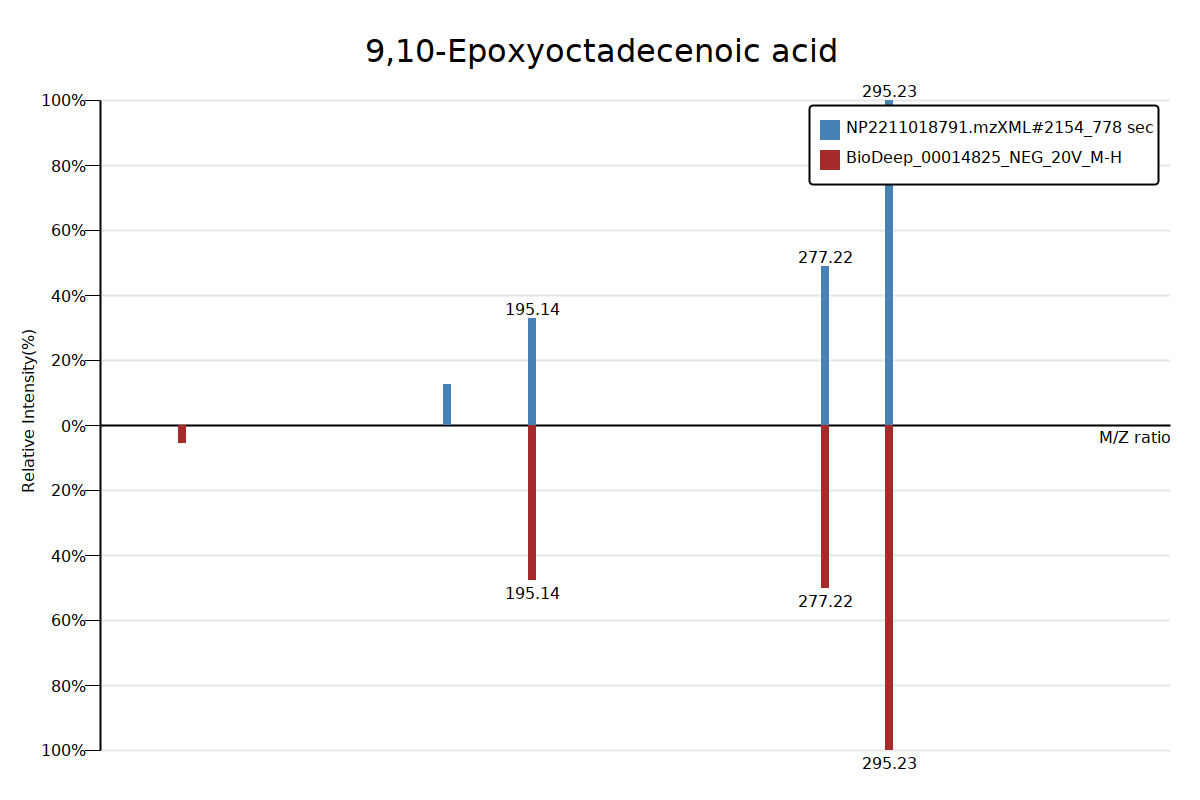

Supplement: Supplementary file 5 [file DataSheet1.ZIP › 2 result graphs between the MSMS secondary fragments of each metabolite and the MSMS secondary fragments of the standard substance in the database/9,10-Epoxyoctadecenoic acid.png]

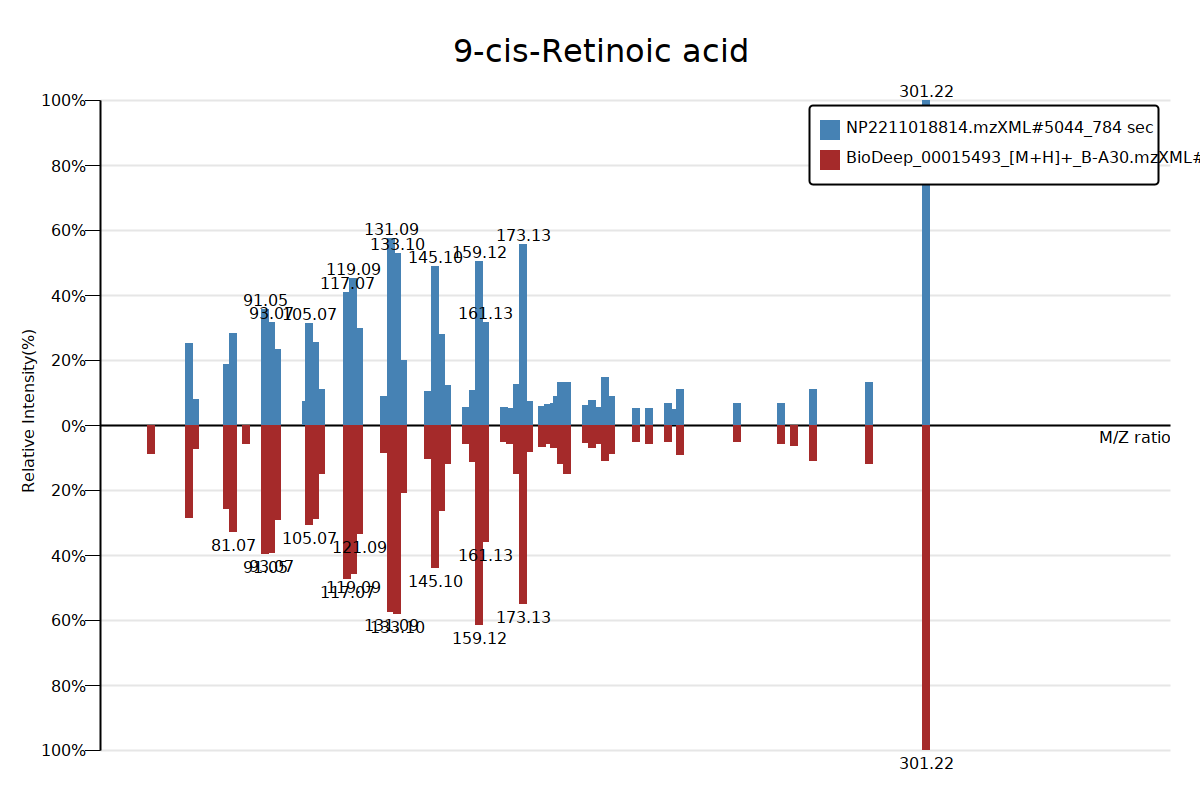

Supplement: Supplementary file 5 [file DataSheet1.ZIP › 2 result graphs between the MSMS secondary fragments of each metabolite and the MSMS secondary fragments of the standard substance in the database/9-cis-Retinoic acid.png]

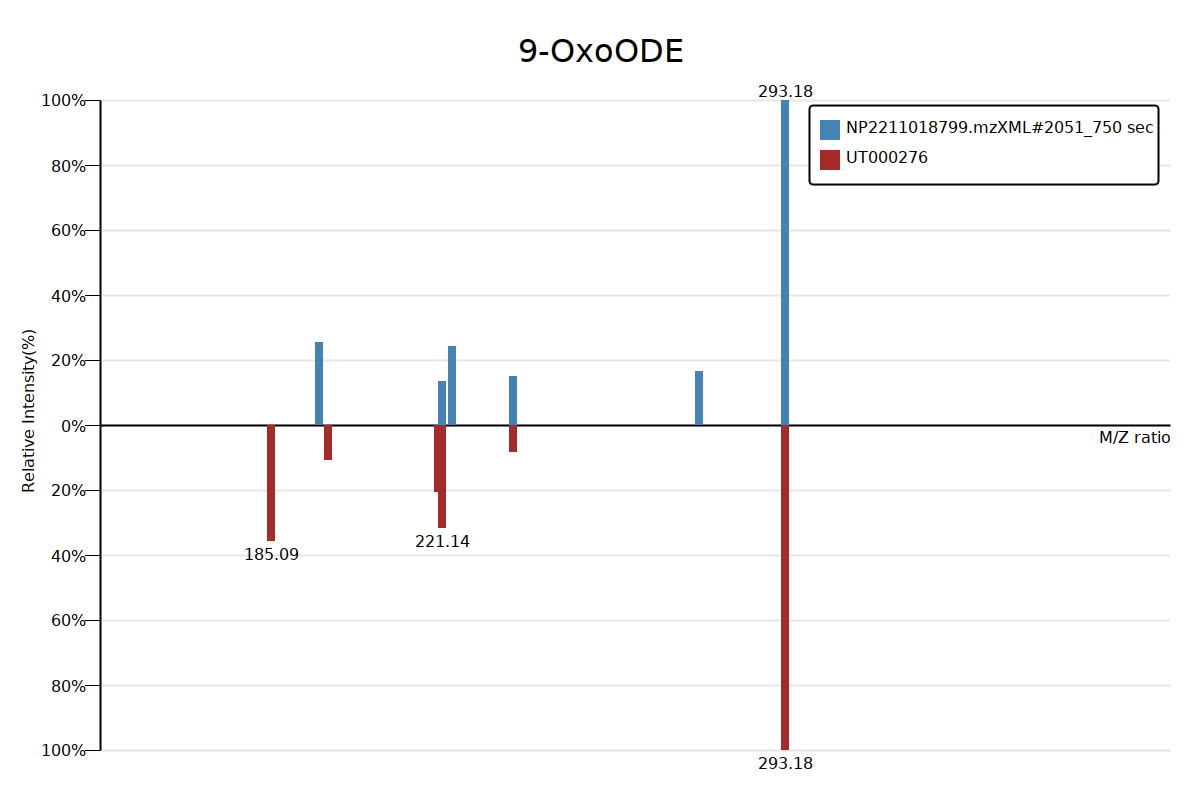

Supplement: Supplementary file 5 [file DataSheet1.ZIP › 2 result graphs between the MSMS secondary fragments of each metabolite and the MSMS secondary fragments of the standard substance in the database/9-OxoODE.png]

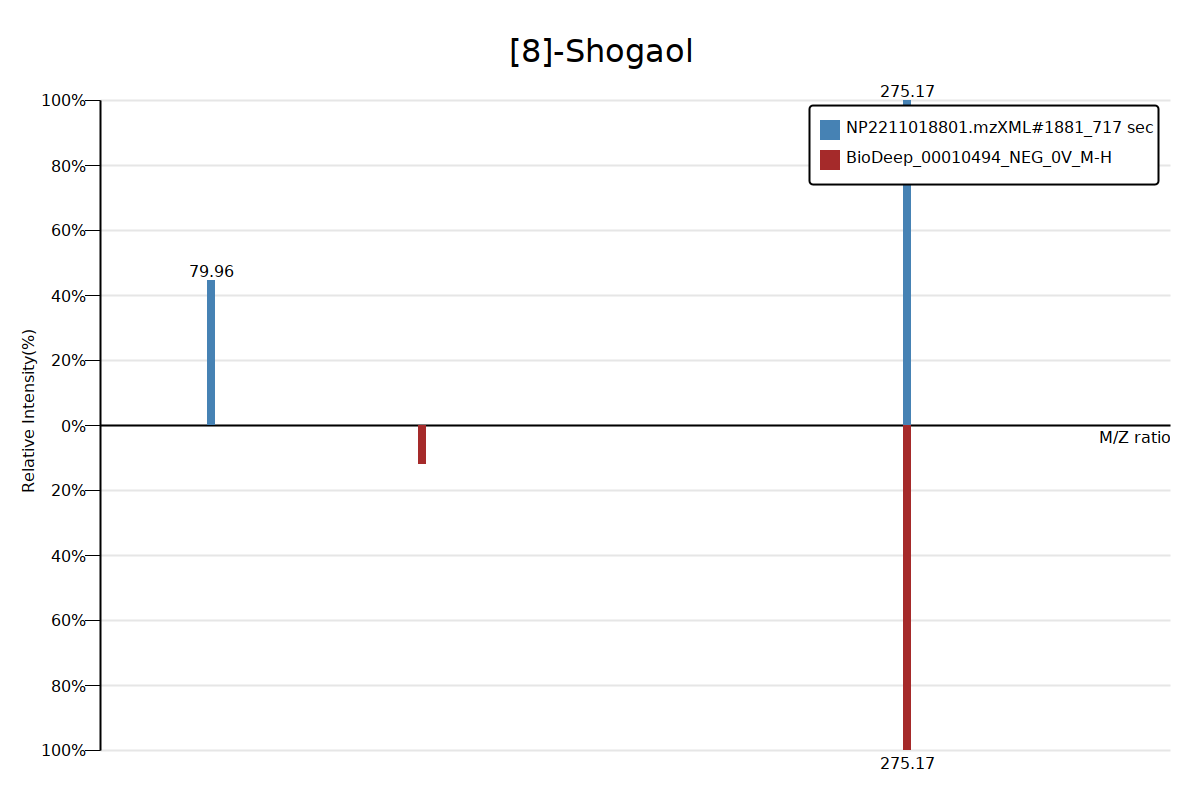

Supplement: Supplementary file 5 [file DataSheet1.ZIP › 2 result graphs between the MSMS secondary fragments of each metabolite and the MSMS secondary fragments of the standard substance in the database/[8]-Shogaol.png]

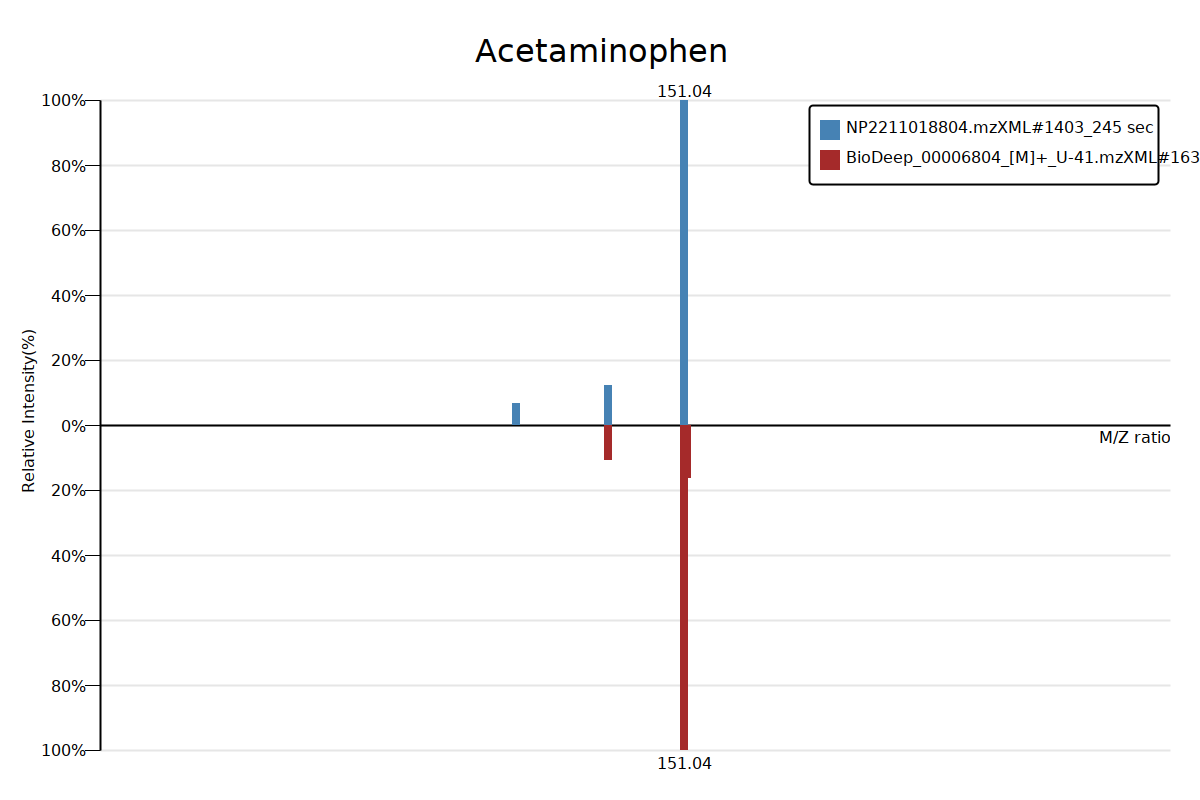

Supplement: Supplementary file 5 [file DataSheet1.ZIP › 2 result graphs between the MSMS secondary fragments of each metabolite and the MSMS secondary fragments of the standard substance in the database/Acetaminophen.png]

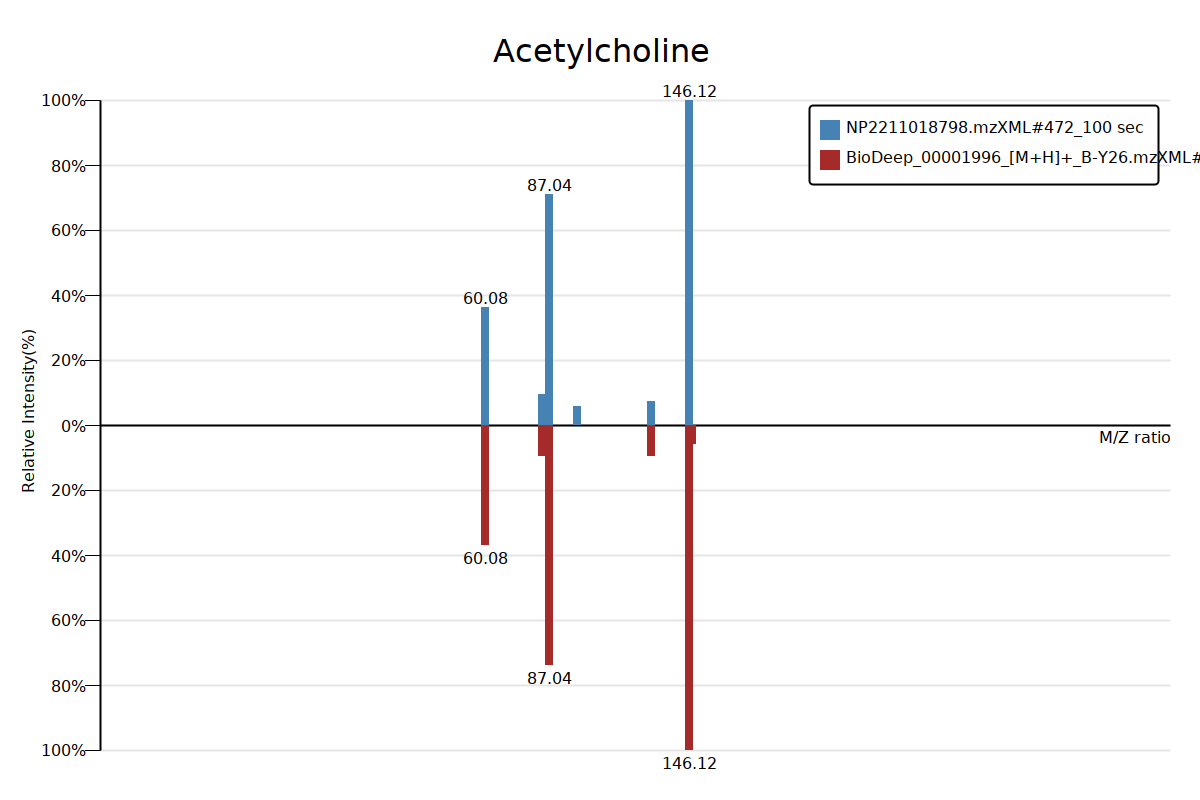

Supplement: Supplementary file 5 [file DataSheet1.ZIP › 2 result graphs between the MSMS secondary fragments of each metabolite and the MSMS secondary fragments of the standard substance in the database/Acetylcholine .png]

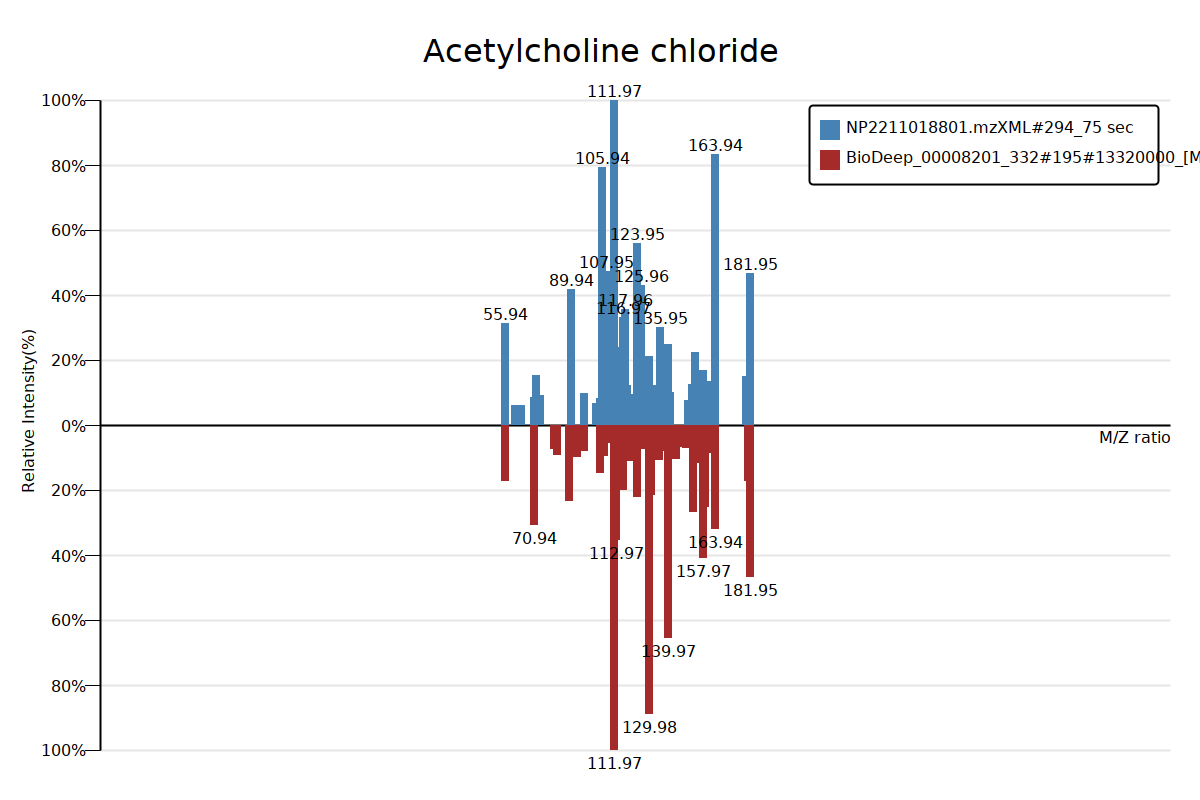

Supplement: Supplementary file 5 [file DataSheet1.ZIP › 2 result graphs between the MSMS secondary fragments of each metabolite and the MSMS secondary fragments of the standard substance in the database/Acetylcholine chloride.png]

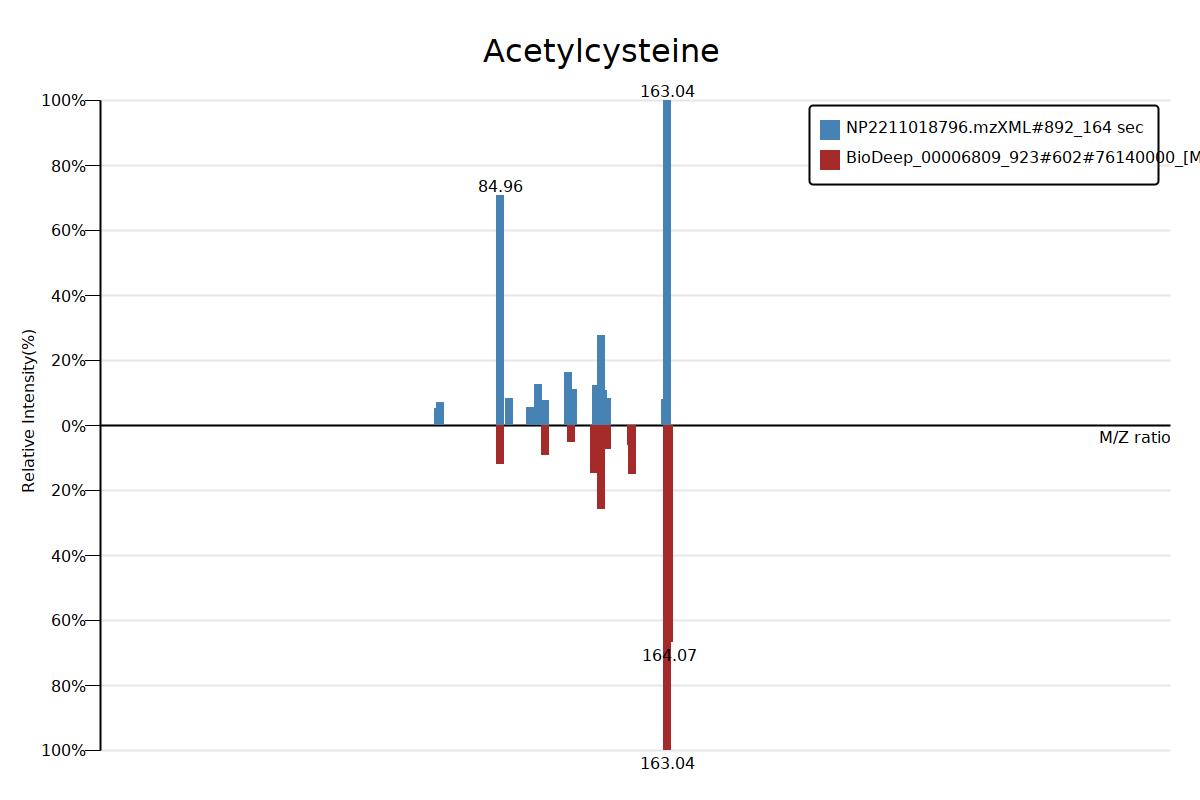

Supplement: Supplementary file 5 [file DataSheet1.ZIP › 2 result graphs between the MSMS secondary fragments of each metabolite and the MSMS secondary fragments of the standard substance in the database/Acetylcysteine.png]

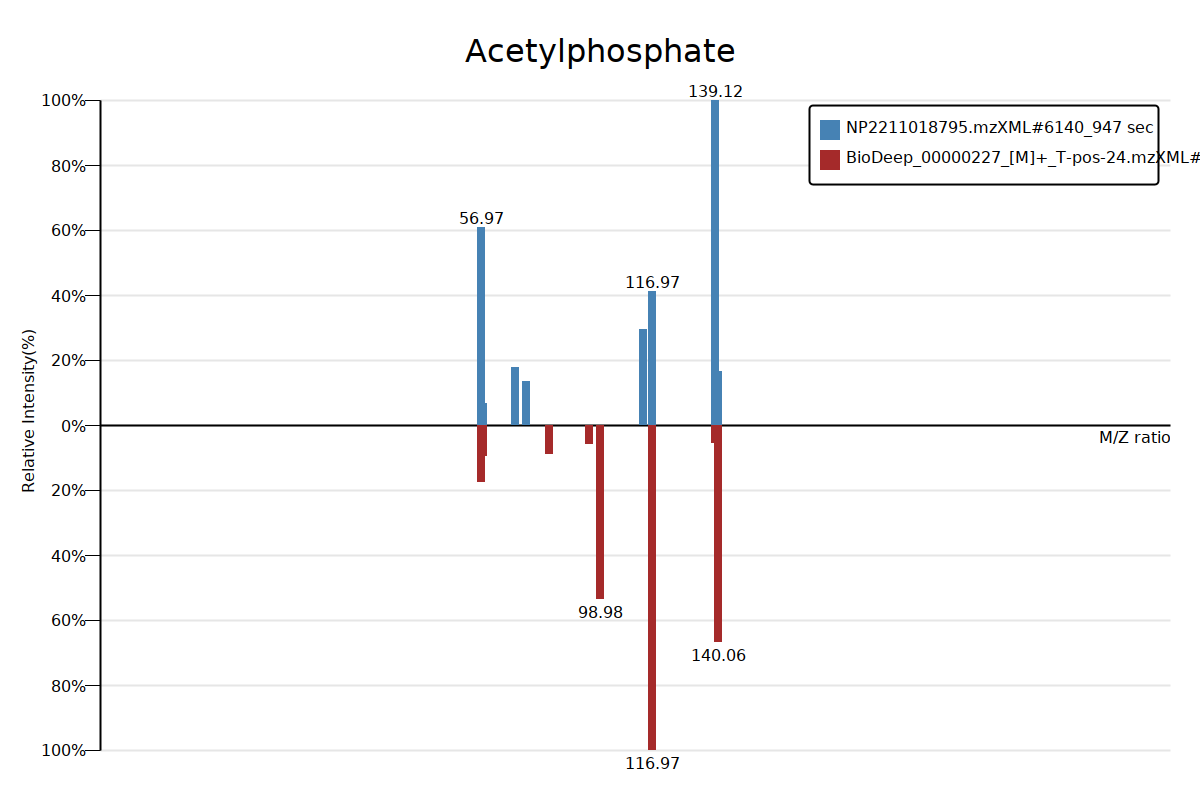

Supplement: Supplementary file 5 [file DataSheet1.ZIP › 2 result graphs between the MSMS secondary fragments of each metabolite and the MSMS secondary fragments of the standard substance in the database/Acetylphosphate.png]

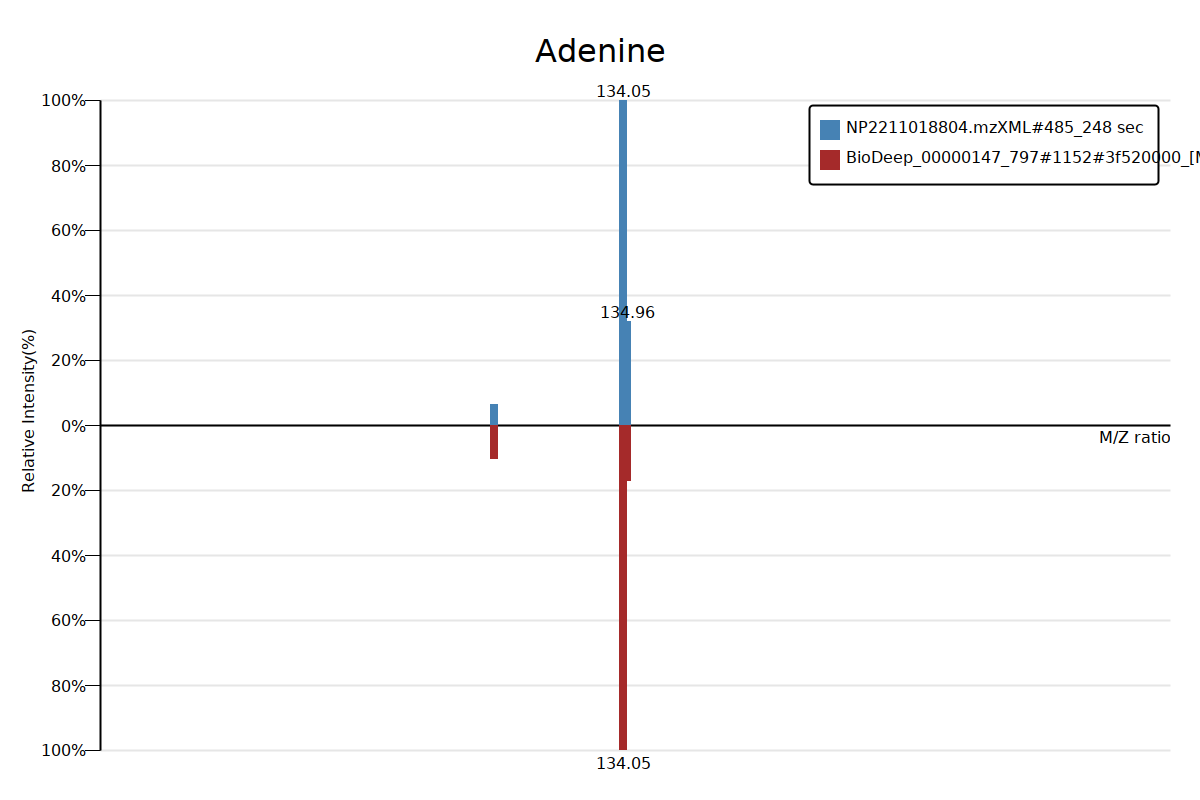

Supplement: Supplementary file 5 [file DataSheet1.ZIP › 2 result graphs between the MSMS secondary fragments of each metabolite and the MSMS secondary fragments of the standard substance in the database/Adenine.png]

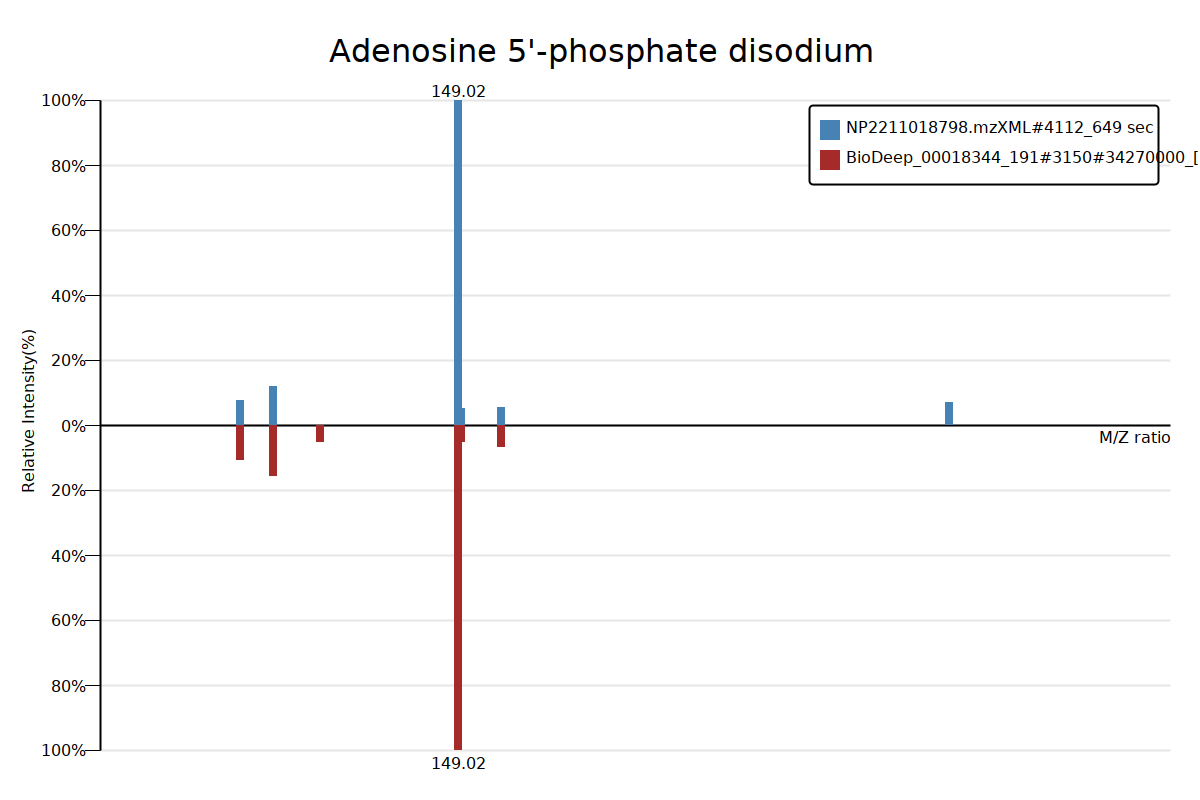

Supplement: Supplementary file 5 [file DataSheet1.ZIP › 2 result graphs between the MSMS secondary fragments of each metabolite and the MSMS secondary fragments of the standard substance in the database/Adenosine 5'-phosphate disodium.png]

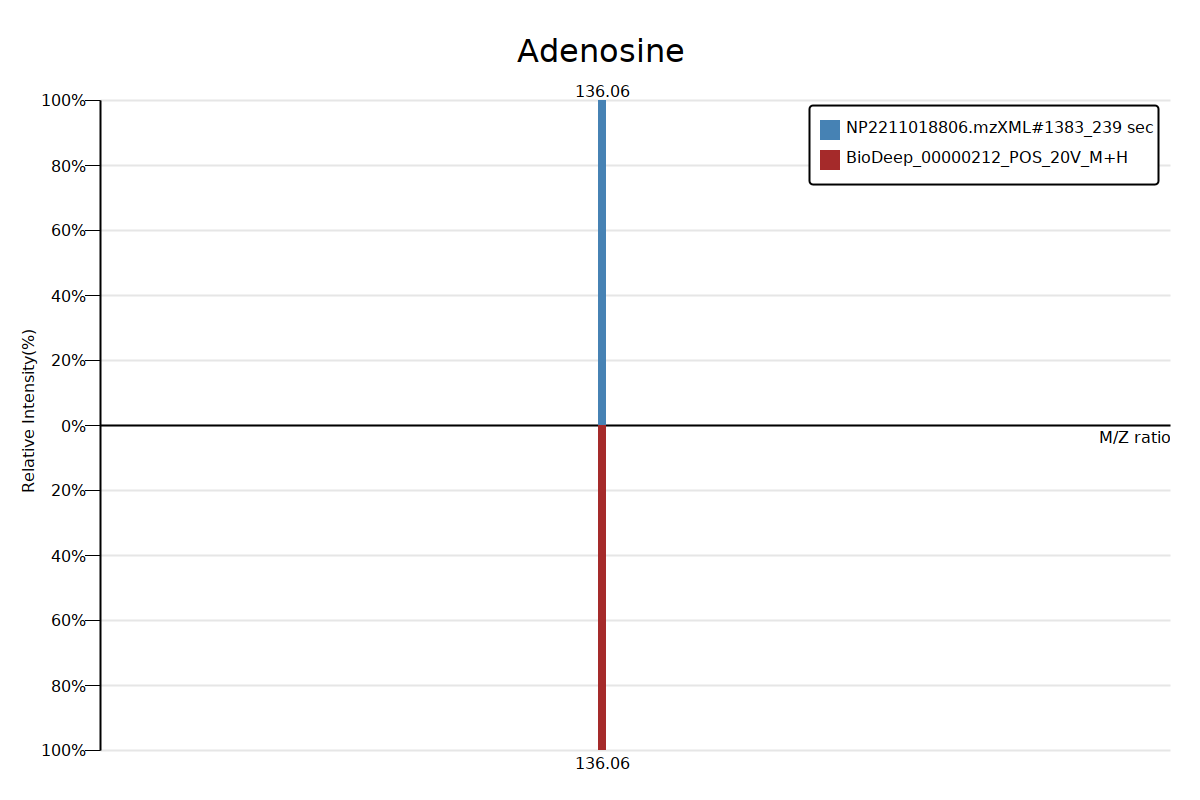

Supplement: Supplementary file 5 [file DataSheet1.ZIP › 2 result graphs between the MSMS secondary fragments of each metabolite and the MSMS secondary fragments of the standard substance in the database/Adenosine.png]

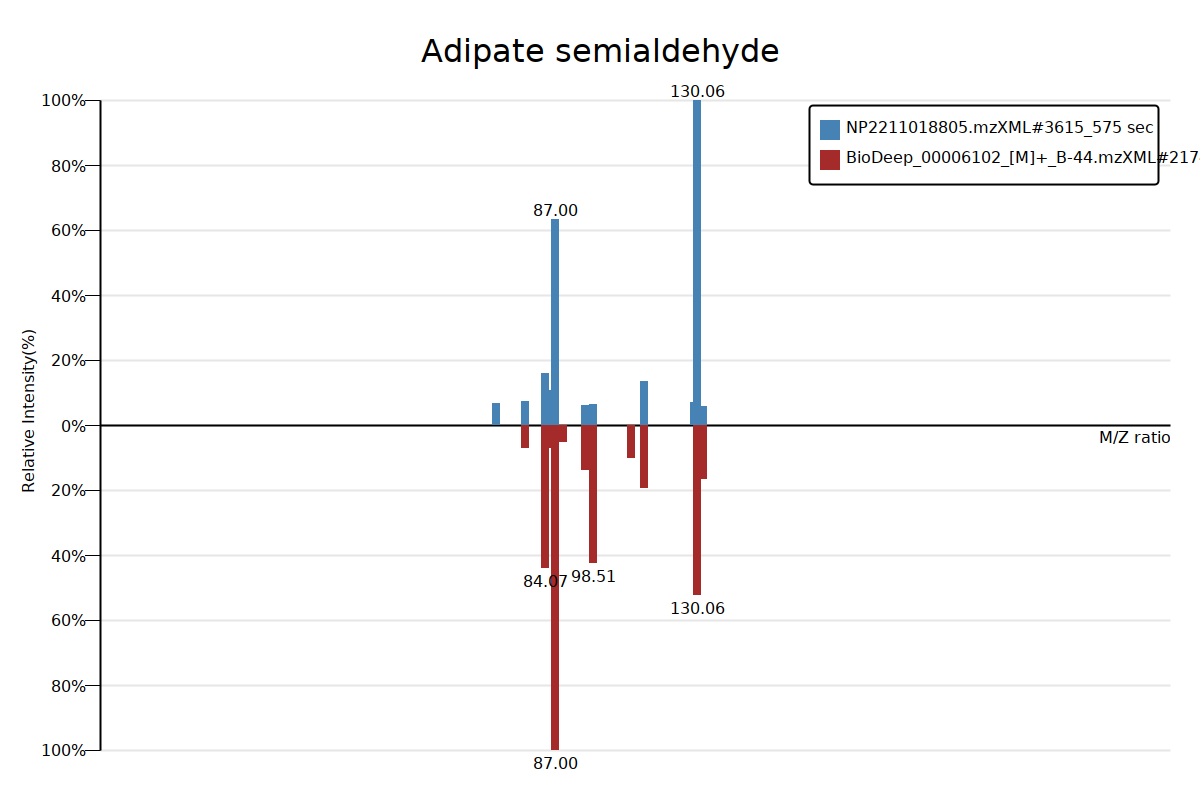

Supplement: Supplementary file 5 [file DataSheet1.ZIP › 2 result graphs between the MSMS secondary fragments of each metabolite and the MSMS secondary fragments of the standard substance in the database/Adipate semialdehyde.png]

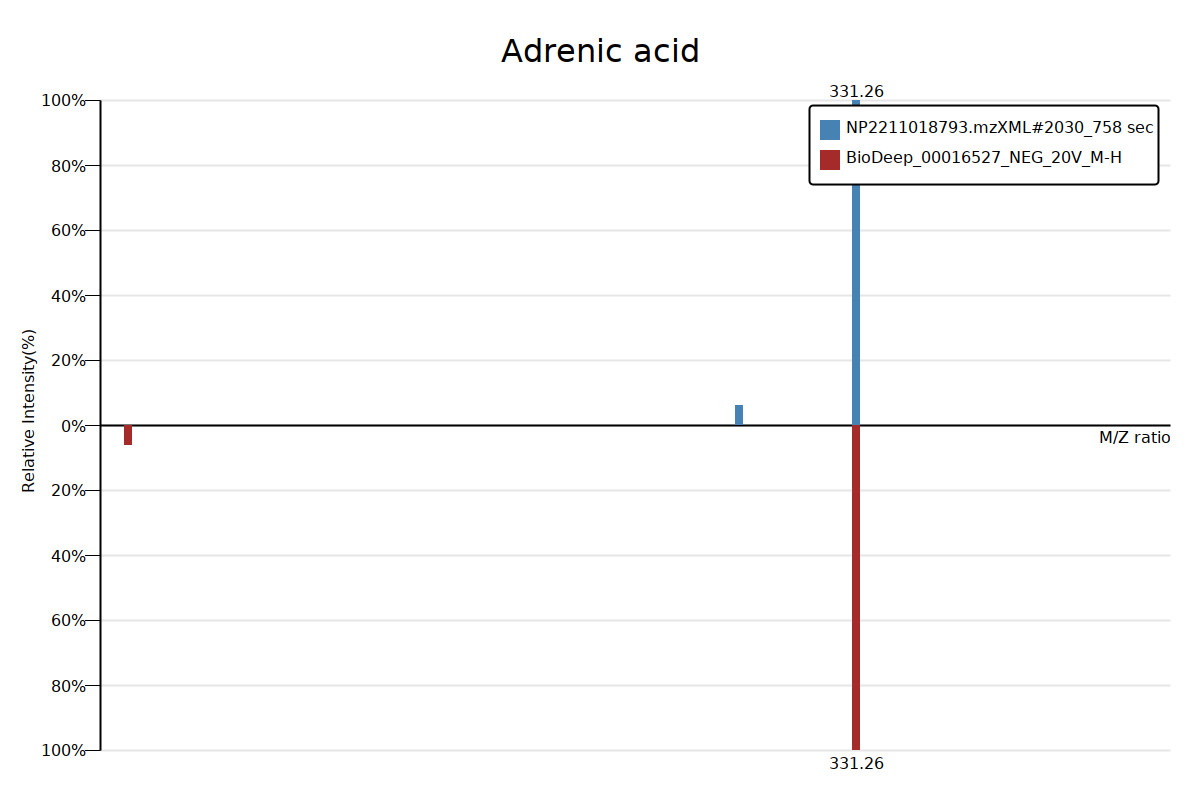

Supplement: Supplementary file 5 [file DataSheet1.ZIP › 2 result graphs between the MSMS secondary fragments of each metabolite and the MSMS secondary fragments of the standard substance in the database/Adrenic acid.png]

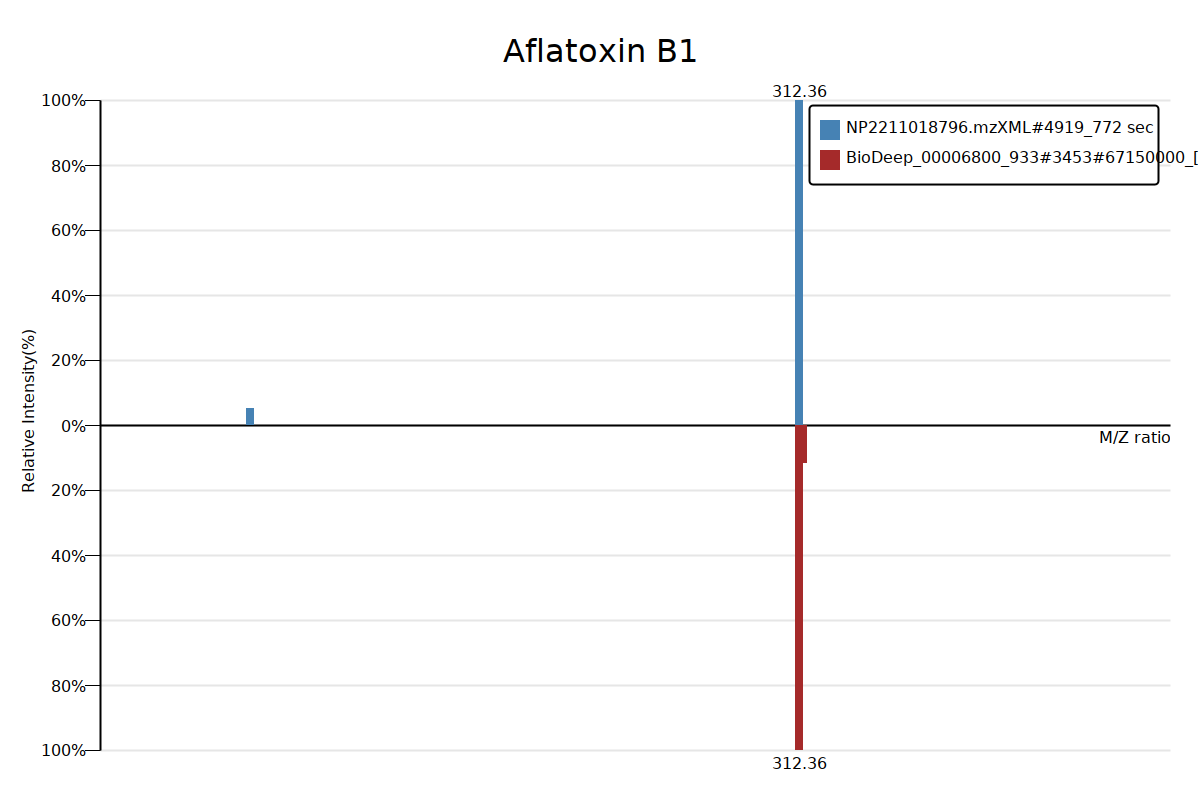

Supplement: Supplementary file 5 [file DataSheet1.ZIP › 2 result graphs between the MSMS secondary fragments of each metabolite and the MSMS secondary fragments of the standard substance in the database/Aflatoxin B1.png]

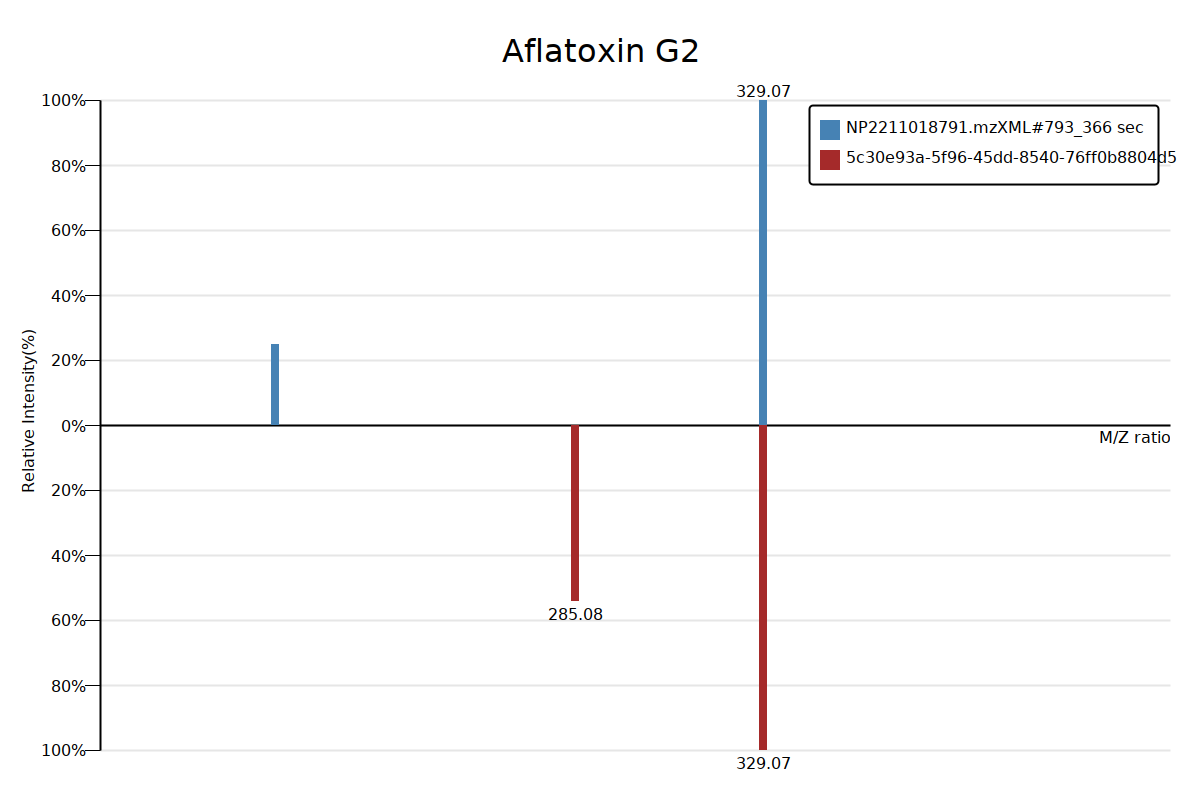

Supplement: Supplementary file 5 [file DataSheet1.ZIP › 2 result graphs between the MSMS secondary fragments of each metabolite and the MSMS secondary fragments of the standard substance in the database/Aflatoxin G2.png]

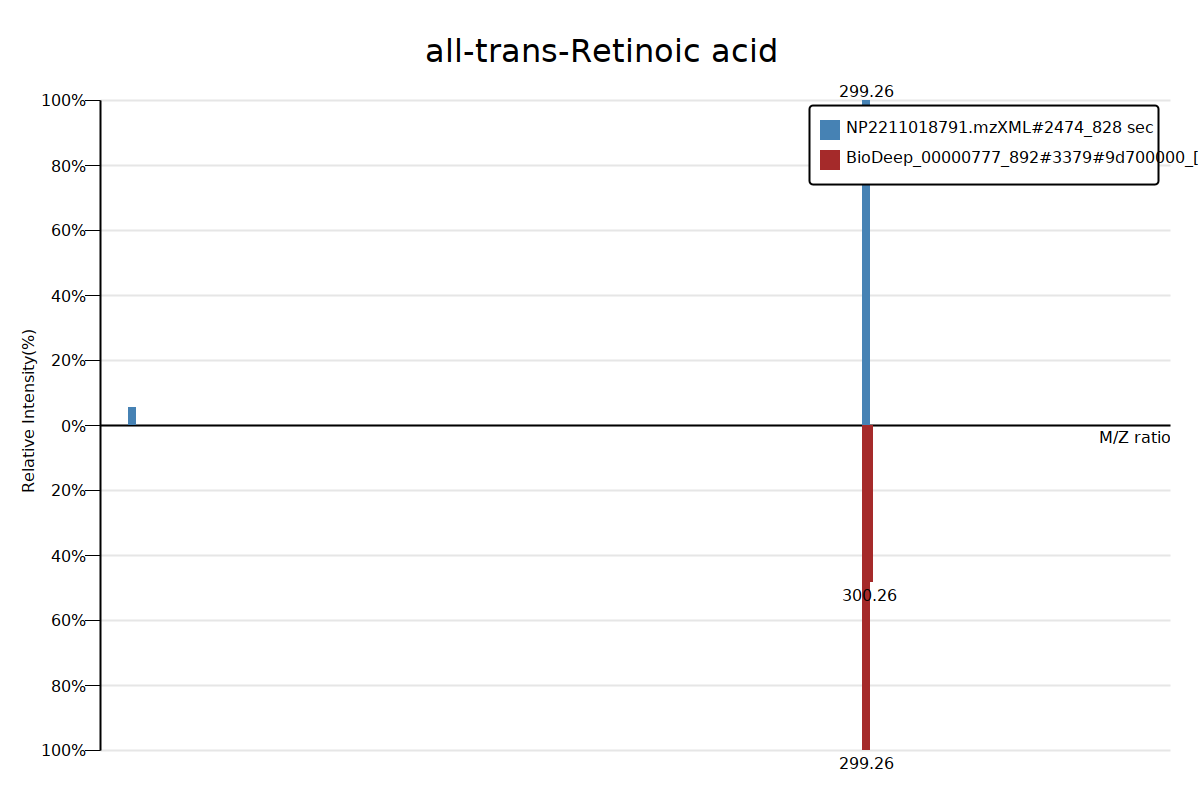

Supplement: Supplementary file 5 [file DataSheet1.ZIP › 2 result graphs between the MSMS secondary fragments of each metabolite and the MSMS secondary fragments of the standard substance in the database/all-trans-Retinoic acid.png]

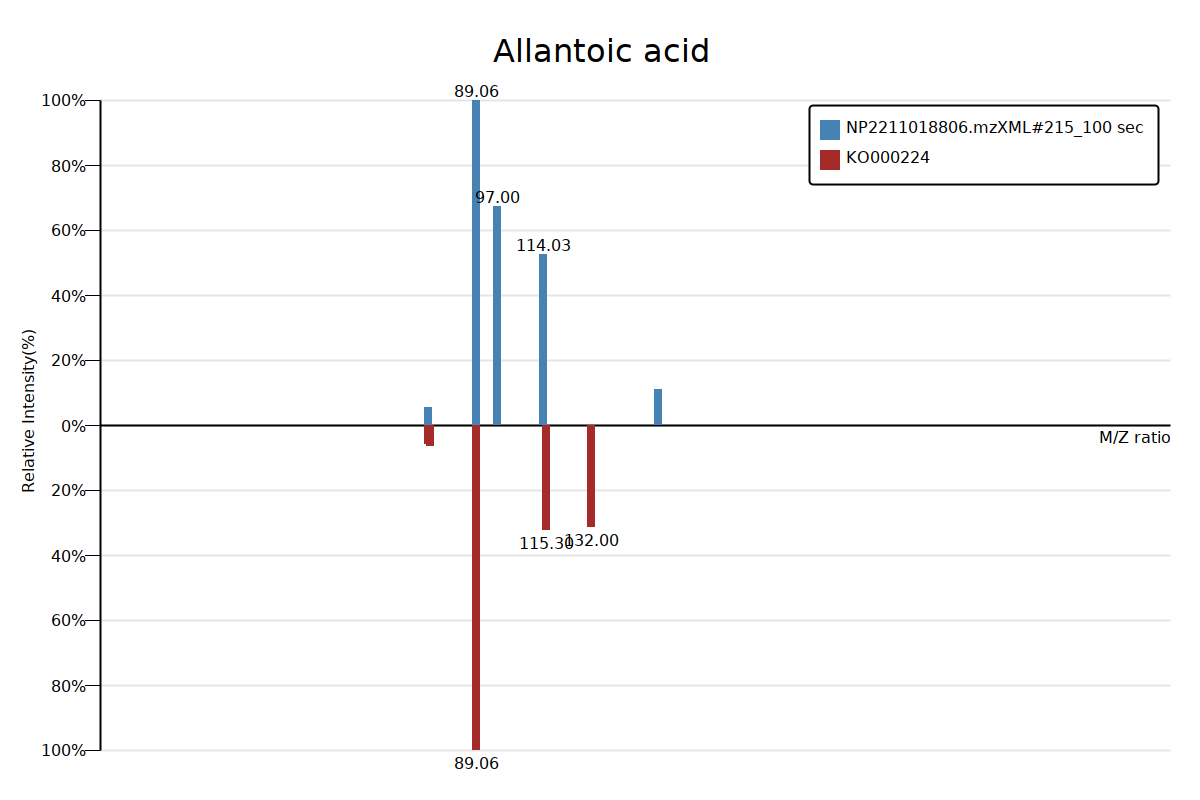

Supplement: Supplementary file 5 [file DataSheet1.ZIP › 2 result graphs between the MSMS secondary fragments of each metabolite and the MSMS secondary fragments of the standard substance in the database/Allantoic acid.png]

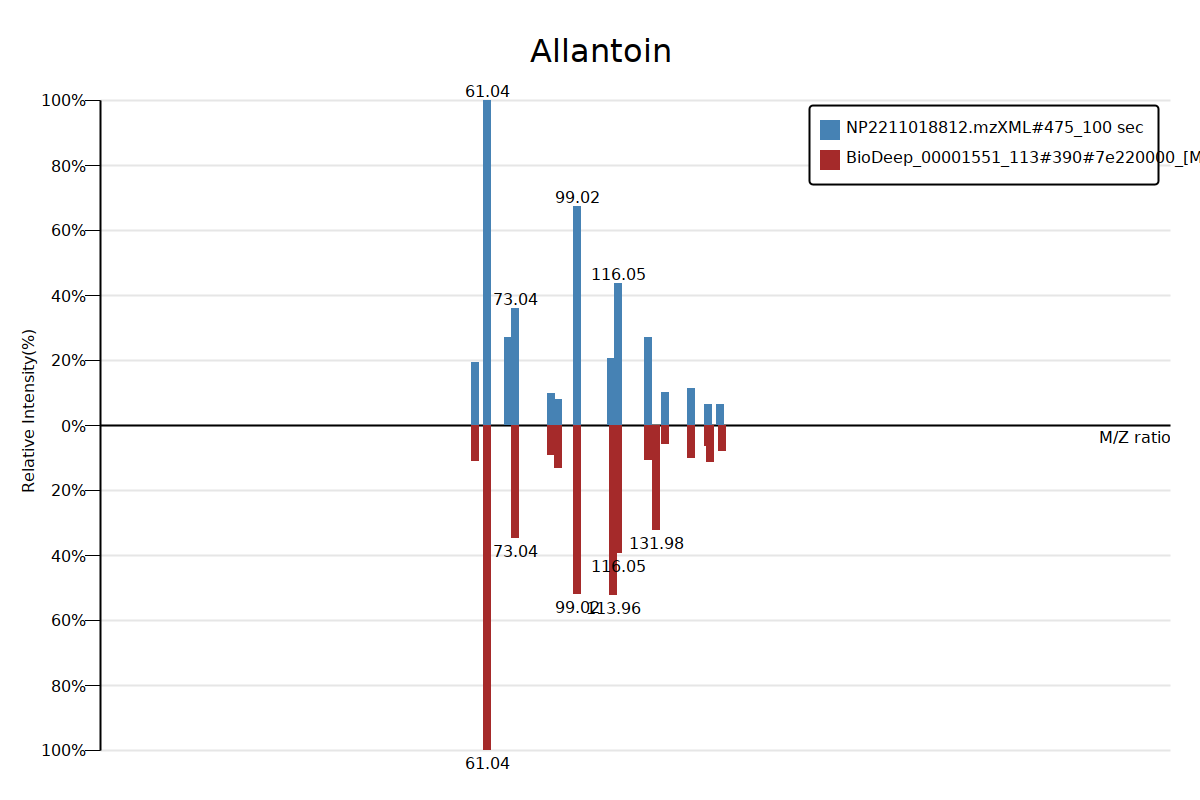

Supplement: Supplementary file 5 [file DataSheet1.ZIP › 2 result graphs between the MSMS secondary fragments of each metabolite and the MSMS secondary fragments of the standard substance in the database/Allantoin.png]

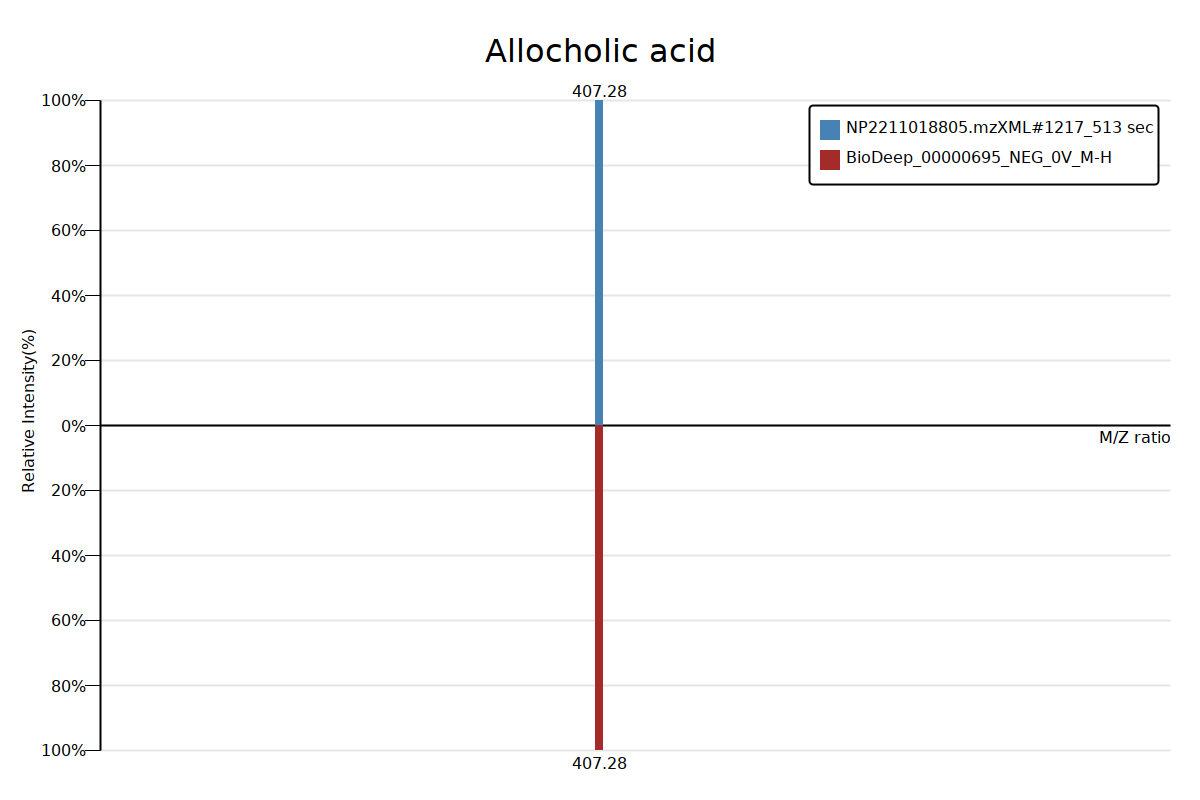

Supplement: Supplementary file 5 [file DataSheet1.ZIP › 2 result graphs between the MSMS secondary fragments of each metabolite and the MSMS secondary fragments of the standard substance in the database/Allocholic acid.png]

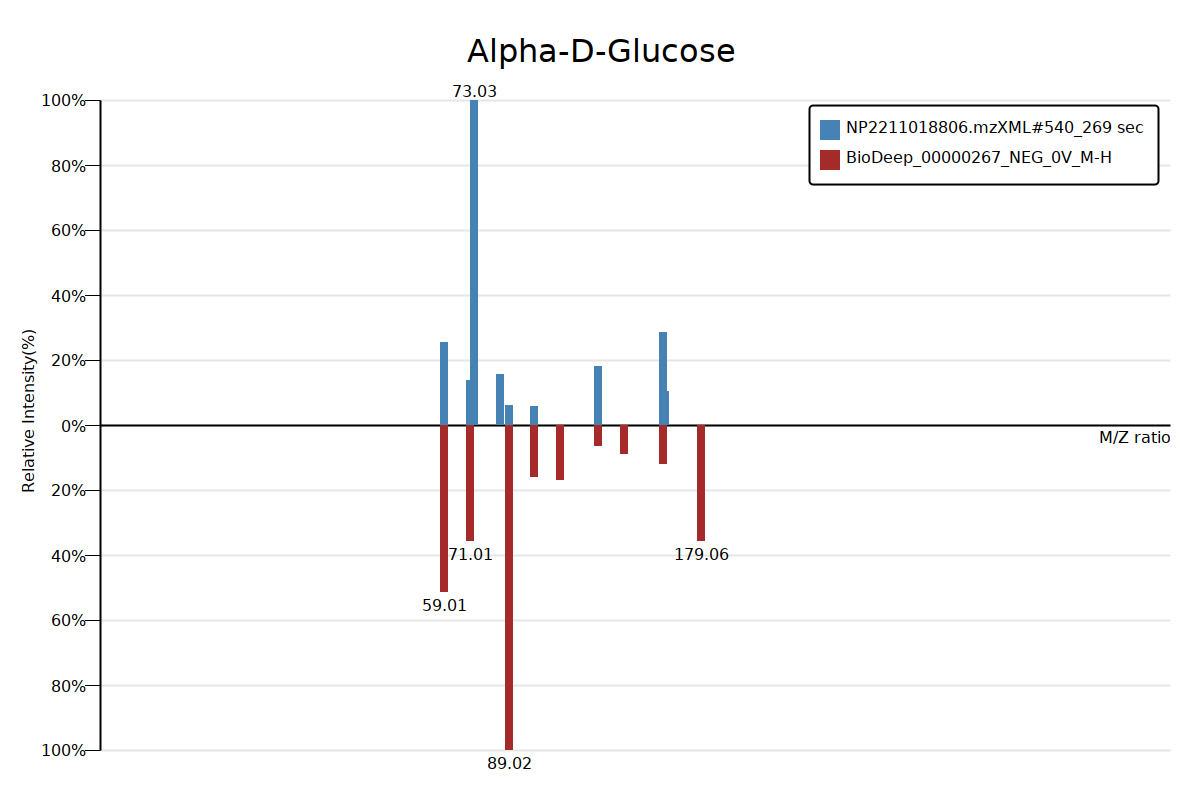

Supplement: Supplementary file 5 [file DataSheet1.ZIP › 2 result graphs between the MSMS secondary fragments of each metabolite and the MSMS secondary fragments of the standard substance in the database/Alpha-D-Glucose.png]

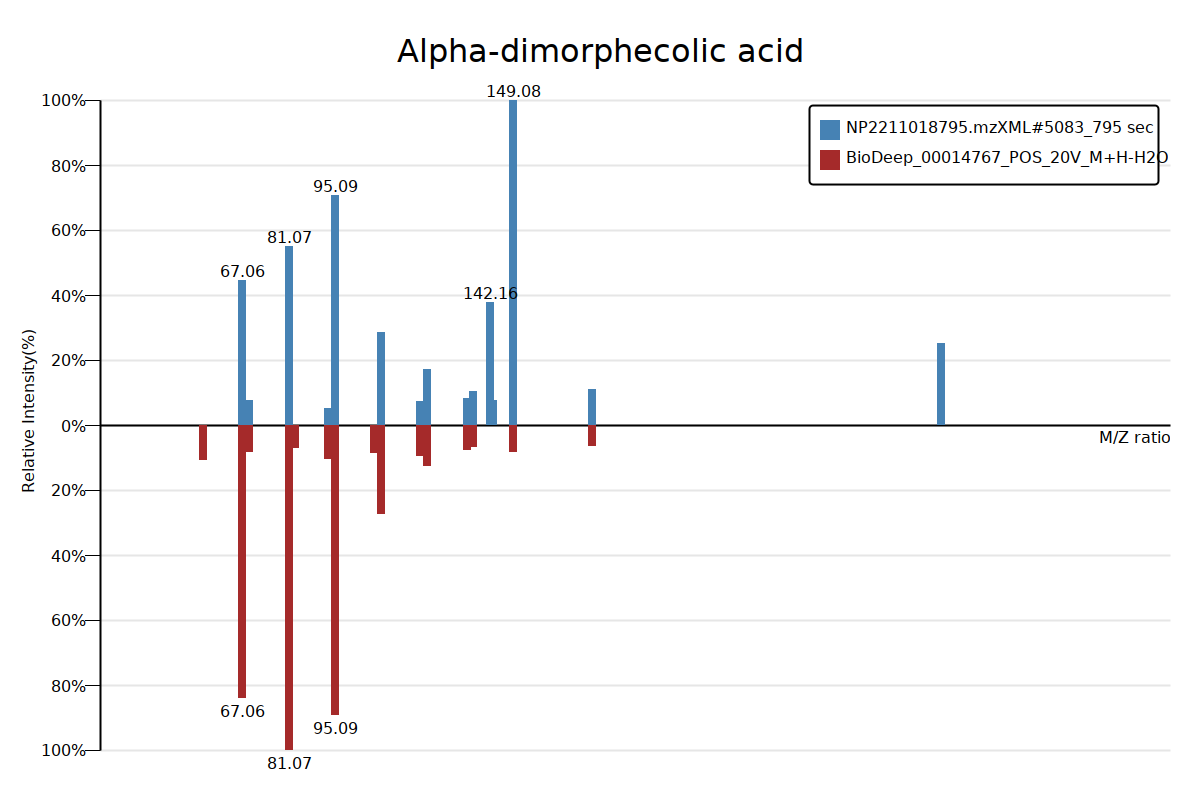

Supplement: Supplementary file 5 [file DataSheet1.ZIP › 2 result graphs between the MSMS secondary fragments of each metabolite and the MSMS secondary fragments of the standard substance in the database/Alpha-dimorphecolic acid.png]

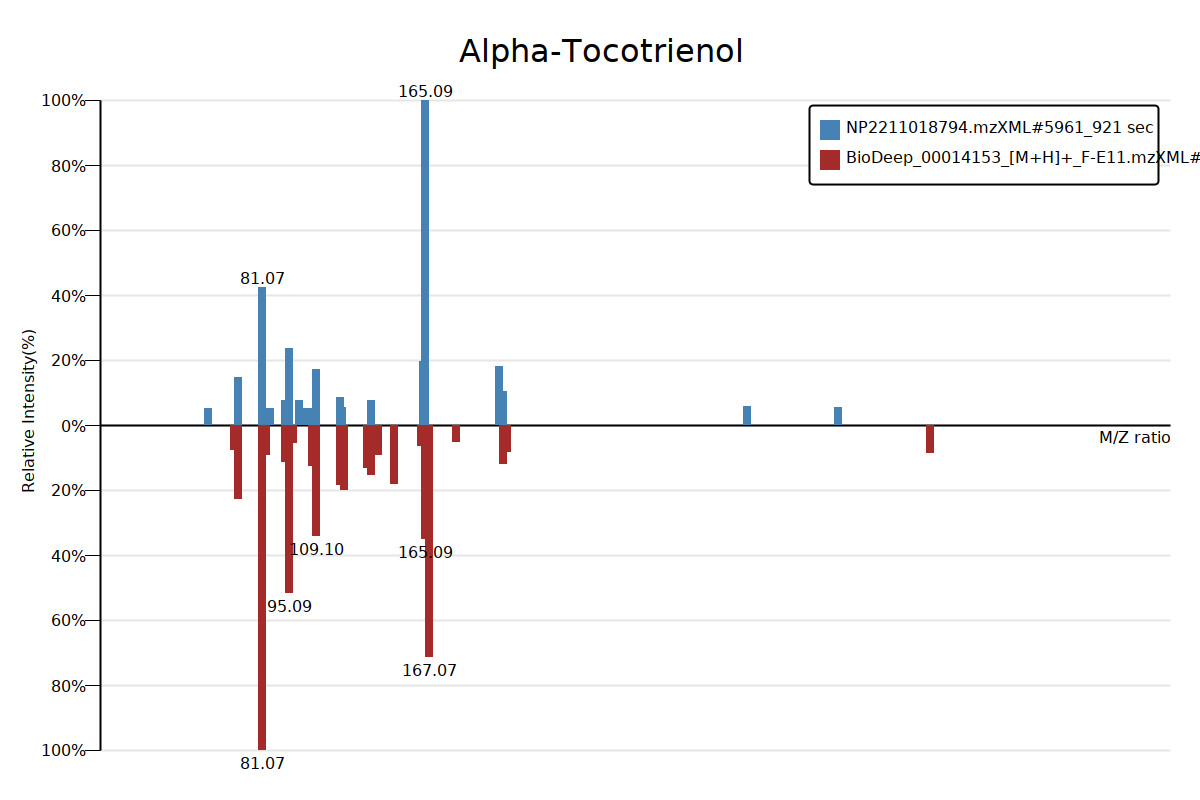

Supplement: Supplementary file 5 [file DataSheet1.ZIP › 2 result graphs between the MSMS secondary fragments of each metabolite and the MSMS secondary fragments of the standard substance in the database/Alpha-Tocotrienol.png]

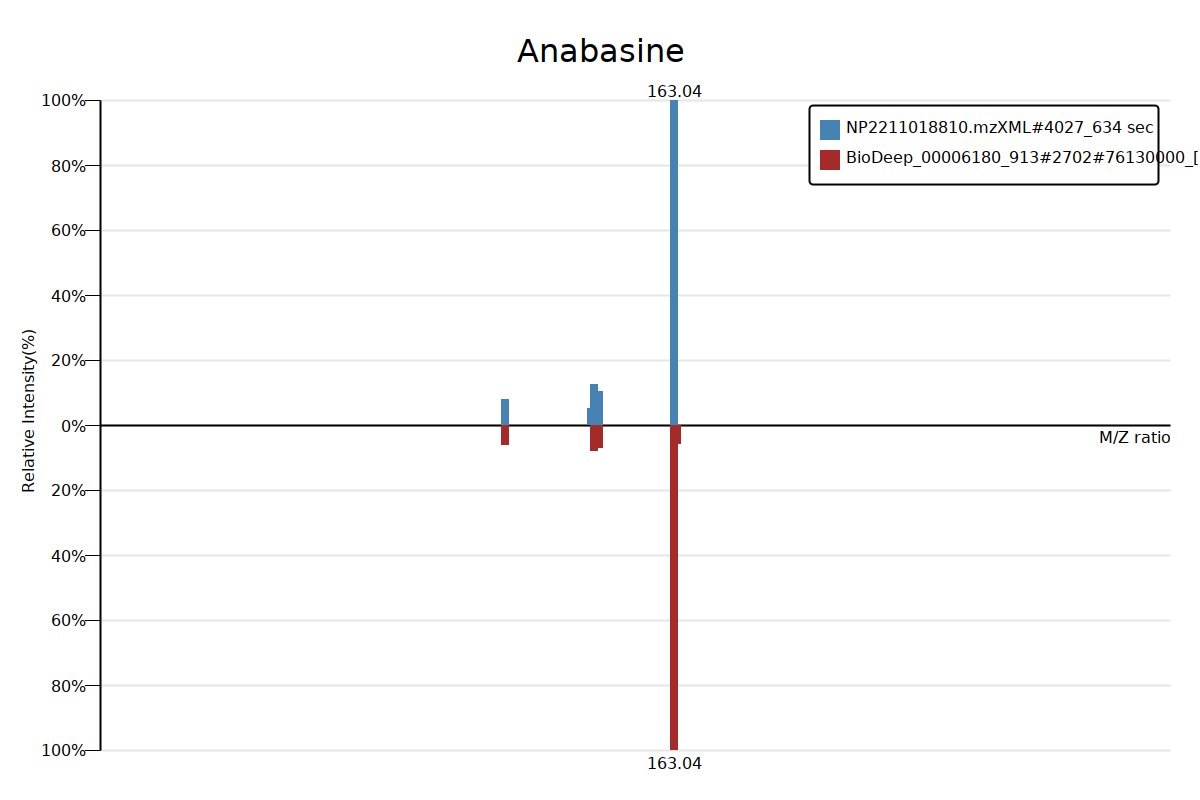

Supplement: Supplementary file 5 [file DataSheet1.ZIP › 2 result graphs between the MSMS secondary fragments of each metabolite and the MSMS secondary fragments of the standard substance in the database/Anabasine.png]

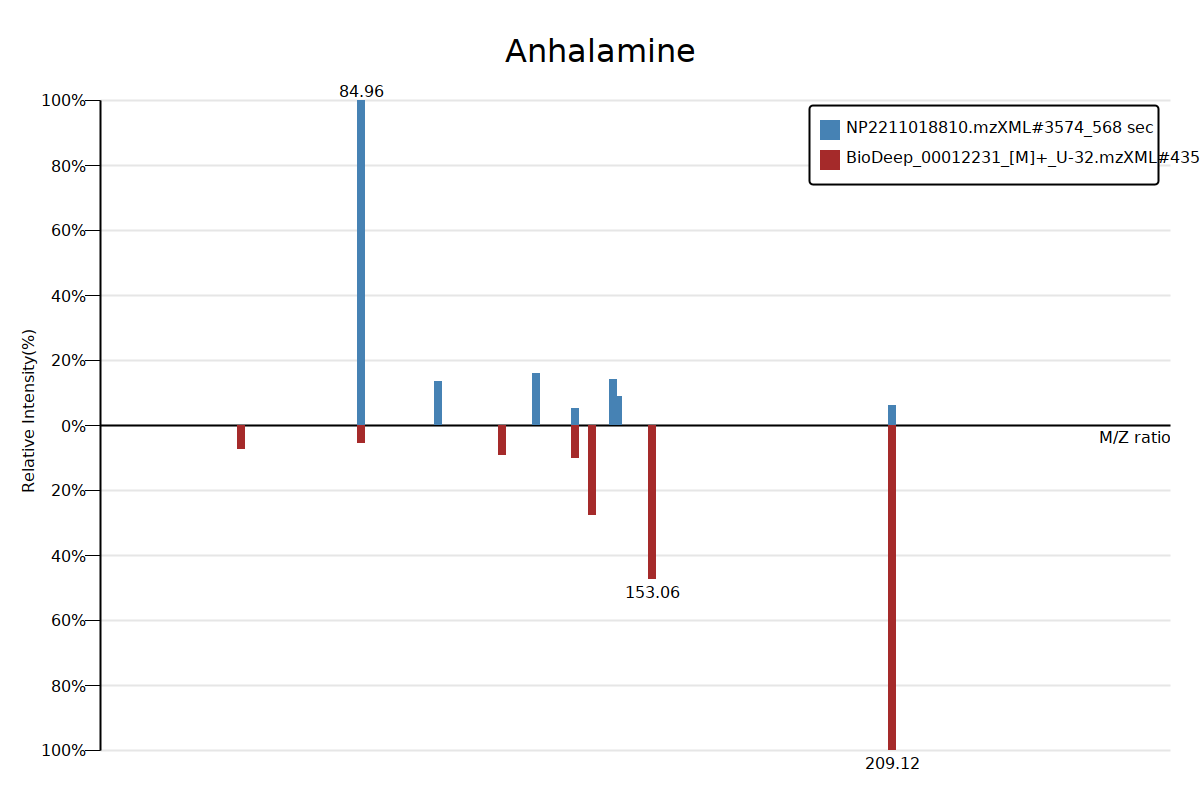

Supplement: Supplementary file 5 [file DataSheet1.ZIP › 2 result graphs between the MSMS secondary fragments of each metabolite and the MSMS secondary fragments of the standard substance in the database/Anhalamine.png]

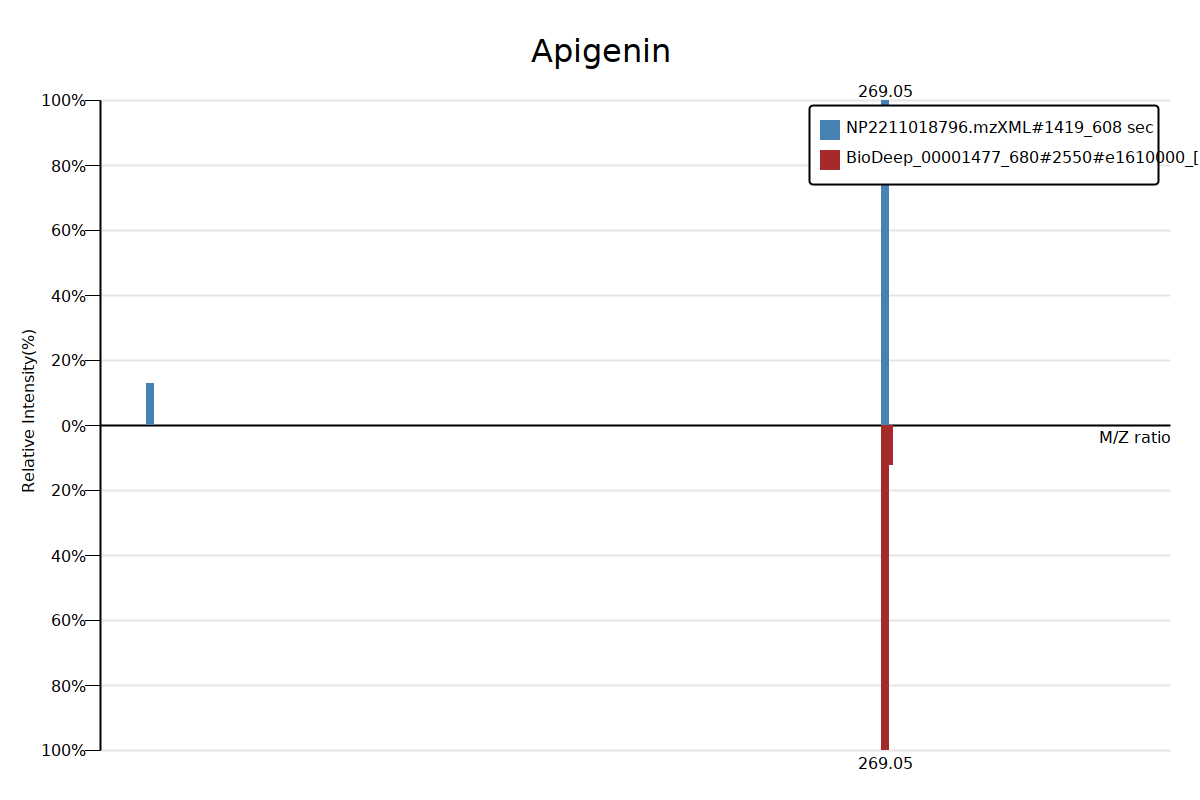

Supplement: Supplementary file 5 [file DataSheet1.ZIP › 2 result graphs between the MSMS secondary fragments of each metabolite and the MSMS secondary fragments of the standard substance in the database/Apigenin.png]

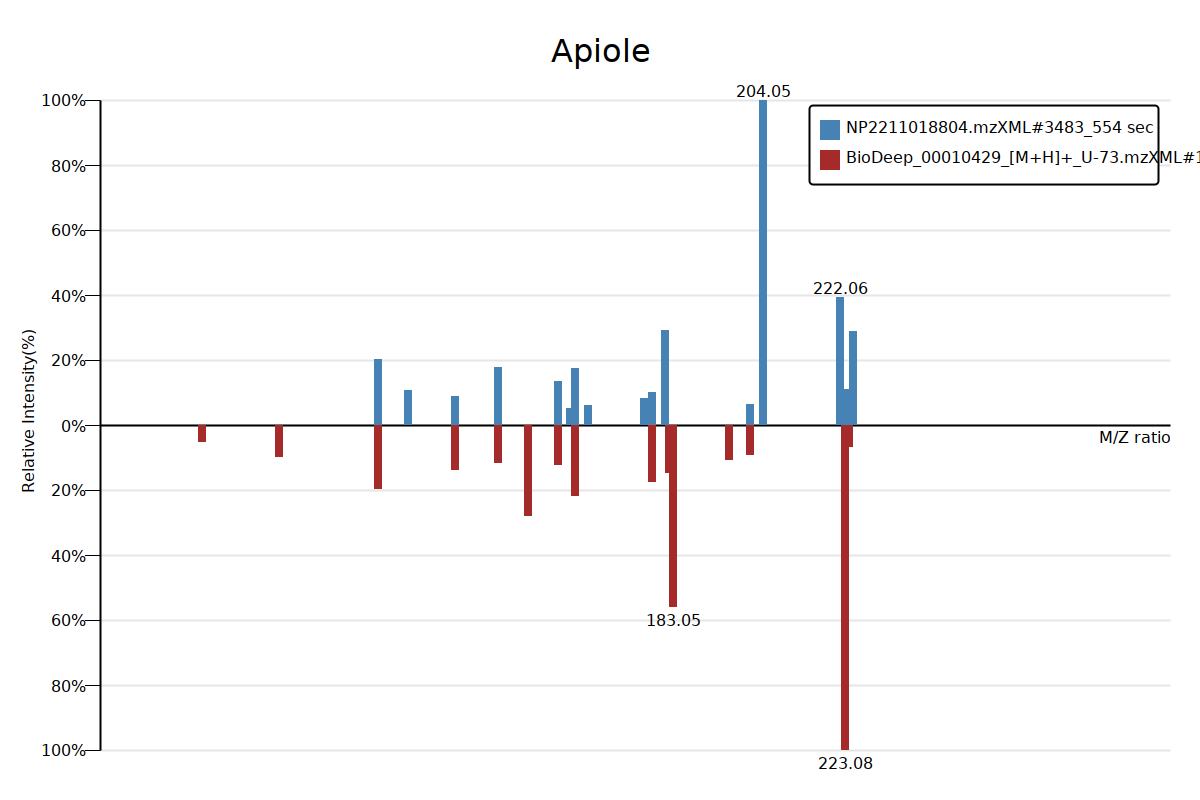

Supplement: Supplementary file 5 [file DataSheet1.ZIP › 2 result graphs between the MSMS secondary fragments of each metabolite and the MSMS secondary fragments of the standard substance in the database/Apiole.png]

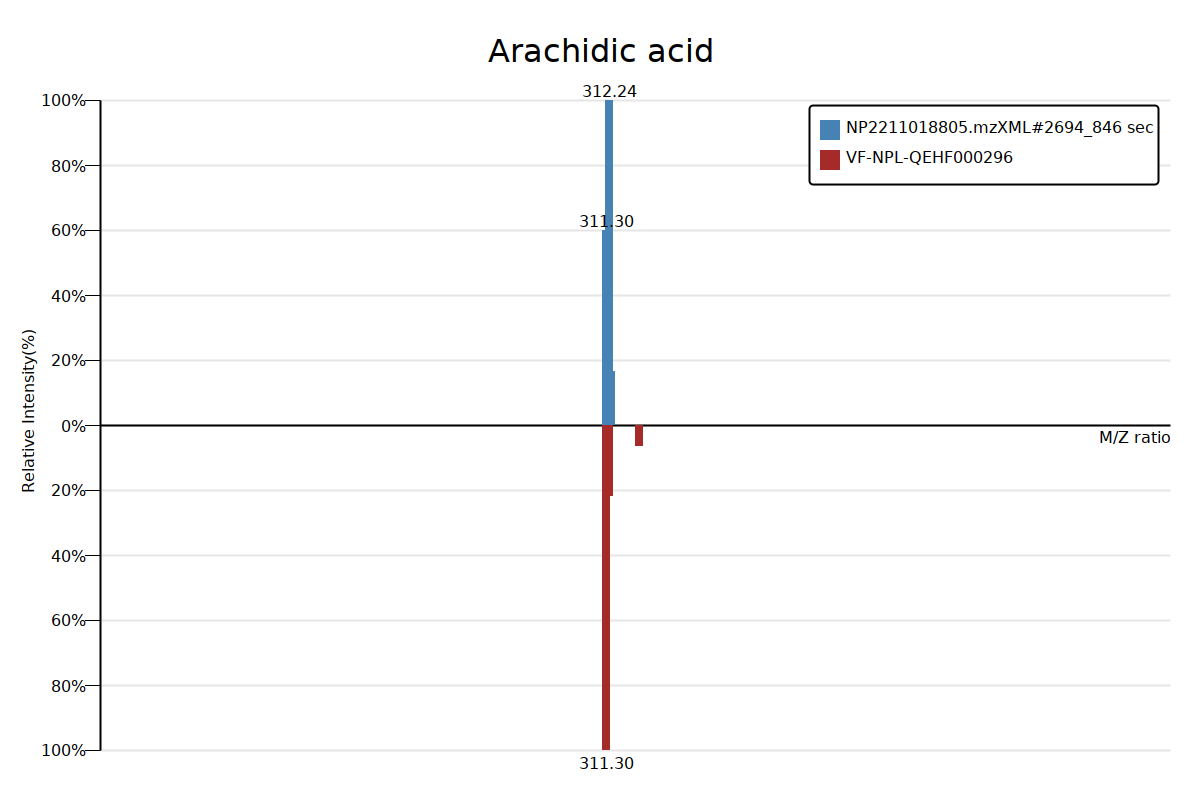

Supplement: Supplementary file 5 [file DataSheet1.ZIP › 2 result graphs between the MSMS secondary fragments of each metabolite and the MSMS secondary fragments of the standard substance in the database/Arachidic acid.png]

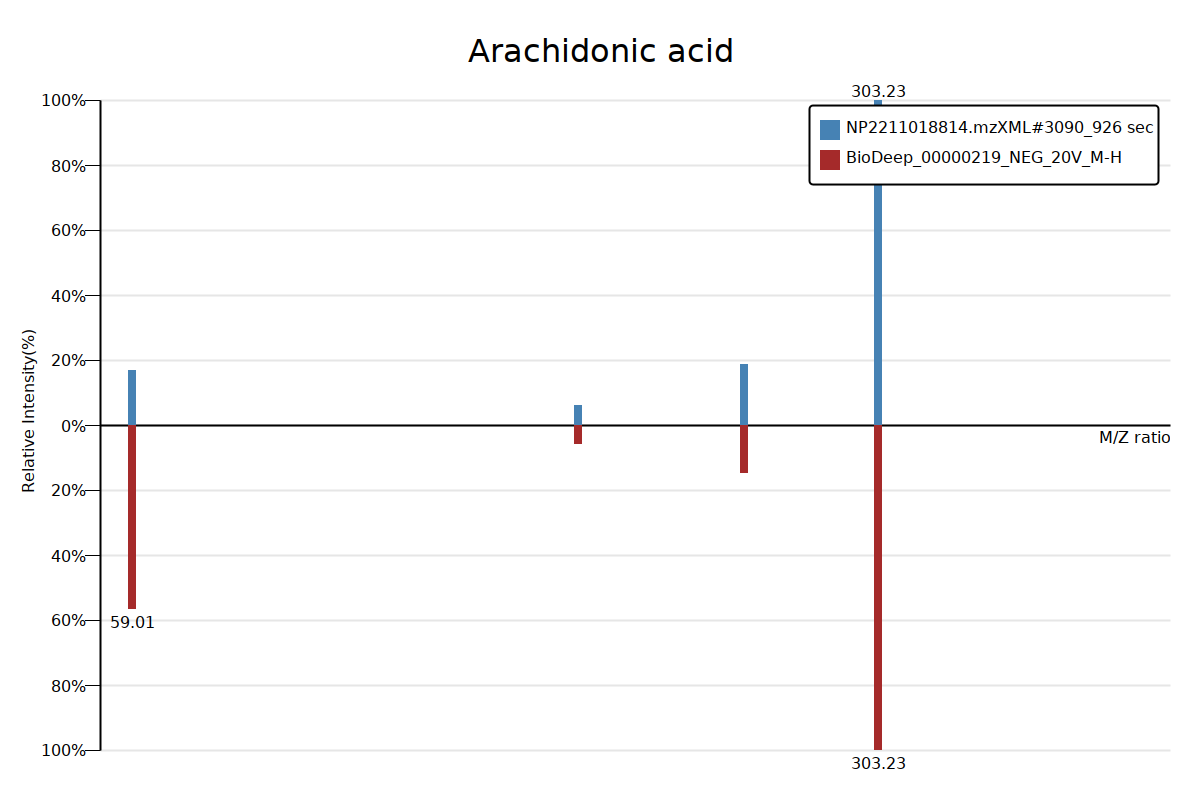

Supplement: Supplementary file 5 [file DataSheet1.ZIP › 2 result graphs between the MSMS secondary fragments of each metabolite and the MSMS secondary fragments of the standard substance in the database/Arachidonic acid.png]

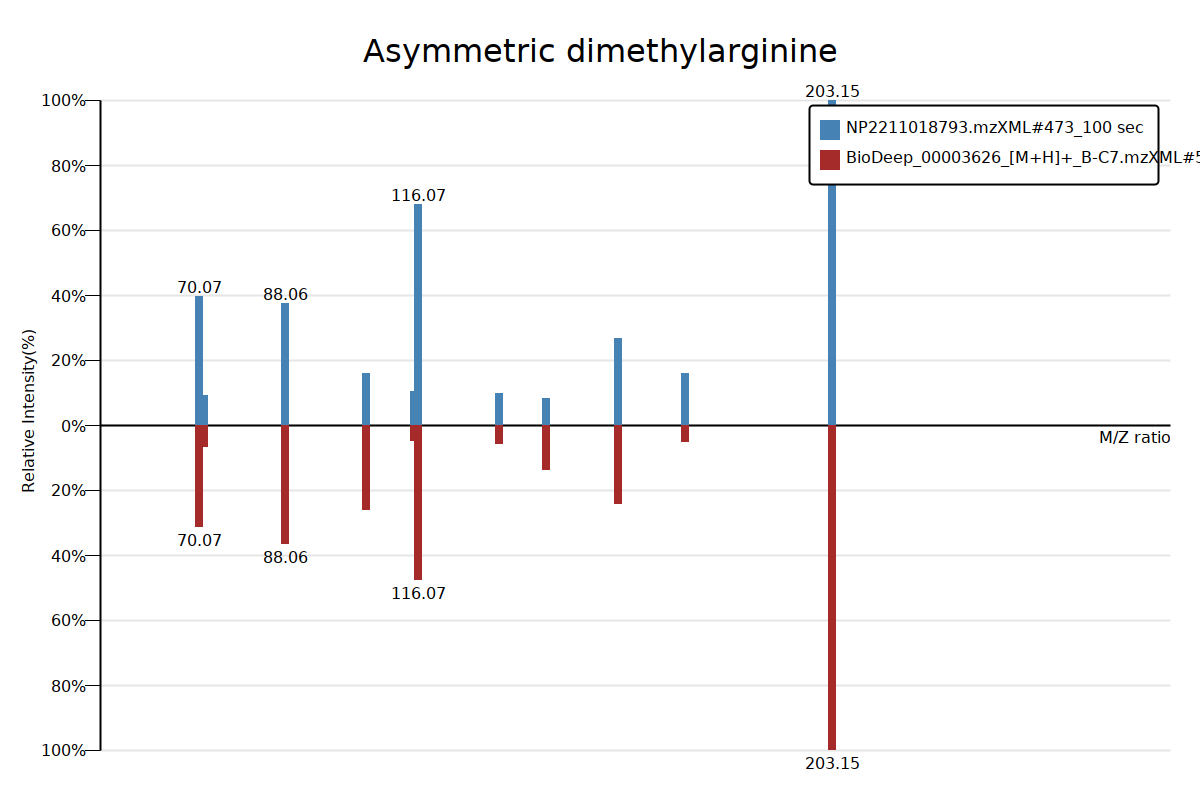

Supplement: Supplementary file 5 [file DataSheet1.ZIP › 2 result graphs between the MSMS secondary fragments of each metabolite and the MSMS secondary fragments of the standard substance in the database/Asymmetric dimethylarginine.png]

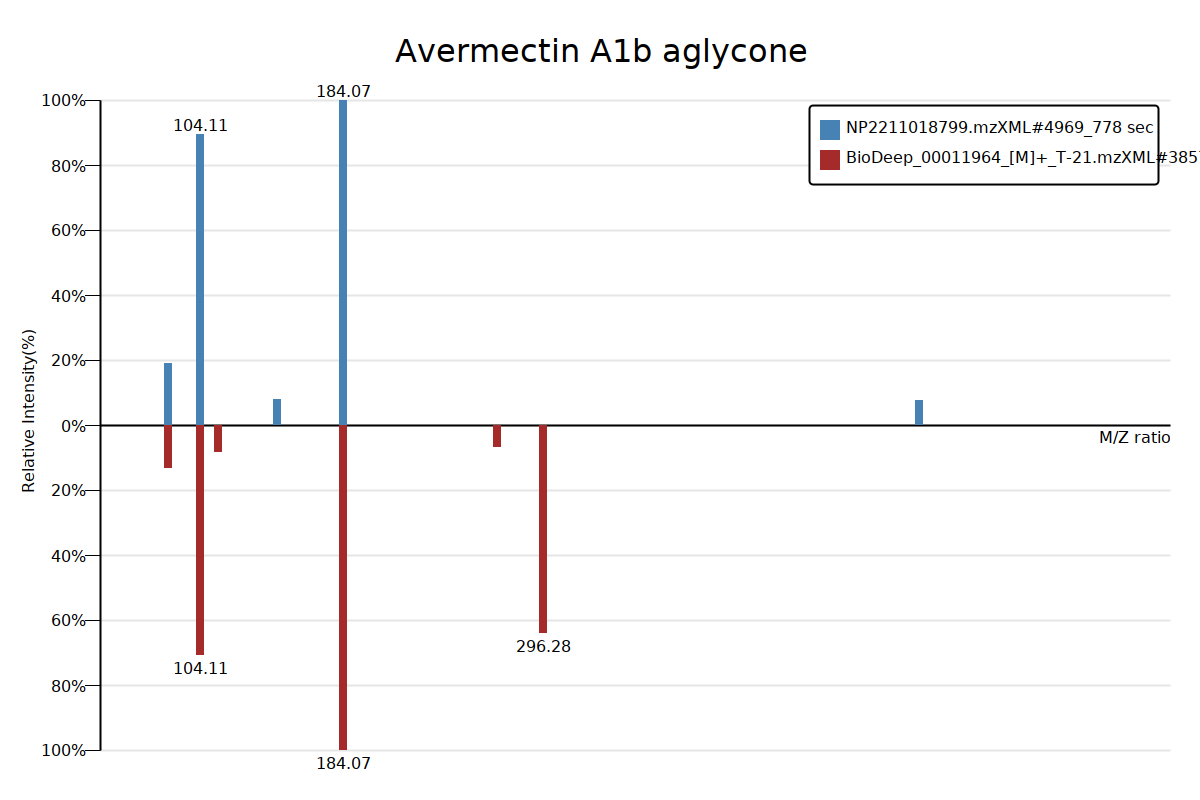

Supplement: Supplementary file 5 [file DataSheet1.ZIP › 2 result graphs between the MSMS secondary fragments of each metabolite and the MSMS secondary fragments of the standard substance in the database/Avermectin A1b aglycone.png]

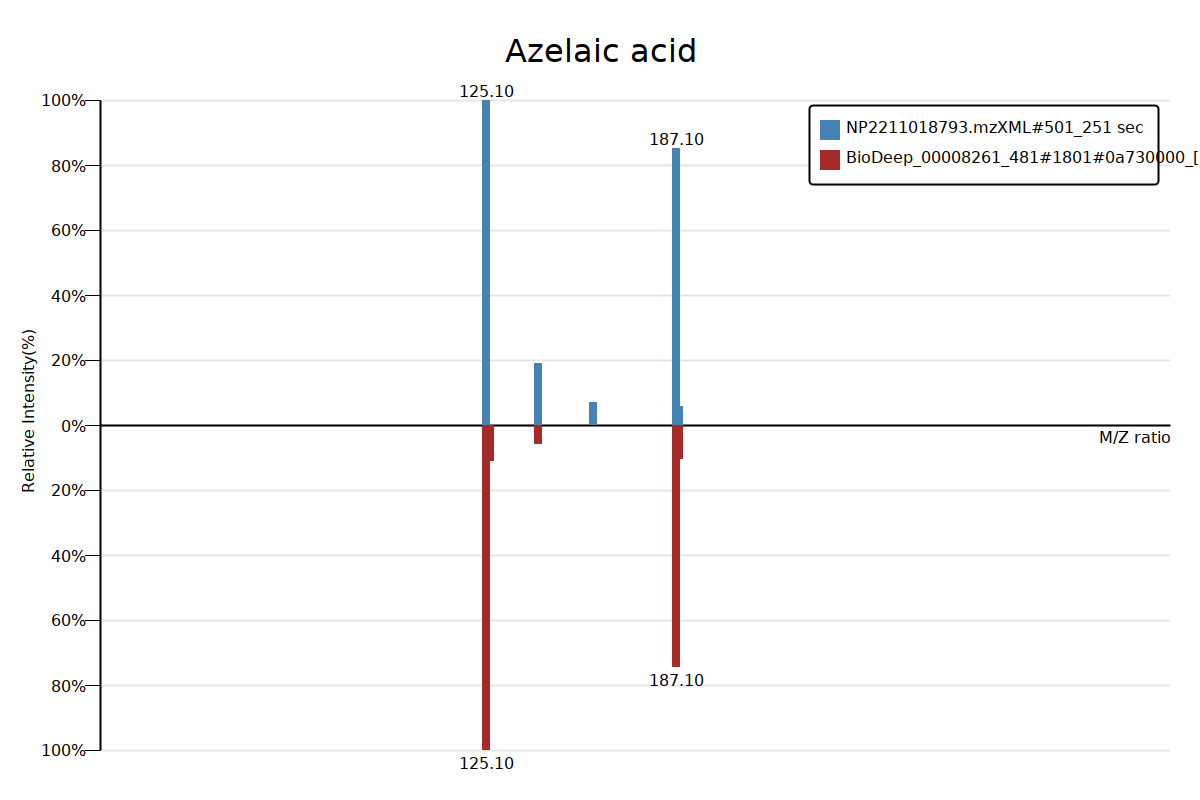

Supplement: Supplementary file 5 [file DataSheet1.ZIP › 2 result graphs between the MSMS secondary fragments of each metabolite and the MSMS secondary fragments of the standard substance in the database/Azelaic acid.png]

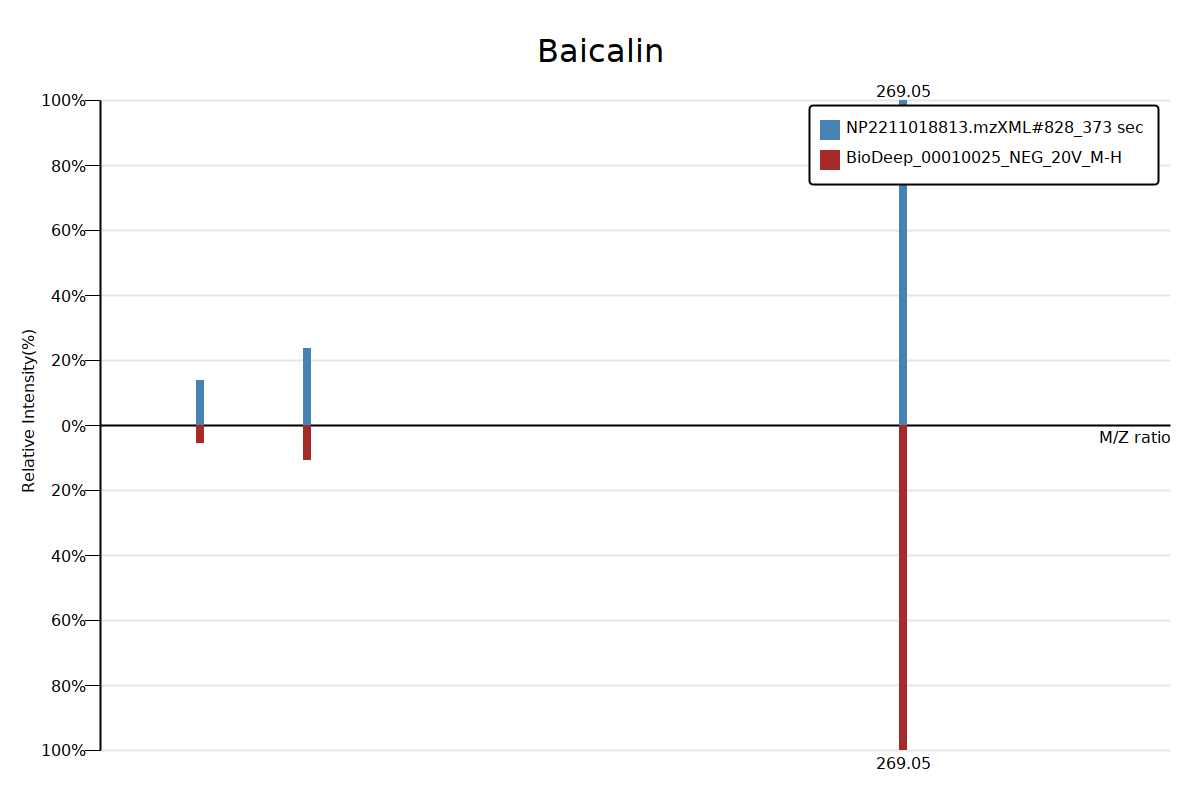

Supplement: Supplementary file 5 [file DataSheet1.ZIP › 2 result graphs between the MSMS secondary fragments of each metabolite and the MSMS secondary fragments of the standard substance in the database/Baicalin.png]

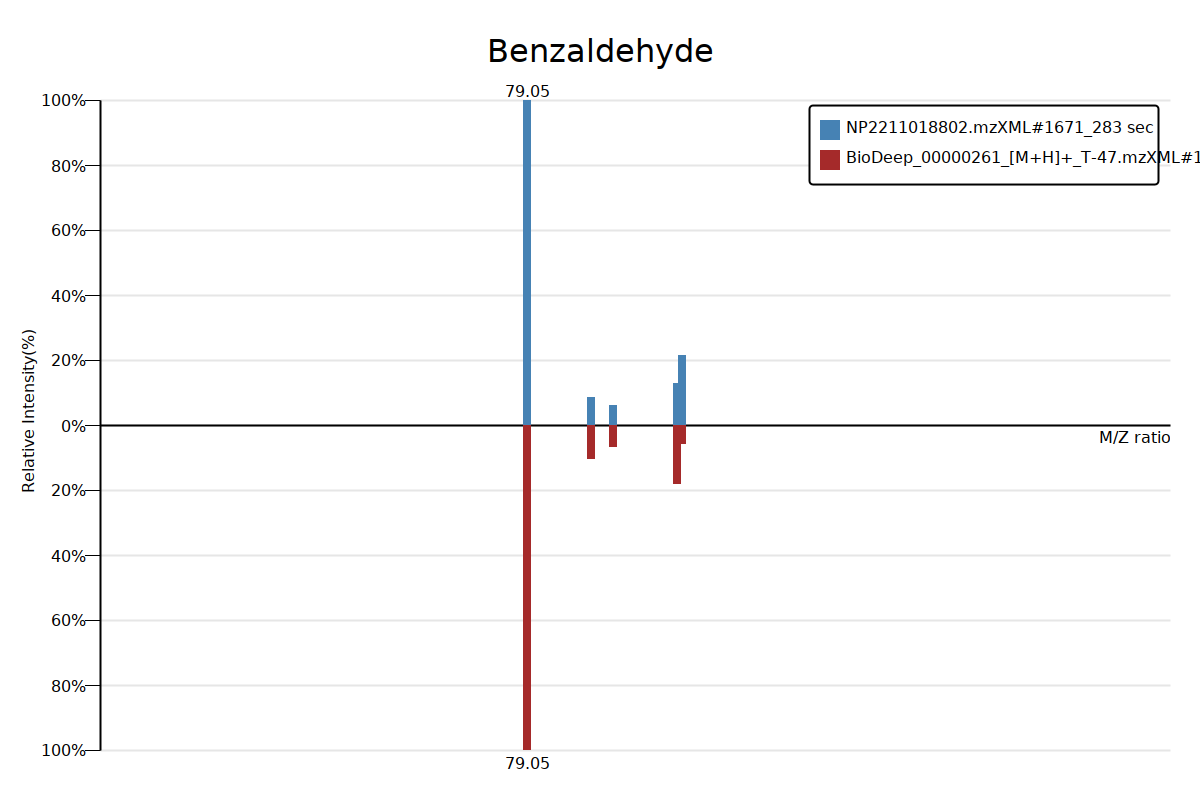

Supplement: Supplementary file 5 [file DataSheet1.ZIP › 2 result graphs between the MSMS secondary fragments of each metabolite and the MSMS secondary fragments of the standard substance in the database/Benzaldehyde.png]

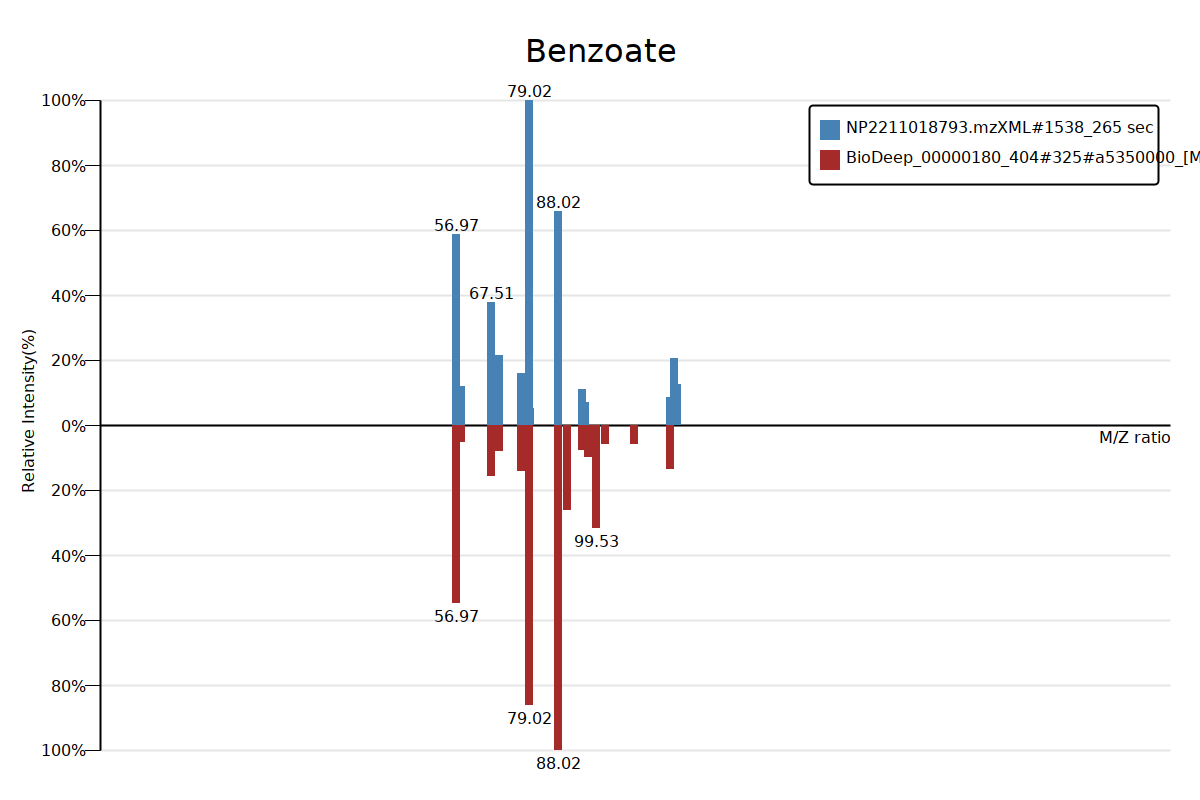

Supplement: Supplementary file 5 [file DataSheet1.ZIP › 2 result graphs between the MSMS secondary fragments of each metabolite and the MSMS secondary fragments of the standard substance in the database/Benzoate.png]

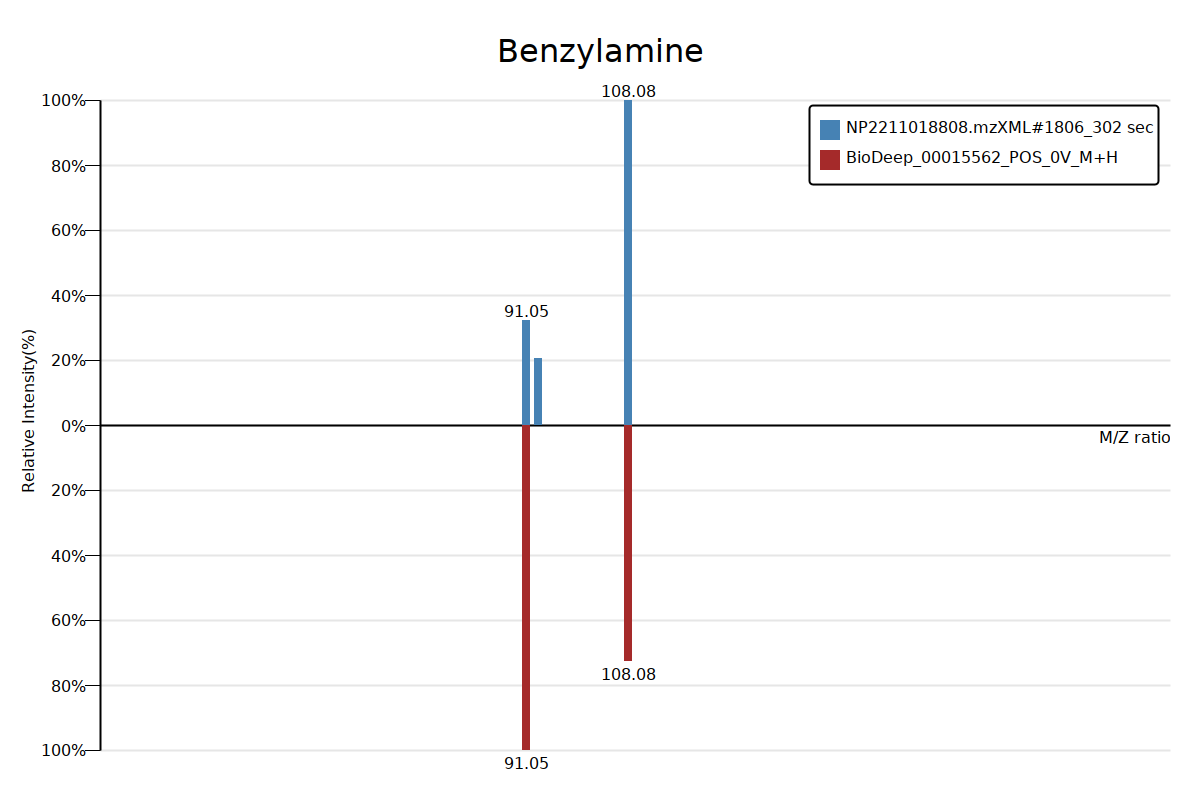

Supplement: Supplementary file 5 [file DataSheet1.ZIP › 2 result graphs between the MSMS secondary fragments of each metabolite and the MSMS secondary fragments of the standard substance in the database/Benzylamine.png]

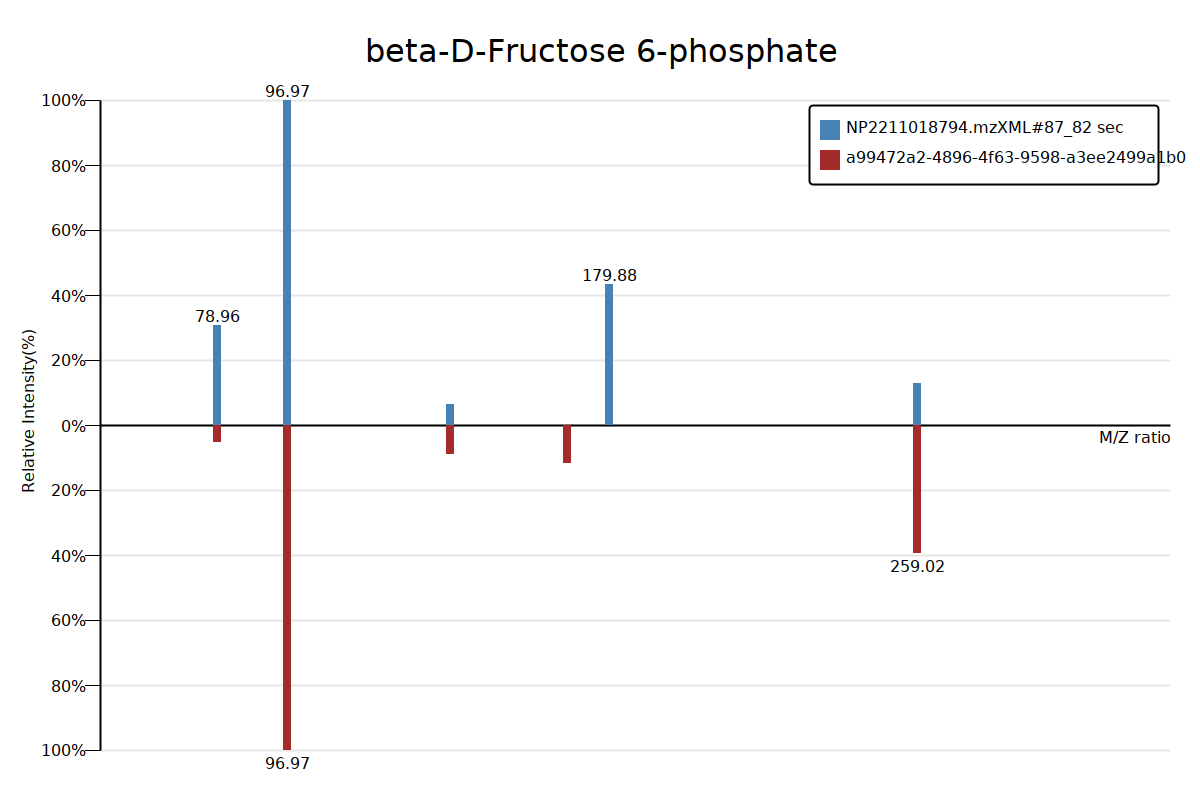

Supplement: Supplementary file 5 [file DataSheet1.ZIP › 2 result graphs between the MSMS secondary fragments of each metabolite and the MSMS secondary fragments of the standard substance in the database/beta-D-Fructose 6-phosphate.png]

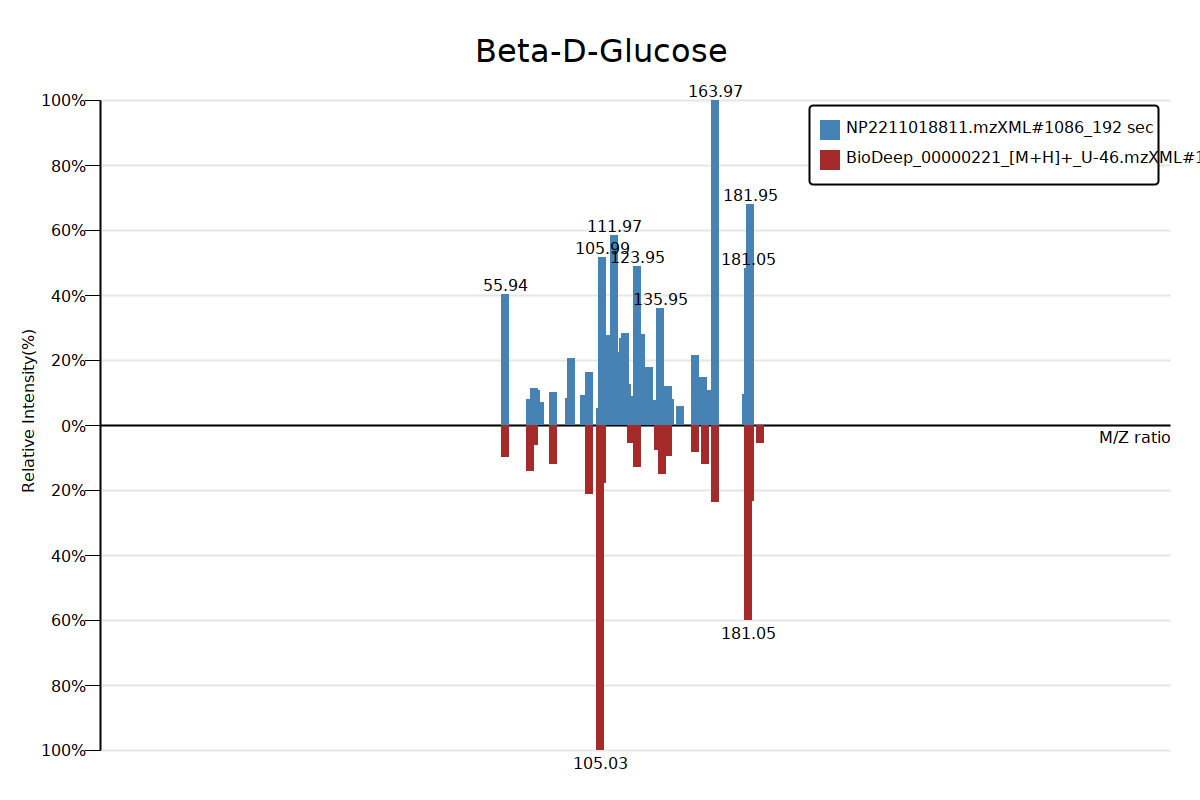

Supplement: Supplementary file 5 [file DataSheet1.ZIP › 2 result graphs between the MSMS secondary fragments of each metabolite and the MSMS secondary fragments of the standard substance in the database/Beta-D-Glucose.png]
